# Azobenzene‐Oxindole Photochromic Dyads

**DOI:** 10.1002/anie.202501872

**Published:** 2025-04-02

**Authors:** Marco Ovalle, Daniel Doellerer, Ben L. Feringa

**Affiliations:** ^1^ Stratingh Institute for Chemistry Center for Systems Chemistry and Zernike Institute for Advanced Materials Faculty of Mathematics and Natural Sciences University of Groningen Nijenborgh 3 Groningen 9747 AG the Netherlands

**Keywords:** Azobenzene, Molecular photoswitches, Oxindole, Photochemistry, Photochromic dyads

## Abstract

Manipulation of molecular geometry using photoresponsive units is a powerful tool in supramolecular chemistry, smart materials, and photopharmacology. Current synthetic chemistry offers many responsive molecules that perform such a task. However, the incorporation of various photoresponsive units in a single molecule to achieve several geometrical changes remains scarce, particularly when they are in close proximity. The development of such systems is limited by challenges arising from selectively addressing the photoresponsive moieties and the analysis of complex mixtures. Here, we overcome these challenges by constructing a novel hetero‐photochromic azobenzene‐oxindole dyad (AOD). Both chromophores can be addressed and quantified in solution by in situ NMR irradiation analysis. Additionally, this method allows us to unravel the intricate photokinetic relationships between the two chromophores, leading to the observation of an unprecedented molecular motion: an azobenzene *E *→ *Z *→ *E* isomerization at a single wavelength due to the oxindole influence. By functionalizing the azobenzene ring, we showed that the responsiveness of the system is maintained in seven distinct AODs. Overall, the photochromic dyad offers dramatic geometrical changes over its four isomers, making it a useful tool for further applications in which such behavior is desired, such as host‐guest systems, responsive materials, photopharmacology, and molecular machines.

## Graphical abstract

A novel photochromic azobenzene‐oxindole dyad is reported. The combination of the two switches promotes drastic molecular geometry changes and allows for precise NMR quantification. This enables the study of the photokinetic behavior under different irradiation conditions and the discovery of a unique photokinetic relationship between the two photoswitches.

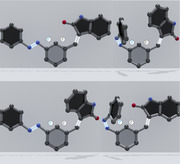

## Introduction

Molecular geometry is a crucial property to key aspects of chemistry, such as molecular recognition^[^ ^1^, ^2^, ^3^, ^4^ ^]^ and self‐assembly.^[^ ^5^, ^6^ ^]^ Molecular switches^[^ ^7^ ^]^ offer a platform to reversibly modulate geometry in a controlled manner. In particular, photoswitches^[^ ^7^, ^8^, ^9^, ^10^, ^11^ ^]^ can reversibly change configuration non‐invasively when stimulated by specific wavelengths, allowing a higher degree of spatiotemporal control over their chemical and physical properties.

In recent years, the regulation of geometry by molecular photoswitches has proved to be a useful tool in a variety of applications. For example, the incorporation of photoswitchable units in biologically active molecules to selectively activate their therapeutic properties is the basis for the emerging field of photopharmacology.^[^ ^12^, ^13^ ^]^ Other applications include the development of structures with intrinsic photoswitches to regulate properties upon light irradiation, such as catalytic activity,^[^ ^14^ ^]^ binding affinity,^[^ ^15^ ^]^ supramolecular assembly,^[^ ^16^ ^]^ dynamic stability,^[^ ^17^ ^]^ and the operation of molecular machines.^[^ ^18^, ^19^, ^20^, ^21^, ^22^ ^]^

The current family of synthetically available molecular photoswitches is large, ranging from well‐established azobenzenes,^[^ ^23^, ^24^ ^]^ spiropyrans,^[^ ^25^ ^]^ and stiff‐stilbenes,^[^ ^26^, ^27^ ^]^ to an ever‐growing collection of photoresponsive compounds with new attractive properties.^[^ ^28^, ^29^, ^30^, ^31^, ^32^, ^33^ ^]^ Despite the development of a variety of molecular photoswitches, the incorporation of distinct photoresponsive units in a single molecule remains limited.^[^ ^34^, ^35^, ^36^, ^37^, ^38^, ^39^ ^]^ A major challenge in this regard is to selectively address the different chromophores as well as the real‐time quantification. In fact, most of the analyses are performed by qualitative methods, and precise quantitative information of the generated states is rarely reported. This limits the study of the photokinetic behavior of multiresponsive systems and the exploration of multi‐addressable systems.

The design of multiswitchable compounds is not as simple as just combining two photoswitches, since the interactions of the different chromophores can vary from being detrimental to the system, leading to inhibition of the formation of expected states, to the display of photosynergetic properties.^[^ ^40^, ^41^ ^]^

One strategy to successfully obtain a multiswitchable orthogonal response is to incorporate two different photoswitches that operate under different stimuli, either as an intermolecular system or covalently linked by a long chain. Previous examples reported by our group include the use of an intramolecular system of an azobenzene and donor–acceptor‐Stenhouse‐adducts (DASA) dyad covalently linked by a long alkyl chain in solution^[^ ^42^ ^]^ and the azobenzene‐spiropyran dyad embedded in a porous network.^[^ ^43^ ^]^ However, when the photoactive units are in close proximity, their individuality is compromised and selective photoswitching becomes challenging.^[^ ^44^, ^45^, ^46^ ^]^ In fact, the “*meta* rule” has been established as an important element to design decoupled photoswitches.^[^ ^47^ ^]^

Our group has been studying the oxindole chromophore in switches^[^ ^48^ ^]^ and motors^[^ ^49^, ^50^, ^51^ ^]^ due to their tunability, robust photochemical behavior, potential biological applications, and ease of synthesis. Oxindole‐based photoswitches can reversibly undergo geometrical *E *→ *Z* isomerization, where both states are thermally stable and can be addressed by wavelengths from UV to visible and, in the case of molecular motors, to IR (via a two‐photon absorption pathway).^[^ ^51^ ^]^

**Figure 1:**
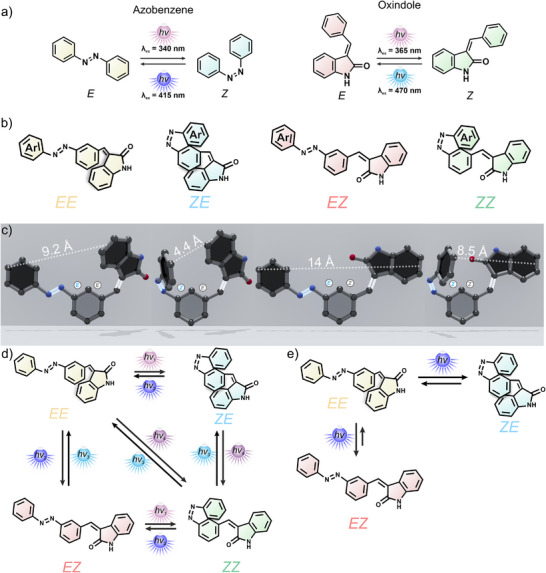
Heterochromic azobenzene‐oxindole dyad (AOD). a) Geometrical photoisomerization of azobenzenes (left) and oxindoles (right). b) Different AOD photoisomers and c) their corresponding calculated three‐dimensional representation (r2scan‐3c method). The dashed lines indicate the distance between the most distant aromatic rings. d) Conventional switching in a multiswitchable system where different wavelengths populate different isomers. e) Newly discovered responsive behavior reported in this work. A single wavelength triggering two photochemical equilibria. First, the ***ZE*** isomer is populated from pristine ***EE*** as this reaction occurs faster. However, the slower conversion of ***EE*** into ***EZ*** ultimately favors the population of this isomer. As a consequence, the ***ZE*** isomer is transiently populated under a single stimulus, and the azobenzene moiety undergoes an *E *→ *Z *→ *E* isomerization in the global process ***EE** *→ ***ZE** *→ ***EZ***.

Here, we combine the emerging oxindole photoswitch explored in our group with the well‐known azobenzene to form the heterochromic azobenzene‐oxindole dyad ((AOD), Figure 1a,b). Both responsive fragments are attached to the same aromatic ring via a *meta* connection (following the “*meta* rule”)^[^ ^47^ ^]^ to enhance selective addressability. This results in four isomers with dramatic geometrical differences (Figure 1b,c), generating strong ring current effects^[^ ^52^ ^]^ that allowed us to identify key ^1^H NMR signals for real‐time quantification. This was complemented with UV–visible absorption measurements and density functional theory (DFT) calculations.

Interestingly, we discovered that besides the expected photoisomerization reactions (Figure 1d), the photokinetic relationships in the dyad can promote new processes. In particular, an unprecedented azobenzene *E *→ *Z *→ *E* isomerization using a single wavelength was observed. We attribute this behavior to the influence of the oxindole within the coupled photoreaction network (Figure 1e). We believe that this behavior might be common in multiphotoswitchable systems; however, measuring the kinetic profile instead of performing the general approach to measure these systems at the beginning and end of the irradiation process made it elusive.

We envision that this versatile photochromic dyad and the newly discovered behavior to precisely control several states will be key to the development of new responsive systems in which, besides a high control in molecular geometry, more complex and multifunctional operations are required. Leading to important advances in actuating self‐assembled structures, reticular materials, photopharmacology, and molecular machines.

## Results and Discussion

### Synthesis

**Scheme 1:**
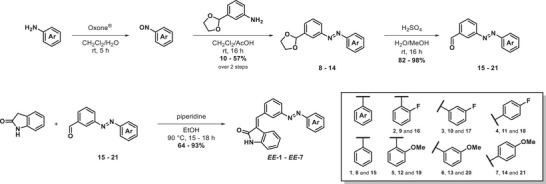
Synthetic schemes to obtain the heterochromic AODs **1**–**7**.

The development of the AODs started by the synthesis of a series of protected azobenzenes **8–14** in two steps (Scheme 1, see Supporting Information for detailed reaction conditions). First, the aniline was oxidized to the corresponding substituted nitroso compound with Oxone in a dichloromethane/water mixture at room temperature. The nitroso compound was then subjected to a Mills reaction with the respective ethylene glycol protected aniline in dichloromethane and acetic acid at the room temperature, resulting in the protected azobenzenes **8–14**.^[^ ^53^ ^]^ Sequential deprotection with sulfuric acid in a water/methanol mixture resulted in the aldehyde substituted azobenzenes **15–21** (Scheme 1).^[^ ^54^ ^]^ Subsequent reaction with oxindole via a Knoevenagel condensation facilitated by piperidine in ethanol yielded the final heterochromic AODs **1–7** in their ***EE*** isomeric form (Scheme 1).^[^ ^48^ ^]^ Additionally, AODs **1** and **7** were obtained in their ***EZ*** configuration by bulk irradiation in a photoreactor (*λ*_irr_ = 420 nm).

### Photoresponsive Behavior

#### In Situ ^1^H NMR Irradiation and Quantification

**Figure 2:**
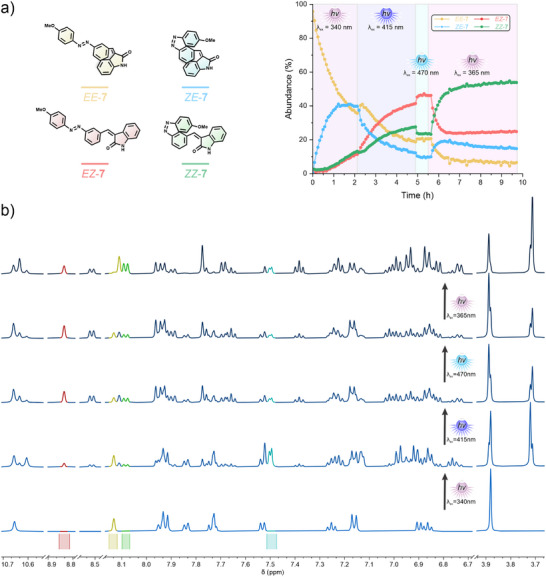
^1^H NMR ((CD_3_)_2_SO, 25 °C) a) Kinetic traces of the different isomers of seven obtained by following the in situ ^1^H NMR irradiation sequence *λ*_irr_ = 340 → 415 → 470 → 365 nm. b) Stacked spectra of the in situ irradiation of seven following the wavelength irradiation sequence (from the pristine sample at the bottom to the top) *λ*_irr_ = 340 → 415 → 470 → 365 nm. The characteristic signals used to follow the kinetic traces are shown color‐coded accordingly (***EE*** **‐** **7** yellow, ***ZE*** **‐** **7** blue, ***EZ*** **‐** **7** red, ***ZZ*** **‐** **7** green). Video S1 shows the ^1^H NMR spectra evolution.

Precise quantification of the isomeric distribution over a time evolution was possible due to the identification of non‐overlapping key ^1^H NMR signals (representative AOD **7** is shown in Figure 2 and Video S1, AODs **1**–**6** are shown in supporting Figures S1–S7 and Videos S2–S7). These signals obey the characteristic behavior of oxindole and azobenzene switching, in which the ring current effect is manifested as dramatic changes in proton signals over a wide ppm range due to the variation of the position of the aromatic rings. Additionally, the assignment of the different isomers to their respective key signals was performed by analysis of the kinetic profile. Since it is expected that single switched isomers ***EZ*** and ***ZE*** appear before the doubly switched ***ZZ*** isomer when both photoswitches are addressed (*λ*_irr_ = 365 nm), the later isomer can be unequivocally assigned by order of appearance (direct conversion of pristine ***EE*** into ***ZZ*** is not possible). The signal of the ***EZ*** isomer can be attributed to the appearance of a deshielded singlet around 9.00 ppm, characteristic for the oxindole *E *→ *Z* isomerization. This isomer is thermically stable, and further isolation after bulk irradiation and heating for representative compounds **1** and **7** confirmed the assignment of the ***EZ*** isomer. Finally, the remaining key signal was attributed to the ***ZE*** isomer.

The identification of these key signals allowed us to study the photokinetic behavior of the AODs under different wavelengths of irradiation. First, we performed the irradiation sequence *λ*_irr_ = 340 → 415 → 470 → 365 nm for AOD **1**–**7** in (CD_3_)_2_SO at 25 °C (Figures 2 and S1–S7 and Videos S1–S7). Typically, the azobenzene *E* → *Z* photoisomerization can be addressed with UV light in a range of ∼310 to 365 nm, while the oxindole needs higher wavelengths of around ∼365 to ∼450 nm. Thus, the first irradiation was expected to favor the formation of the ***ZE*** isomer. In general, all AODs showed selective azobenzene isomerization upon 340 nm irradiation, achieving an abundance of the ***ZE*** isomer that ranges from ∼30% to ∼50%, with AOD **2** and **4** showing the highest selectivity (fluorine substituted in *ortho*‐ and *para*‐position, respectively).

Subsequently, the irradiation was changed to 415 nm as soon as the change in the kinetic profile was minimal (Figures 2 and S1–S7 and Videos S1–S7). Usually, this wavelength will promote the *Z *→ *E* isomerization of the azobenzene, while it can still address the *E *→ *Z* isomerization of the oxindole. Indeed, for all AODs we see a decrease of the ***ZE*** isomer, accompanied by an increase in the concentration of the ***EE*** isomer followed quickly by the population of the ***EZ*** isomer. In general, this isomer was selectively populated in an abundance of ∼40% to ∼60% for all evaluated AODs. The next wavelength in the investigated sequence was 470 nm. By applying this stimulus, the *E* azobenzene is expected, and the oxindole photoswitch can moderately undergo *Z *→ *E* isomerization.^[^ ^48^ ^]^ For AODs **1**–**7**, this wavelength only promoted minor changes in the photoisomeric distribution. Finally, 365 nm wavelength irradiation was performed to promote both azobenzene and oxindole *E *→ *Z* isomerization processes. This stimulus resulted in the largest differences with respect to the isomeric distribution within AOD **1**–**7**. For AODs **1**–**4** (Figures S1–S4), a minor increase in the abundance of the ***ZZ*** isomer from ∼30% to ∼40% was observed, accompanied by a decrease of ***EE***. For the methoxy‐substituted AODs **5**–**7** (Figures 2, S5, and S6), the ***ZZ*** form was indeed populated from ∼40% up to ∼55%.

**Figure 3:**
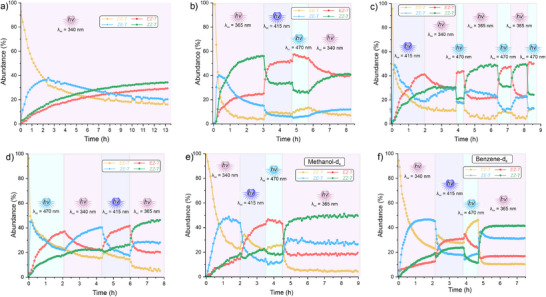
Operation of AOD **7** under different conditions and irradiation sequences. a) Continuous irradiation using 340 nm light. Irradiation of pristine ***EE*‐7** using the sequences in DMSO b) 365 → 415 → 470 → 340 nm, c) 415 → 340 → 470 → 365 → 470 → 365 → 470 nm, and d) 470 → 340 → 415 → 365 nm. Irradiation of pristine ***EE*‐7** using the sequence 340 → 415 → 470 → 365 nm in different solvents; e) methanol‐d_4_ and f) benzene‐d_6_.

The use of methoxy substituted AODs **5**–**7** (Figure 3e–g) enables the transition between the four possible isomers with optimal selectivity, leading to the preferential population of the desired isomer as the main compound in solution upon irradiation at the appropriate wavelength. In particular, the *para*‐methoxy AOD **7** (Figure 3g) showed the best performance, allowing the transitions from the pristine ***EE*** isomer to the ***ZE*** photoisomer in 40% yield, converting this into the most abundant species after irradiation. The subsequent irradiation with visible light (*λ*_irr_ = 415 and 470 nm) promotes the ***ZE*** photoisomer as the main component in solution, with an abundance of ∼55% to finally favor the ***ZZ*** form up to 55% upon 365 nm irradiation.

Next, we performed an in‐depth analysis of AOD **7** under different conditions. First, we studied the prolongated irradiation at 340 nm for 13.5 h (Figure 3a and Video S8). It was observed that after the 3 h mark, the photostationary state (PSS) at this wavelength (PSS_340_) was not reached, but rather the spectra continued changing, favoring the increase in abundance of ***EZ*** and ***ZZ*** (30% and 35%, respectively) with the latter being the main species. This experiment indicates that the previously observed selectivity for the ***ZE*** isomer is due to the interruption of the irradiation and that the oxindole *E *→ *Z* isomerization is very slow in comparison. However, this isomer is maintained as the main compound in the mixture for a period of ∼3 h, and its abundance only drops from 38% to 29%.

Next, we irradiated pristine ***EE*‐7** with 365 nm light (Figure 3b, Video S9). At this wavelength, both photoswitches are expected to undergo *E *→ *Z* isomerization, and by following the evolution of the ^1^H NMR spectra, one can identify which isomerization occurs first. We observed the sequential switching of the azobenzene generating ***ZE*‐7** to a maximum of 40%. Half an hour later, the oxindole photoisomerization of this species takes place as a second step to generate ***ZZ*‐7**, which ends up being the most abundant compound (58%). In fact, the composition of the mixture after 3 h of irradiation with 365 nm is the same as the one reached previously, indicating that PSS_365_ was reached. Subsequently, we changed the irradiation wavelength to 415 nm for ∼1.5 h during the same experiment. The ***EZ*** isomer quickly becomes the main isomer in solution, with an abundance of 52%, as the simultaneous azobenzene *Z *→ *E* and oxindole *E *→ *Z* isomerizations are favored. Interestingly, reaching PSS_415_ was faster than in the previous experiment (Figure 2a, Video S1) in which 340 nm light was used first and isomers ***EZ*** and ***EE*** were the most abundant, opposite to this experiment in which the ***ZE*** isomer is the main isomer in solution. Subsequent irradiation at 470 nm promoted again only minor changes in the composition of the mixture. Finally, irradiation with 340 nm light promotes the slow *E *→ *Z* azobenzene isomerization.

When pristine ***EE*‐7** is irradiated with visible light, either at 415 or 470 nm (Figure 3c–d, Videos S10 and S11), an interesting phenomenon occurs. Just like in the previous experiments, the ***EZ*** isomer is favored; however, this occurs only after ∼40% of the ***ZE*** isomer is generated. It is important to note that a direct transformation from ***ZE*** to ***EZ*** is not possible, but it rather requires a stepwise isomerization of both photoswitches. This experimental evidence indicates that, instead of the direct conversion from ***EE*** to ***EZ*** via oxindole *E *→ *Z* isomerization, an unexpected pathway in which the azobenzene *E *→ *Z* isomerization takes place first and the ***EE *** ↔ ***ZE*** equilibrium is reached. Subsequently, the oxindole undergoes the slow *E *→ *Z* isomerization ***EE *** → ***EZ***, affecting the previously mentioned equilibria. As a consequence, the first equilibrium is reached faster, which explains the fast population of ***EZ***, followed by the slow equilibrium of the second process, which drains the ***ZE*** isomer in favor of ***EZ*** via ***EE***. In fact, regardless of the wavelength used, the azobenzene *E *→ Z isomerization occurs first, even when the *Z* azobenzenes are the minor species at PSS, which means that isomer ***ZE*** acts as a “gatekeeper” for all the other transformations. Resulting in a photochemically coupled reaction network that allows, to the best of our knowledge, the first azobenzene *E *→ *Z *→ *E* isomerization under a single‐wavelength stimulus. We modeled the 470 nm irradiation of pristine ***EE*‐7** (Figure 3f) to a network of first order reactions (Figure S8). The difference between the coupled reaction network intrinsic of the AOD is evident when compared to an uncoupled system modeled with the same reaction constants. We propose that the azobenzene isomerization occurs faster due to the characteristic high molar absorption (*ε* = 11400 M^−1^cm^−1^, 340 nm in acetonitrile) and quantum yield (*Φ* = 0.15, 340 nm in acetonitrile, see Supporting Information for details) by comparison to that of the oxindole (*ε* = 4200 M^−1^cm^−1^, 365 nm in acetonitrile, *Φ* = 0.1).^[^ ^48^ ^]^ This observed behavior reveals an important design feature for future multiple switching systems in which the kinetic behavior could be engineered to perform specific switching events, operating beyond the traditional PSS approach.

When irradiation with a 340 nm LED is performed after irradiation with 415 nm (Figure 3c, Video S10), we observe again the slow azobenzene *E *→ *Z* isomerization. On the contrary to our previous observation, subsequent irradiation at 470 nm promotes major changes in the photoisomeric distribution favoring ***EZ*** up to 43%. Next, two cycles of 365 and 470 nm irradiation were executed to change the main species in solution from ***ZZ*** and ***EZ***, respectively. Indeed, we observed that this stimulus allows for a photostationary distribution (PSD) ***EE***: ***ZE***: ***EZ***: ***ZZ*** of 5:23:23:49 (PSD_365_) and 12:13:49:26 (PSD_470_).

Next, we performed the irradiation sequence *λ*_irr_ = 470 → 340 → 415 → 365 nm each for (Figure 3d, Video S11). After the 470 nm irradiation period described above, 340 nm irradiation promoted the azobenzene *Z *→ *E* isomerization as expected, favoring the transition from 37% of ***EZ*** as the main isomer in solution to the ***ZE*** in an abundance of 40%. Subsequent irradiation with 415 nm light promoted the population of the ***EZ*** isomer to 42% and finally 365 nm irradiation promoted ***ZZ*** as the main isomer in a 47% abundance.

Finally, we performed the irradiation sequence *λ*_irr_ = 340 → 415 → 470 → 365 nm in methanol (Figure 3e, Video S12) and benzene (Figure 3f, video S13). In the first solvent, we observed a similar behavior as in the case of DMSO; however, in benzene (Figure 3e, Video S13), the behavior of the AOD did change dramatically. After the initial irradiation at 340 nm favoring the ***ZE*** isomer (46%), the subsequent irradiation with 415 nm light did not favor the ***EZ*** (31%) isomer as much as in DMSO or methanol. The main difference occurs when the mixture is irradiated with 470 nm light. Unlike the previous examples, the ***EE*** isomer is populated as the main isomer in solution with an abundance of 47%, indicating a successful oxindole *Z *→ *E* isomerization in this solvent. Finally, 365 nm irradiation favored the ***ZZ*** isomer in a 42% abundance.

The kinetic profiles allowed us to assign the different signals of the spectra to the corresponding photoisomer. We fitted the different signals to the linear combination of the previously identified kinetic profiles (Figures S9–S14). Additionally, a series of ex situ irradiations were performed to test the operation of the AOD without continuous monitoring (Figures S9–S15).

#### UV–Visible Absorption Studies

The UV–vis absorption spectra of AOD **7** were measured and compared with the corresponding parent azobenzene **21** and the unsubstituted oxindole switch (**Ox‐S**; Figure 4). The same experiments were made for the rest of AOD **1**–**6** (Figures S16–S64). In general, the pristine ***EE*** isomers show π → π* bands observed in both individual chromophores overlapping around 340 nm, except for the methoxy derivatives substituted in *ortho*‐ and *para*‐positions ***EE*‐5** and ***EE‐*7**, in which the π → π* bands experience a bathochromic shift of around 40 nm. The red‐shifted shoulder of **Ox‐S** ^[^ ^48^ ^]^ overlaps with the n → π* band of the azobenzene in the visible range. Thus, no evident band separation is observed in the AOD system between the two chromophores.

**Figure 4:**
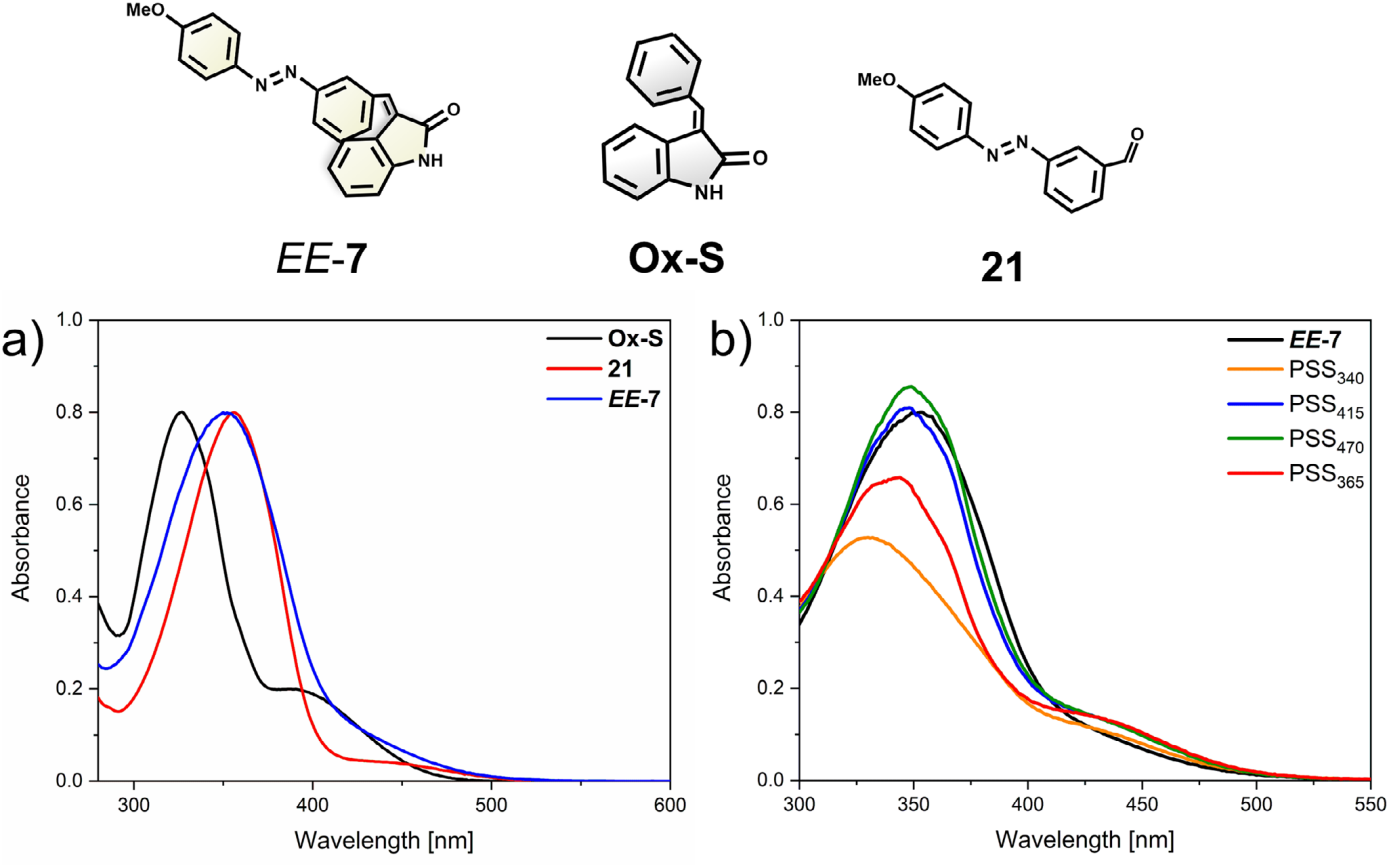
a) Normalized absorption spectra (DMSO at 20 °C) for the parental oxindole‐based switch (**Ox‐S**; black), the azobenzene precursor (**21**; red), and ***EE*‐7** (blue). b) Normalized UV–vis spectra of ***EE*‐7** in DMSO at 20 °C upon sequential irradiation with *λ*_irr_ = 340, 415, 470, and 365 nm to the respective PSSs.

UV–vis measurements support our findings that when the ***EE*** isomer of each AOD **1–7** is irradiated with 340 nm light, the azobenzene moiety is selectively addressed due to a low rate of switching of the C═C double bond. This leads to an increase of the abundance of the ***ZE*** isomer, which is represented by a fast decrease of absorption around 350 nm. Subsequent irradiation with a 415 nm LED favors the formation of the *E* isomer of the azobenzene and the *Z* isomer of the oxindole, which is represented by a fast increase of absorption when the irradiation is started followed by a slow but steady increase originating from the C═C double bond isomerization.^[^ ^48^, ^55^ ^]^ Irradiation with 470 nm resulted just in small changes regarding the absorption, but applying 365 nm light displayed another decrease, which was assigned to the *E* to *Z* isomerization of the azobenzene.

**Figure 5:**
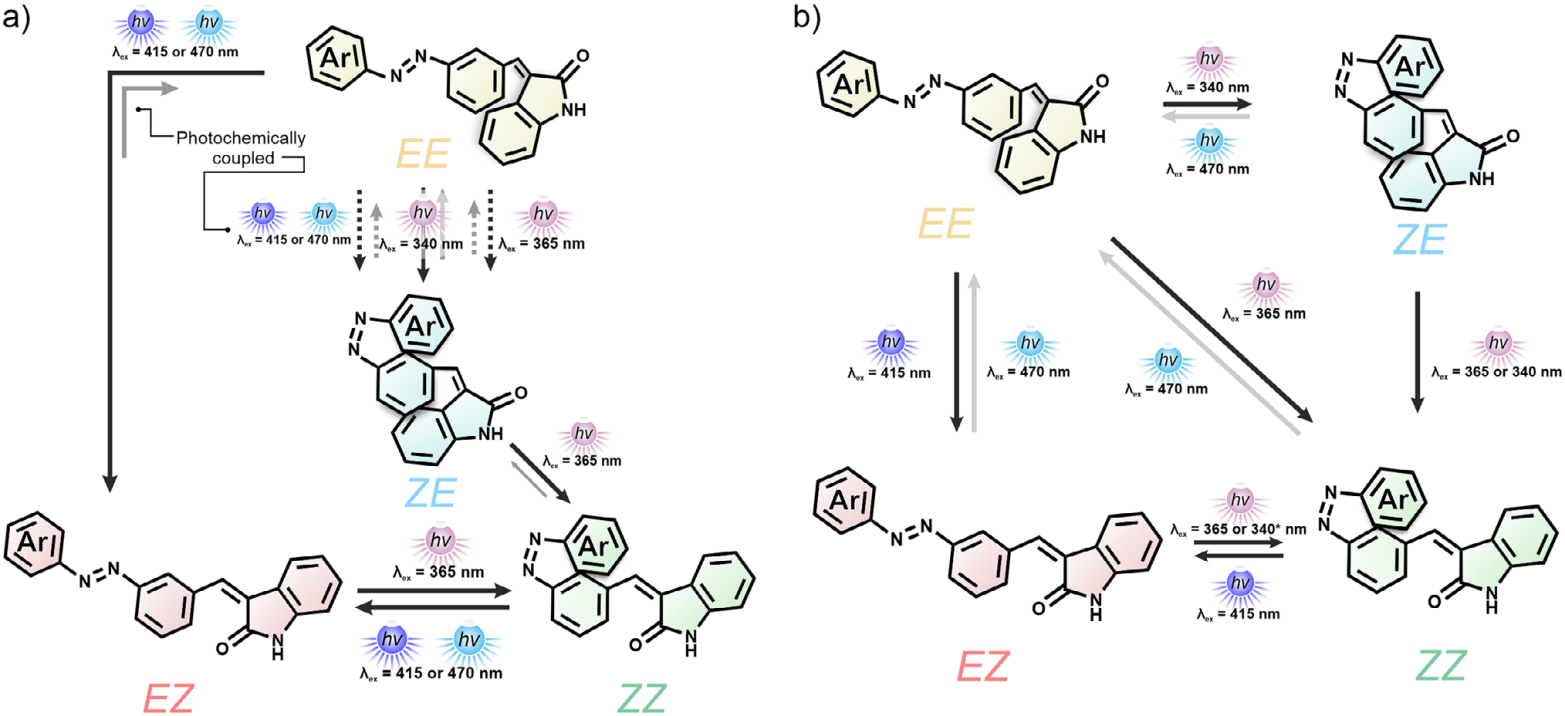
a) Photochemical behavior of the AOD. The dashed lines represent fast reactions in which the equilibria are quickly shifted to favor the next reaction. For the case of visible light irradiation (*λ*_irr_ = 415 or 470 nm) of the ***EE*** isomer, the first reached equilibrium favors isomer ***ZE***, which is observed transiently as the main isomer in solution. Prolonged irradiation with the same wavelength (*λ*_irr_ = 415 or 470 nm) favors the ***EZ*** isomer through the ***EE*** pathway. b) States that can be populated at the PSS or pseudo‐PSS. The grey arrows show the population of the ***EE*** isomer achieved only in benzene.

Based on our observations, the photoresponsive behavior can be described in terms of photokinetics and at the photostationary or pseudo‐PSS. The photokinetic behavior (Figure 5a) is characterized by the coupled reaction triggered by either 415 or 470 nm irradiation and by the population of the ***ZE*** isomer upon 340 nm irradiation, followed by the slow overtaking of the ***ZZ*** and ***EZ*** isomers. This step gives a wide window of time to stop the irradiation while ***EZ*** is the major isomer in solution. We refer to this state as a pseudo‐PSS (Figure 5b).

#### DFT Calculations

**Figure 6:**
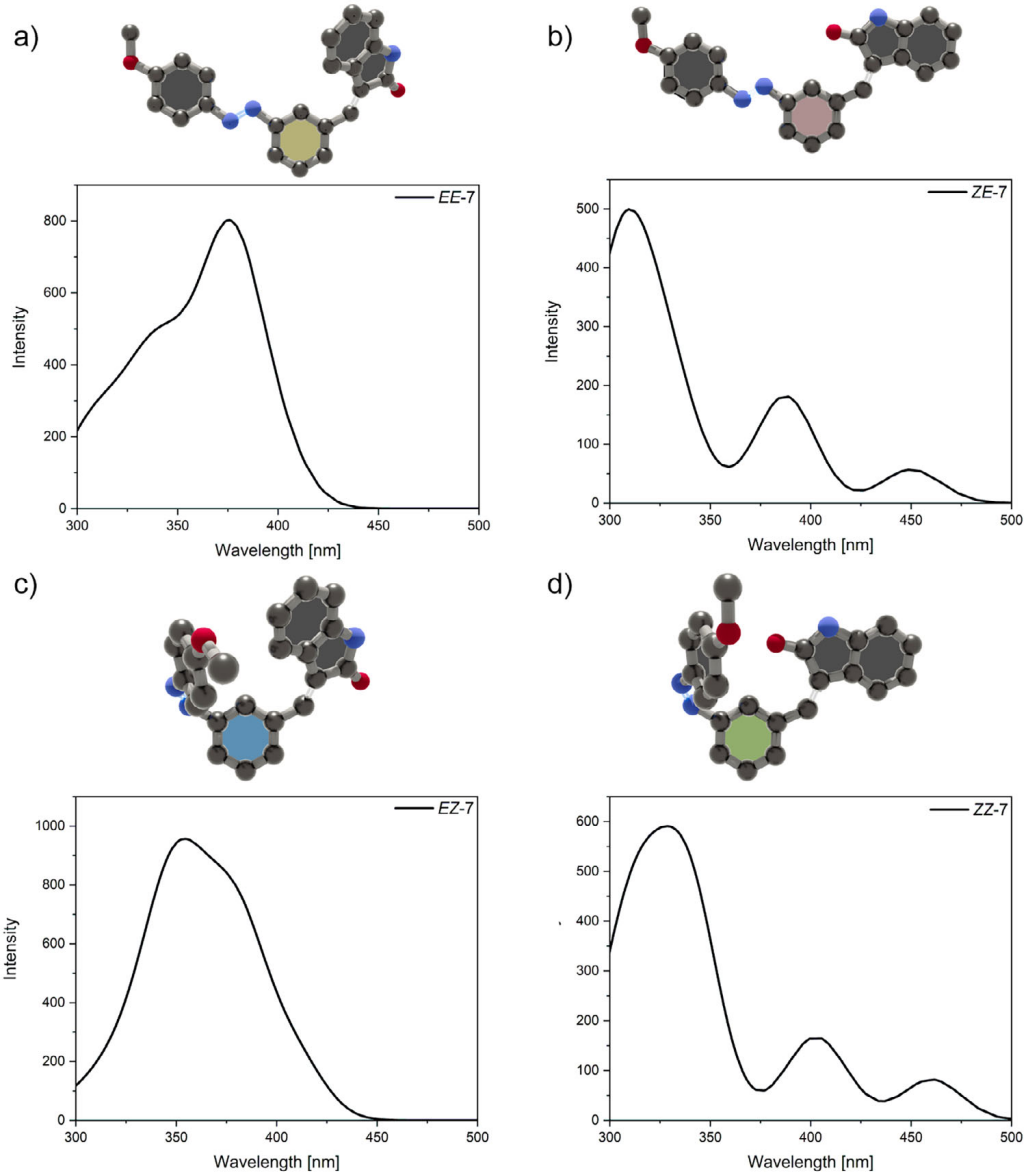
Optimized structures of AOD **7** in all four stable configurations (gas phase; r2scan‐3c method) and simulated UV–vis spectra in DMSO (TD‐PBE0/def2‐TZVP) a) ***EE*‐7**, b) ***ZE*‐7**, c) ***EZ*‐7**, and d) ***ZZ*‐7**.

All four stable configurations of AOD **1–7** were studied by DFT calculations to relate and predict their energies, geometrical orientation, and absorption spectra (TD‐PBE0/def2‐TZVP). All structures were thereby optimized with the r2scan‐3c method for both the gas phase and in DMSO using the SMD solvation model. As mentioned afore, the results showcase that the isolated ***EE*** isomer is indeed the most stable configuration, displaying the lowest energy of all four isomers (see Computational Analysis and Simulated UV–vis Spectra section in the Supporting Information). Isomers where the azobenzene occupies the *Z* configuration display higher energies in comparison. According to these calculations, the distance between the aromatic rings of the azobenzene and oxindole moieties increases more than twice, and their geometry (arene‐arene angle) changes from almost perpendicular to planar. The simulated UV–vis spectra in the ***EE*** isomeric form display no separation which is in accordance with the experimental obtained spectra (see Figure 6 and Supporting Information). All other experimentally obtained spectra refer to isomeric mixtures except for ***EZ*‐1** and ***EZ*‐7**.

## Conclusions

In summary, we present seven dyad‐photoactive molecules, containing electron‐donating and ‐withdrawing groups, built by combining azobenzene‐ and oxindole‐based molecular switches. The newly obtained azobenzene‐oxindole dyad exhibits similar absorption bands and has the two chromophores in close proximity; nevertheless, they can be selectively addressed by using different wavelengths and thus display sequential switching behavior. These drastic geometrical changes originating from the four possible isomers open up a myriad of possible future applications. Additionally, the analysis of the time evolution of the photoisomeric distribution by in situ ^1^H NMR allowed us to study the photokinetic behavior of the AODs. By doing so, we discovered that the switching behavior of the two photoresponsive moieties can be coupled when visible light is used. This results in an unprecedented azobenzene *E *→ *Z *→ *E* isomerization using a single stimulus due to the influence of the oxindole moiety. As a result, regardless of the PSS, the ***ZE*** photoisomer is generated first, acting as a gatekeeper for all the following transformations. We expect that this new phenomenon can be further explored and applied to generate new types of photochemical induced molecular motions.

## Author Contributions

M.O. performed synthesis of the azobenzenes and NMR analysis of the final molecules. D.D. performed synthesis, UV–vis spectroscopy and DFT calculations of the final molecules. M.O. and D.D. wrote the manuscript M.O., D.D., and B.L.F. reviewed and edited the manuscript. B.L.F. supervised the work. All authors discussed and commented on the manuscript. B.L.F. acquired funding.

## Conflict of Interests

The authors declare no conflict of interest.

## Supporting information



Supporting Information S1

Supporting Video S1

Supporting Video S2

Supporting Video S3

Supporting Video S4

Supporting Video S5

Supporting Video S6

Supporting Video S7

Supporting Video S8

Supporting Video S9

Supporting Video S10

Supporting Video S11

Supporting Video S12

Supporting Video S13

Supporting Information S2

## Data Availability

The data that support the findings of this study are available in the supplementary material of this article.

## References

[1] E. Fischer , Ber. Dtsch. Chem. Ges. 1894, 27, 29853.

[2] S. Mecozzi , J. Rebek , Chem. Eur. J. 1998, 4, 1016–1022.

[3] D.‐H. Qu , Q.‐C. Wang , Q.‐W. Zhang , X. Ma , H. Tian , Chem. Rev. 2015, 115, 7543–7588. 25697681 10.1021/cr5006342

[4] F. J. Rizzuto , L. K. S. von Krbek , J. R. Nitschke , Nat. Rev. Chem. 2019, 3, 204–222.

[5] H. Jiang , D. Alezi , M. Eddaoudi , Nat. Rev. Mater. 2021, 6, 466–487.

[6] S. Pullen , J. Tessarolo , G. H. Clever , Chem. Sci. 2021, 12, 7269–7293. 34163819 10.1039/d1sc01226f PMC8171321

[7] B. L. Feringa , W. R. Browne , Molecular Switches, Wiley‐VCH Verlag GmbH & Co. KGaA, Weinhein 2011.

[8] J. Volarić , W. Szymanski , N. A. Simeth , B. L. Feringa , Chem. Soc. Rev. 2021, 50, 12377–12449. 34590636 10.1039/d0cs00547a PMC8591629

[9] M.‐M. Russew , S. Hecht , Adv. Mater. 2010, 22, 3348–3360. 20422653 10.1002/adma.200904102

[10] Z. L. Pianowski , Molecular Photoswitches: Chemistry, Properties, and Applications, Wiley‐VCH Verlag GmbH & Co. KGaA, Weinheim 2022.

[11] Z. Zhang , W. Wang , M. O'Hagan , J. Dai , J. Zhang , H. Tian , Angew. Chem. Int. Ed. 2022, 61, e202205758. 10.1002/anie.202205758 35524420

[12] P. Kobauri , F. J. Dekker , W. Szymanski , B. L. Feringa , Angew. Chem. Int. Ed. 2023, 62, e202300681. 10.1002/anie.202300681 37026576

[13] J. Broichhagen , J. A. Frank , D. Trauner , Acc. Chem. Res. 2015, 48, 1947–1960. 26103428 10.1021/acs.accounts.5b00129

[14] R. Dorel , B. L. Feringa , Chem. Commun. 2019, 55, 6477–6486. 10.1039/c9cc01891c 31099809

[15] D. Villarón , M. A. Siegler , S. J. Wezenberg , Chem. Sci. 2021, 12, 3188–3193. 34164086 10.1039/d0sc06686a PMC8179391

[16] F. Xu , B. L. Feringa , Adv. Mater. 2023, 35, 2204413. 10.1002/adma.202204413 36239270

[17] M. Ovalle , C. N. Stindt , B. L. Feringa , J. Am. Chem. Soc. 2024, 146, 31892–31900. 39500717 10.1021/jacs.4c11206 PMC11583216

[18] V. Serreli , C.‐F. Lee , E. R. Kay , D. A. Leigh , Nature 2007, 445, 523–527. 17268466 10.1038/nature05452

[19] R. Costil , M. Holzheimer , S. Crespi , N. A. Simeth , B. L. Feringa , Chem. Rev. 2021, 121, 13213–13237. 34533944 10.1021/acs.chemrev.1c00340 PMC8587610

[20] B. Shao , H. Fu , I. Aprahamian , Science 2024, 385, 544–549. 39088617 10.1126/science.adp3506

[21] M. Kathan , S. Crespi , N. O. Thiel , D. L. Stares , D. Morsa , J. de Boer , G. Pacella , T. van den Enk , P. Kobauri , G. Portale , C. A. Schalley , B. L. Feringa , Nat. Nanotechnol. 2022, 17, 159–165. 34916655 10.1038/s41565-021-01021-z PMC8956507

[22] S. Corra , M. T. Bakić , J. Groppi , M. Baroncini , S. Silvi , E. Penocchio , M. Esposito , A. Credi , Nat. Nanotechnol. 2022, 17, 746–751. 35760895 10.1038/s41565-022-01151-y

[23] S. Crespi , N. A. Simeth , B. König , Nat. Rev. Chem. 2019, 3, 133–146.

[24] F. A. Jerca , V. V. Jerca , R. Hoogenboom , Nat. Rev. Chem. 2022, 6, 51–69. 37117615 10.1038/s41570-021-00334-w

[25] L. Kortekaas , W. R. Browne , Chem. Soc. Rev. 2019, 48, 3406–3424. 31150035 10.1039/c9cs00203k

[26] F. Xu , J. Sheng , C. N. Stindt , S. Crespi , W. Danowski , M. F. Hilbers , W. J. Buma , B. L. Feringa , Chem. Sci. 2024, 15, 6763–6769. 38725493 10.1039/d4sc00983e PMC11077541

[27] D. Villarón , S. J. Wezenberg , Angew. Chem. Int. Ed. 2020, 59, 13192–13202. 10.1002/anie.202001031 PMC7496324 32222016

[28] S. Crespi , N. A. Simeth , M. Di Donato , S. Doria , C. N. Stindt , M. F. Hilbers , F. L. Kiss , R. Toyoda , S. Wesseling , W. J. Buma , B. L. Feringa , W. Szymański , Angew. Chem. Int. Ed. 2021, 60, 25290–25295. 10.1002/anie.202111748 PMC9298291 34609785

[29] T. T. Ngoc , N. Grabicki , E. Irran , O. Dumele , J. F. Teichert , Nat. Chem. 2023, 15, 377–385. 36702883 10.1038/s41557-022-01121-w PMC9986110

[30] I. Aprahamian , Chem. Commun. 2017, 53, 6674–6684. 10.1039/c7cc02879b 28540954

[31] V. Josef , F. Hampel , H. Dube , Angew. Chem. Int. Ed. 2022, 61, e202210855. 10.1002/anie.202210855 PMC9826360 36040861

[32] W. Wang , W. Yang , Z. Zhang , J. Dai , Y. Xu , J. Zhang , Chem. Sci. 2024, 15, 5539–5547. 38638239 10.1039/d4sc00423j PMC11023046

[33] Z. Zhang , W. Wang , P. Jin , J. Xue , L. Sun , J. Huang , J. Zhang , H. Tian , Nat. Commun. 2019, 10, 4232. 31530814 10.1038/s41467-019-12302-6 PMC6748945

[34] L. Köttner , H. Dube , Angew. Chem. Int. Ed. 2024, 63, e202409214. 10.1002/anie.202409214 38958439

[35] J. Andréasson , S. D. Straight , T. A. Moore , A. L. Moore , D. Gust , J. Am. Chem. Soc. 2008, 130, 11122–11128. 18661987 10.1021/ja802845z

[36] F. Zhao , L. Grubert , S. Hecht , D. Bléger , Chem. Commun. 2017, 53, 3323–3326. 10.1039/c7cc00504k 28210737

[37] K. Mutoh , Y. Kobayashi , T. Nakashima , Angew. Chem. Int. Ed. 2024, 63, e202410115. 10.1002/anie.202410115 38894673

[38] A. Mengots , A. Erbs Hillers‐Bendtsen , S. Doria , F. Ørsted Kjeldal , N. Machholdt Høyer , A. Ugleholdt Petersen , K. V. Mikkelsen , M. Di Donato , M. Cacciarini , M. Brøndsted Nielsen , Chem. Eur. J. 2021, 27, 12437–12446. 34096662 10.1002/chem.202101533

[39] M. Dowds , S. G. Stenspil , J. H. de Souza , B. W. Laursen , M. Cacciarini , M. B. Nielsen , ChemPhotoChem 2022, 6, e202200152.

[40] W. Zhao , E. M. Carreira , J. Am. Chem. Soc. 2002, 124, 1582–1583. 11853425 10.1021/ja017291b

[41] Y. Kobayashi , K. Mutoh , J. Abe , J. Phys. Chem. Lett. 2016, 7, 3666–3675. 27585058 10.1021/acs.jpclett.6b01690

[42] M. M. Lerch , M. J. Hansen , W. A. Velema , W. Szymanski , B. L. Feringa , Nat. Commun. 2016, 7, 12054. 27401266 10.1038/ncomms12054 PMC4945879

[43] J. Sheng , J. Perego , S. Bracco , P. Cieciórski , W. Danowski , A. Comotti , B. L. Feringa , Angew. Chem. Int. Ed. 2024, 63, e202404878. 10.1002/anie.202404878 38530132

[44] A. Samat , V. Lokshin , K. Chamontin , D. Levi , G. Pepe , R. Guglielmetti , Tetrahedron 2001, 57, 7349–7359.

[45] K. Kinashi , K. Furuta , Y. Harada , Y. Ueda , Chem. Lett. 2006, 35, 298–299.

[46] T. Saßmannshausen , A. Kunz , N. Oberhof , F. Schneider , C. Slavov , A. Dreuw , J. Wachtveitl , H. A. Wegner , Angew. Chem. Int. Ed. 2024, 63, e202314112. 10.1002/anie.202314112 38059778

[47] C. Slavov , C. Yang , L. Schweighauser , C. Boumrifak , A. Dreuw , H. A. Wegner , J. Wachtveitl , Phys. Chem. Chem. Phys. 2016, 18, 14795–14804. 26996604 10.1039/c6cp00603e

[48] D. Doellerer , D. R. S. Pooler , A. Guinart , S. Crespi , B. L. Feringa , Chem. Eur. J. 2023, 29, e202301634. 37345715 10.1002/chem.202301634

[49] D. Roke , M. Sen , W. Danowski , S. J. Wezenberg , B. L. Feringa , J. Am. Chem. Soc. 2019, 141, 7622–7627. 31017421 10.1021/jacs.9b03237 PMC6509644

[50] D. R. S. Pooler , D. Doellerer , S. Crespi , B. L. Feringa , Org. Chem. Front. 2022, 9, 2084–2092. 35516070 10.1039/d2qo00129b PMC9003629

[51] A. Guinart , D. Doellerer , D. R. S. Pooler , J. Y. de Boer , S. Doria , L. Bussotti , M. Di Donato , B. L. Feringa , J. Photochem. Photobiol. Chem. 2024, 453, 115649.

[52] M. Barfield , D. M. Grant , D. Ikenberry , J. Am. Chem. Soc. 1975, 97, 6956–6961.

[53] K. Rustler , P. Nitschke , S. Zahnbrecher , J. Zach , S. Crespi , B. König , J. Org. Chem. 2020, 85, 4079–4088. 32070094 10.1021/acs.joc.9b03097

[54] P. Gerstel , S. Klumpp , F. Hennrich , A. Poschlad , V. Meded , E. Blasco , W. Wenzel , M. M. Kappes , C. Barner‐Kowollik , ACS Macro Lett. 2014, 3, 10–15. 35632861 10.1021/mz400472q

[55] M. Ovalle , M. Kathan , R. Toyoda , C. N. Stindt , S. Crespi , B. L. Feringa , Angew. Chem. Int. Ed. 2023, 62, e202214495. 10.1002/anie.202214495 36453623

