# Supplementary material for: Azobenzene‐Oxindole Photochromic Dyads
Source: Angew Chem Int Ed Engl. 2025 Apr 2;64(22):e202501872. doi: 10.1002/anie.202501872 (PMC12105706; doi:10.1002/anie.202501872)
Supplement: Supplementary file 1 — Supporting Information S1 [file ANIE-64-e202501872-s005.pdf]

Supporting Information

Azobenzene-Oxindole Photochromic Dyads

Marco Ovalle‡, Daniel Doellerer‡ & Ben L. Feringa*

Stratingh Institute for Chemistry, Center for Systems Chemistry and Zernike Institute for Advanced Materials, Faculty of Mathematics and Natural Sciences, University of Groningen, Nijenborgh 3, 9747 AG Groningen, The Netherlands

‡ contributed equally
* corresponding author: b.l.feringa@rug.nl

Table of Contents

GENERAL INFORMATION **3**
SYNTHESIS **4**
NMR EXPERIMENTS **13**
UV-VIS SPECTROSCOPY **28**
QY DETERMINATION **53**
COMPUTATIONAL ANALYSIS AND SIMULATED UV-VIS SPECTRA **55**
REFERENCES **60**
APPENDIX **61**
NMR SPECTRA 61

General Information

Chemicals were purchased from commercial sources, Sigma-Aldrich, Fluorochem, TCI, BLDpharm and used without further purification. Dried solvents were obtained from Acros Organics, Alfa Aesar or from a solvent purification system (MBraun SPS-800). Unless stated otherwise, all reactions were carried out in oven-dried glassware under a nitrogen atmosphere using standard Schlenk techniques. Solids were added in a counter flow of nitrogen or before crimping the vials and cycled three times between vacuum and nitrogen before addition of liquids. Solutions and reagents were added with nitrogen-flushed disposable syringes/needles.

Analytical thin layer chromatography (TLC) was performed on silica gel 60 G/UV265 aluminum sheets from Merck (0.25 mm). Flash column chromatography was performed on silica gel Davisil LC60A (Merck type 9385, 230–400 mesh) or a Biotage Selekt system (MPLC) using the indicated solvents. NMR spectra were recorded on a Varian Mercury-Plus 400, a Varian Unity Plus 500 or a Bruker 600 MHz NMR spectrometer at 298 K unless stated otherwise. Chemical shifts are reported in parts per million (ppm) and referenced to the residual solvent signal ($CDCl_3$: δ = 7.26 for ^{1}H and 77.2 for ^{13}C{^{1}H}; $(CD_3)_2SO$: δ = 2.50 for ^{1}H and 39.5 for ^{13}C{^{1}H}; C_6D_6: δ = 7.16 for ^{1}H; CD_3OD: δ = 3.31 for ^{1}H) and thereby stated relatively to TMS. The resonance multiplicity is indicated as s = singlet, d = doublet, t = triplet, m = multiplet, dd (doublet of doublets), ddd (doublet of doublets of doublets), dt (doublet of triplets), td (triplet of doublets), tdd (triplet of doublets of doublets), br = broad and the coupling constant values (*J*) are given in hertz (Hz). High resolution mass spectra (HRMS) were recorded on a LTQ Orbitrap XL spectrometer. UV-Vis absorption spectra were recorded on an Agilent 8453 UV-Vis Diode Array System, equipped with a Quantum Northwest Peltier controller in 10 mm quartz cuvettes. Irradiation experiments were performed using LEDs from Thorlabs Incorporated (340, 365, 415 and 470 nm; 0.7 mA for 340, 1.2 mA for 365 and 415 nm and 1.0 mA for 470 nm).

^{1}H NMR and *in-situ* irradiation experiments were performed using a Varian Inova 500 (500 MHz) spectrometer a fiber-coupled LED and a 1000 μm optical fiber (FP1000URT) modified to fit into the NMR tube, as described in the literature[1] or *ex-situ* with the same LEDs positioned at a distance of 3 cm from the samples. Irradiation experiments were performed using Thorlabs LEDs (M340F3, M365FP1, M415L4, M470L5).

The experimental data was fitted by the default Levenberg-Marquardt COPASI algorithm[2]Click or tap here to enter text. with a tolerance of $1x10^{-6}$ h^{-1}. The initial guess for the kinetic parameter estimation was a random value.

To obtain ***EZ*-1** and ***EZ*-7**, a photoreactor 'm2' from Acceled was used at 420 nm for 30 min at 100% light intensity (30 s post cool down at 250 rpm with a fan speed of 6500 rpm).

All NMR spectra of the synthesized compounds can be found in the appendix of the Supporting Information.

Synthesis
General Procedure A[3]

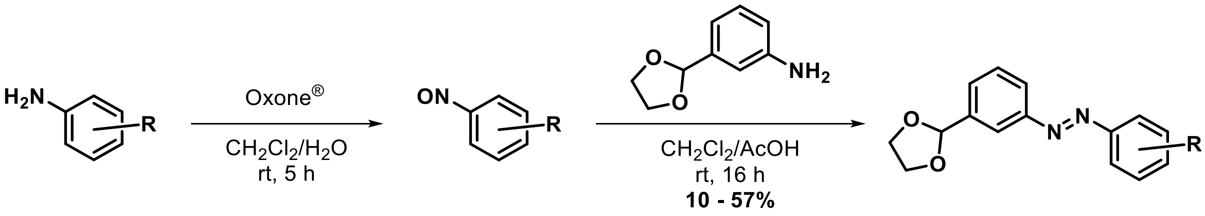

8 - 14

R = H, *o-/m-/p*-F, *o-/m-/p*-OMe

Azobenzenes **8 – 14** were synthesized over 2 steps, starting by the oxidation of the respective aniline (1.0 eq.) dissolved in CH_2Cl_2, preserving a concentration of 0.1M, a solution of oxone® in H_2O (1.0 eq., 10 mL/mmol amine) was added and the biphasic reaction mixture stirred at room temperature for 5 h. The organic phase was separated, washed with H_2O and brine and dried over anhydrous Na_2SO_4. The crude compound was purified by filtering over silica and the solvent removed under reduced pressure. A 0.05 M solution of 3-(1,3-dioxolan-2-yl)aniline (1.0 eq.) in a 1:1 mixture of CH_2Cl_2 and AcOH was added and the mixture stirred at room temperature for 16 h. The solvent was removed under reduced pressure, the residue redissolved in CH_2Cl_2, washed with a saturated aqueous solution of $NaHCO_3$ and brine, dried over anhydrous Na_2SO_4 and the solvent removed under reduced pressure The crude was purified *via* flash column chromatography using *n*-pentane and EtOAc (9:1) as an eluent.

(*E*)-1-(3-(1,3-dioxolan-2-yl)phenyl)-2-phenyldiazene (8)

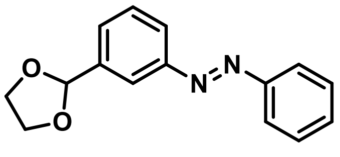

8

$C_{15}H_{14}N_2O_2$
Mw = 254.29 g/mol

Synthesized following general procedure **A**, using aniline (282 mg, 3.03 mmol, 1.0 eq.), oxone® and 3-(1,3-dioxolan-2-yl)aniline (500 mg, 3.03 mmol, 1.0 eq.). Purification *via* flash column chromatography yielded **8** as an orange oil (408 mg, 1.61 mmol, 53%).

^{1}H NMR (400 MHz, $CDCl_3$): δ = 8.10 (s, 1H), 8.00 – 7.92 (m, 3H), 7.63 (d, *J* = 7.7 Hz, 1H), 7.58 – 7.46 (m, 4H), 5.95 (s, 1H), 4.20 – 4.04 (m, 4H).
^{13}C{^{1}H} NMR (101 MHz, $CDCl_3$): δ = 152.7, 152.7, 139.4, 131.2, 129.2, 129.2, 129.0, 123.9, 123.0, 120.8, 103.3, 65.4.
HRMS-ESI (ESI+): calculated for $C_{15}H_{14}N_2O_2H^+$ $[M+H]^+$ 255.1128, found 255.1128.

(*E*)-1-(3-(1,3-dioxolan-2-yl)phenyl)-2-(2-fluorophenyl)diazene (9)

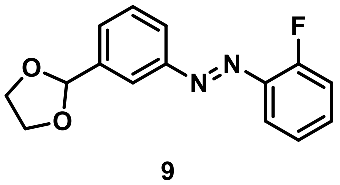

9

$C_{15}H_{13}FN_2O_2$
Mw = 272.28 g/mol

Synthesized following general procedure **A**, using 2-fluoroaniline (337 mg, 3.03 mmol, 1.0 eq.), oxone® and 3-(1,3-dioxolan-2-yl)aniline (500 mg, 3.03 mmol, 1.0 eq.). Purification *via* flash column chromatography yielded **9** as an orange oil (354 mg, 1.30 mmol, 43%).

^{1}H NMR (400 MHz, $CDCl_3$): δ = 8.08 (t, *J* = 1.9 Hz, 1H), 7.96 (ddd, *J* = 7.9, 2.0, 1.3 Hz, 1H), 7.76 (td, *J* = 7.8, 1.8 Hz, 1H), 7.63 (dt, *J* = 7.6, 1.5 Hz, 1H), 7.54 (t, *J* = 7.7 Hz, 1H), 7.51 – 7.41 (m, 1H), 7.30 – 7.26 (m, 1H), 7.25 – 7.19 (m, 1H), 5.93 (s, 1H), 4.22 – 4.04 (m, 4H).
^{13}C{^{1}H} NMR (101 MHz, $CDCl_3$): δ = 160.3 (d), 152.9, 140.8 (d), 139.5, 132.7 (d), 129.5, 129.3, 124.4 (d), 123.9, 121.6, 117.9, 117.2 (d), 103.4, 65.5.
^{19}F NMR (376 MHz, $CDCl_3$): δ = -124.2.
HRMS-ESI (ESI+): calculated for $C_{15}H_{13}FN_2O_2H^+$ $[M+H]^+$ 273.1034, found 273.1036.

(*E*)-1-(3-(1,3-dioxolan-2-yl)phenyl)-2-(3-fluorophenyl)diazene (10)

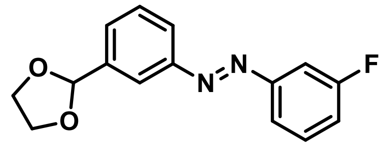

10

$C_{15}H_{13}FN_2O_2$
Mw = 272.28 g.mol

Synthesized following general procedure **A**, using 3-fluoroaniline (337 mg, 3.03 mmol, 1.0 eq.), oxone® and 3-(1,3-dioxolan-2-yl)aniline (500 mg, 3.03 mmol, 1.0 eq.). Purification *via* flash column chromatography yielded **10** as an orange oil (425 mg, 1.56 mmol, 52%).

^{1}H NMR (400 MHz, $CDCl_3$): δ = 8.10 (s, 1H), 7.95 (d, *J* = 7.9 Hz, 1H), 7.78 (d, *J* = 7.9 Hz, 1H), 7.67 – 7.61 (m, 2H), 7.54 (t, *J* = 7.7 Hz, 1H), 7.47 (td, *J* = 8.1, 5.9 Hz, 1H), 7.18 (td, *J* = 8.1, 2.6 Hz, 1H), 5.92 (s, 1H), 4.19 – 3.99 (m, 4H).
^{13}C{^{1}H} NMR (101 MHz, $CDCl_3$): δ = 163.2 (d), 154.0 (d), 152.3, 139.5, 130.3 (d), 129.4, 129.2, 124.0, 120.9, 120.6 (d), 117.8 (d), 108.0 (d), 103.1, 65.3.
^{19}F NMR (376 MHz, $CDCl_3$): δ = -111.8.
HRMS-ESI (ESI+): calculated for $C_{15}H_{13}FN_2O_2H^+$ $[M+H]^+$ 273.1034, found 273.1033.

(*E*)-1-(3-(1,3-dioxolan-2-yl)phenyl)-2-(4-fluorophenyl)diazene (11)

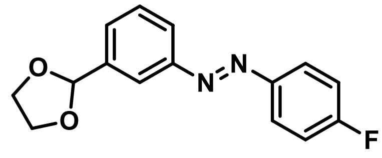

11

$C_{15}H_{13}FN_2O_2$
Mw = 272.28 g/mol

Synthesized following general procedure **A**, using 4-fluoroaniline (337 mg, 3.03 mmol, 1.0 eq.), oxone® and 3-(1,3-dioxolan-2-yl)aniline (500 mg, 3.03 mmol, 1.0 eq.). Purification *via* flash column chromatography yielded **11** as an orange oil (354 mg, 1.30 mmol, 43%).

^{1}H NMR (400 MHz, $CDCl_3$): δ = 8.04 (t, *J* = 1.8 Hz, 1H), 7.98 – 7.89 (m, 3H), 7.61 (dt, *J* = 7.6, 1.5 Hz, 1H), 7.54 (t, *J* = 7.7 Hz, 1H), 7.20 (t, *J* = 8.6 Hz, 2H), 5.93 (s, 1H), 4.21 – 4.03 (m, 4H).
^{13}C{^{1}H} NMR (101 MHz, $CDCl_3$): δ = 164.5 (d), 152.6, 149.2 (d), 139.5, 129.3, 129.1, 125.0 (d), 123.9, 120.8, 116.2 (d), 103.4, 65.5.
^{19}F NMR (376 MHz, $CDCl_3$): δ = -109.2.
HRMS-ESI (ESI+): calculated for $C_{15}H_{13}FN_2O_2H^+$ $[M+H]^+$ 273.1034, found 273.1035.

(*E*)-1-(3-(1,3-dioxolan-2-yl)phenyl)-2-(2-methoxyphenyl)diazene (12)

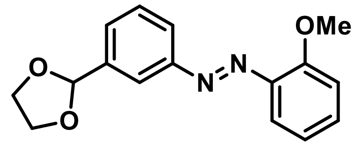

12

$C_{16}H_{16}N_2O_3$
Mw = 284.32 g/mol

Synthesized following general procedure **A**, using *o*-anisidine (373 mg, 3.03 mmol, 1.0 eq.), oxone® and 3-(1,3-dioxolan-2-yl)aniline (500 mg, 3.03 mmol, 1.0 eq.). Purification *via* flash column chromatography yielded **12** as an orange oil (292 mg, 1.03 mmol, 34%).

^{1}H NMR (400 MHz, $CDCl_3$): δ = 8.03 (s, 1H), 7.91 (d, *J* = 7.8 Hz, 1H), 7.65 (dd, *J* = 8.0, 1.7 Hz, 1H), 7.59 (d, *J* = 7.7 Hz, 1H), 7.52 (t, *J* = 7.7 Hz, 1H), 7.48 – 7.42 (m, 1H), 7.10 (d, *J* = 8.4 Hz, 1H), 7.02 (t, *J* = 7.6 Hz, 1H), 5.93 (s, 1H), 4.19 – 4.05 (m, 4H), 4.03 (s, 3H).
^{13}C{^{1}H} NMR (101 MHz, $CDCl_3$): δ = 157.2, 153.3, 142.5, 139.4, 132.7, 129.3, 128.8, 123.6, 121.6, 120.9, 117.2, 112.9, 103.5, 65.5, 56.5.
HRMS-ESI (ESI+): calculated for $C_{16}H_{12}N_2O_2H^+$ $[M+H]^+$ 285.1234, found 285.1232.

(*E*)-1-(3-(1,3-dioxolan-2-yl)phenyl)-2-(3-methoxyphenyl)diazene (13)

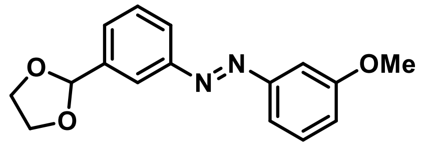

13

$C_{16}H_{16}N_2O_3$
Mw = 284.32 g/mol

Synthesized following general procedure **A**, using *m*-anisidine (373 mg, 3.03 mmol, 1.0 eq.), oxone® and 3-(1,3-dioxolan-2-yl)aniline (500 mg, 3.03 mmol, 1.0 eq.). Purification *via* flash column chromatography yielded **13** as an orange oil (491 mg, 1.73 mmol, 57%).

^{1}H NMR (400 MHz, $CDCl_3$): δ = 8.07 (t, *J* = 1.8 Hz, 1H), 7.94 (ddd, *J* = 7.8, 2.0, 1.3 Hz, 1H), 7.63 – 7.52 (m, 3H), 7.47 (t, *J* = 2.2 Hz, 1H), 7.43 (t, *J* = 8.0 Hz, 1H), 7.05 (ddd, *J* = 8.2, 2.6, 1.0 Hz, 1H), 5.93 (s, 1H), 4.19 – 4.05 (m, 4H), 3.90 (s, 3H).
^{13}C{^{1}H} NMR (101 MHz, $CDCl_3$): δ = 160.4, 153.9, 152.7, 139.4, 129.9, 129.3, 129.1, 123.9, 120.9, 118.1, 117.3, 105.8, 103.4, 65.5, 55.6.
HRMS-ESI (ESI+): calculated for $C_{16}H_{16}N_2O_2H^+$ $[M+H]^+$ 285.1234, found 285.1232.

(*E*)-1-(3-(1,3-dioxolan-2-yl)phenyl)-2-(4-methoxyphenyl)diazene (14)

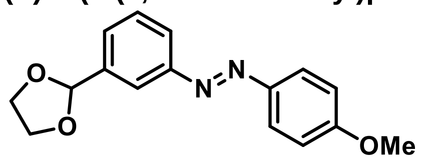

14

$C_{16}H_{16}N_2O_3$
Mw = 284.32 g/mol

Synthesized following general procedure **A**, using *o*-anisidine (746 mg, 6.06 mmol, 1.0 eq.), oxone® and 3-(1,3-dioxolan-2-yl)aniline (1.00 g, 6.06 mmol, 1.0 eq.). Purification *via* flash column chromatography yielded **14** as an orange oil (163 mg, 0.61 mmol, 10%).

^{1}H NMR (400 MHz, $CDCl_3$): δ = 8.01 (s, 1H), 7.95 – 7.91 (m, 2H), 7.89 (dt, *J* = 7.6, 1.7 Hz, 1H), 7.57 (d, *J* = 7.6 Hz, 1H), 7.52 (t, *J* = 7.6 Hz, 1H), 7.04 – 6.99 (m, 2H), 5.93 (s, 1H), 4.20 – 4.05 (m, 4H), 3.89 (s, 3H).
^{13}C{^{1}H} NMR (101 MHz, $CDCl_3$): δ = 162.3, 152.9, 147.1, 139.3, 129.3, 128.4, 125.0, 123.7, 120.6, 114.4, 103.5, 65.5, 55.7.
HRMS-ESI (ESI+): calculated for $C_{15}H_{13}FN_2O_2H^+$ $[M+H]^+$ 285.1234, found 285.1232.

General Procedure **B**[4]

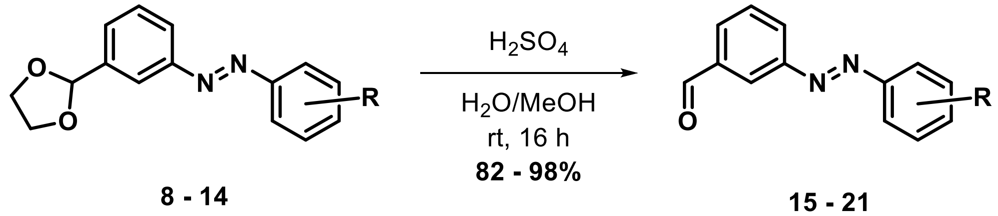

8 - 14 **15 - 21**

R = H, *o*-/*m*-/*p*-F, *o*-/*m*-/*p*-OMe

The specific protected azobenzene **8** – **14** (1.0 eq.) was dissolved in MeOH (20 mL/mmol of azobenzene), aqueous H_2SO_4 (10%, 30 mL/mmol of azobenzene) added and the mixture stirred at room temperature for 16 h. The resulting orange precipitate was filtered off, washed with water and redissolved in CH_2Cl_2. The solution was washed with a saturated solution of aqueous $NaHCO_3$, H_2O and brine, dried over anhydrous Na_2SO_4 and the solvent removed under reduced pressure. The crude compound was purified *via* flash column chromatography using *n*-pentane and EtOAc (7:3) as an eluent.

(*E*)-3-(phenyldiazenyl)benzaldehyde (15)

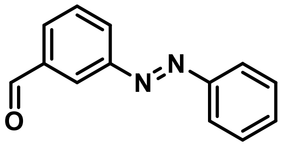

15

$C_{13}H_{10}N_2O$

Mw = 210.24 g/mol

Synthesized following general procedure **B**, using **8** (100 mg, 0.39 mmol, 1.0 eq.). Purification *via* flash column chromatography yielded **15** as an orange solid (73.0 mg, 0.35 mmol, 89%).

^{1}H NMR (400 MHz, $CDCl_3$): δ = 10.13 (s, 1H), 8.40 (t, *J* = 1.8 Hz, 1H), 8.18 (ddd, *J* = 7.9, 2.0, 1.2 Hz, 1H), 8.00 (dt, *J* = 7.6, 1.4 Hz, 1H), 7.99 – 7.91 (m, 2H), 7.68 (t, *J* = 7.7 Hz, 1H), 7.57 – 7.50 (m, 3H).
^{13}C{^{1}H} NMR (101 MHz, $CDCl_3$): δ = 191.8, 153.1, 152.4, 137.4, 131.8, 131.2, 130.0, 129.3, 128.9, 123.9, 123.2.
HRMS-ESI (ESI+): calculated for $C_{13}H_{10}N_2OH^+$ $[M+H]^+$ 211.0866, found 211.0864.

(*E*)-3-((2-fluorophenyl)diazenyl)benzaldehyde (16)

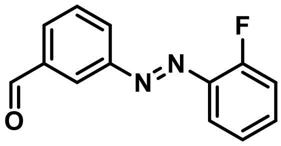

16

$C_{13}H_9FN_2O$

Mw = 228.23 g/mol

Synthesized following general procedure **B**, using **9** (100 mg, 0.37 mmol, 1.0 eq.). Purification *via* flash column chromatography yielded **16** as an orange solid (78.0 mg, 0.34 mmol, 93%).

^{1}H NMR (400 MHz, $CDCl_3$): δ = 10.13 (s, 1H), 8.41 (t, *J* = 1.8 Hz, 1H), 8.19 (ddd, *J* = 7.9, 2.0, 1.2 Hz, 1H), 8.02 (dt, *J* = 7.6, 1.4 Hz, 1H), 7.78 (td, *J* = 7.8, 1.8 Hz, 1H), 7.69 (t, *J* = 7.7 Hz, 1H), 7.53 – 7.45 (m, 1H), 7.33 – 7.19 (m, 2H).
^{13}C{^{1}H} NMR (101 MHz, $CDCl_3$): δ = 191.7, 160.5 (d), 153.2, 140.5 (d), 137.5, 133.4 (d), 131.5, 130.0, 128.7, 124.6, 124.5 (d), 117.8, 117.4 (d).
^{19}F NMR (376 MHz, $CDCl_3$): δ = -123.7.
HRMS-ESI (ESI+): calculated for $C_{15}H_9FN_2OH^+$ $[M+H]^+$ 229.0772, found 229.0771.

(*E*)-3-((3-fluorophenyl)diazenyl)benzaldehyde (17)

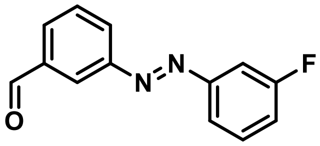

17

$C_{13}H_9FN_2O$

Mw = 228.23 g/mol

Synthesized following general procedure **B**, using **10** (100 mg, 0.37 mmol, 1.0 eq.). Purification *via* flash column chromatography yielded **17** as an orange solid (83.0 mg, 0.36 mmol, 98%).

^{1}H NMR (400 MHz, $CDCl_3$): δ = 10.14 (s, 1H), 8.40 (t, *J* = 1.8 Hz, 1H), 8.18 (ddd, *J* = 7.9, 2.0, 1.2 Hz, 1H), 8.02 (dt, *J* = 7.6, 1.4 Hz, 1H), 7.80 (ddd, *J* = 7.9, 1.8, 1.0 Hz, 1H), 7.70 (t, *J* = 7.7 Hz, 1H), 7.63 (dt, *J* = 9.6, 2.2 Hz, 1H), 7.52 (td, *J* = 8.1, 5.9 Hz, 1H), 7.22 (tdd, *J* = 8.2, 2.6, 1.0 Hz, 1H).
^{13}C{^{1}H} NMR (101 MHz, $CDCl_3$): δ = 191.7, 163.4 (d), 153.9 (d), 152.8, 137.5, 131.7, 130.5 (d), 130.1, 129.0, 124.0, 121.0 (d), 118.5 (d), 108.3 (d).
^{19}F NMR (376 MHz, $CDCl_3$): δ = -111.7.
HRMS-ESI (ESI+): calculated for $C_{15}H_9FN_2OH^+$ $[M+H]^+$ 229.0772, found 229.0772.

(*E*)-3-((4-fluorophenyl)diazenyl)benzaldehyde (18)

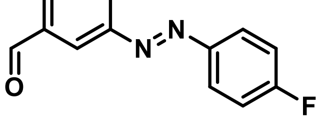

18

$C_{13}H_9FN_2O$

Mw = 228.23 g/mol

Synthesized following general procedure **B**, using **11** (100 mg, 0.37 mmol, 1.0 eq.). Purification *via* flash column chromatography yielded **18** as an orange solid (73.0 mg, 0.32 mmol, 88%).

^{1}H NMR (400 MHz, $CDCl_3$): δ = 10.10 (s, 1H), 8.34 (s, 1H), 8.13 (d, *J* = 7.9 Hz, 1H), 8.01 – 7.90 (m, 3H), 7.65 (t, *J* = 7.7 Hz, 1H), 7.19 (t, *J* = 8.6 Hz, 2H).
^{13}C{^{1}H} NMR (101 MHz, $CDCl_3$): δ = 191.7, 164.8 (d), 152.8, 148.9 (d), 137.4, 131.3, 129.9, 128.8, 125.3 (d), 123.7, 116.3 (d).
^{19}F NMR (376 MHz, $CDCl_3$): δ = -108.0.
HRMS-ESI (ESI+): calculated for $C_{13}H_9FN_2OH^+$ $[M+H]^+$ 229.0772, found 229.0770.

(*E*)-3-((2-methoxyphenyl)diazenyl)benzaldehyde (19)

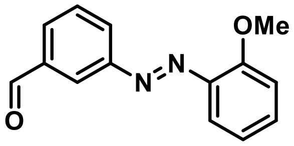

19
$C_{14}H_{12}N_2O_2$
Mw = 240.26 g/mol

Synthesized following general procedure **B**, using **12** (100 mg, 0.35 mmol, 1.0 eq.). Purification *via* flash column chromatography yielded **19** as an orange solid (80.0 mg, 0.33 mmol, 95%).

^{1}H NMR (400 MHz, $CDCl_3$): δ = 10.13 (s, 1H), 8.38 (s, 1H), 8.17 (d, *J* = 7.8 Hz, 1H), 7.99 (d, *J* = 7.6 Hz, 1H), 7.74 – 7.64 (m, 2H), 7.53 – 7.45 (m, 1H), 7.12 (d, *J* = 8.4 Hz, 1H), 7.04 (t, *J* = 7.7 Hz, 1H), 4.05 (s, 3H).
^{13}C{^{1}H} NMR (101 MHz, $CDCl_3$): δ = 192.0, 157.5, 153.7, 142.1, 137.5, 133.5, 130.8, 130.0, 129.1, 124.3, 120.9, 117.1, 113.0, 56.4.
HRMS-ESI (ESI+): calculated for $C_{14}H_{12}N_2O_2H^+$ $[M+H]^+$ 241.0972, found 241.0970.

(*E*)-3-((3-methoxyphenyl)diazenyl)benzaldehyde (20)

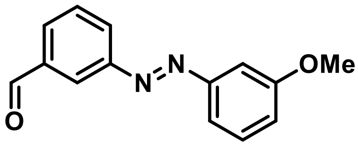

20
$C_{14}H_{12}N_2O_2$
Mw = 240.26 g/mol

Synthesized following general procedure **B**, using **13** (100 mg, 0.35 mmol, 1.0 eq.). Purification *via* flash column chromatography yielded **20** as an orange solid (82.0 mg, 0.34 mmol, 98%).

^{1}H NMR (400 MHz, $CDCl_3$): δ = 10.13 (s, 1H), 8.39 (t, *J* = 1.7 Hz, 1H), 8.17 (ddd, *J* = 7.9, 2.0, 1.1 Hz, 1H), 8.00 (dt, *J* = 7.6, 1.4 Hz, 1H), 7.68 (t, *J* = 7.7 Hz, 1H), 7.59 (ddd, *J* = 7.8, 1.8, 1.0 Hz, 1H), 7.50 – 7.41 (m, 2H), 7.08 (ddd, *J* = 8.2, 2.6, 1.0 Hz, 1H), 3.90 (s, 3H).
^{13}C{^{1}H} NMR (101 MHz, $CDCl_3$): δ = 191.8, 160.5, 153.7, 153.0, 137.5, 131.3, 130.0, 130.0, 128.9, 124.0, 118.6, 117.7, 105.9, 55.6.
HRMS-ESI (ESI+): calculated for $C_{14}H_{12}N_2O_2H^+$ $[M+H]^+$ 241.0972, found 241.0970.

(*E*)-3-((4-methoxyphenyl)diazenyl)benzaldehyde (21)

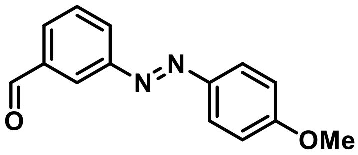

21
$C_{14}H_{12}N_2O_2$
Mw = 240.26 g/mol

Synthesized following general procedure **B**, using **14** (100 mg, 0.35 mmol, 1.0 eq.). Purification *via* flash column chromatography yielded **21** as an orange solid (75.0 mg, 0.31 mmol, 90%).

^{1}H NMR (400 MHz, $CDCl_3$): δ = 10.13 (s, 1H), 8.36 (t, *J* = 1.7 Hz, 1H), 8.14 (ddd, *J* = 7.9, 2.0, 1.2 Hz, 1H), 7.99 – 7.93 (m, 3H), 7.67 (t, *J* = 7.7 Hz, 1H), 7.06 – 7.01 (m, 2H), 3.91 (s, 3H).
^{13}C{^{1}H} NMR (101 MHz, $CDCl_3$): δ = 192.0, 162.8, 153.3, 146.9, 137.4, 130.6, 129.9, 128.7, 125.3, 123.7, 114.5, 55.8.
HRMS-ESI (ESI+): calculated for $C_{14}H_{12}N_2O_2H^+$ $[M+H]^+$ 241.0972, found 242.070.

General Procedure **C**[5]

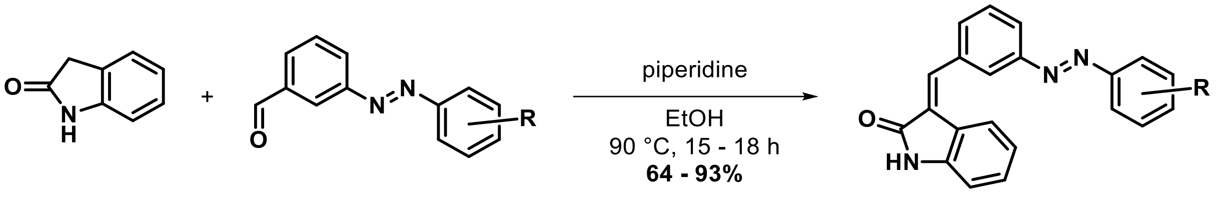

15 - 21 **_EE_-1 - _EE_-7**

R = H, *o-*/*m-*/*p-*F, *o-*/*m-*/*p-*OMe

An oven-dried crimp top vial equipped with a stirring bar was charged with oxindole (1.0 eq.) and aldehyde **15** – **21** (1.0 eq.). The vial was crimped, flushed with nitrogen, dry EtOH and piperidine (40.0 µL) were added and the reaction mixture heated under continuous stirring at 90 °C for 15 – 18 h. The reaction mixture was allowed to cool down to room temperature and put on ice. Precipitates were filtered off using a glass filter frit pore 4, washed with *n*-pentane and dried under reduced pressure. The solvent was removed under reduced pressure for compounds that did not precipitate and were purified *via* flash column chromatography using a gradient starting from *n*-pentane to EtOAc.

Azobenzene-Oxindole Dyad 1 (*EE*-1)

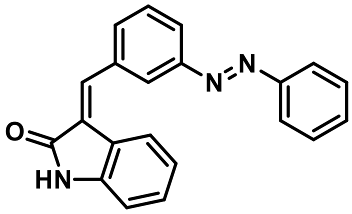

***EE*-1**
$C_{21}H_{15}N_3O$
Mw = 325.37 g/mol

Synthesized following general procedure **C**, using oxindole (65.0 mg, 0.49 mmol, 1.05 eq.), **15** (99.5 mg, 0.47 mmol, 1.00 eq.), piperidine (40.0 µL) and dry EtOH (6.5 mL). Purification *via* flash column chromatography yielded ***EE*-1** as a yellow solid (104 mg, 0.32 mmol, 67%).

^{1}H NMR (400 MHz, $(CD_3)_2SO$): δ = 10.66 (br s, 1H), 8.18 (s, 1H), 7.99 (d, *J* = 7.9 Hz, 1H), 7.95 – 7.86 (m, 3H), 7.79 – 7.72 (m, 2H), 7.66 – 7.58 (m, 3H), 7.52 (d, *J* = 7.7 Hz, 1H), 7.25 (t, *J* = 7.7 Hz, 1H), 6.92 – 6.82 (m, 2H).
^{13}C{^{1}H} NMR (101 MHz, $(CD_3)_2SO$): δ = 168.5, 152.0, 151.8, 143.2, 135.8, 134.6, 132.1, 131.9, 130.5, 130.1, 129.6, 128.7, 123.9, 122.7, 122.6, 122.4, 121.2, 120.7, 110.3.
HRMS-ESI (ESI+): calculated for $C_{21}H_{15}N_3OH^+$ $[M+H]^+$ 326.1288, found 326.1287.

Azobenzene-Oxindole Dyad 2 (*EE*-2)

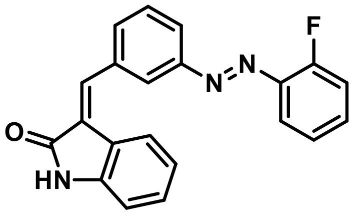

***EE*-2**
$C_{21}H_{14}FN_3O$
Mw = 343.36 g/mol

Synthesized following general procedure **C**, using oxindole (56.7 mg, 0.43 mmol, 1.05 eq.), **16** (95.2 mg, 0.40 mmol, 1.00 eq.), piperidine (40.0 µL) and dry EtOH (8.0 mL). Purification *via* filtration yielded ***EE*-2** as a pale yellow solid (92.8 mg, 0.27 mmol, 68%).

^{1}H NMR (600 MHz, $(CD_3)_2SO$): δ = 10.66 (br s, 1H), 8.20 (s, 1H), 8.01 (d, *J* = 8.0 Hz, 1H), 7.92 (d, *J* = 7.5 Hz, 1H), 7.80 – 7.74 (m, 2H), 7.73 (s, 1H), 7.69 – 7.63 (m, 1H), 7.55 – 7.50 (m, 2H), 7.38 (t, *J* = 7.7 Hz, 1H), 7.25 (t, *J* = 7.7 Hz, 1H), 6.90 (d, *J* = 7.8 Hz, 1H), 6.85 (t, *J* = 7.6 Hz, 1H).
^{13}C{^{1}H} NMR (151 MHz, $(CD_3)_2SO$): δ = 168.4, 159.5 (d), 152.2, 143.2, 139.7 (d), 135.8, 134.5, 134.0 (d), 132.6, 130.6, 130.2, 128.7, 125.1 (d), 124.2, 122.6, 122.5, 121.3, 120.6, 117.5, 117.4 (d), 110.3.
^{19}F NMR (565 MHz, $(CD_3)_2SO$): δ = -124.5.
HRMS-ESI (ESI+): calculated for $C_{21}H_{14}FN_3ONa^+$ $[M+Na]^+$ 366.1013, found 366.1013.

Azobenzene-Oxindole Dyad 3 (*EE*-3)

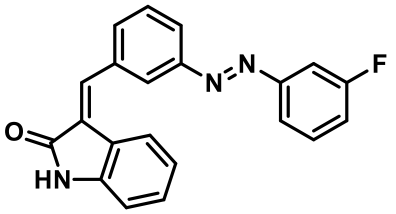

***EE*-3**
$C_{21}H_{14}FN_3O$
Mw = 343.36 g/mol

Synthesized following general procedure **C**, using oxindole (34.2 mg, 0.26 mmol, 1.15 eq.), **17** (54.6 mg, 0.23 mmol, 1.00 eq.), piperidine (40.0 µL) and dry EtOH (6.0 mL). Purification *via* filtration yielded ***EE*-3** as a yellow solid (72.7 mg, 0.21 mmol, 93%).

^{1}H NMR (600 MHz, $(CD_3)_2SO$): δ = 10.66 (br s, 1H), 8.20 (s, 1H), 8.01 (d, *J* = 7.9 Hz, 1H), 7.92 (d, *J* = 7.5 Hz, 1H), 7.82 (d, *J* = 7.8 Hz, 1H), 7.78 (t, *J* = 7.8 Hz, 1H), 7.73 (s, 1H), 7.72 – 7.65 (m, 2H), 7.50 (d, *J* = 7.7 Hz, 1H), 7.47 (td, *J* = 8.4, 2.7 Hz, 1H), 7.25 (t, *J* = 7.7 Hz, 1H), 6.90 (d, *J* = 7.8 Hz, 1H), 6.86 (t, *J* = 7.7 Hz, 1H).
^{13}C{^{1}H} NMR (151 MHz, $(CD_3)_2SO$): δ = 168.4, 162.7 (d), 153.4 (d), 151.8, 143.2, 135.9, 134.5, 132.5, 131.4 (d), 130.6, 130.2, 128.8, 124.0, 122.9, 122.4, 121.3, 120.6 (d), 120.6, 118.5 (d), 110.3, 107.8 (d).
^{19}F NMR (565 MHz, $(CD_3)_2SO$): δ = -111.6.
HRMS-ESI (ESI+): calculated for $C_{21}H_{14}FN_3OH^+$ $[M+H]^+$ 344.1194, found 344.1194.

Azobenzene-Oxindole Dyad 4 (*EE*-4)

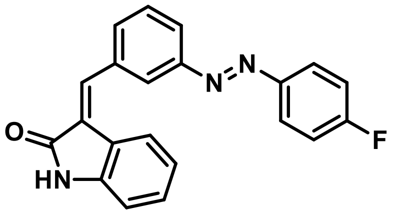

***EE*-4**
$C_{21}H_{14}FN_3O$
Mw = 343.36 g/mol

Synthesized following general procedure **C**, using oxindole (17.2 mg, 0.13 mmol, 1.50 eq.), **18** (20.5 mg, 0.09 mmol, 1.00 eq.), piperidine (40.0 µL) and dry EtOH (2.0 mL). Purification *via* flash column chromatography yielded ***EE*-4** as a yellow solid (18.8 mg, 0.05 mmol, 64%).

^{1}H NMR (400 MHz, $(CD_3)_2SO$): δ = 10.66 (br s, 1H), 8.17 (s, 1H), 8.04 – 7.96 (m, 3H), 7.89 (d, *J* = 7.6 Hz, 1H), 7.79 – 7.72 (m, 2H), 7.51 (d, *J* = 7.8 Hz, 1H), 7.46 (t, *J* = 8.8 Hz, 2H), 7.25 (t, *J* = 7.7 Hz, 1H), 6.92 – 6.83 (m, 2H).
^{13}C{^{1}H} NMR (101 MHz, $(CD_3)_2SO$): δ = 168.4, 150.3 (d), 143.2, 135.8, 134.6, 132.1, 130.5, 130.1, 128.7, 125.2, 125.1, 123.8, 122.6, 122.4, 121.2, 120.7, 116.7, 116.4, 110.3.
^{19}F NMR (376 MHz, $(CD_3)_2SO$): δ = -108.5.
HRMS-ESI (ESI+): calculated for $C_{21}H_{14}FN_3OH^+$ $[M+H]^+$ 344.1194, found 344.1195.

Azobenzene-Oxindole Dyad 5 (*EE*-5)

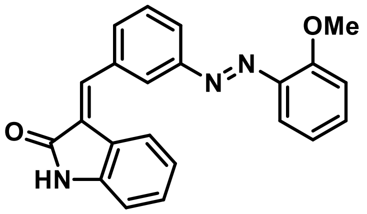

***EE*-5**
$C_{22}H_{17}N_3O_2$
Mw = 355.40 g/mol

Synthesized following general procedure **C**, using oxindole (16.8 mg, 0.13 mmol, 1.25 eq.), **19** (25.4 mg, 0.10 mmol, 1.00 eq.), piperidine (40.0 µL) and dry EtOH (3.0 mL). Purification *via* filtration yielded ***EE*-5** as an orange/red solid (24.6 mg, 0.07 mmol, 69%).

^{1}H NMR (400 MHz, $(CD_3)_2SO$): δ = 10.66 (br s, 1H), 8.11 (s, 1H), 7.94 (d, *J* = 8.2 Hz, 1H), 7.86 (d, *J* = 7.6 Hz, 1H), 7.77 – 7.71 (m, 2H), 7.59 – 7.51 (m, 3H), 7.32 – 7.22 (m, 2H), 7.06 (t, *J* = 7.6 Hz, 1H), 6.90 (d, *J* = 7.8 Hz, 1H), 6.85 (t, *J* = 7.7 Hz, 1H), 3.95 (s, 3H).
^{13}C{^{1}H} NMR (101 MHz, $(CD_3)_2SO$): δ = 168.5, 157.0, 152.6, 143.2, 141.3, 135.7, 134.7, 133.5, 131.8, 130.5, 130.0, 128.6, 124.0, 122.5, 122.4, 121.3, 120.7, 120.6, 116.3, 113.7, 110.3, 56.1.
HRMS-ESI (ESI+): calculated for $C_{22}H_{17}N_3O_2H^+$ $[M+H]^+$ 356.1394, found 356.1394.

Azobenzene-Oxindole Dyad 6 (*EE*-6)

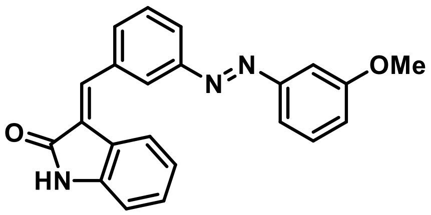

***EE*-6**

$C_{22}H_{17}N_3O_2$

Mw = 355.40 g/mol

Synthesized following general procedure **C**, using oxindole (39.8 mg, 0.30 mmol, 1.00 eq.), **20** (78.6 mg, 0.31 mmol, 1.05 eq.), piperidine (40.0 µL) and dry EtOH (7.0 mL). Purification *via* filtration yielded ***EE*-6** as a yellow solid (98.4 mg, 0.28 mmol, 93%).

^{1}H NMR (400 MHz, $(CD_3)_2SO$): δ = 10.66 (br s, 1H), 8.18 (s, 1H), 7.99 (d, *J* = 8.1 Hz, 1H), 7.89 (d, *J* = 7.6 Hz, 1H), 7.80 – 7.71 (m, 2H), 7.57 – 7.49 (m, 3H), 7.43 (s, 1H), 7.25 (t, *J* = 7.7 Hz, 1H), 7.18 (s, 1H), 6.92 – 6.82 (m, 2H), 3.86 (s, 3H).
^{13}C{^{1}H} NMR (101 MHz, $(CD_3)_2SO$): δ = 168.4, 160.1, 153.1, 152.0, 143.2, 135.8, 134.6, 132.1, 130.5, 130.4, 130.1, 123.9, 123.3, 122.6, 122.4, 121.2, 120.2, 118.2, 116.5, 110.3, 106.1, 55.4.
HRMS-ESI (ESI+): calculated for $C_{22}H_{17}N_3O_2H^+$ $[M+H]^+$ 356.1394, found 356.1398.

Azobenzene-Oxindole Dyad 7 (*EE*-7)

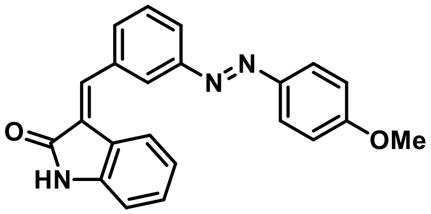

***EE*-7**

$C_{22}H_{17}N_3O_2$

Mw = 355.40 g/mol

Synthesized following general procedure **C**, using oxindole (17.8 mg, 0.13 mmol, 1.25 eq.), **21** (27.0 mg, 0.11 mmol, 1.00 eq.), piperidine (40.0 µL) and dry EtOH (2.5 mL). Purification *via* filtration yielded ***EE*-7** as a yellow solid (31.2 mg, 0.09 mmol, 82%).

^{1}H NMR (400 MHz, $(CD_3)_2SO$): δ = 10.65 (br s, 1H), 8.13 (s, 1H), 7.97 – 7.89 (m, 3H), 7.84 (d, *J* = 7.4 Hz, 1H), 7.76 – 7.70 (m, 2H), 7.53 (d, *J* = 7.7 Hz, 1H), 7.25 (t, *J* = 7.7 Hz, 1H), 7.16 (d, *J* = 9.0 Hz, 2H), 6.92 – 6.83 (m, 2H), 3.88 (s, 3H).
^{13}C{^{1}H} NMR (101 MHz, $(CD_3)_2SO$): δ = 168.4, 161.8, 152.2, 146.1, 143.3, 135.7, 134.8, 133.7, 131.3, 130.5, 130.0, 125.3, 124.7, 123.6, 122.4, 122.3, 121.2, 114.7, 110.3, 56.8.
HRMS-ESI (ESI+): calculated for $C_{22}H_{17}N_3O_2H^+$ $[M+H]^+$ 356.1394, found 356.1393.

General Procedure **D**

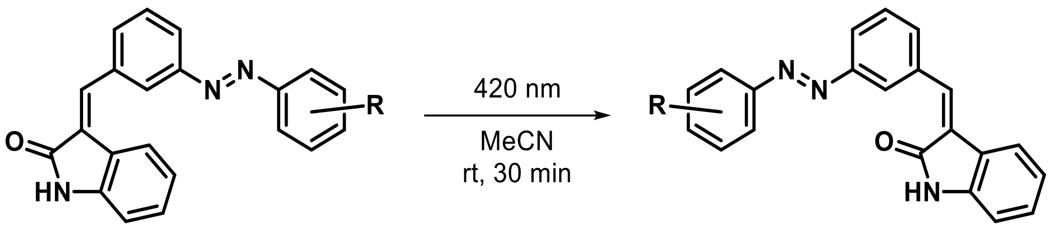

***EE*-1/*EE*-7** ***EZ*-1/*EZ*-7**

***EE*-1** or ***EE*-7** was dissolved in MeCN and irradiated in a photoreactor with 420 nm light for 30 min. The solution was then heated to 80 ºC for 16 h and ***EZ*-1** or ***EZ*-7** obtained *via* flash column chromatography using a gradient starting from *n*-pentane to EtOAc.

***EZ*-1**

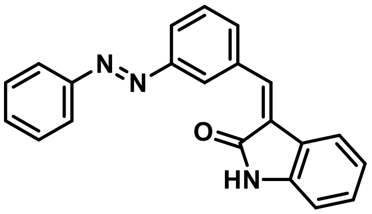

***EZ*-1**
$C_{21}H_{15}N_3O$
Mw = 325.37 g/mol

***EZ*-1** was obtained *via* flash column chromatography as an orange/yellow solid.

^{1}H NMR (600 MHz, $(CD_3)_2SO$): δ = 10.67 (br s, 1H), 8.90 (s, 1H), 8.55 (d, *J* = 8.4 Hz, 1H), 7.98 (s, 1H), 7.96 – 7.91 (m, 3H), 7.77 (d, *J* = 7.5 Hz, 1H), 7.69 (t, *J* = 7.8 Hz, 1H), 7.66 – 7.58 (m, 3H), 7.25 (t, *J* = 7.6 Hz, 1H), 7.02 (t, *J* = 7.5 Hz, 1H), 6.85 (d, *J* = 7.7 Hz, 1H).
^{13}C{^{1}H} NMR (101 MHz, $(CD_3)_2SO$): δ = 167.0, 151.9, 151.9, 141.1, 135.6, 135.2, 134.4, 131.7, 129.6, 129.4, 129.3, 128.0, 126.6, 124.7, 123.3, 122.6, 121.2, 120.2, 109.5.
HRMS-ESI (ESI+): calculated for $C_{21}H_{15}N_3ONa^+$ $[M+Na]^+$ 348.1107, found 348.1107.

***EZ*-7**

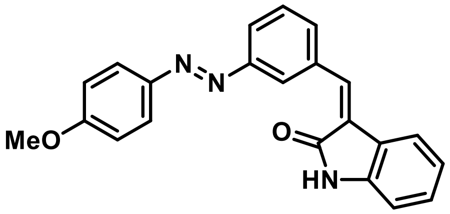

***EZ*-7**
$C_{22}H_{17}N_3O_2$
Mw = 355.40 g/mol

***EZ*-7** was obtained *via* flash column chromatography as an orange solid.

^{1}H NMR (400 MHz, $(CD_3)_2SO$): δ = 10.65 (s, 1H), 8.83 (s, 1H), 8.51 (d, *J* = 7.8 Hz, 1H), 7.96 (s, 1H), 7.93 (d, *J* = 9.0 Hz, 2H), 7.89 (d, *J* = 8.0 Hz, 1H), 7.76 (d, *J* = 7.5 Hz, 1H), 7.65 (t, *J* = 7.8 Hz, 1H), 7.24 (t, *J* = 7.6 Hz, 1H), 7.17 (d, *J* = 9.0 Hz, 2H), 7.02 (t, *J* = 7.6 Hz, 1H), 6.85 (d, *J* = 7.7 Hz, 1H), 3.89 (s, 3H).
^{13}C{^{1}H} NMR (101 MHz, $(CD_3)_2SO$): δ = 167.0, 162.2, 152.0, 146.2, 141.0, 135.8, 135.1, 133.8, 129.3, 129.2, 127.8, 126.3, 124.7, 124.7, 123.0, 121.2, 120.1, 114.7, 109.5, 55.7.
HRMS-ESI (ESI+): calculated for $C_{22}H_{17}N_3OH^+$ $[M+H]^+$ 356.1394, found 356.1389.

NMR Experiments

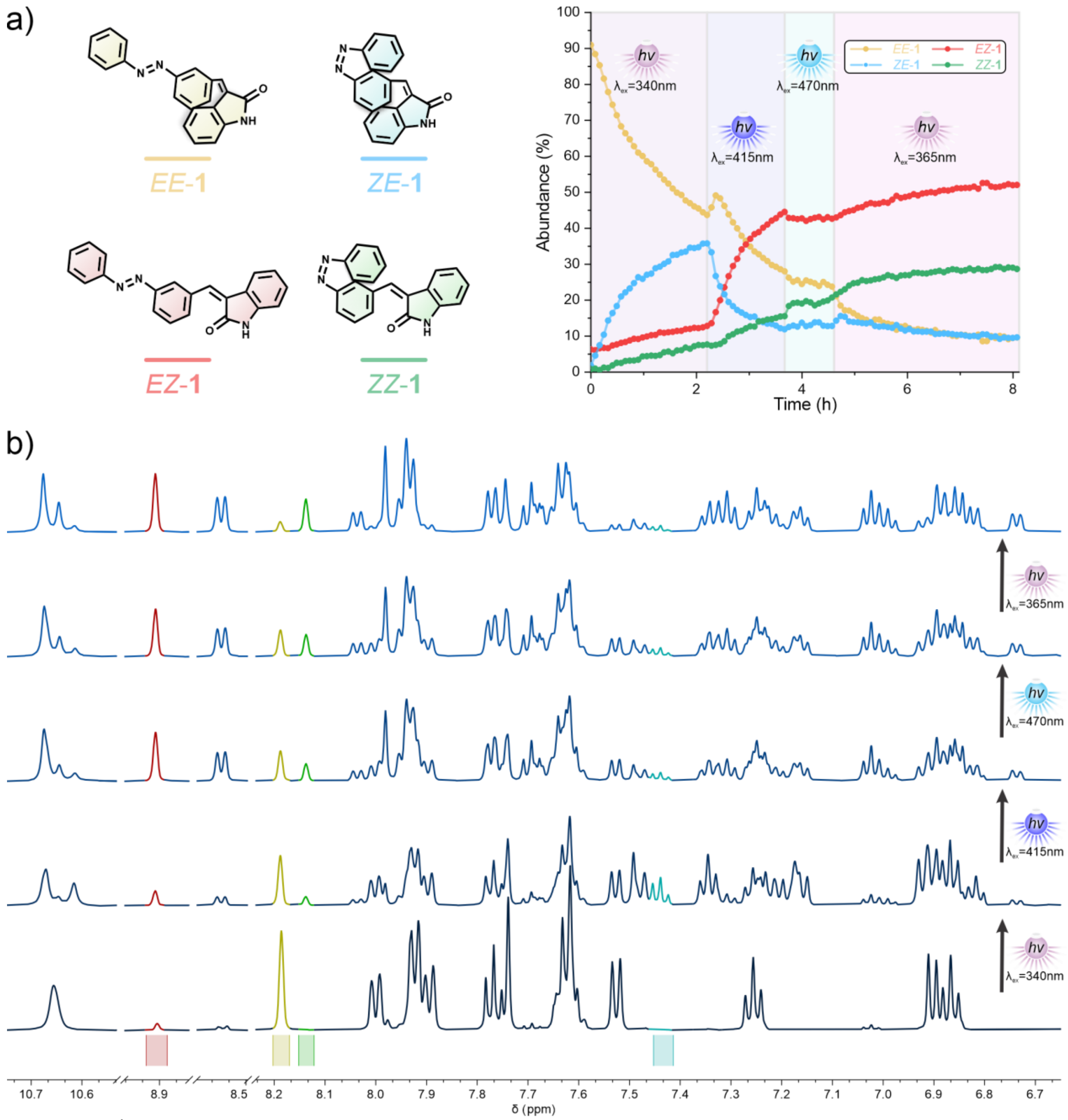

Figure S1: ^{1}H NMR ($(CD_3)_2SO$, 25 °C) **a)** Kinetic traces of the different isomers of **1** obtained by following the *in-situ* ^{1}H NMR irradiation sequence λ_{irr} = 340 → 415 → 470 → 365 nm. **b)** Stacked spectra of the *in-situ* irradiation of **1** following the wavelength irradiation sequence (from the pristine sample at bottom to top) λ_{irr} = 340 → 415 → 470 → 365 nm. The characteristic signals used to follow the kinetic traces are shown color-coded accordingly (***EE*-1** yellow, ***ZE*-1** blue, ***EZ*-1** red, ***ZZ*-1** green). Supporting video 1 shows the ^{1}H NMR spectra evolution.

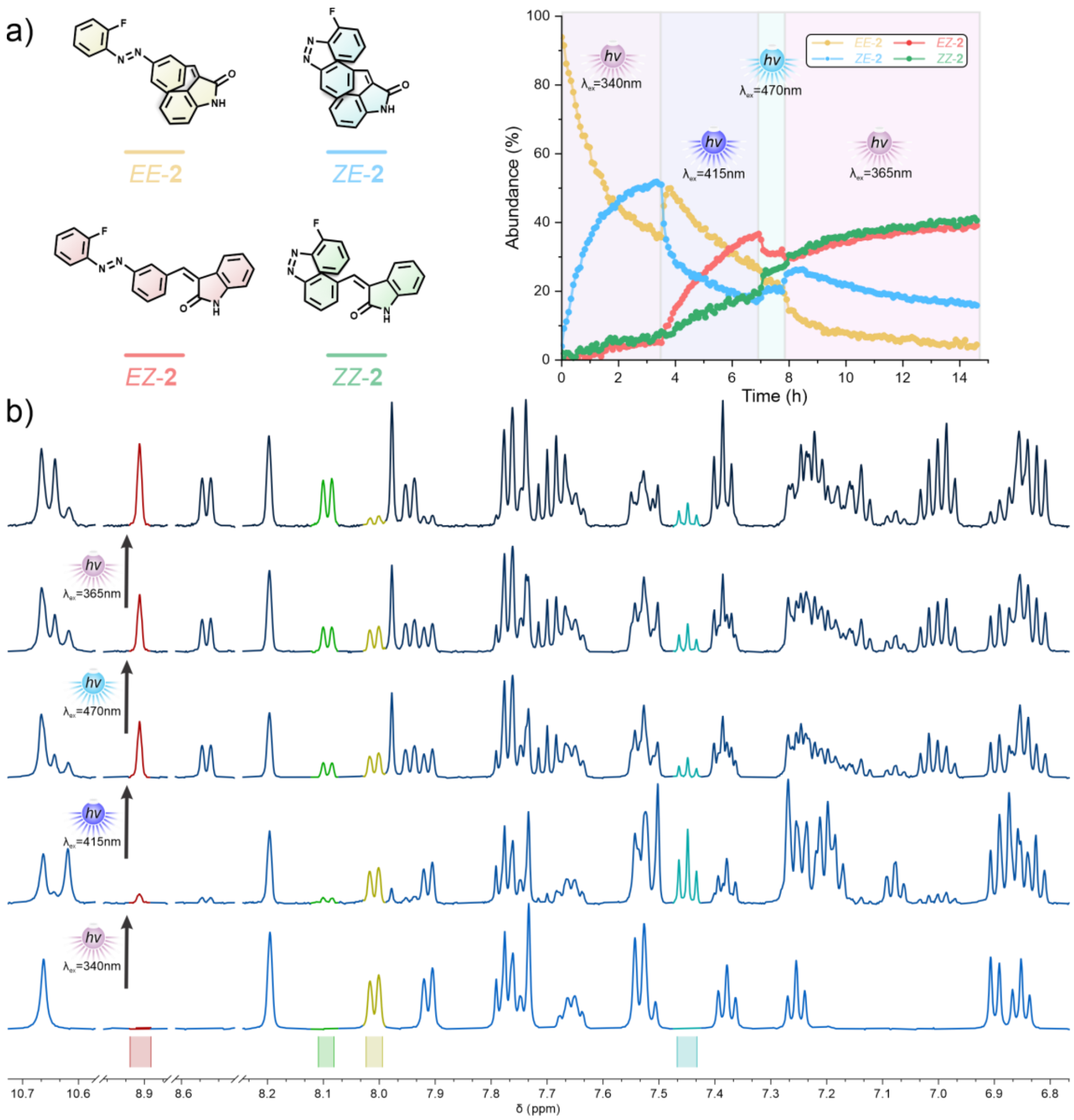

Figure S2: ^{1}H NMR ($(CD_3)_2SO$, 25 °C) **a)** Kinetic traces of the different isomers of **2** obtained by following the *in-situ* ^{1}H NMR irradiation sequence λ_{irr} = 340 → 415 → 470 → 365 nm. **b)** Stacked spectra of the *in-situ* irradiation of **2** following the wavelength irradiation sequence (from the pristine sample at bottom to top) λ_{irr} = 340 → 415 → 470 → 365 nm. The characteristic signals used to follow the kinetic traces are shown color-coded accordingly (***EE*-2** yellow, ***ZE*-2** blue, ***EZ*-2** red, ***ZZ*-2** green). Supporting video 2 shows the ^{1}H NMR spectra evolution.

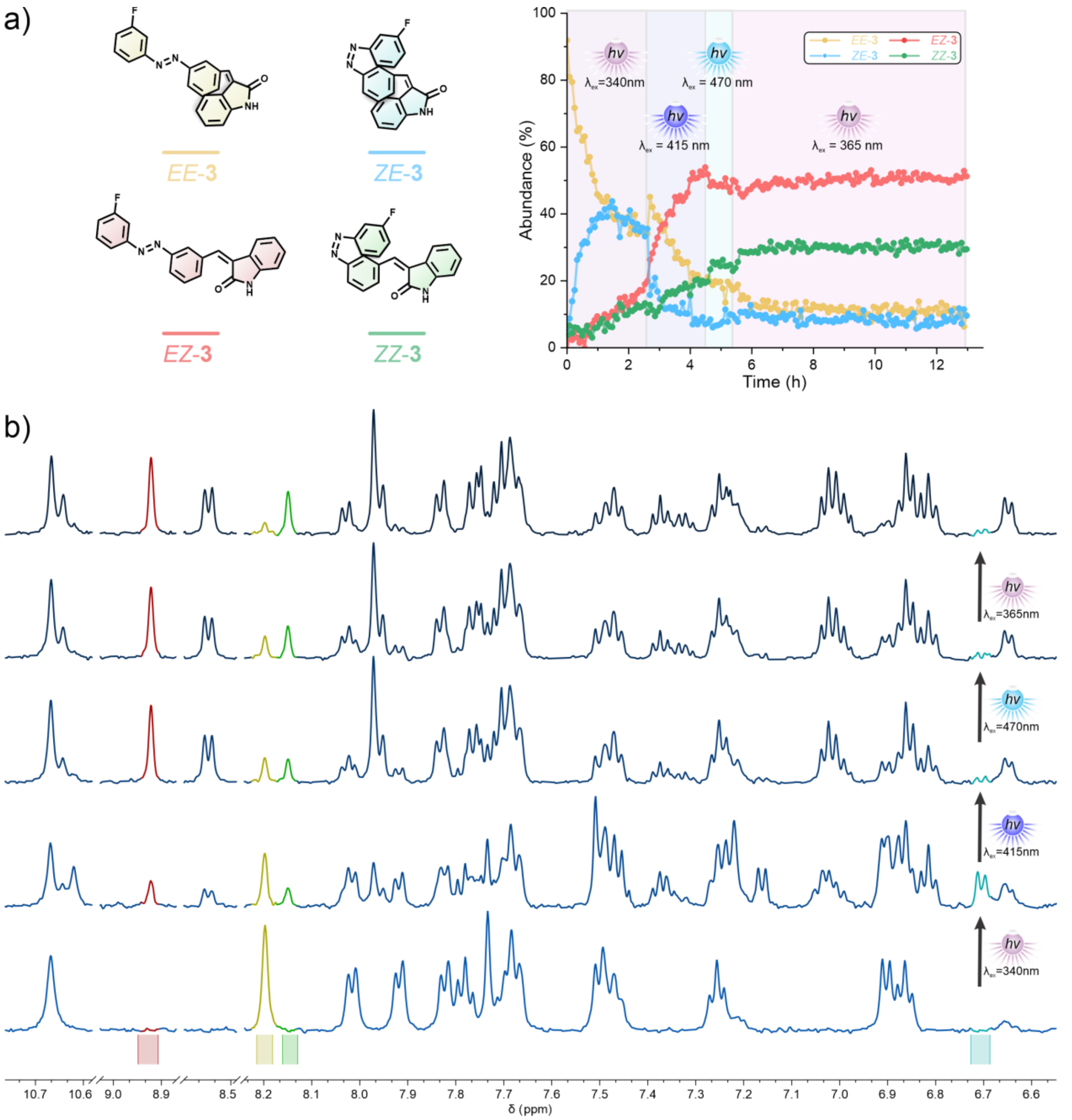

Figure S3: ^{1}H NMR ($(CD_3)_2SO$, 25 °C) **a)** Kinetic traces of the different isomers of **3** obtained by following the *in-situ* ^{1}H NMR irradiation sequence λ_{irr} = 340 → 415 → 470 → 365 nm. **b)** Stacked spectra of the *in-situ* irradiation of **3** following the wavelength irradiation sequence (from the pristine sample at bottom to top) λ_{irr} = 340 → 415 → 470 → 365 nm. The characteristic signals used to follow the kinetic traces are shown color-coded accordingly (***EE*-3** yellow, ***ZE*-3** blue, ***EZ*-3** red, ***ZZ*-3** green). Supporting video 3 shows the ^{1}H NMR spectra evolution.

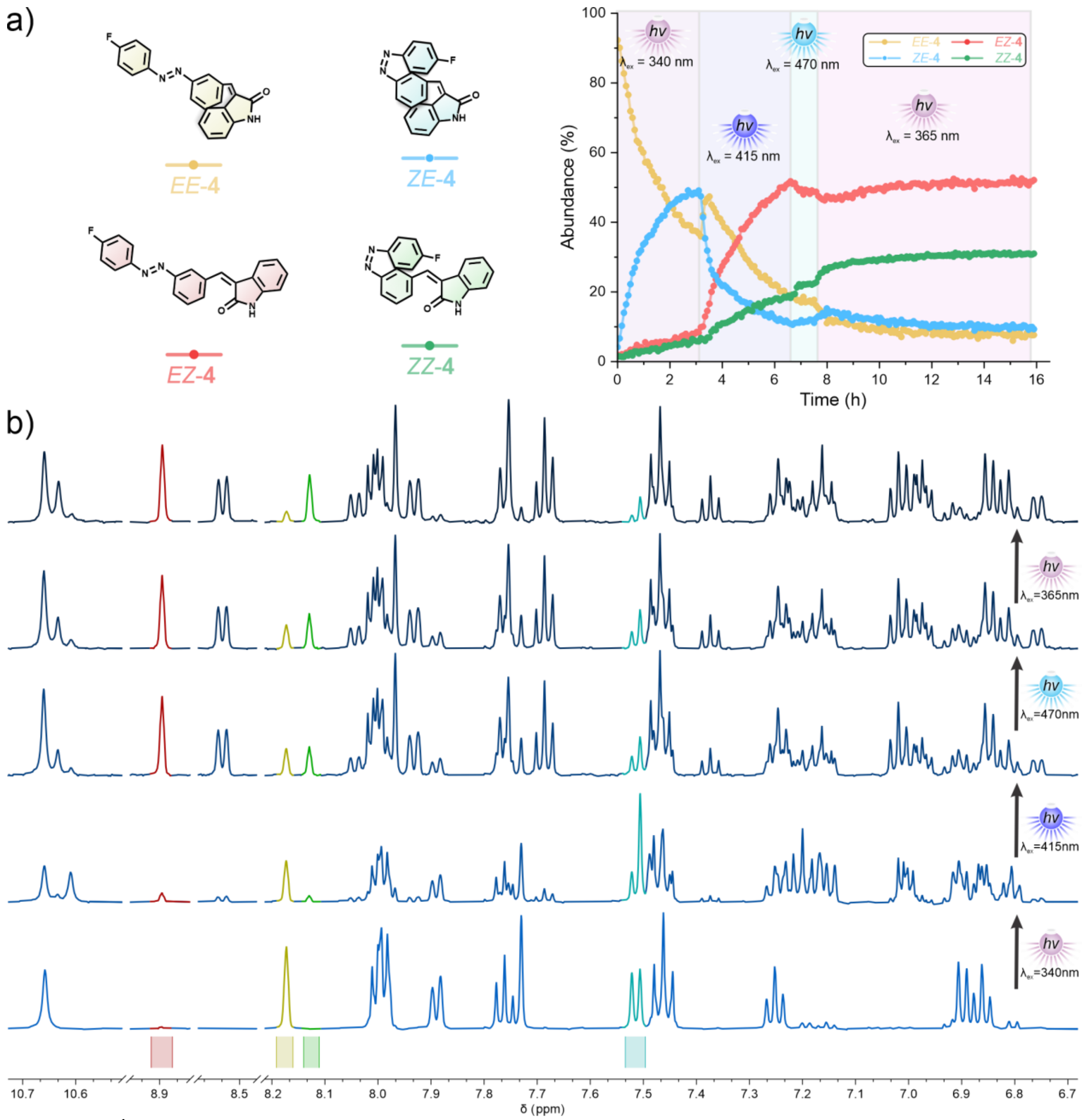

Figure S4: ^{1}H NMR ($(CD_3)_2SO$, 25 °C) **a)** Kinetic traces of the different isomers of **4** obtained by following the *in-situ* ^{1}H NMR irradiation sequence λ_{irr} = 340 → 415 → 470 → 365 nm. **b)** Stacked spectra of the *in-situ* irradiation of **4** following the wavelength irradiation sequence (from the pristine sample at bottom to top) λ_{irr} = 340 → 415 → 470 → 365 nm. The characteristic signals used to follow the kinetic traces are shown color-coded accordingly (***EE*-4** yellow, ***ZE*-4** blue, ***EZ*-4** red, ***ZZ*-4** green). Supporting video 4 shows the ^{1}H NMR spectra evolution. The signal used for the following of for the photoisomer ***EZ*-4** overlaps only with a signal of ***EE*-4**, thus the kinetic analysis was done by subtracting the value of the ***EE*** isomer from the integration.

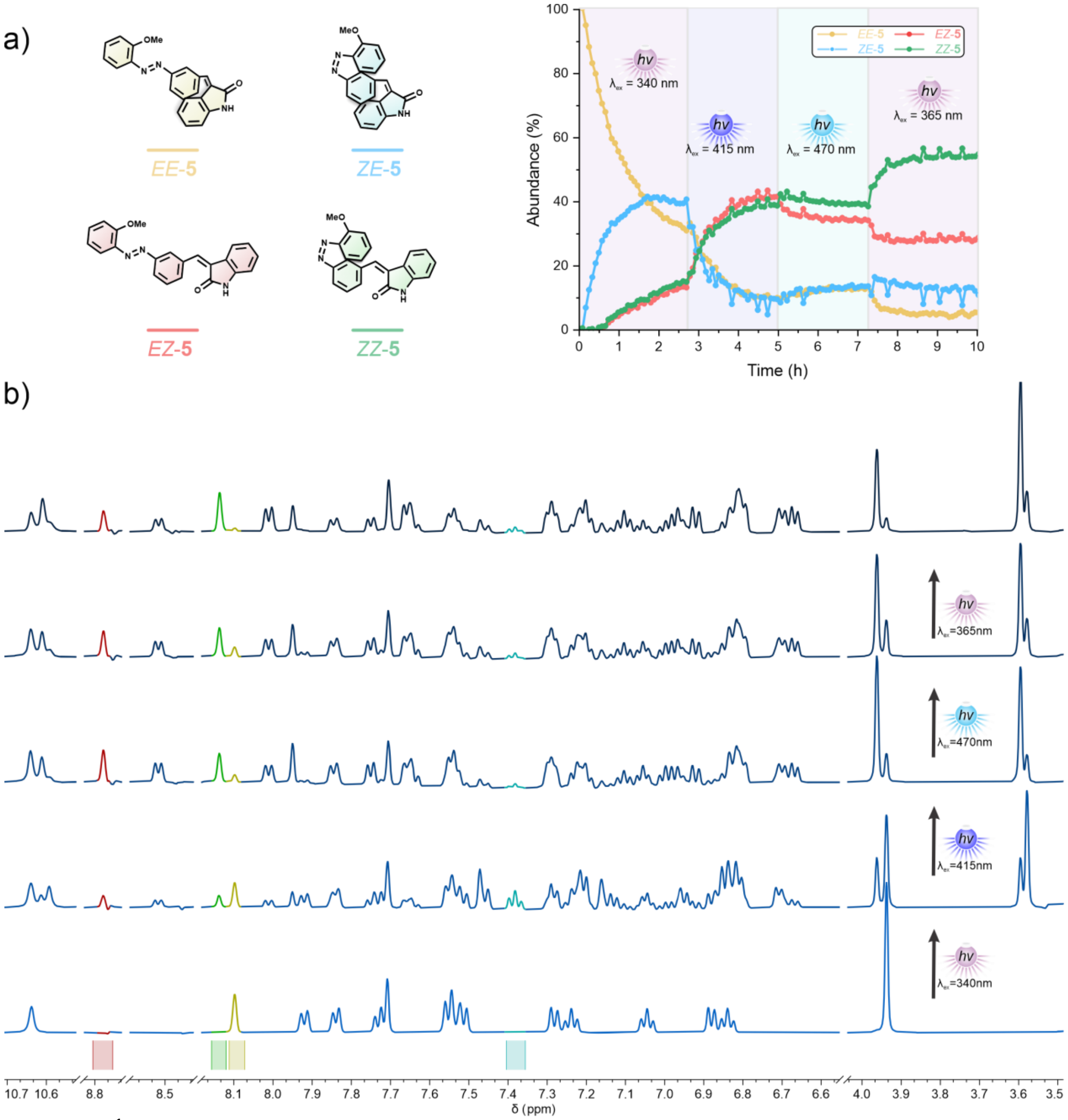

Figure S5: ^{1}H NMR ($(CD_3)_2SO$, 25 °C) **a)** Kinetic traces of the different isomers of **5** obtained by following the *in-situ* ^{1}H NMR irradiation sequence λ_{irr} = 340 → 415 → 470 → 365 nm. **b)** Stacked spectra of the *in-situ* irradiation of **5** following the wavelength irradiation sequence (from the pristine sample at bottom to top) λ_{irr} = 340 → 415 → 470 → 365 nm. The characteristic signals used to follow the kinetic traces are shown color-coded accordingly (***EE*-5** yellow, ***ZE*-5** blue, ***EZ*-5** red, ***ZZ*-5** green). Supporting video 5 shows the ^{1}H NMR spectra evolution.

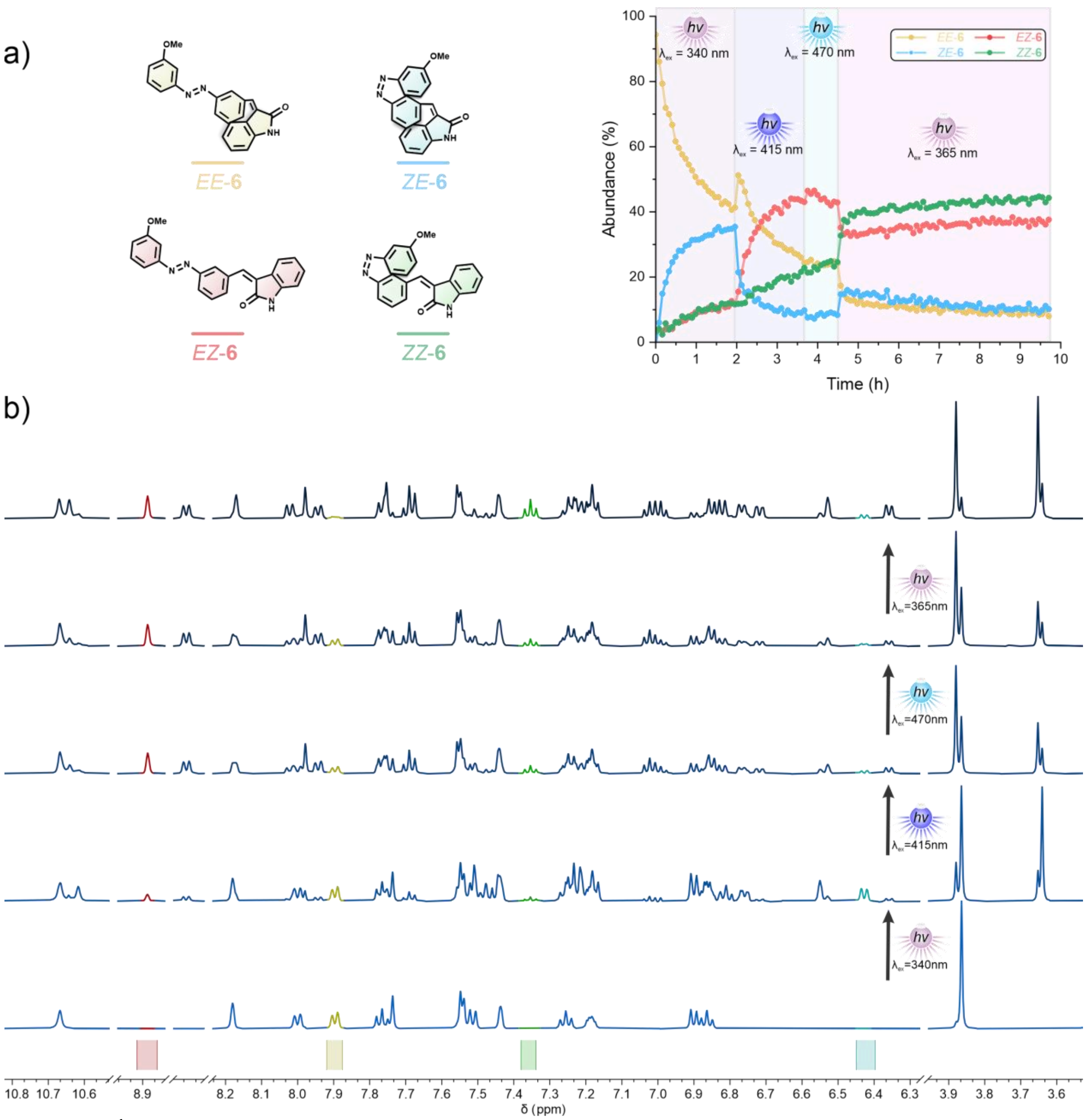

Figure S6: ^{1}H NMR ($(CD_3)_2SO$, 25 °C) **a)** Kinetic traces of the different isomers of **6** obtained by following the *in-situ* ^{1}H NMR irradiation sequence λ_{irr} = 340 → 415 → 470 → 365 nm. **b)** Stacked spectra of the *in-situ* irradiation of **6** following the wavelength irradiation sequence (from the pristine sample at bottom to top) λ_{irr} = 340 → 415 → 470 → 365 nm. The characteristic signals used to follow the kinetic traces are shown color-coded accordingly (***EE*-6** yellow, ***ZE*-6** blue, ***EZ*-6** red, ***ZZ*-6** green). Supporting video 6 shows the ^{1}H NMR spectra evolution.

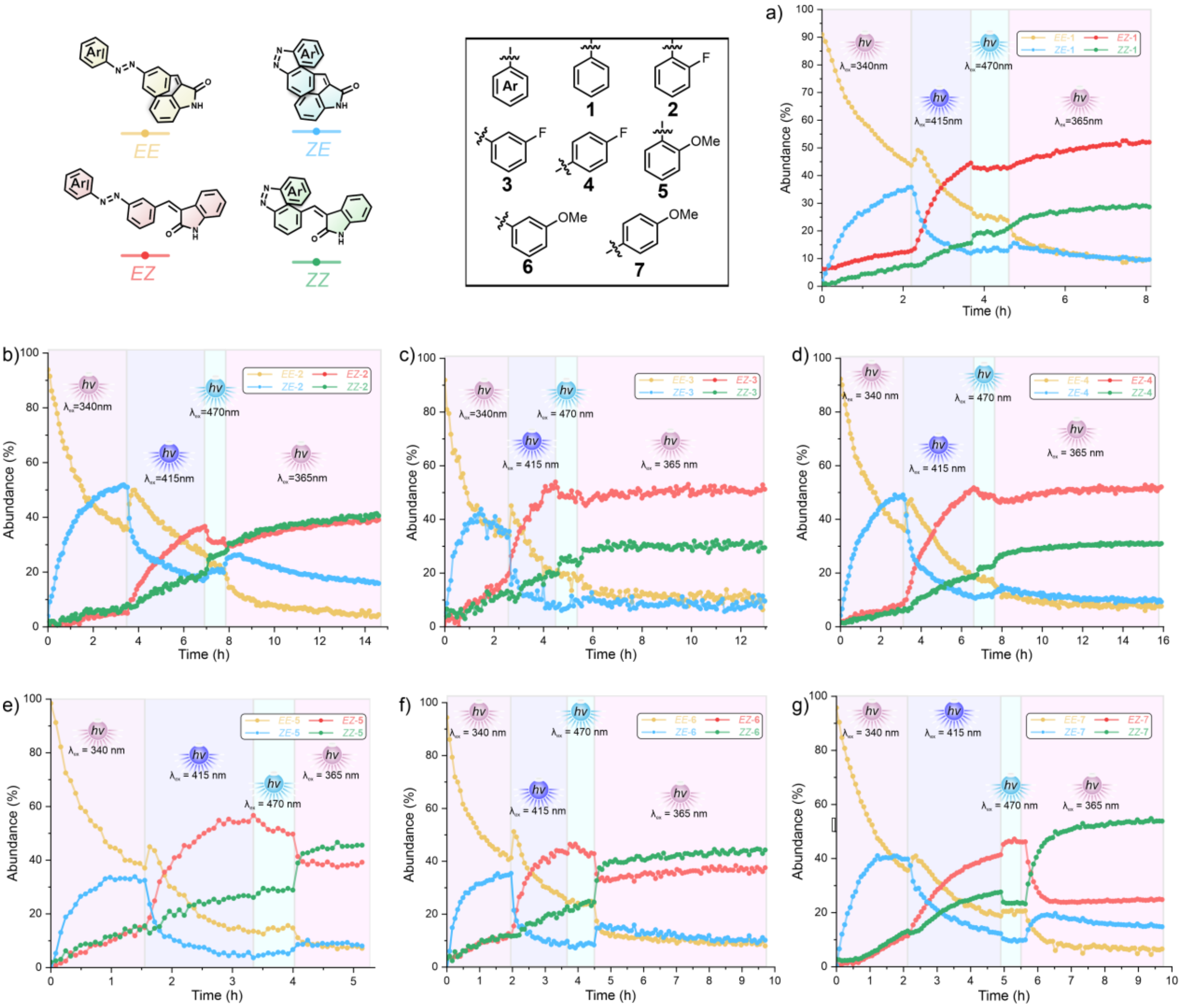

Figure S7: Comparison of the isomerization kinetics of the different AODs. **a)** kinetic traces obtained from the *in-situ* ^{1}H NMR irradiation ($DMSO-d_6$, 4 mmol) using the irradiation sequence 340 → 415 → 470 → 365 nm for **a) 1** (unsubstituted) **b) 2** (2-F substituted), **c) 3** (3-F substituted), **d) 4** (3-F substituted), **e) 5** (2-OMe substituted), **f) 6** (3-OMe substituted), and **g) 7** (4-OMe substituted). Each irradiation was performed until the variation of the isomeric distribution was minimal, which varies depending on the stimuli and the compound.

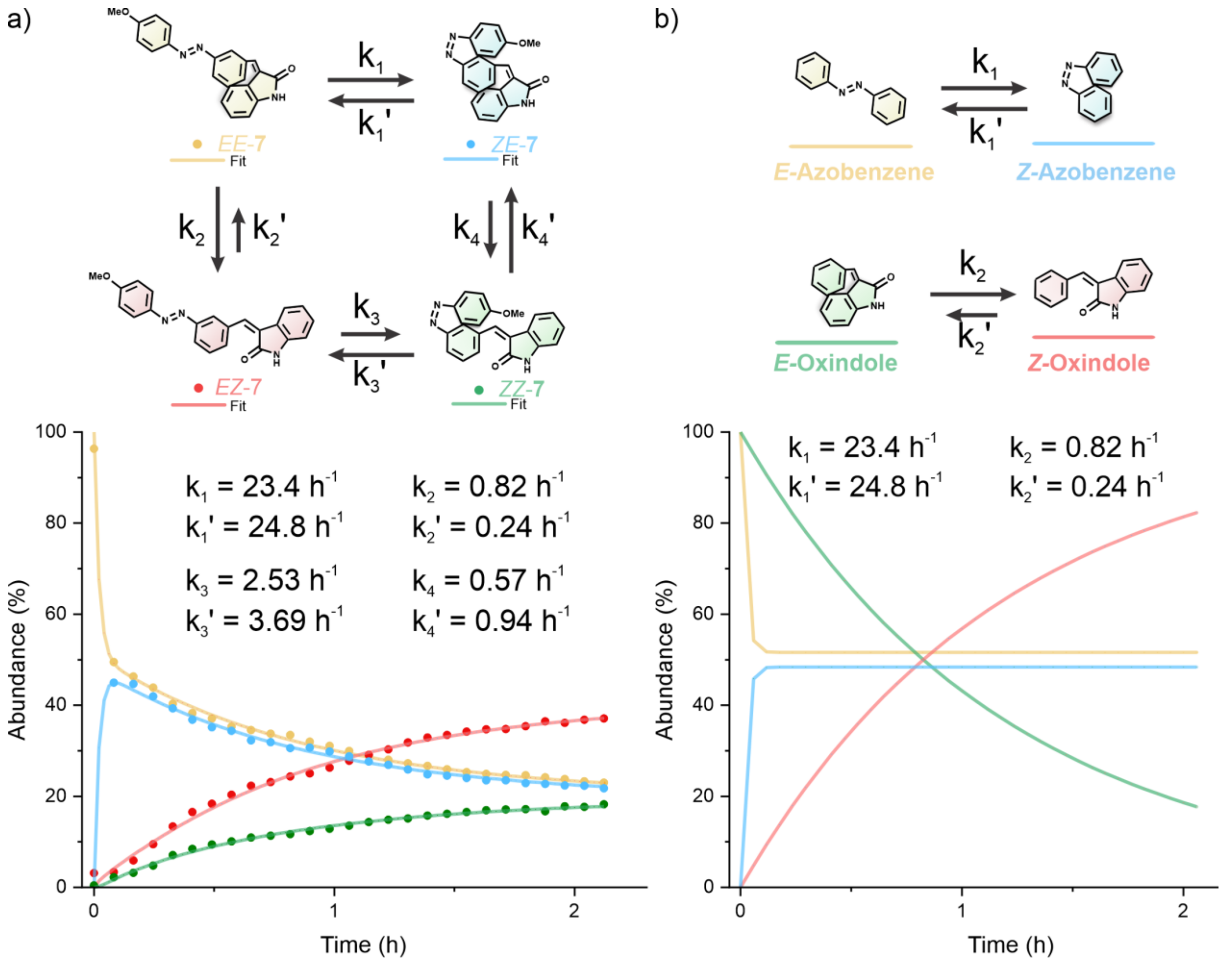

Figure S8: Kinetic analysis of the irradiation of pristine ***EE*-7** with 470 nm light (the data is extracted from the experiment displayed in the first part of main figure 4d and supporting video 11). **a)** Fitting of the **experimental** data to the first order reactions displayed in the reaction network and values of the different observed reaction constants. **b)** Model of the photoisomerization of an azobenzene and an oxindole with the kinetic constraints obtained for their respective isomerizations (k_1 and k_1' for the azobenzene *E* → *Z* isomerization and k_2 and k_2' for the oxindole *E* → *Z* isomerization).

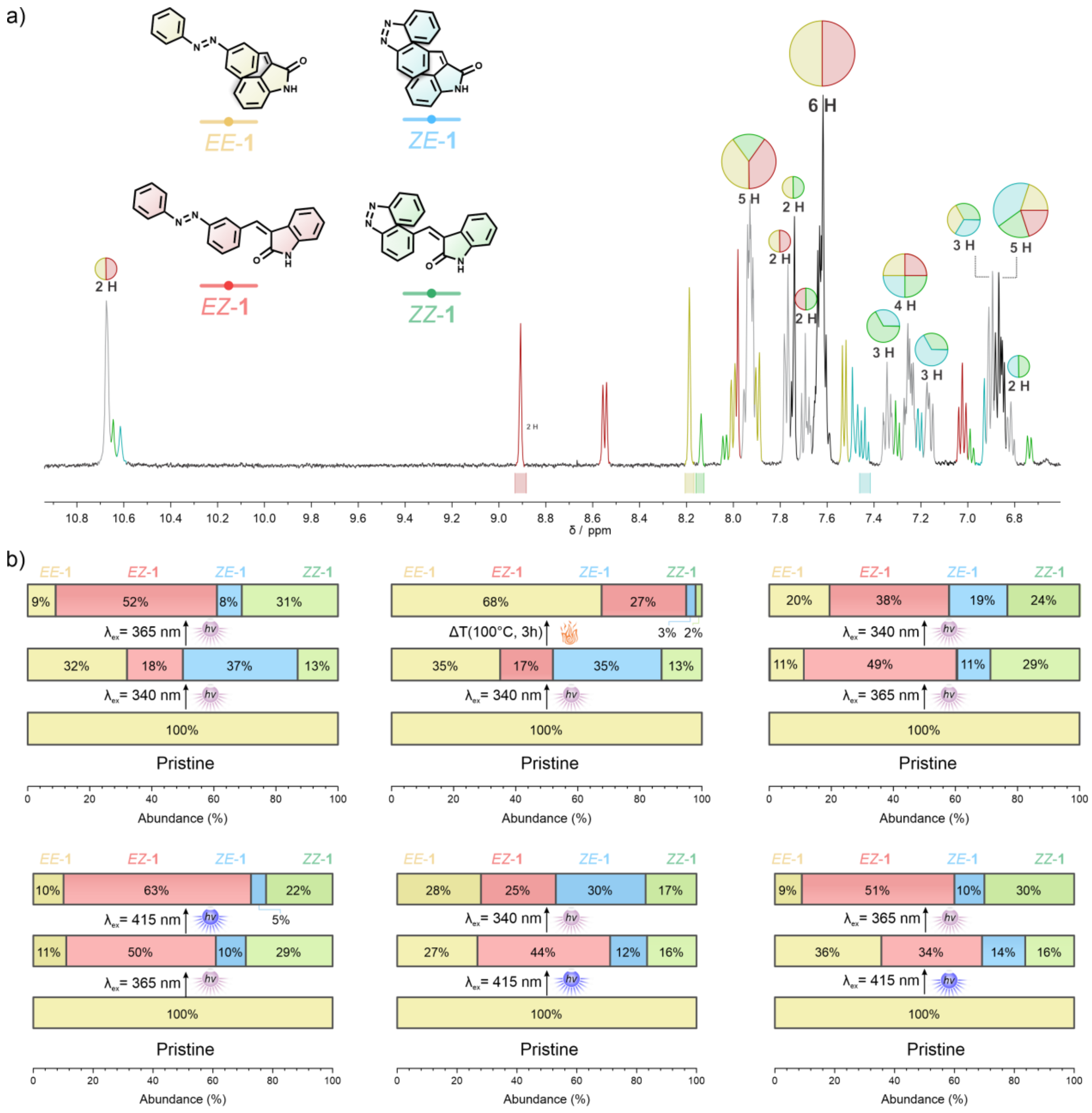

Figure S9: a) ^{1}H NMR ((CD$_3$)$_2$SO, 25 °C) spectra of the different light-generated isomers of **1**. The different signals were assigned by fitting top the formula f(t) = A[***EE*-1**] + B[***ZE*-1**] + C[***EZ*-1**] + D[***ZZ*-1**] and are shown color-coded respectively. Overlapping signals are shown in grey with an associated pie chart indicating the best-fitted combination of profiles. **b)** Isomeric distribution after difference sequences of *ex-situ* irradiation. Each chart starts with the pristine sample containing mainly ***EE*-1**. The following distributions reflect the changes after the indicated stimuli.

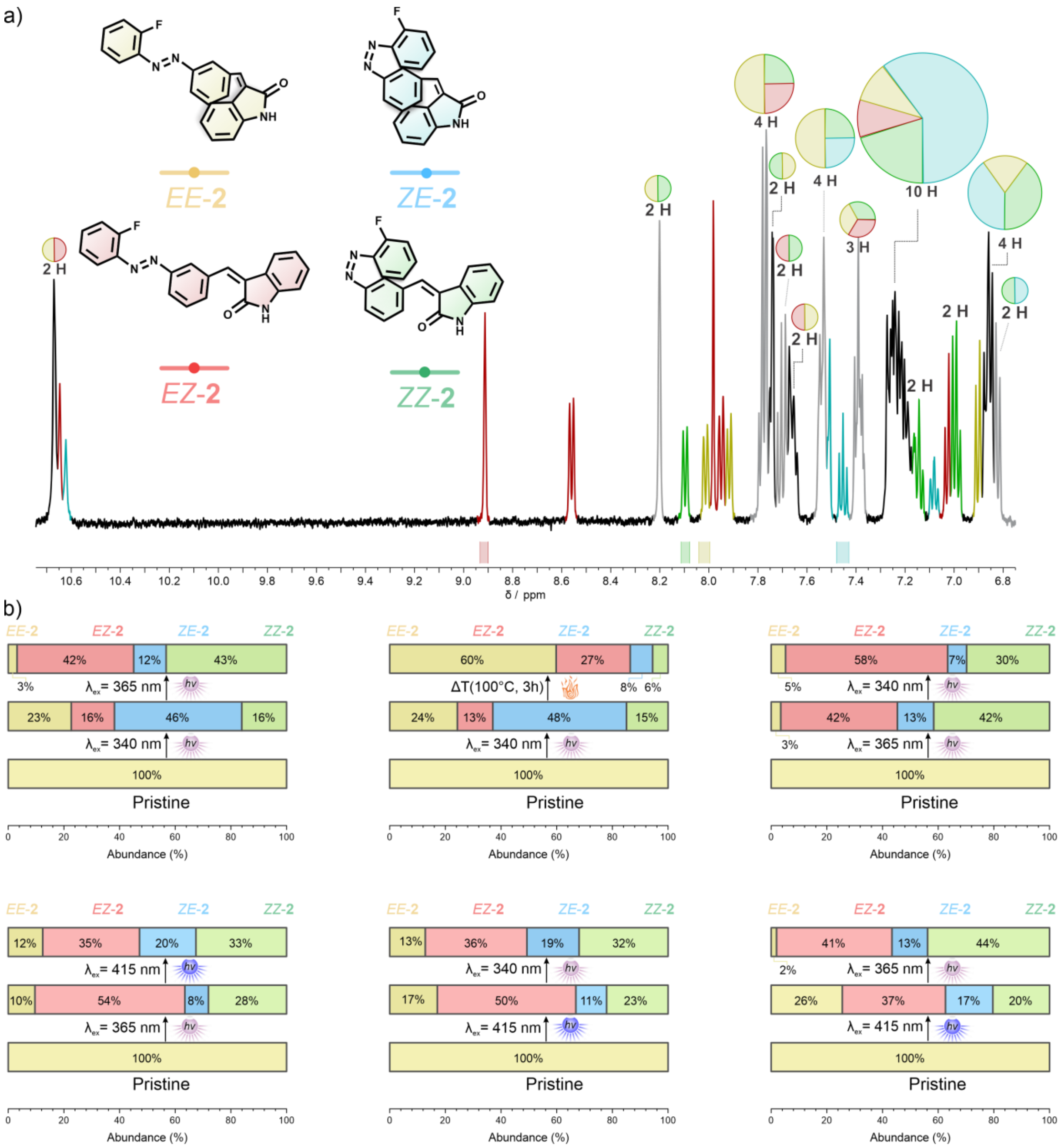

Figure S10: a) ^{1}H NMR ($(CD_3)_2SO$, 25 °C) spectra of the different light-generated isomers of **2**. The different signals were assigned by fitting top the formula f(t) = A[***EE*-2**] + B[***ZE*-2**] + C[***EZ*-2**] + D[***ZZ*-2**] and are shown color-coded respectively. Overlapping signals are shown in grey with an associated pie chart indicating the best-fitted combination of profiles. **b)** Isomeric distribution after difference sequences of *ex-situ* irradiation. Each chart starts with the pristine sample containing mainly ***EE*-2**. The following distributions reflect the changes after the indicated stimuli.

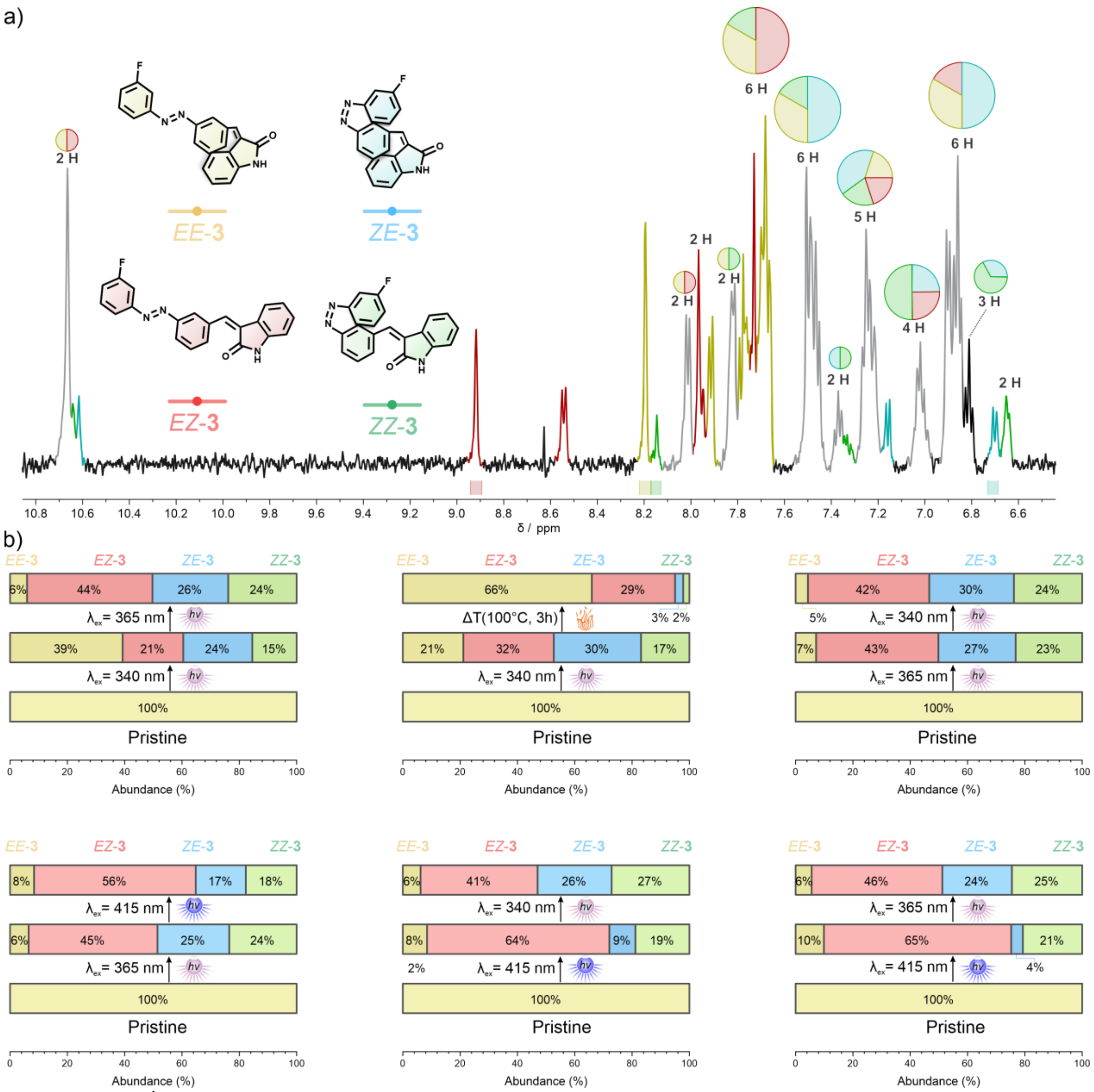

Figure S11: a) ^{1}H NMR ($(CD_3)_2SO$, 25 °C) spectra of the different light-generated isomers of **1**. The different signals were assigned by fitting top the formula f(t) = A[***EE*-3**] + B[***ZE*-3**] + C[***EZ*-3**] + D[***ZZ*-3**] and are shown color-coded respectively. Overlapping signals are shown in grey with an associated pie chart indicating the best-fitted combination of profiles. **b)** Isomeric distribution after difference sequences of *ex-situ* irradiation. Each chart starts with the pristine sample containing mainly ***EE*-3**. The following distributions reflect the changes after the indicated stimuli.

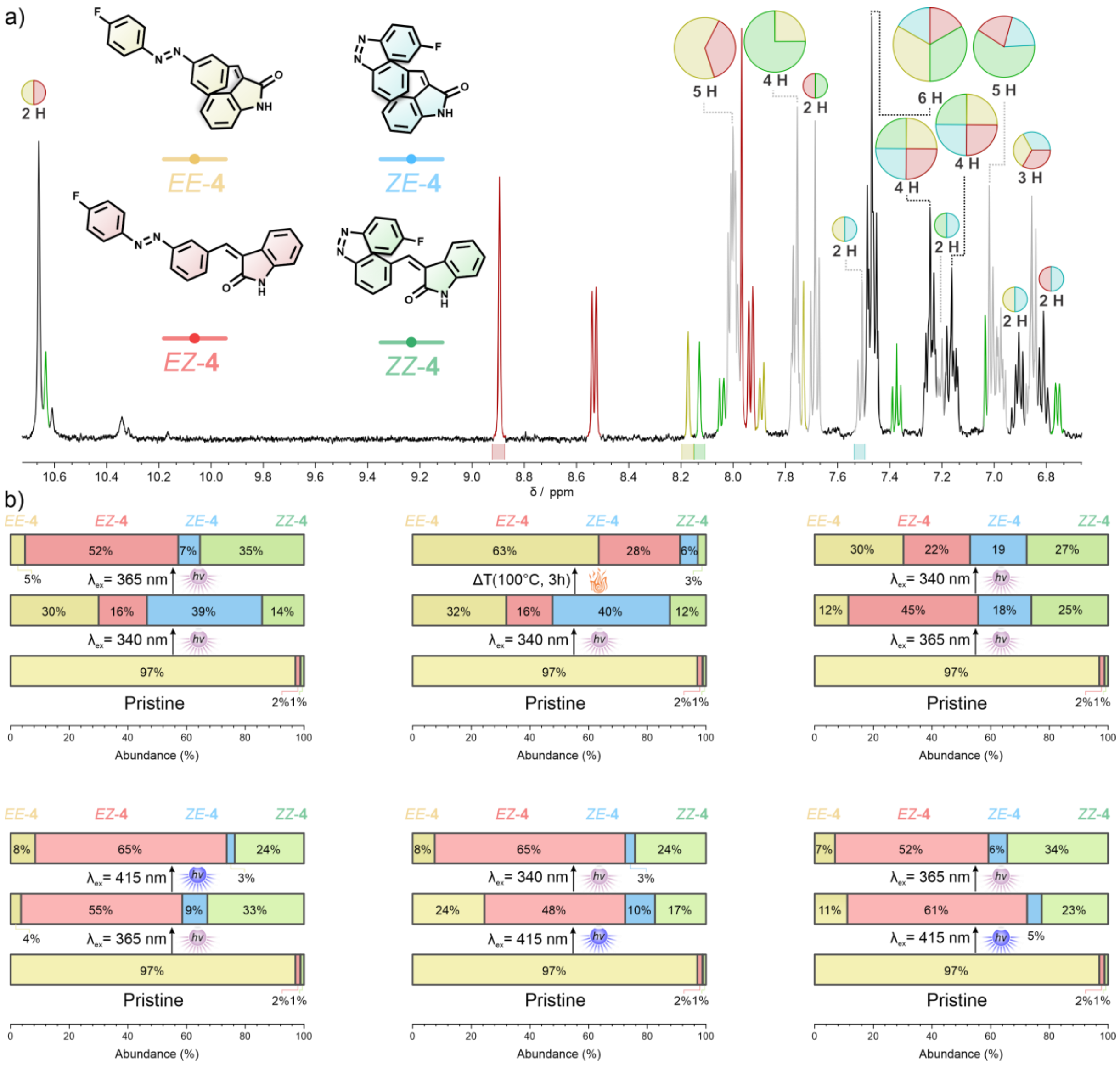

Figure S12: a) ^{1}H NMR ($(CD_3)_2SO$, 25 °C) spectra of the different light-generated isomers of **4**. The different signals were assigned by fitting top the formula f(t) = A[***EE*-4**] + B[***ZE*-4**] + C[***EZ*-4**] + D[***ZZ*-4**] and are shown color-coded respectively. Overlapping signals are shown in grey with an associated pie chart indicating the best-fitted combination of profiles. **b)** Isomeric distribution after difference sequences of *ex-situ* irradiation. Each chart starts with the pristine sample containing mainly ***EE*-4**. The following distributions reflect the changes after the indicated stimuli.

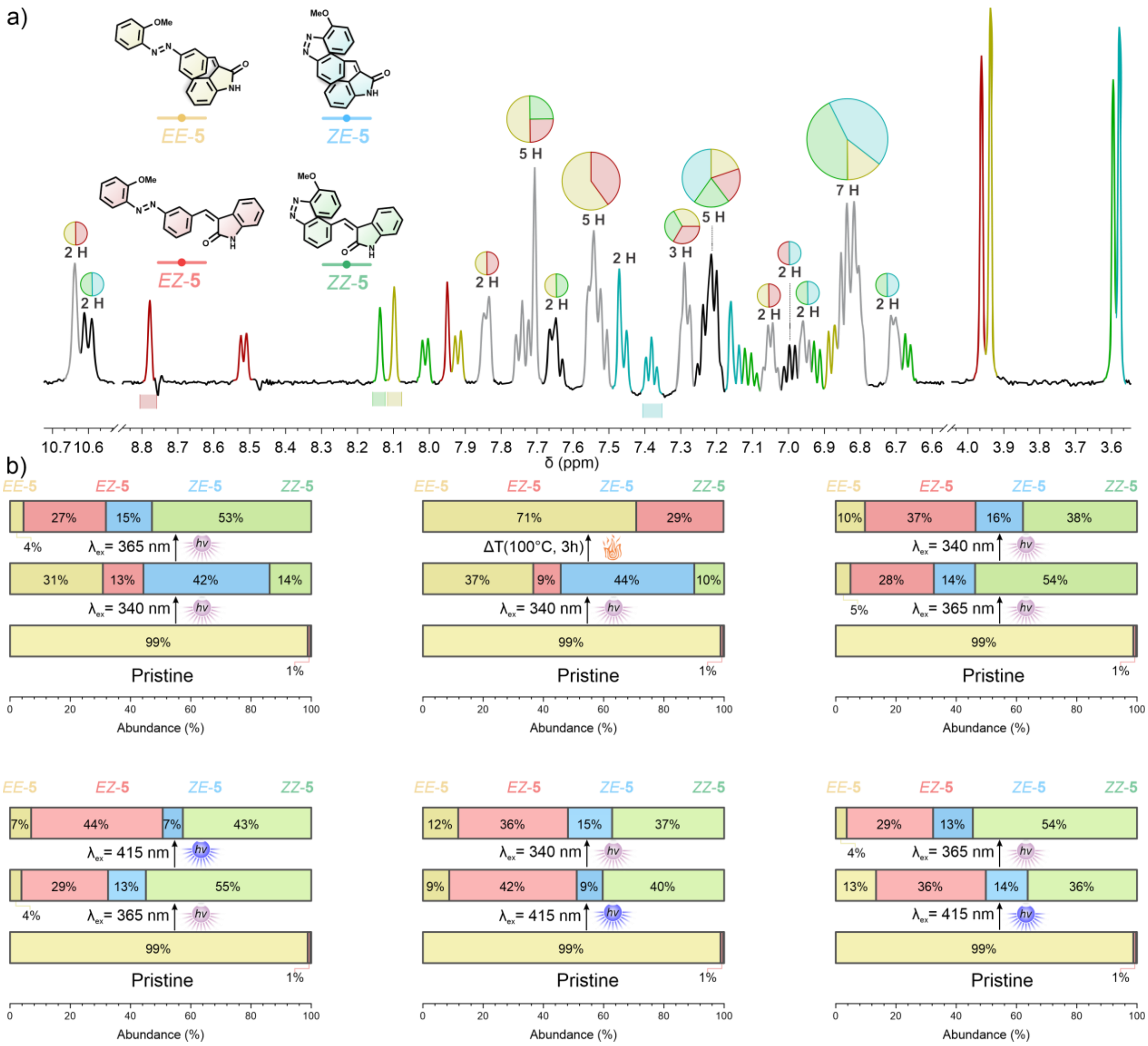

Figure S13: a) ^{1}H NMR ($(CD_3)_2SO$, 25 °C) spectra of the different light-generated isomers of **5**. The different signals were assigned by fitting top the formula f(t) = A[***EE*-5**] + B[***ZE*-5**] + C[***EZ*-5**] + D[***ZZ*-5**] and are shown color-coded respectively. Overlapping signals are shown in grey with an associated pie chart indicating the best-fitted combination of profiles. **b)** Isomeric distribution after difference sequences of *ex-situ* irradiation. Each chart starts with the pristine sample containing mainly ***EE*-5**. The following distributions reflect the changes after the indicated stimuli.

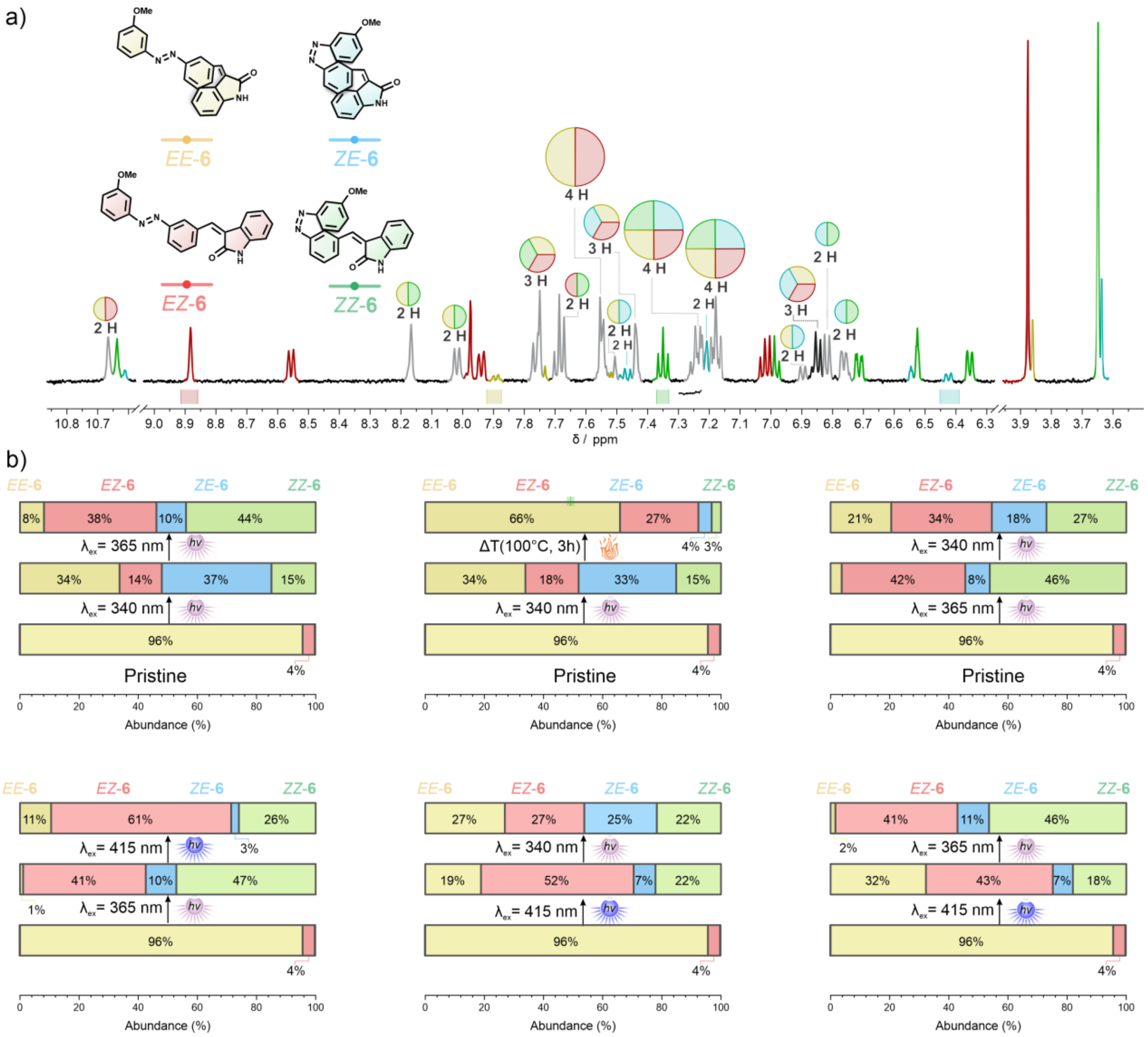

Figure S14: a) ^{1}H NMR ($(CD_3)_2SO$, 25 °C) spectra of the different light-generated isomers of **6**. The different signals were assigned by fitting top the formula f(t) = A[***EE*-6**] + B[***ZE*-6**] + C[***EZ*-6**] + D[***ZZ*-6**] and are shown color-coded respectively. Overlapping signals are shown in grey with an associated pie chart indicating the best-fitted combination of profiles. **b)** Isomeric distribution after difference sequences of *ex-situ* irradiation. Each chart starts with the pristine sample containing mainly ***EE*-6**. The following distributions reflect the changes after the indicated stimuli.

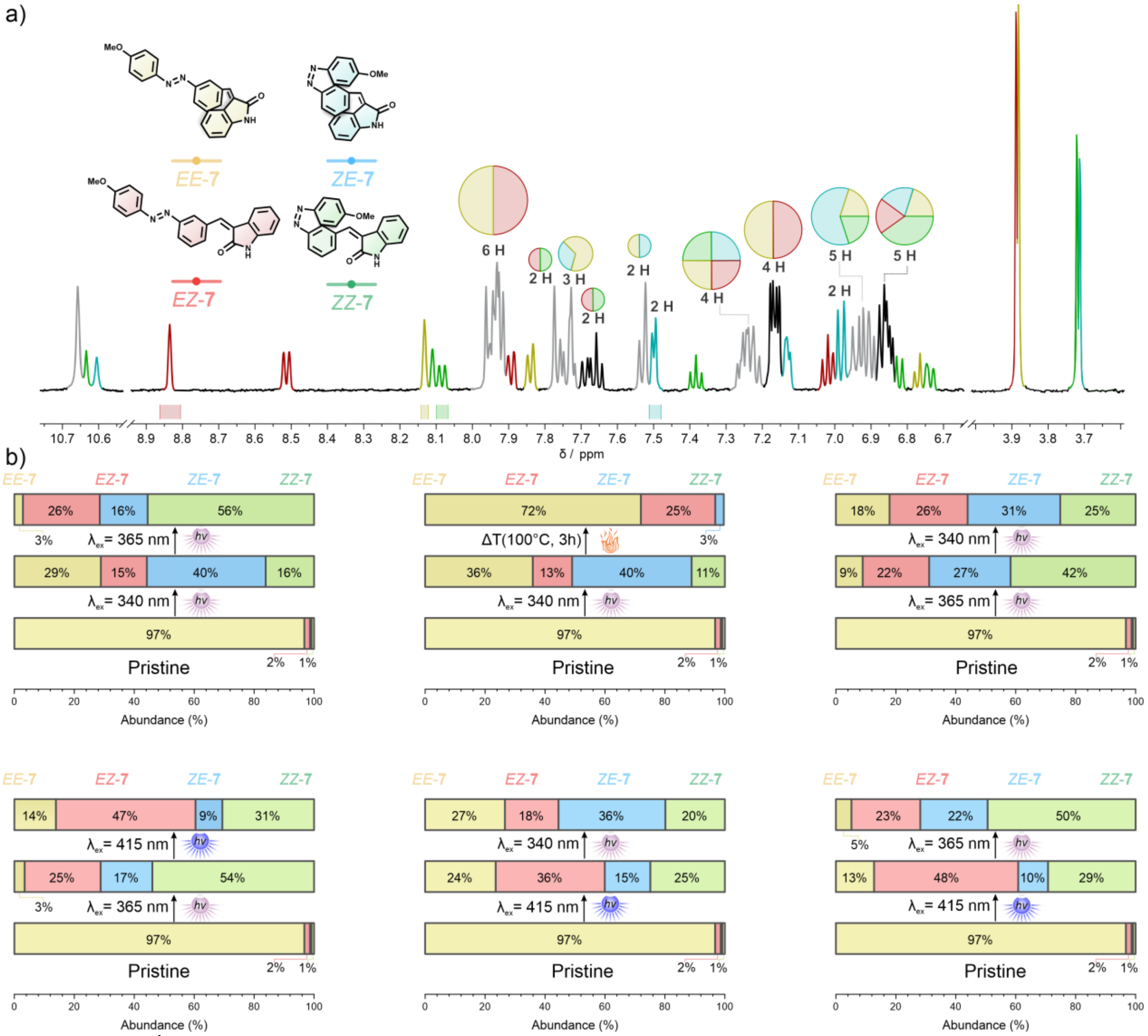

Figure S15: a) ^{1}H NMR ($(CD_3)_2SO$, 25 °C) spectra of the different light-generated isomers of **7**. The different signals were assigned by fitting top the formula f(t) = A[***EE*-7**] + B[***ZE*-7**] + C[***EZ*-7**] + D[***ZZ*-7**] and are shown color-coded respectively. Overlapping signals are shown in grey with an associated pie chart indicating the best-fitted combination of profiles. **b)** Isomeric distribution after difference sequences of *ex-situ* irradiation. Each chart starts with the pristine sample containing mainly ***EE*-7**. The following distributions reflect the changes after the indicated stimuli.

UV-Vis Spectroscopy

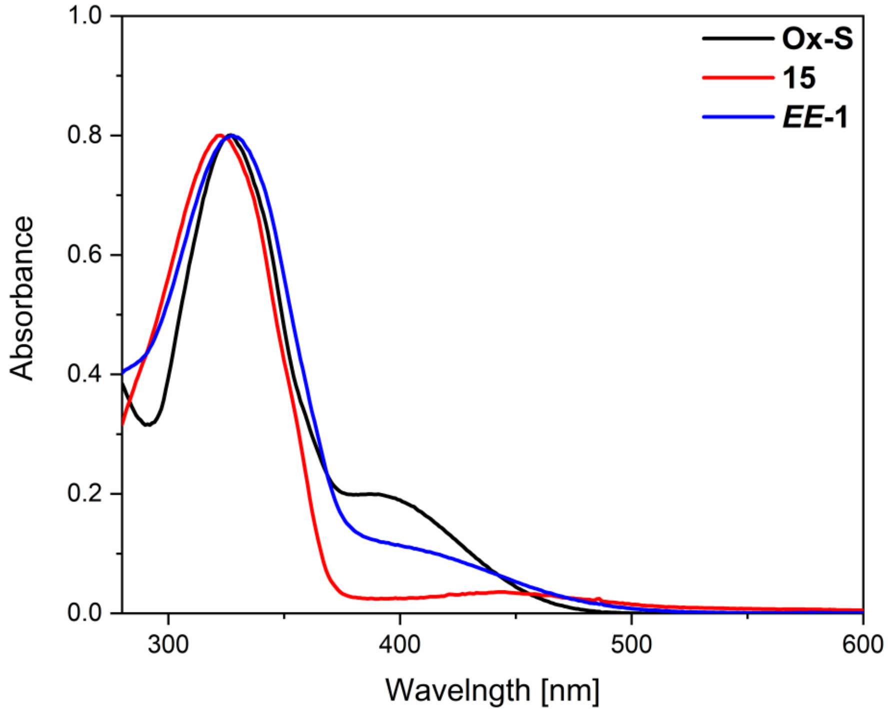

Figure S16: Comparison of normalized UV-Vis spectra of the oxindole switch (black), azobenzene (**15**) (red) and ***EE*-1** (blue) in DMSO at 20 ºC.

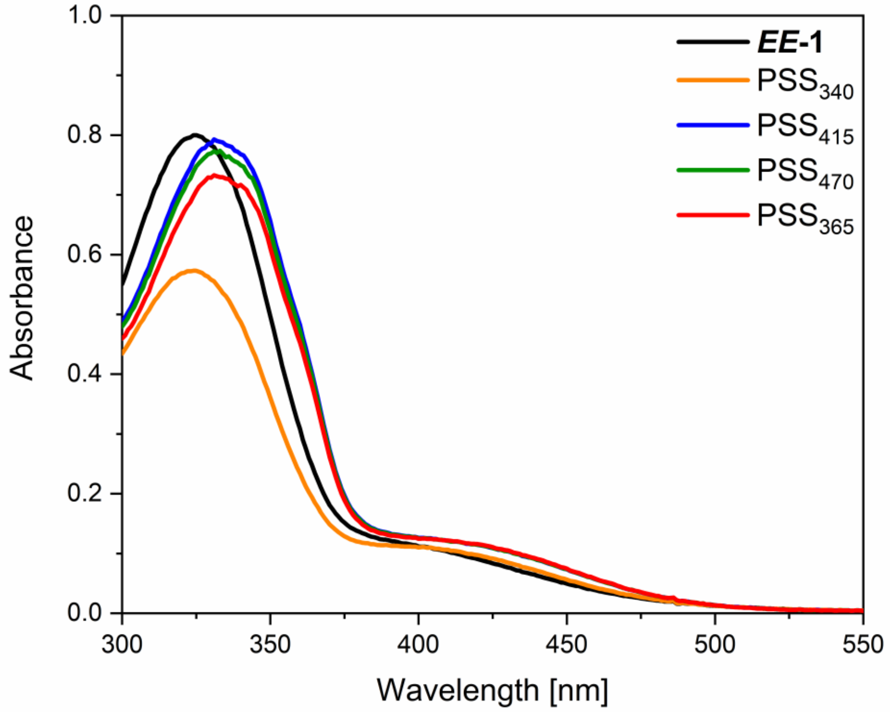

Figure S17: Normalized UV-Vis spectrum of ***EE*-1** in DMSO at 20 °C upon sequential irradiation with λ_{irr} = 340, 415, 470 and 365 nm to the respective PSSs.

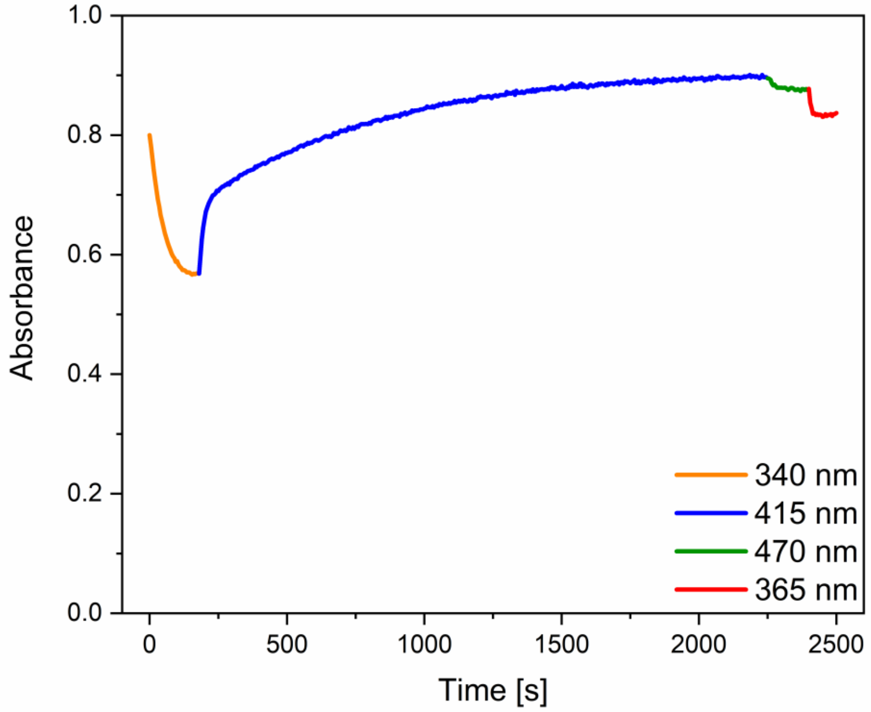

Figure S18: Change in absorbance at 340 nm (normalized) during the sequential irradiation of ***EE*-1** with a 340, 415, 470 and 365 nm LED at 20 ºC in DMSO.

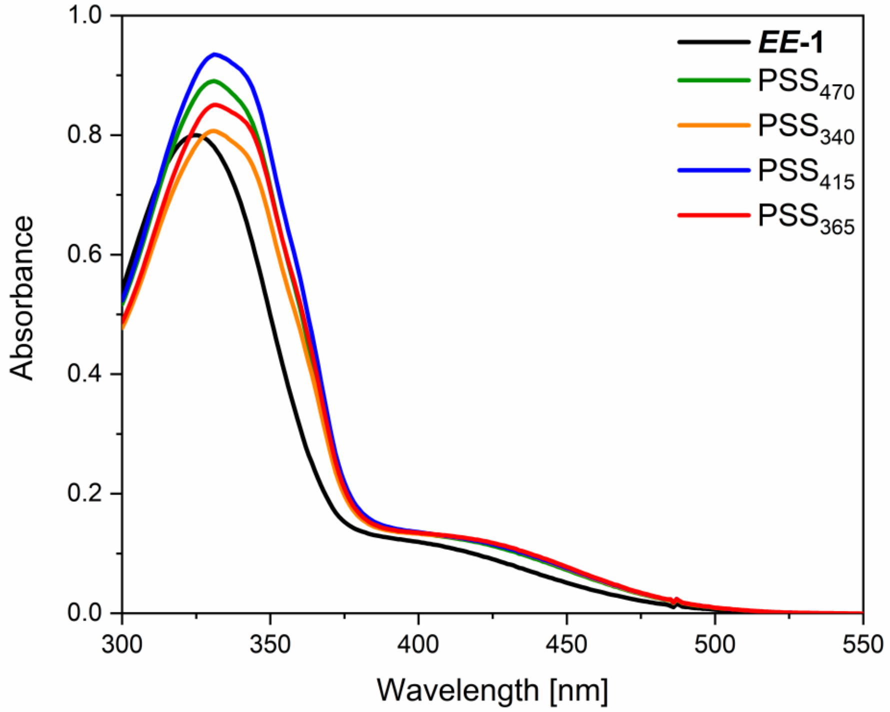

Figure S19: Normalized UV-Vis spectrum of ***EE*-1** in DMSO at 20 °C upon sequential irradiation with λ_{irr} = 470, 340, 415 and 365 nm to the respective PSSs.

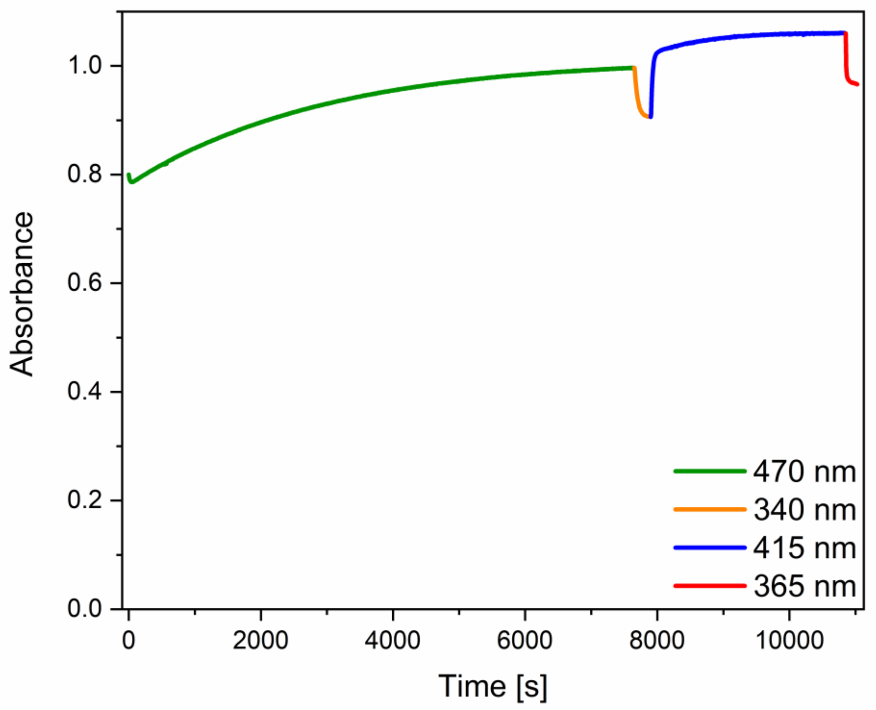

Figure S20: Change in absorbance at 340 nm (normalized) during the sequential irradiation of ***EE*-1** with a 470, 340, 415 and 365 nm LED at 20 ºC in DMSO.

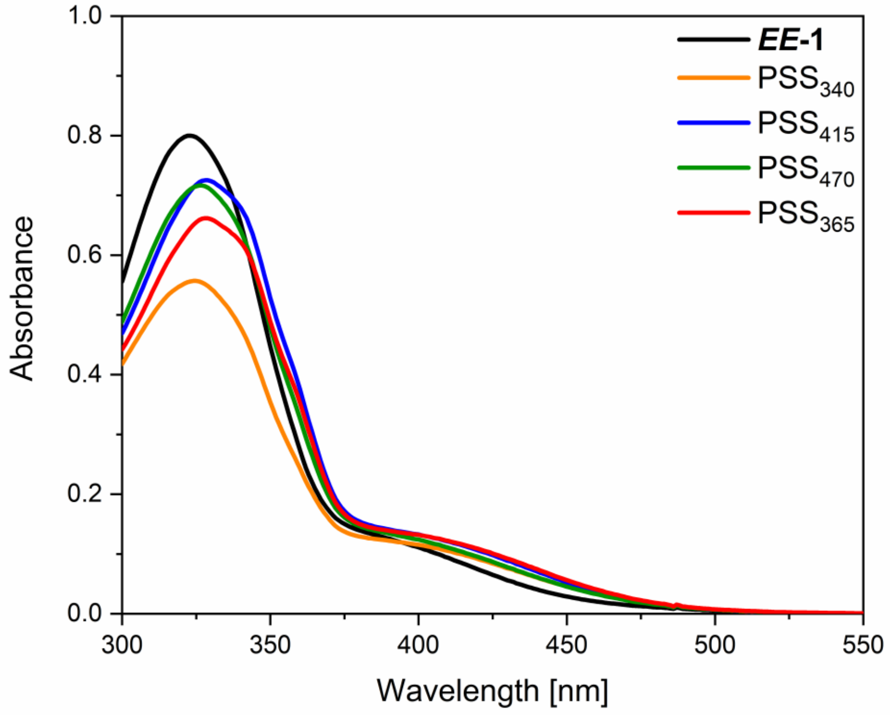

Figure S21: Normalized UV-Vis spectrum of ***EE*-1** in benzene at 20 °C upon sequential irradiation with λ_{irr} = 340, 415, 470 and 365 nm to the respective PSSs.

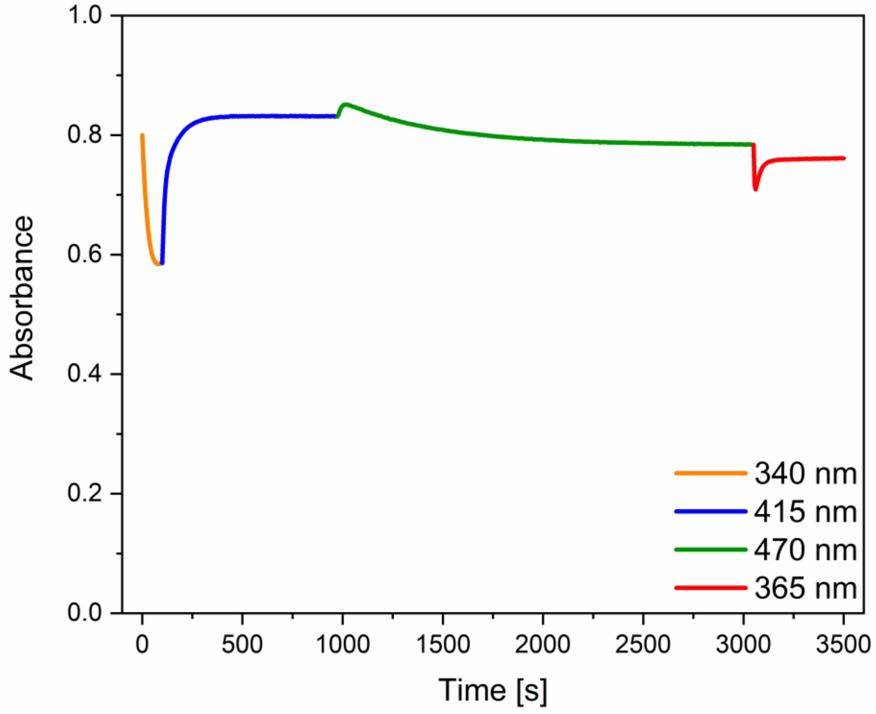

Figure S22: Change in absorbance at 340 nm (normalized) during the sequential irradiation of ***EE*-1** with a 340, 415, 470 and 365 nm LED at 20 ºC in benzene.

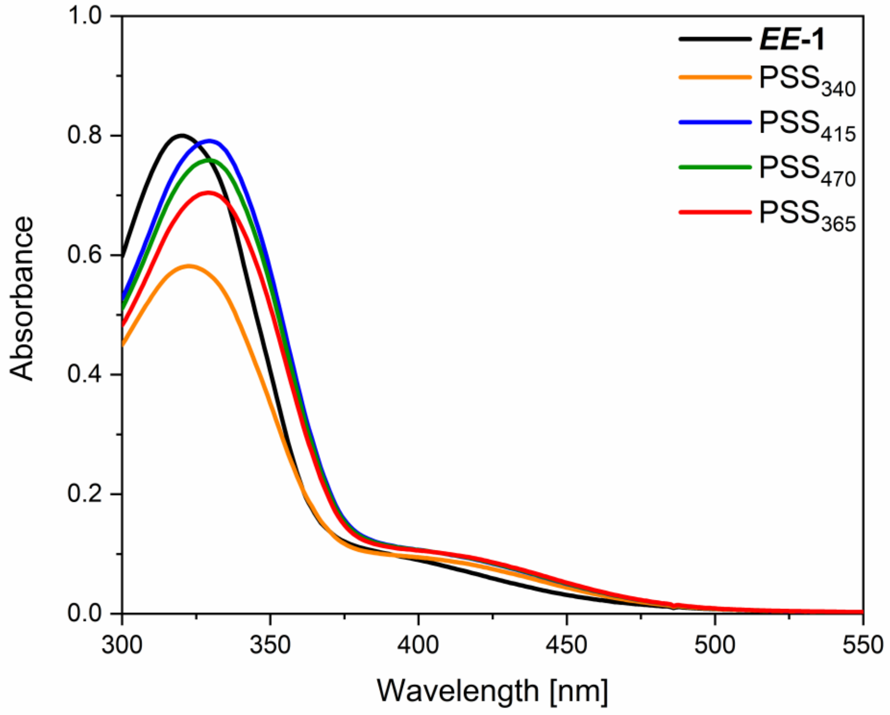

Figure S23: Normalized UV-Vis spectrum of ***EE*-1** in MeOH at 20 °C upon sequential irradiation with λ_{irr} = 340, 415, 470 and 365 nm to the respective PSSs.

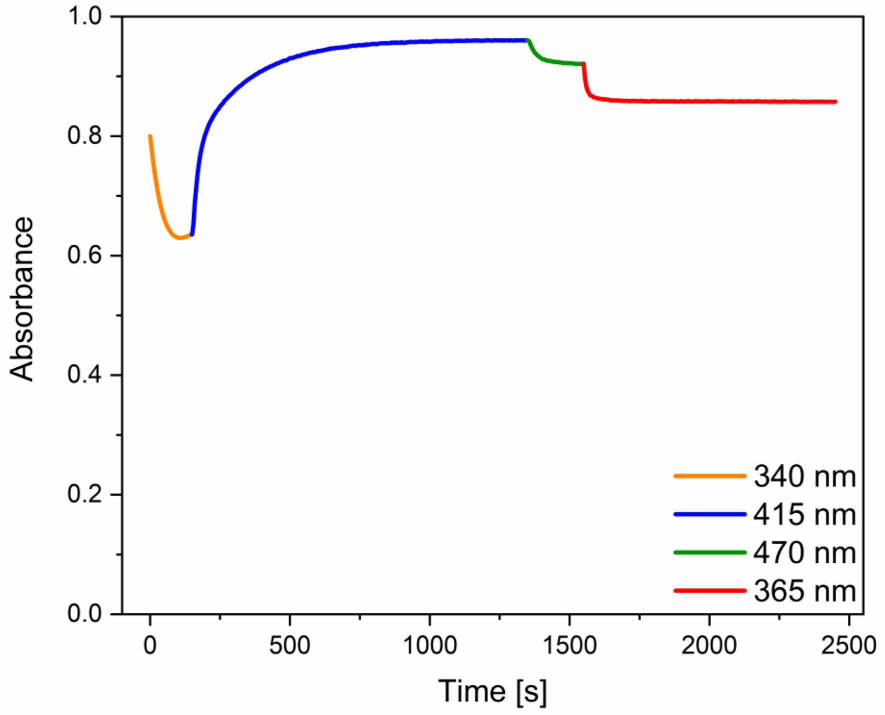

Figure S24: Change in absorbance at 340 nm (normalized) during the sequential irradiation of ***EE*-1** with a 340, 415, 470 and 365 nm LED at 20 ºC in MeOH.

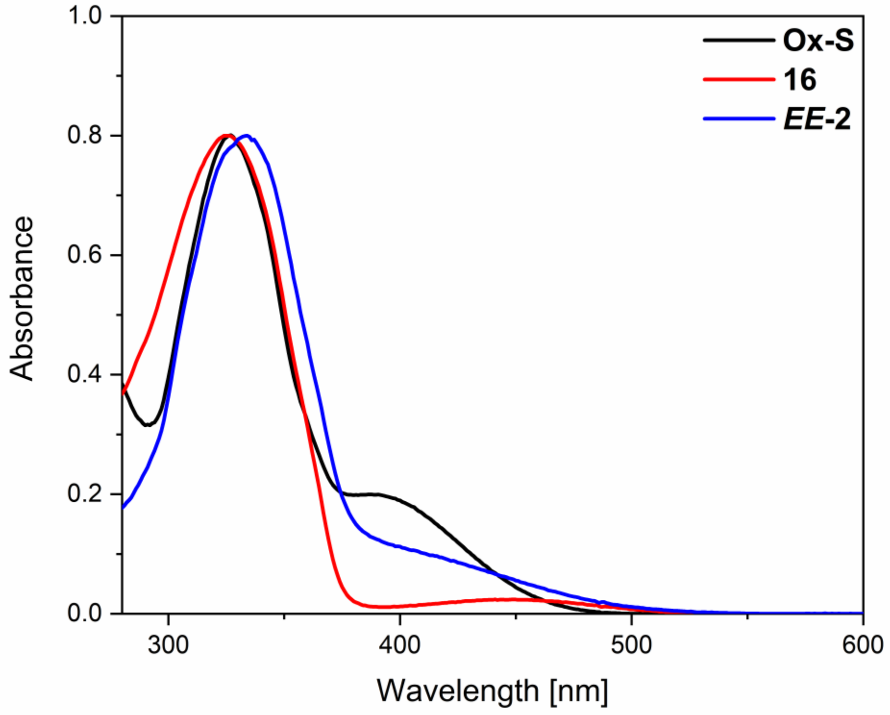

Figure S25: Comparison of normalized UV-Vis spectra of the oxindole switch (black), azobenzene (**16**) (red) and ***EE*-2** (blue) in DMSO at 20 ºC.

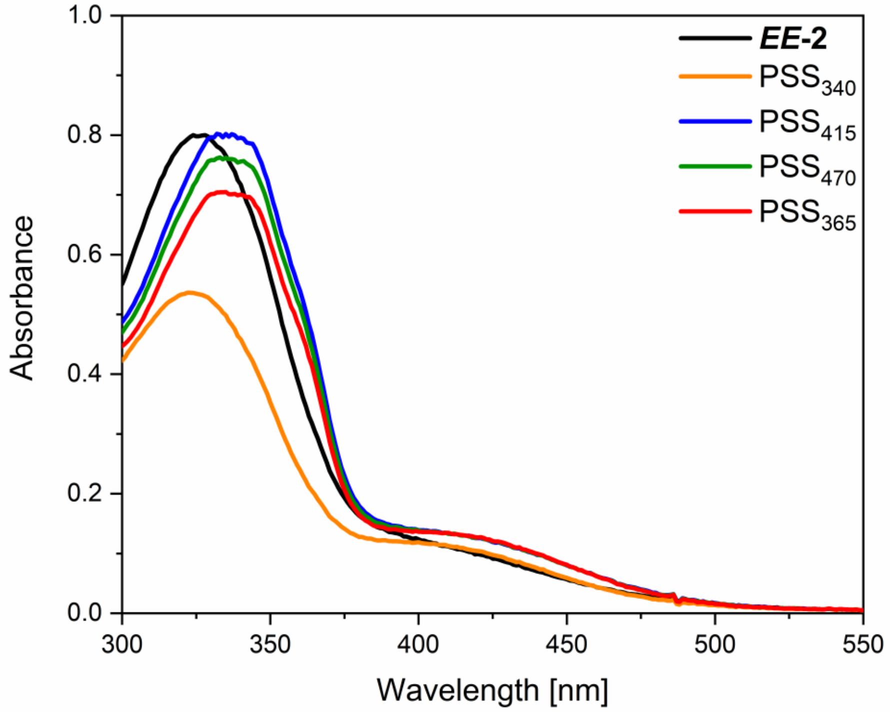

Figure S26: Normalized UV-Vis spectrum of ***EE*-2** in DMSO at 20 °C upon sequential irradiation with λ_{irr} = 340, 415, 470 and 365 nm to the respective PSSs.

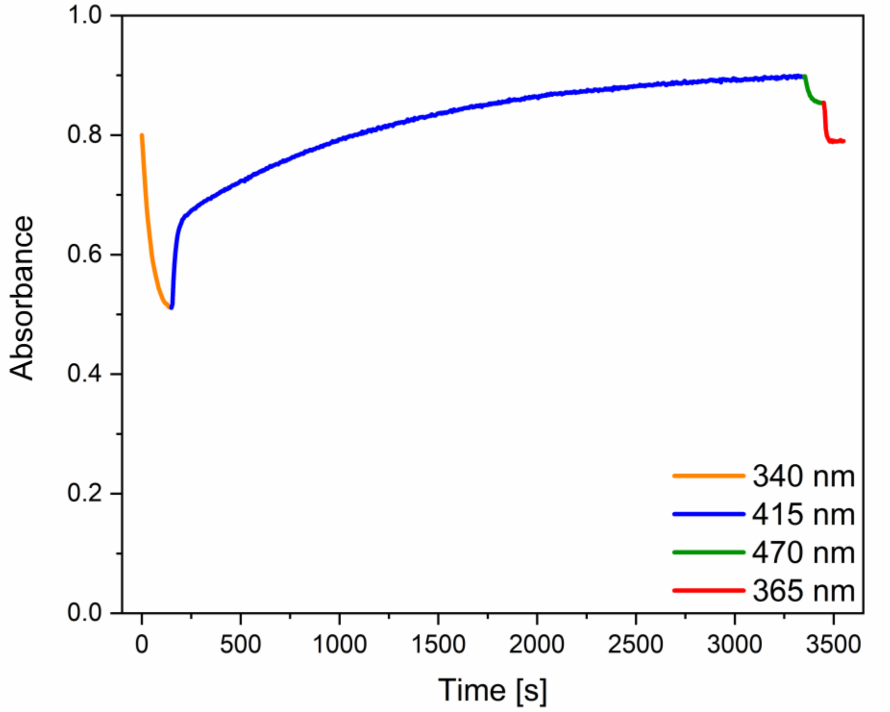

Figure S27: Change in absorbance at 340 nm (normalized) during the sequential irradiation of ***EE*-2** with a 340, 415, 470 and 365 nm LED at 20 ºC in DMSO.

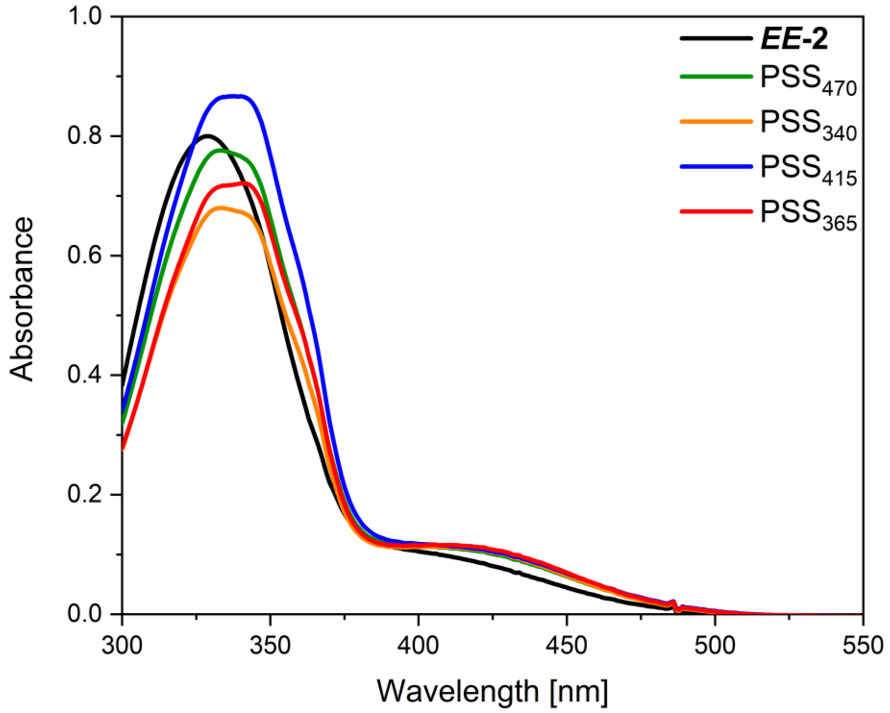

Figure S28: Normalized UV-Vis spectrum of ***EE*-2** in DMSO at 20 °C upon sequential irradiation with λ_{irr} = 470, 340, 415 and 365 nm to the respective PSSs.

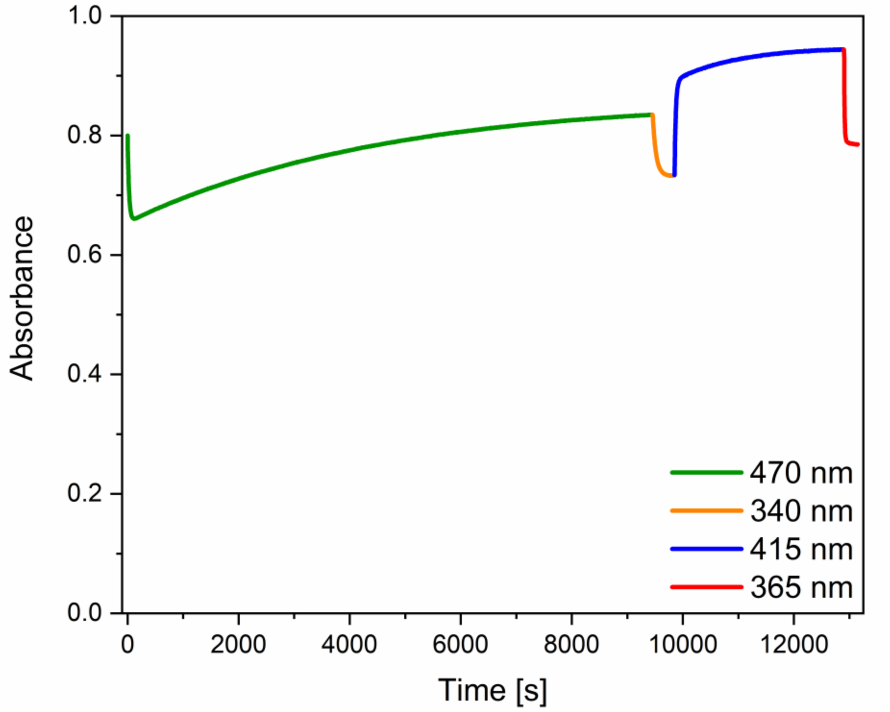

Figure S29: Change in absorbance at 340 nm (normalized) during the sequential irradiation of ***EE*-2** with a 470, 340, 415 and 365 nm LED at 20 ºC in DMSO.

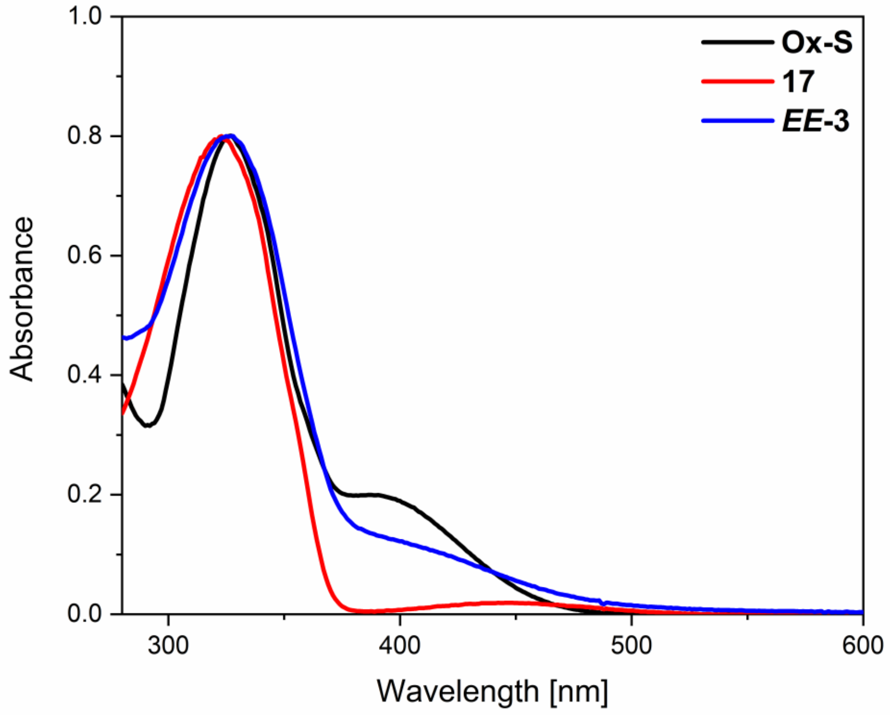

Figure S30: Comparison of normalized UV-Vis spectra of the oxindole switch (black), azobenzene (**17**) (red) and ***EE*-3** (blue) in DMSO at 20 ºC.

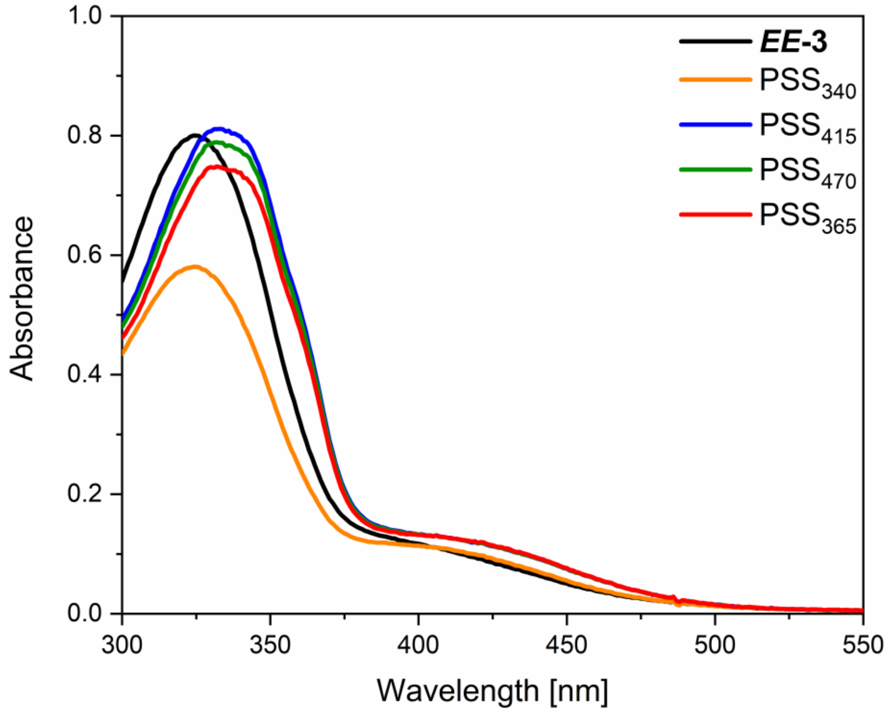

Figure S31: Normalized UV-Vis spectrum of ***EE*-3** in DMSO at 20 °C upon sequential irradiation with λ_{irr} = 340, 415, 470 and 365 nm to the respective PSSs.

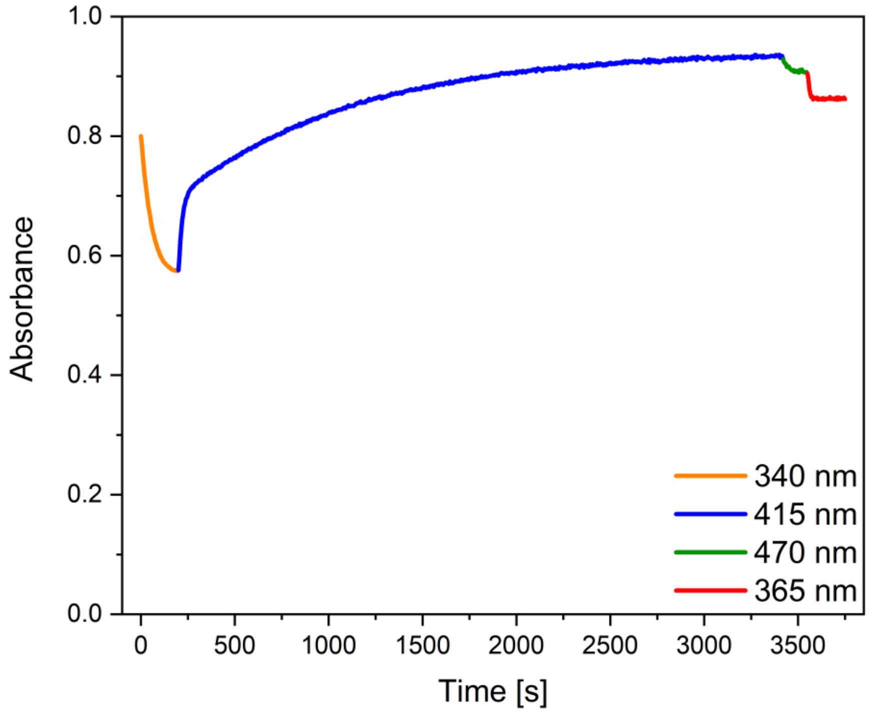

Figure S32: Change in absorbance at 340 nm (normalized) during the sequential irradiation of ***EE*-3** with a 340, 415, 470 and 365 nm LED at 20 ºC in DMSO.

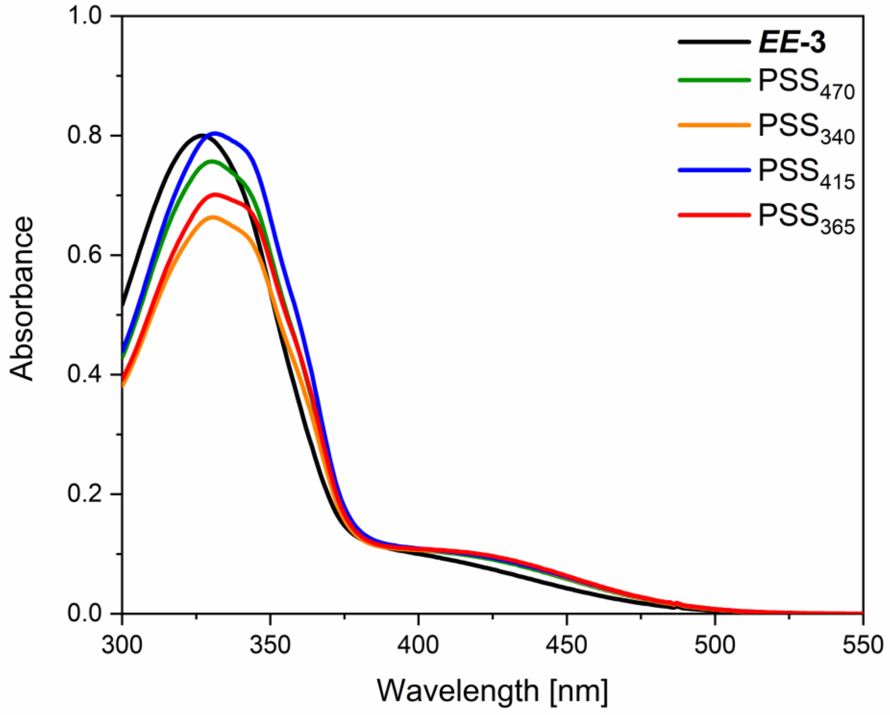

Figure S33: Normalized UV-Vis spectrum of ***EE*-3** in DMSO at 20 °C upon sequential irradiation with λ_{irr} = 470, 340, 415 and 365 nm to the respective PSSs.

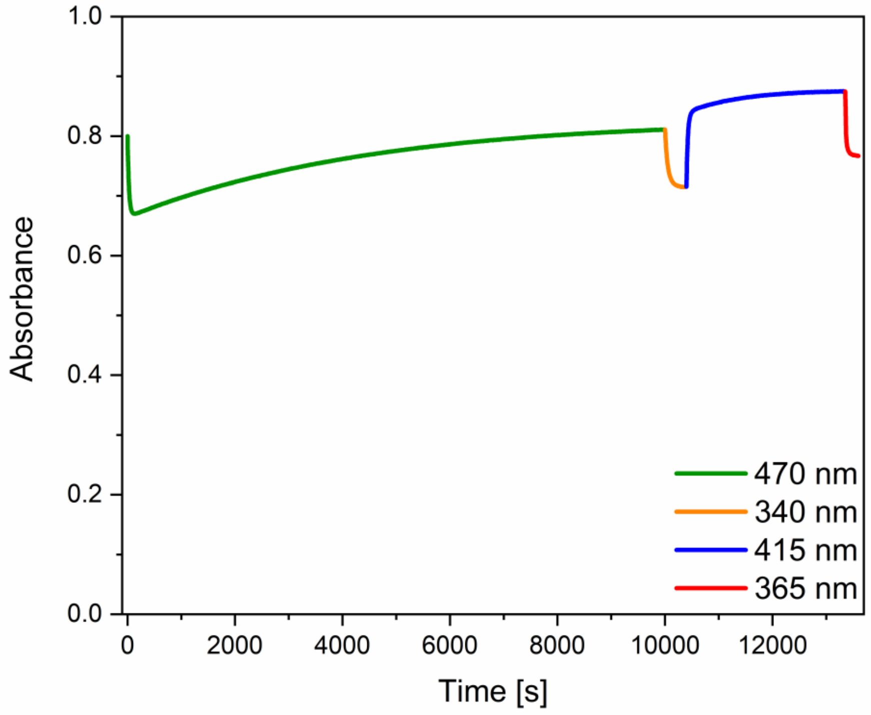

Figure S34: Change in absorbance at 340 nm (normalized) during the sequential irradiation of ***EE*-3** with a 470, 340, 415 and 365 nm LED at 20 ºC in DMSO.

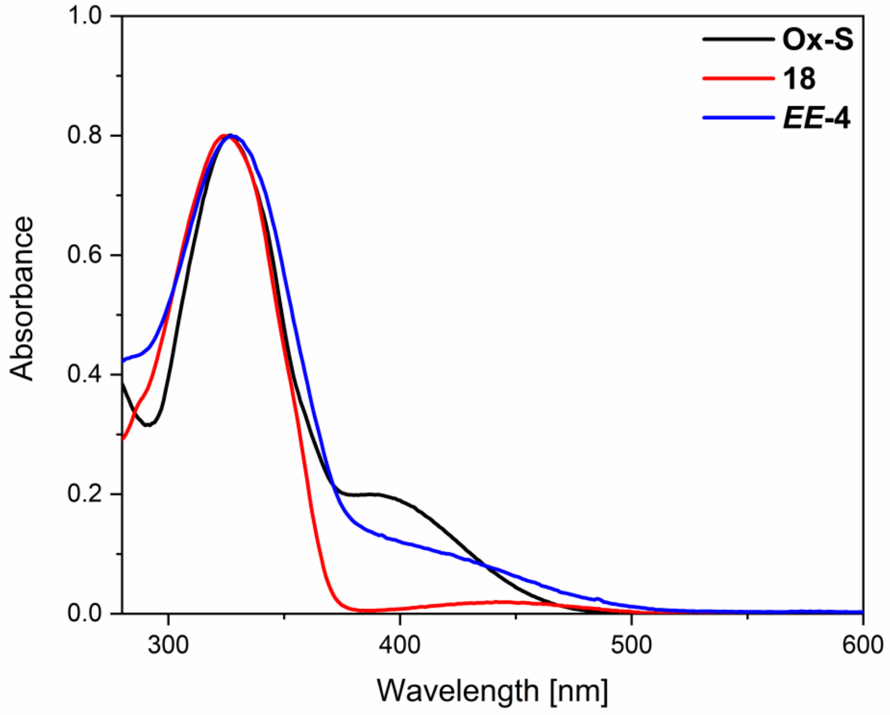

Figure S35: Comparison of normalized UV-Vis spectra of the oxindole switch (black), azobenzene (**18**) (red) and ***EE*-4** (blue) in DMSO at 20 ºC.

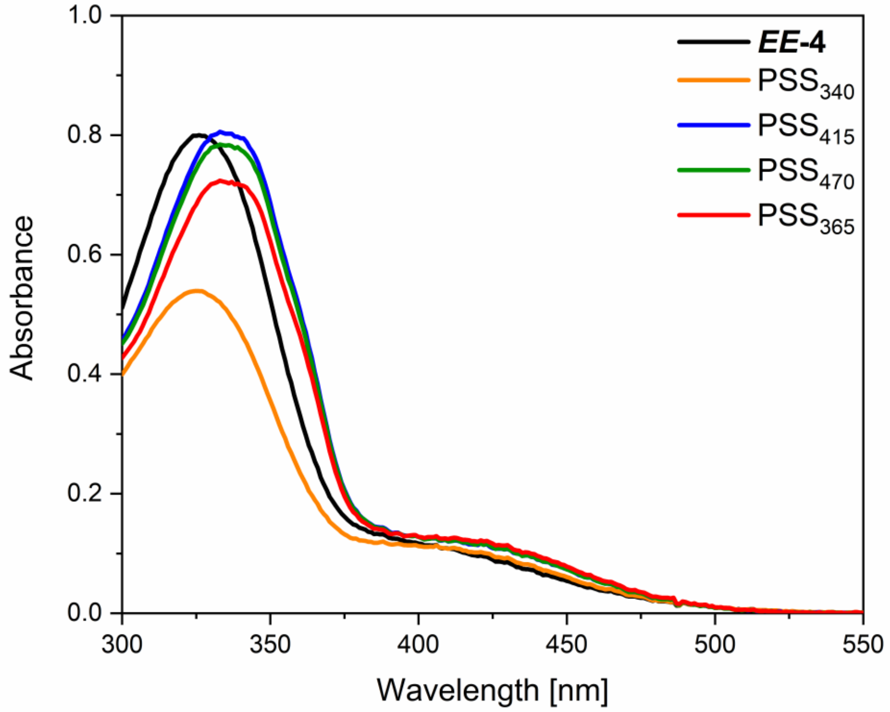

Figure S36: Normalized UV-Vis spectrum of ***EE*-4** in DMSO at 20 °C upon sequential irradiation with λ_{irr} = 340, 415, 470 and 365 nm to the respective PSSs.

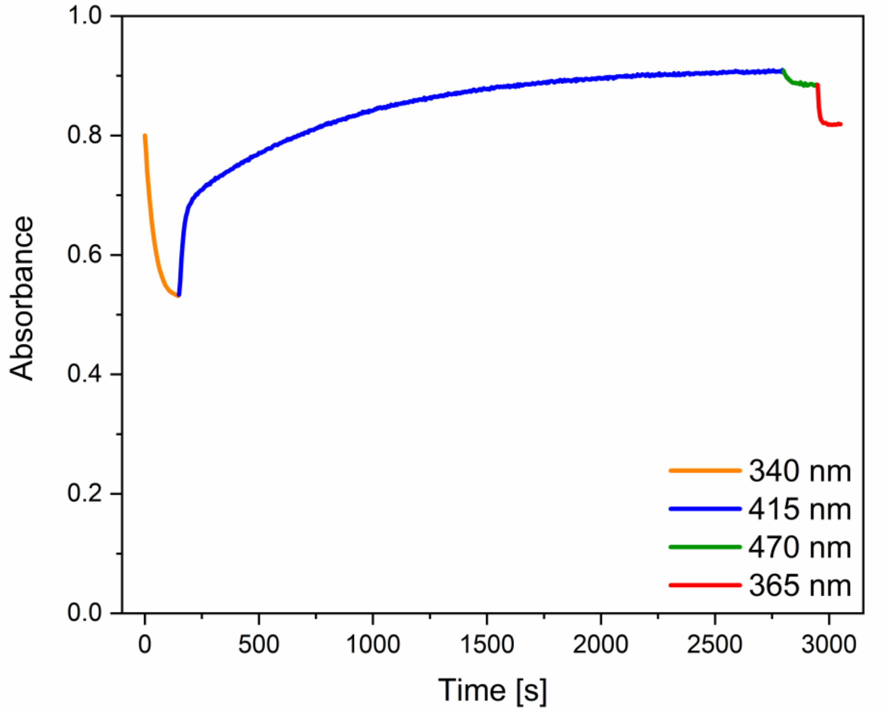

Figure S37: Change in absorbance at 340 nm (normalized) during the sequential irradiation of ***EE*-4** with a 340, 415, 470 and 365 nm LED at 20 ºC in DMSO.

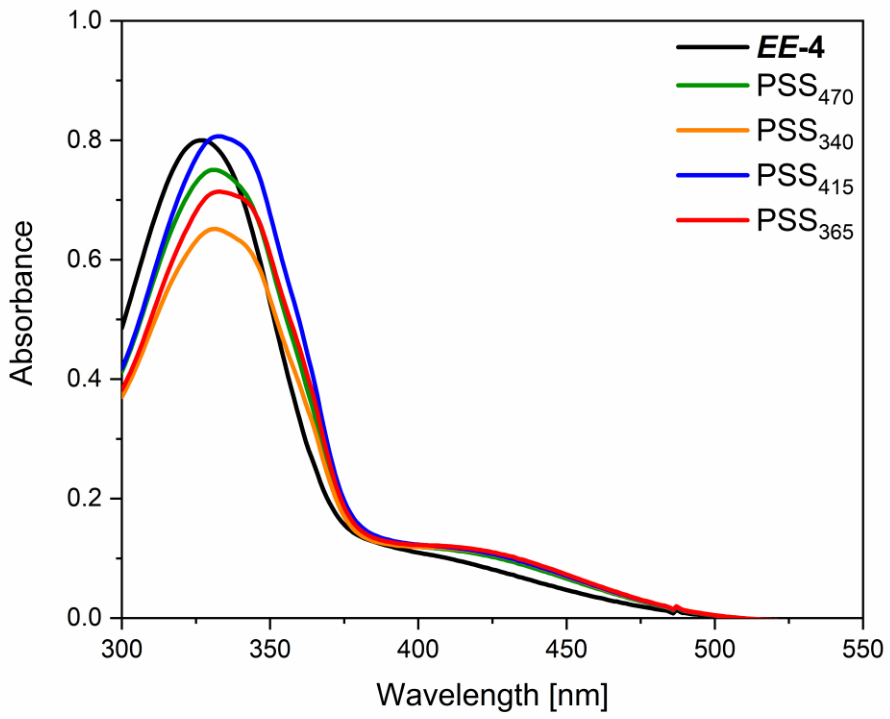

Figure S38: Normalized UV-Vis spectrum of ***EE*-4** in DMSO at 20 °C upon sequential irradiation with λ_{irr} = 470, 340, 415 and 365 nm to the respective PSSs.

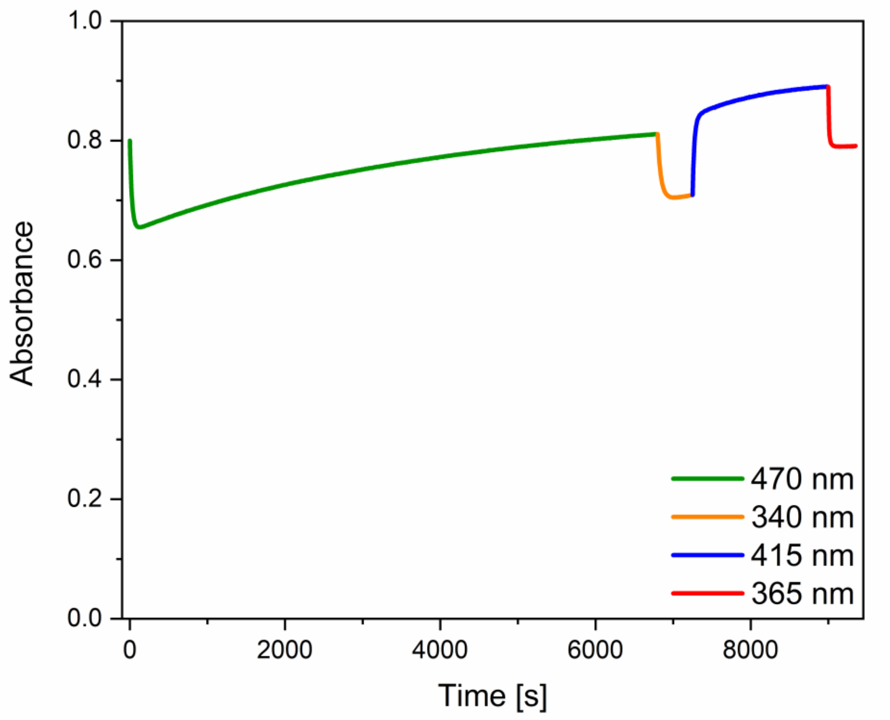

Figure S39: Change in absorbance at 340 nm (normalized) during the sequential irradiation of ***EE*-4** with a 470, 340, 415 and 365 nm LED at 20 ºC in DMSO.

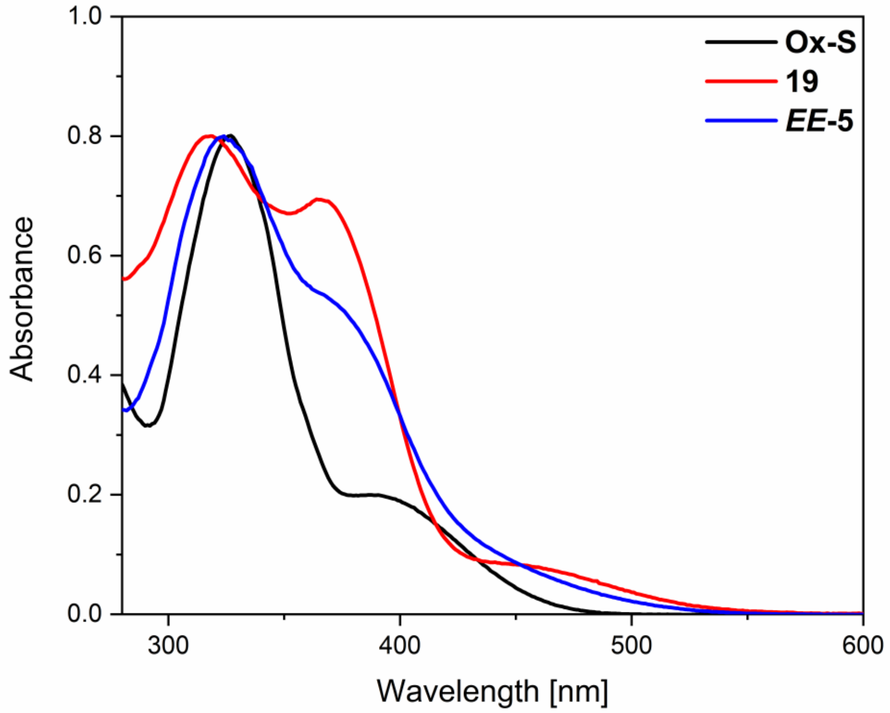

Figure S40: Comparison of normalized UV-Vis spectra of the oxindole switch (black), azobenzene (**19**) (red) and ***EE*-5** (blue) in DMSO at 20 ºC.

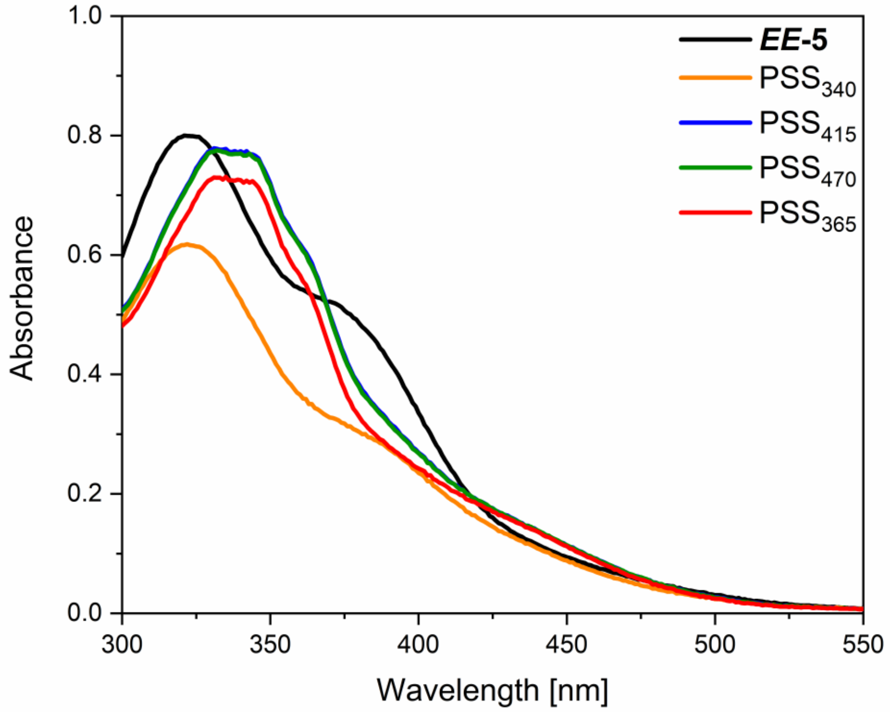

Figure S41: Normalized UV-Vis spectrum of ***EE*-5** in DMSO at 20 °C upon sequential irradiation with λ_{irr} = 340, 415, 470 and 365 nm to the respective PSSs.

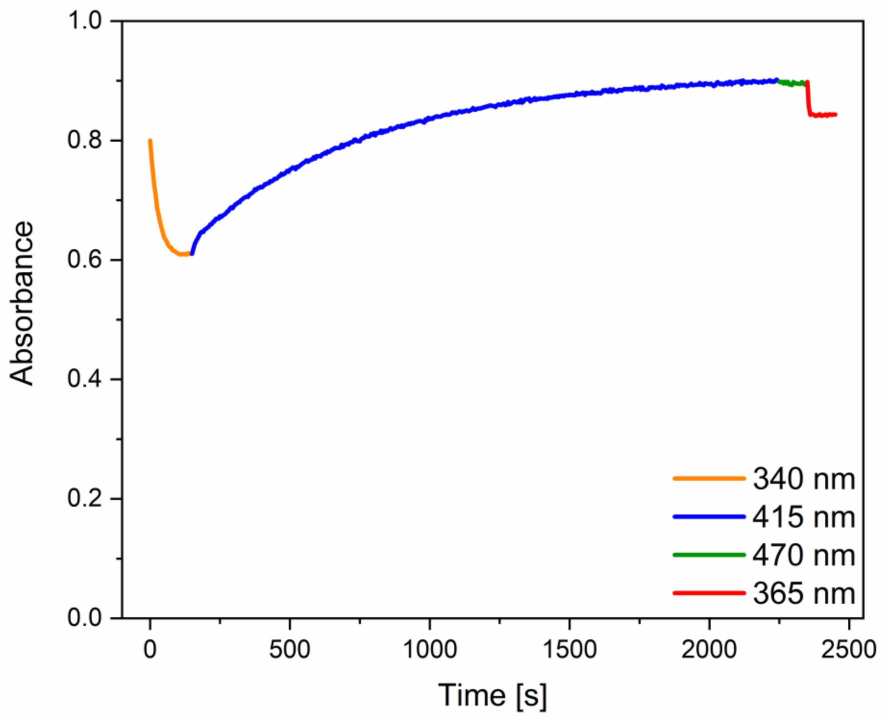

Figure S42: Change in absorbance at 340 nm (normalized) during the sequential irradiation of ***EE*-5** with a 340, 415, 470 and 365 nm LED at 20 ºC in DMSO.

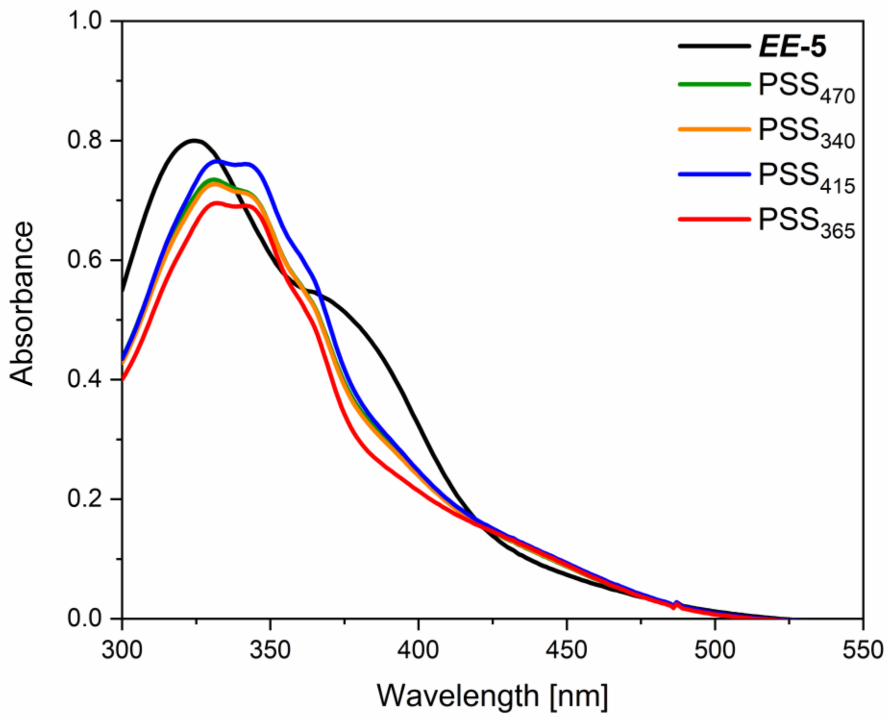

Figure S43: Normalized UV-Vis spectrum of ***EE*-5** in DMSO at 20 °C upon sequential irradiation with λ_{irr} = 470, 340, 415 and 365 nm to the respective PSSs.

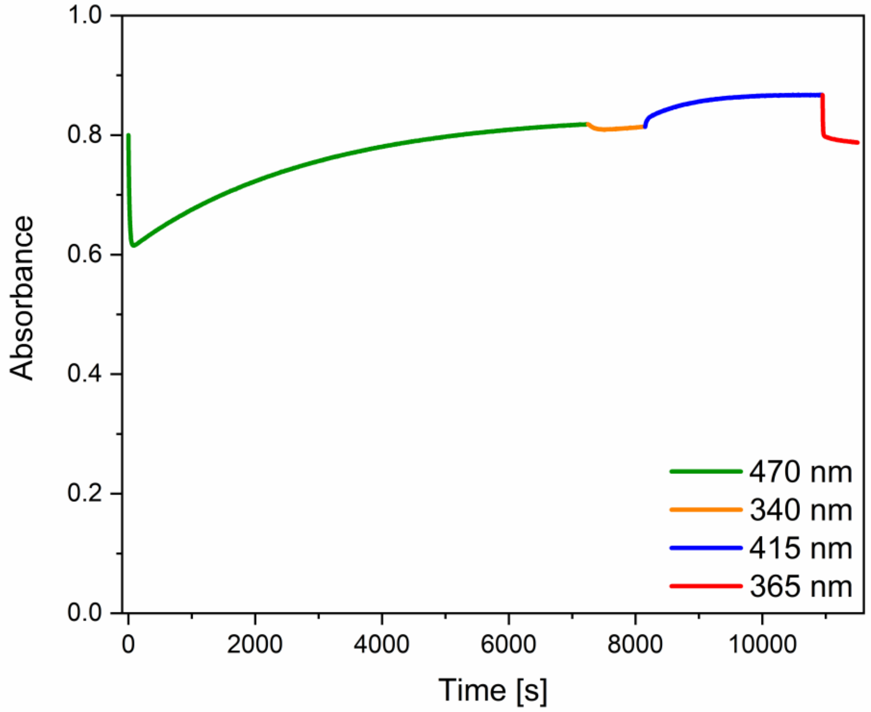

Figure S44: Change in absorbance at 340 nm (normalized) during the sequential irradiation of ***EE*-5** with a 470, 340, 415 and 365 nm LED at 20 ºC in DMSO.

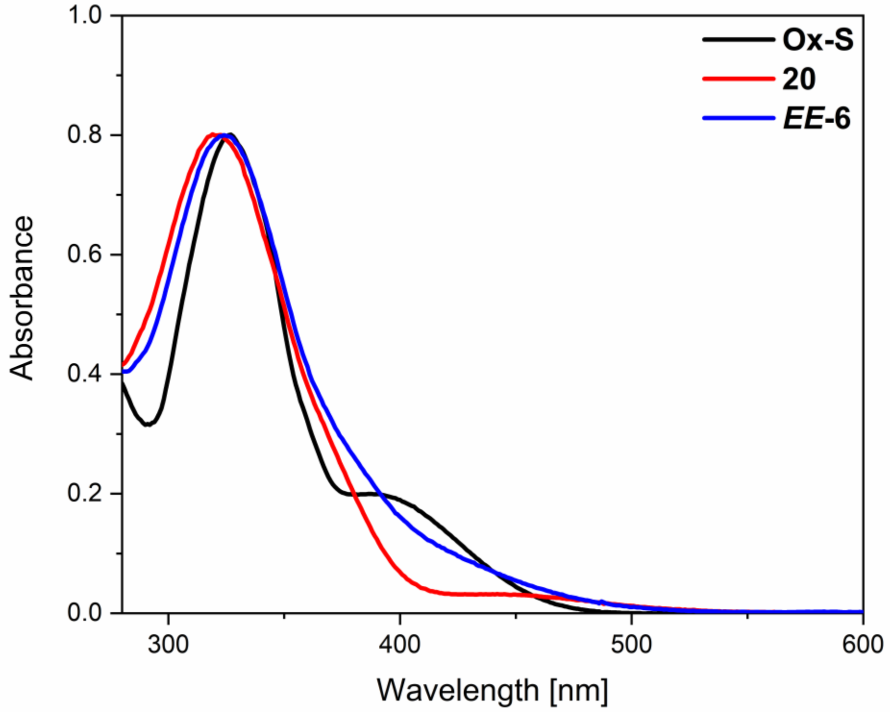

Figure S45: Comparison of normalized UV-Vis spectra of the oxindole switch (black), azobenzene (**20**) (red) and ***EE*-6** (blue) in DMSO at 20 ºC.

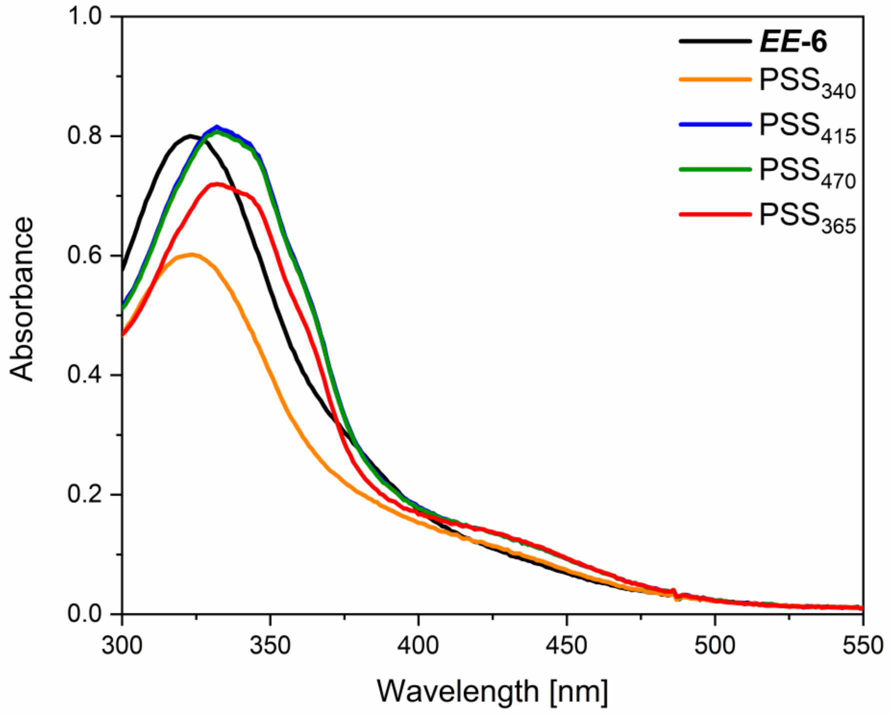

Figure S46: Normalized UV-Vis spectrum of ***EE*-6** in DMSO at 20 °C upon sequential irradiation with λ_{irr} = 340, 415, 470 and 365 nm to the respective PSSs.

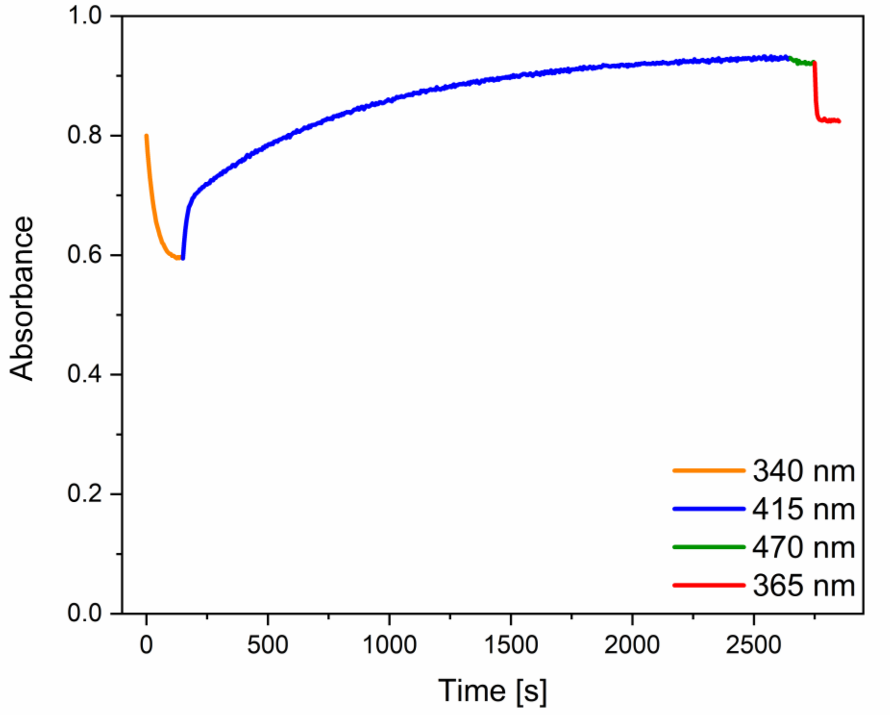

Figure S47: Change in absorbance at 340 nm (normalized) during the sequential irradiation of ***EE*-6** with a 340, 415, 470 and 365 nm LED at 20 ºC in DMSO.

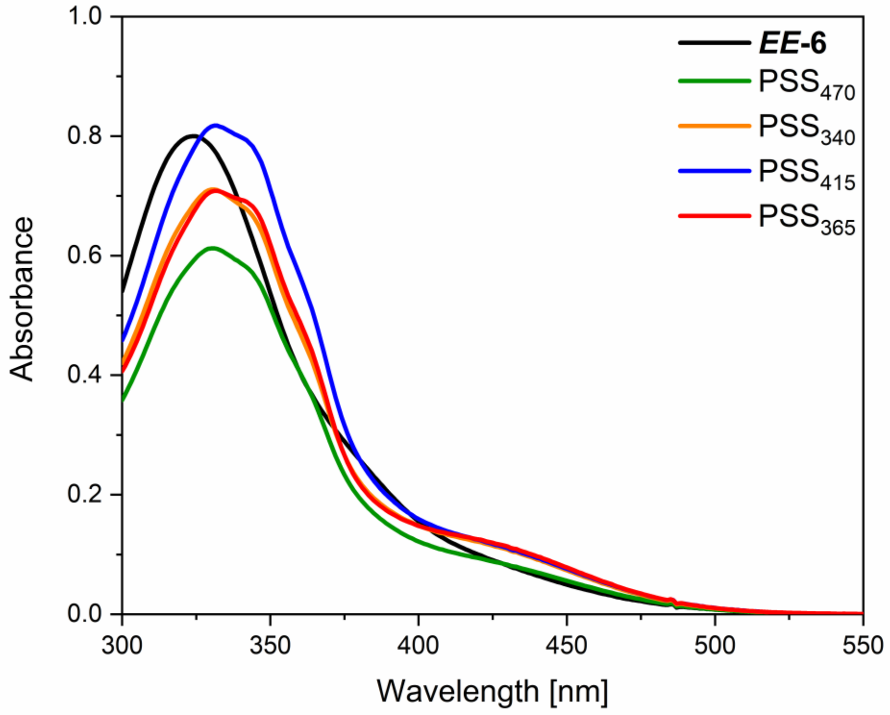

Figure S48: Normalized UV-Vis spectrum of ***EE*-6** in DMSO at 20 °C upon sequential irradiation with λ_{irr} = 470, 340, 415 and 365 nm to the respective PSSs.

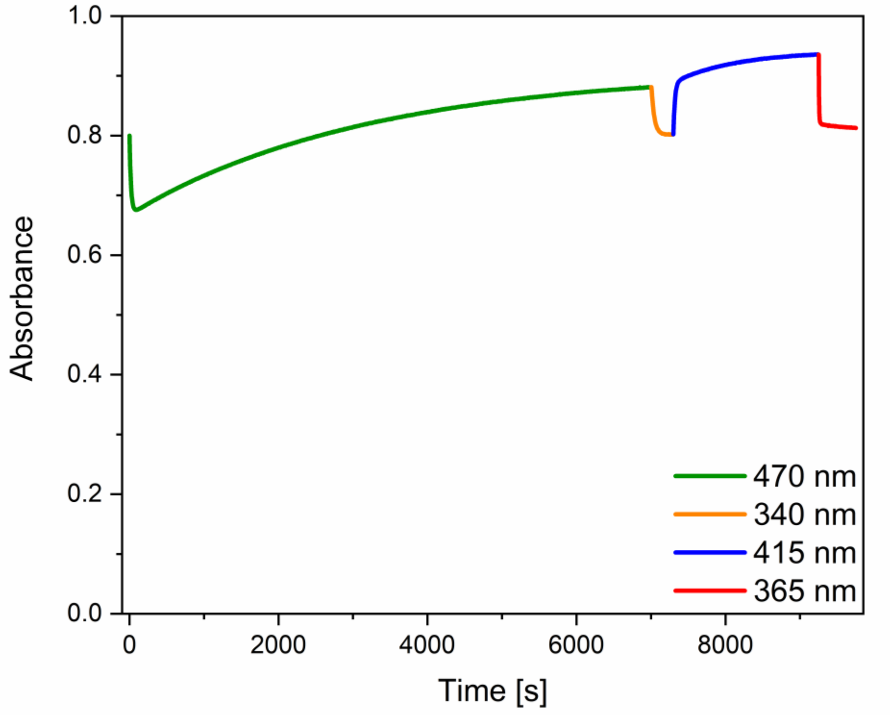

Figure S49: Change in absorbance at 340 nm (normalized) during the sequential irradiation of ***EE*-6** with a 470, 340, 415 and 365 nm LED at 20 ºC in DMSO.

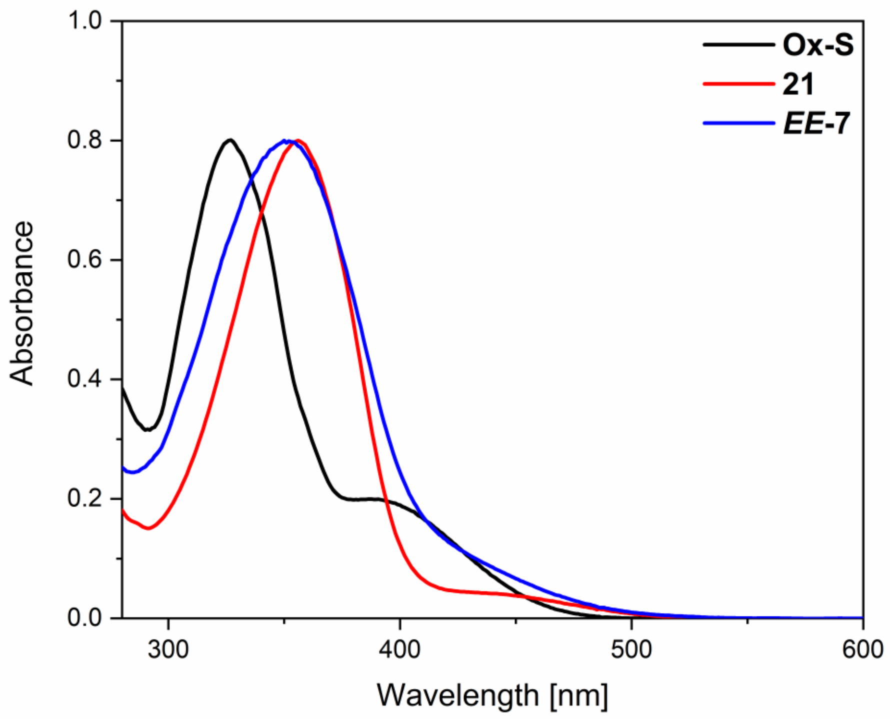

Figure S50: Comparison of normalized UV-Vis spectra of the oxindole switch (black), azobenzene (**21**) (red) and ***EE*-7** (blue) in DMSO at 20 ºC.

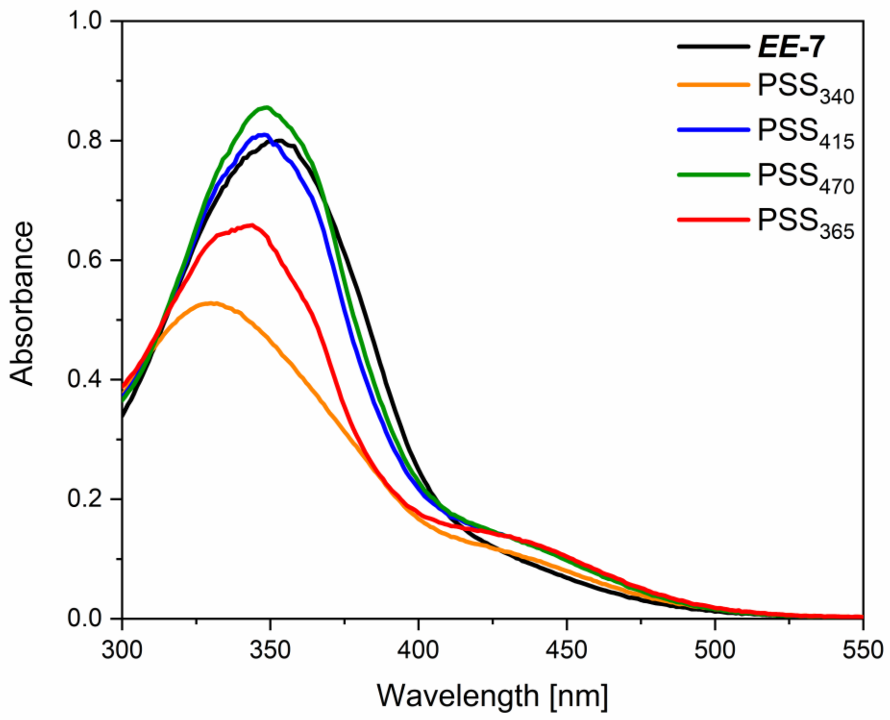

Figure S51: Normalized UV-Vis spectrum of ***EE*-7** in DMSO at 20 °C upon sequential irradiation with λ_{irr} = 340, 415, 470 and 365 nm to the respective PSSs.

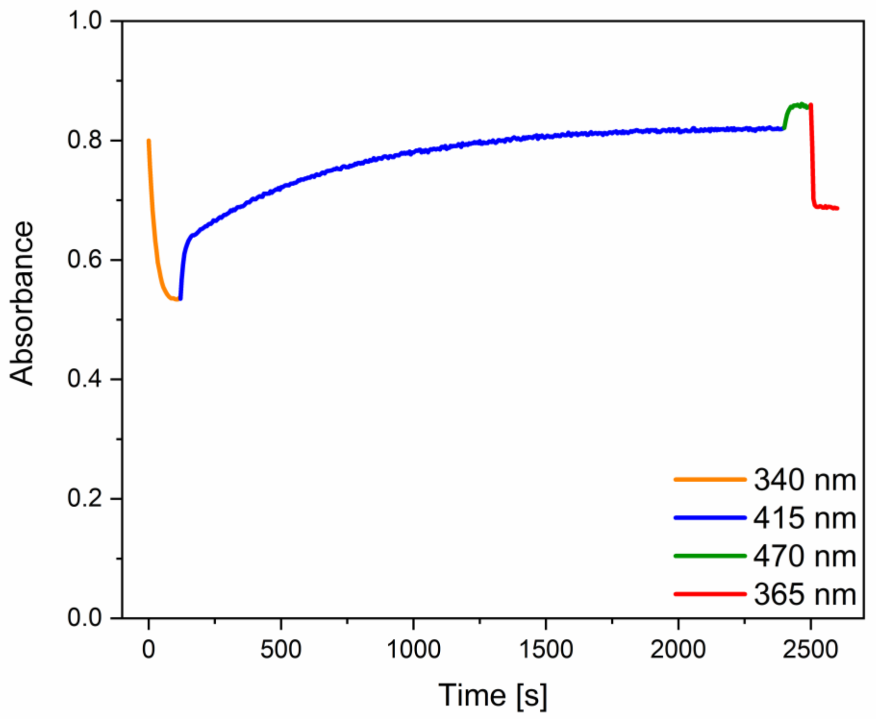

Figure S52: Change in absorbance at 340 nm (normalized) during the sequential irradiation of ***EE*-7** with a 340, 415, 470 and 365 nm LED at 20 ºC in DMSO.

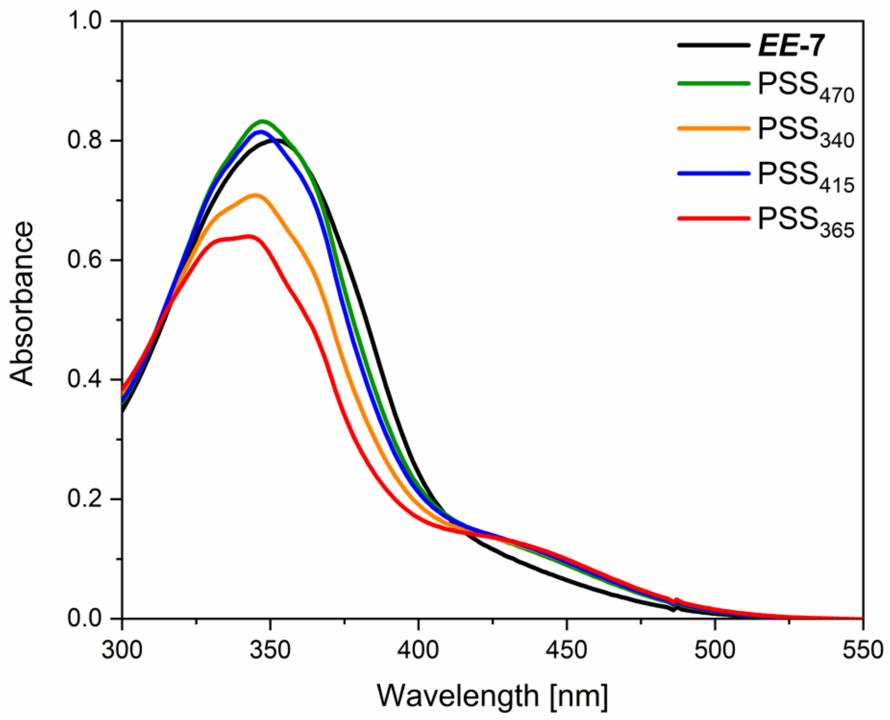

Figure S53: Normalized UV-Vis spectrum of ***EE*-7** in DMSO at 20 °C upon sequential irradiation with λ_{irr} = 470, 340, 415 and 365 nm to the respective PSSs.

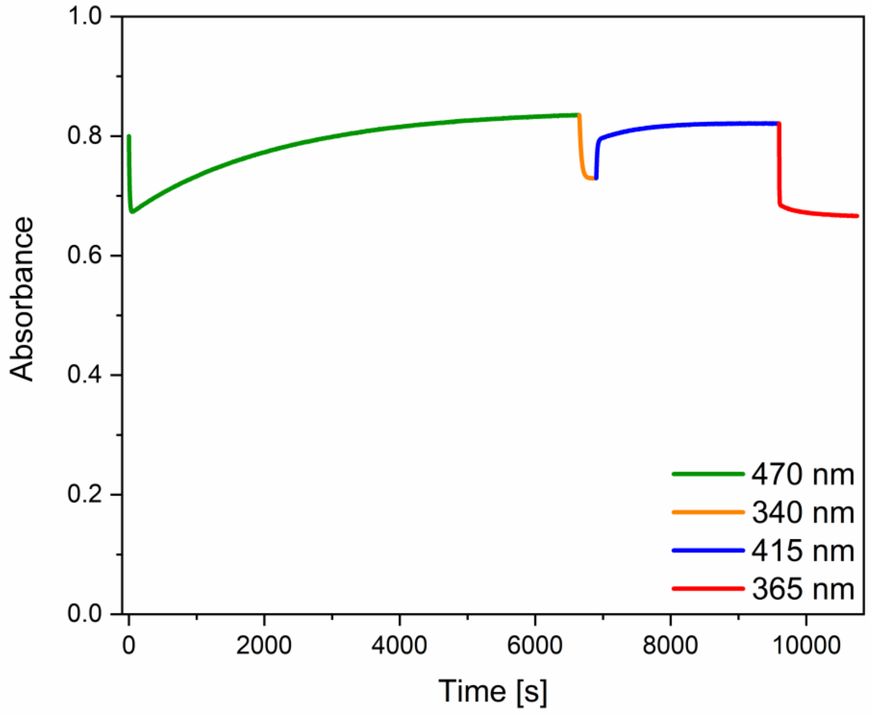

Figure S54: Change in absorbance at 340 nm (normalized) during the sequential irradiation of ***EE*-7** with a 470, 340, 415 and 365 nm LED at 20 ºC in DMSO.

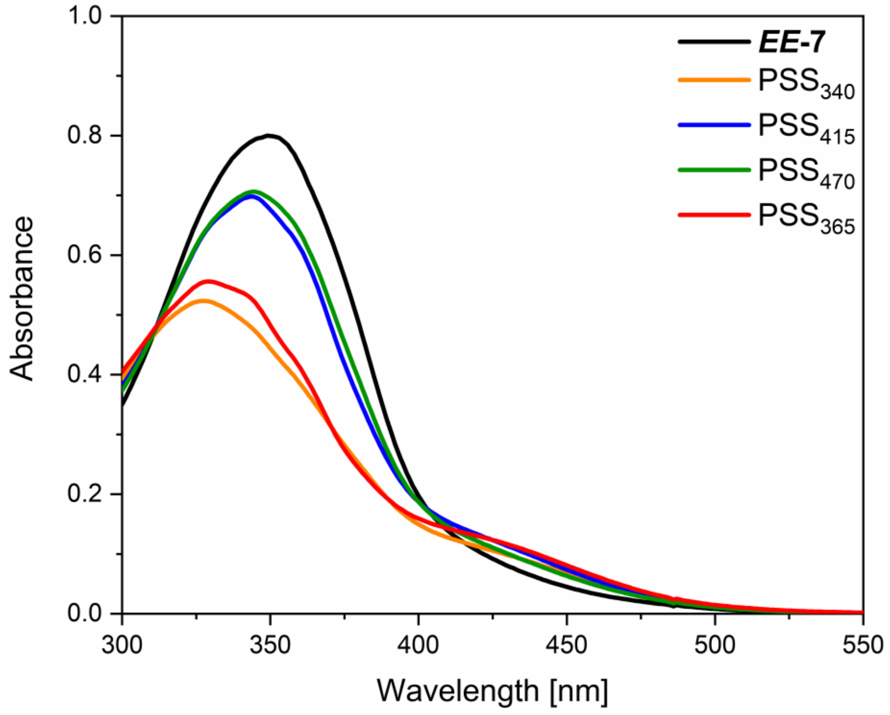

Figure S55: Normalized UV-Vis spectrum of ***EE*-7** in benzene at 20 °C upon sequential irradiation with λ_{irr} = 340, 415, 470 and 365 nm to the respective PSSs.

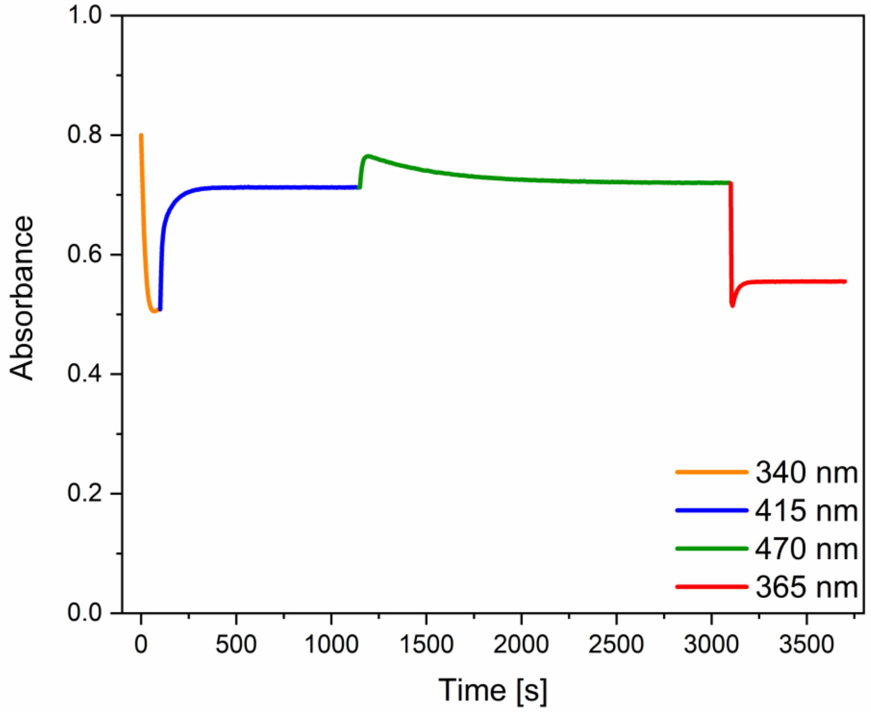

Figure S56: Change in absorbance at 340 nm (normalized) during the sequential irradiation of ***EE*-7** with a 340, 415, 470 and 365 nm LED at 20 ºC in benzene.

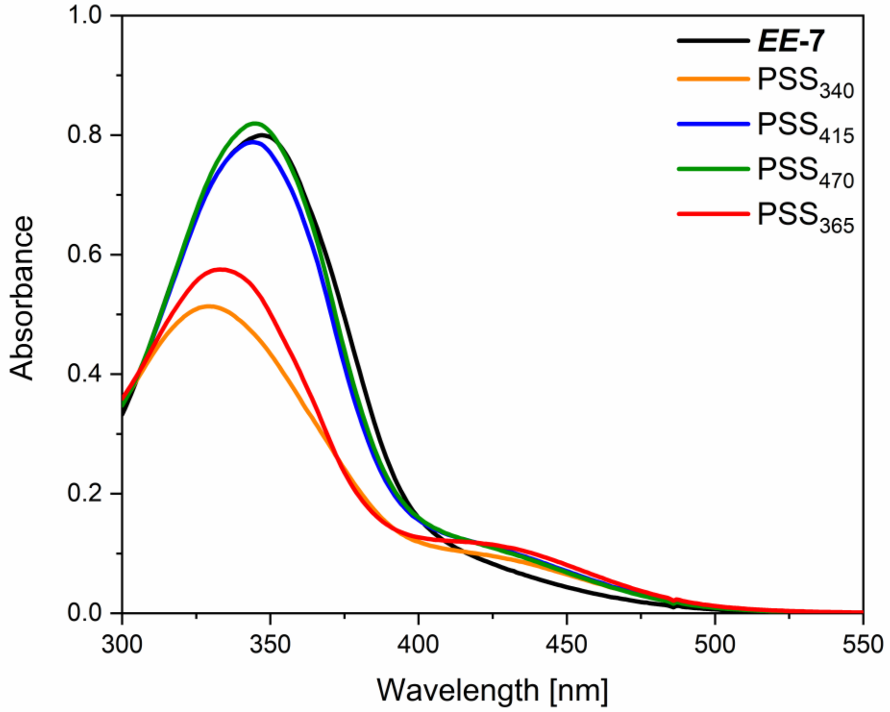

Figure S57: Normalized UV-Vis spectrum of ***EE*-7** in MeOH at 20 °C upon sequential irradiation with λ_{irr} = 340, 415, 470 and 365 nm to the respective PSSs.

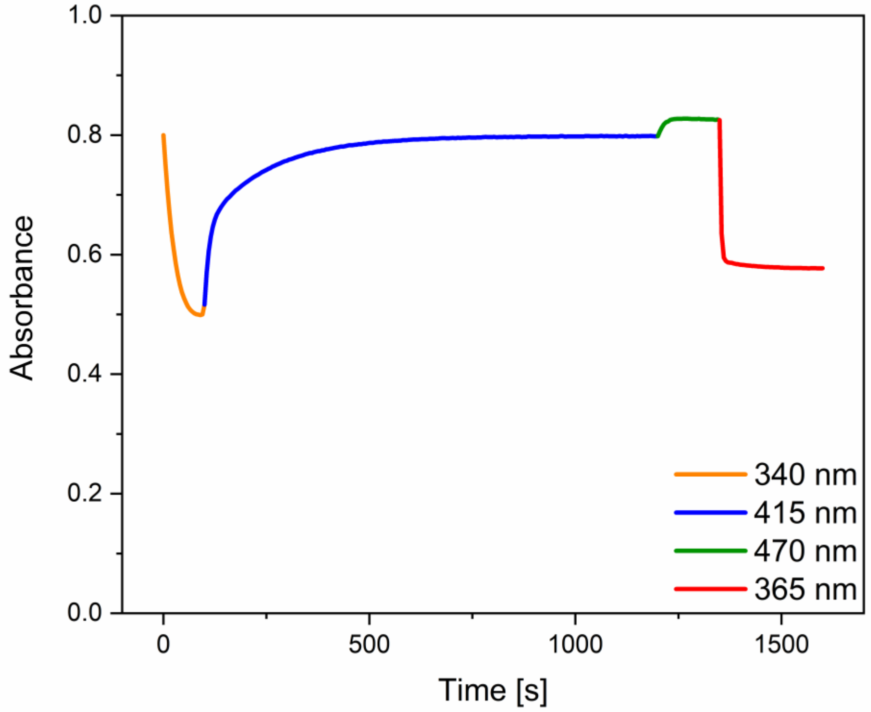

Figure S58: Change in absorbance at 340 nm (normalized) during the sequential irradiation of ***EE*-7** with a 340, 415, 470 and 365 nm LED at 20 ºC in MeOH.

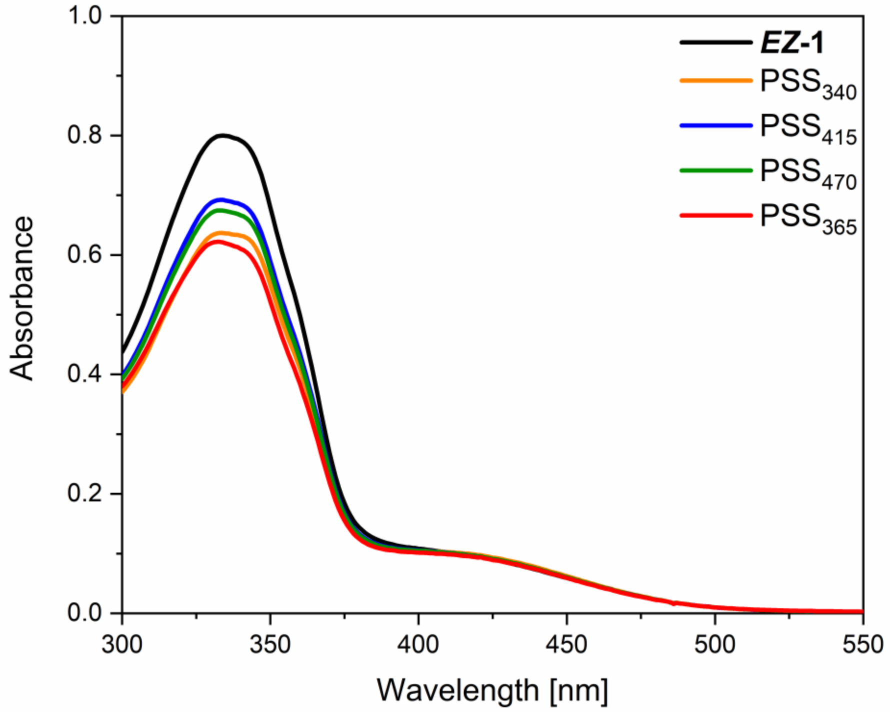

Figure S59: Normalized UV-Vis spectrum of ***EZ*-1** in DMSO at 20 °C upon sequential irradiation with λ_{irr} = 340, 415, 470 and 365 nm to the respective PSSs.

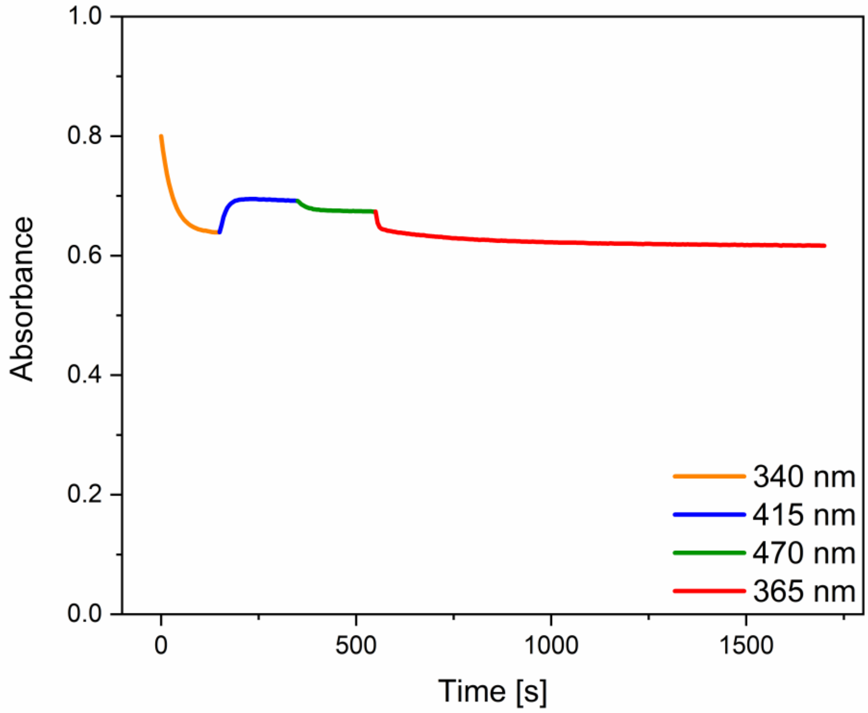

Figure S60: Change in absorbance at 340 nm (normalized) during the sequential irradiation of ***EZ*-1** with a 340, 415, 470 and 365 nm LED at 20 ºC in DMSO.

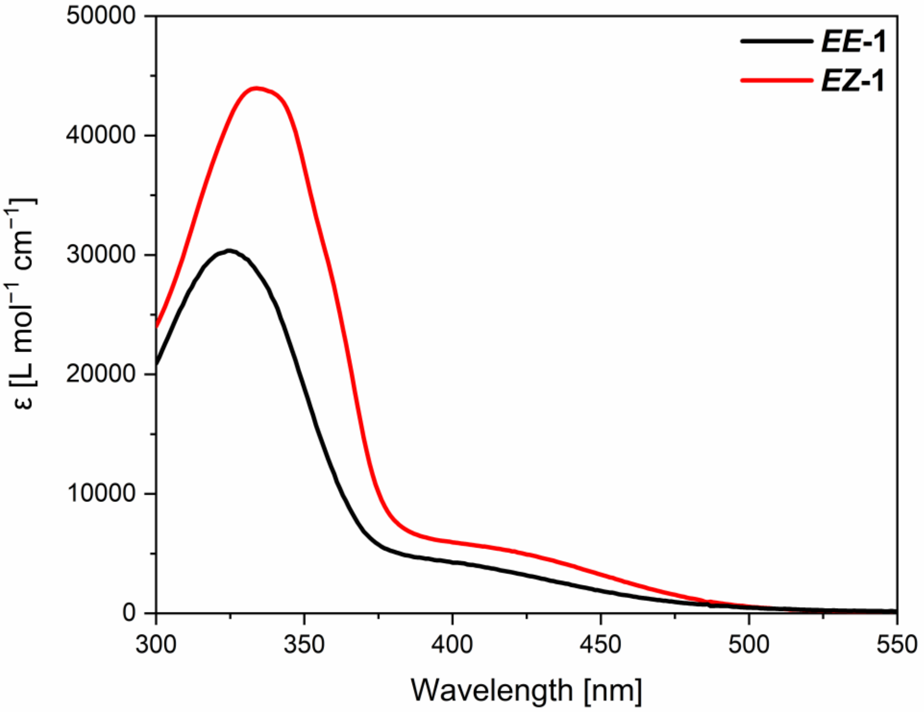

Figure S61: Comparison of **UV-Vis** spectra of ***EE*-1** and ***EZ*-1** in DMSO.

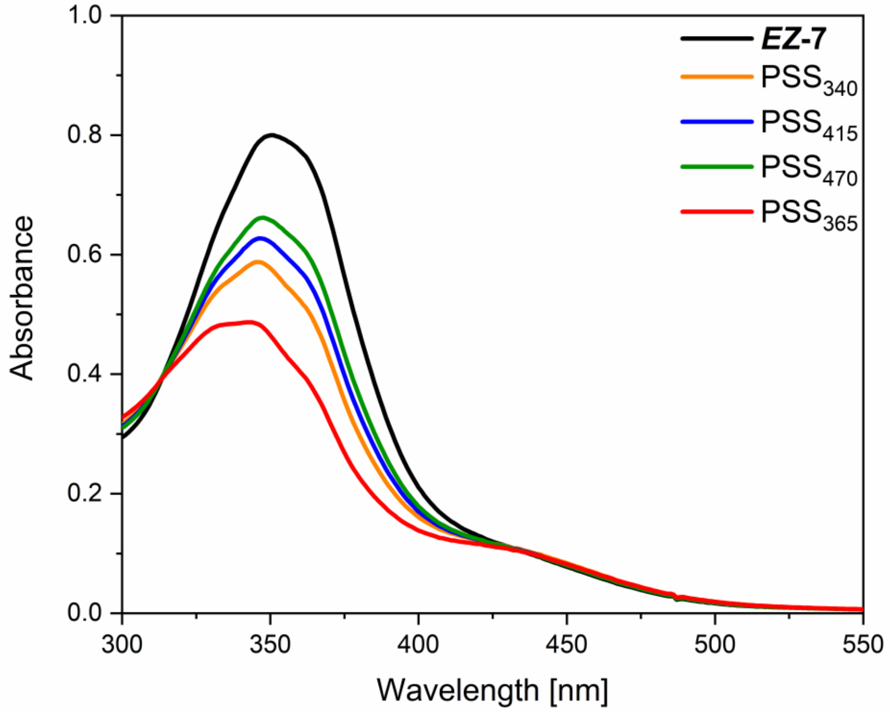

Figure S62: Normalized UV-Vis spectrum of ***EZ*-7** in DMSO at 20 °C upon sequential irradiation with λ_{irr} = 340, 415, 470 and 365 nm to the respective PSSs.

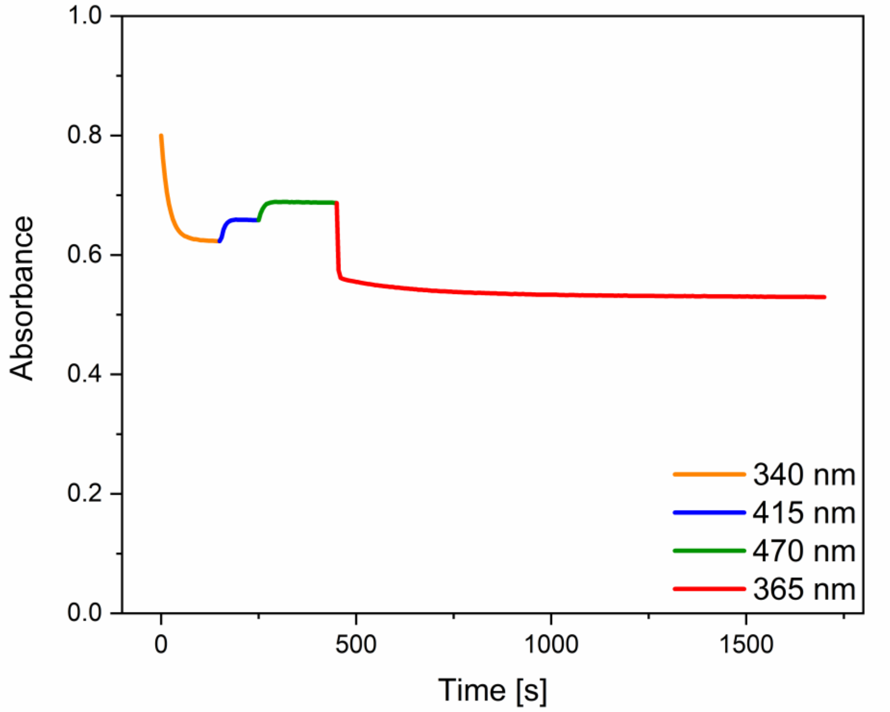

Figure S63: Change in absorbance at 340 nm (normalized) during the sequential irradiation of ***EZ*-7** with a 340, 415, 470 and 365 nm LED at 20 ºC in DMSO.

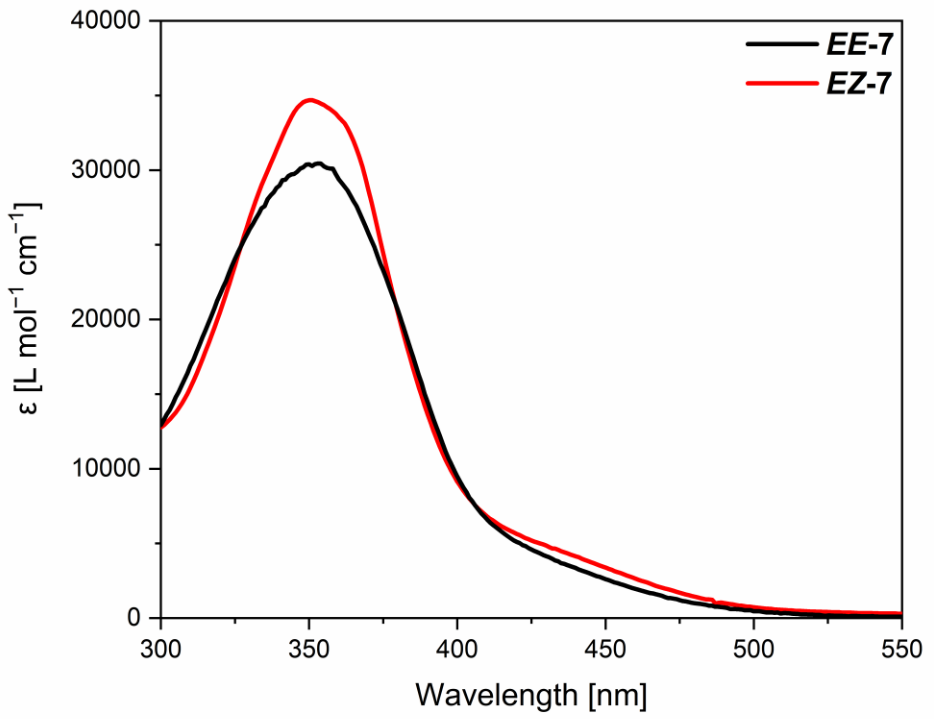

Figure S64: Comparison of **UV-Vis** spectra of ***EE*-7** and ***EZ*-7** in DMSO.

QY Determination

Solutions of unsubstituted azobenzene (**AB**) in MeCN (~3.0 × 10^{-5} M) were irradiated with a 340 nm LED at 20 °C and the spectra collected under constant irradiation until PSS was reached, following the evolution of the absorption maximum. UV-Vis spectra were recorded every second. Baseline corrections were carried out to account for baseline drifting, and the absorbance at 340 nm over the course of the measurement was extracted. The data was subsequently processed in COPASI, using a method outlined by Stranius & Börjesson, based on **Equation S1**:[6,7]

Equation S1

$$\frac{d[A]}{dt} = -\frac{QY_{EZ} \cdot I \cdot \beta_E(t)}{N_A \cdot V} + \frac{QY_{ZE} \cdot I \cdot \beta_Z(t)}{N_A \cdot V}$$

Where I is the molar photon flux (I = 6.45 × 10^{-6} mol s^{-1} for 340 nm, previously determined by chemical actinometry after a modified standard protocol)[7,8], N_A is Avogadro's constant, V is the total volume of the irradiated solution (2 mL), and β is the fraction of photons absorbed by either the *E* or *Z* isomer, a number that is determined using the PSS ratio (determined *via* ^{1}H NMR) and the corresponding molar extinction coefficients for each isomer. This equation was used to determine the QYs of **AB**.

Table S1: Molar extinction coefficients at 20 ºC in CH_3CN at 340 nm, PSS obtained upon irradiation with a 340 nm LED and QY for **AB**.

Entry	Compound	ε(*E*) [M^{-1} cm^{-1}]	ε(*Z*) [M^{-1} cm^{-1}]	PSS ratio λ_{MAX} = 340 nm (*E*:*Z*)	QY (%) *E* → *Z*	QY (%) *Z* → *E*
1	**AB**	11400	1800	2:98	15.0	1.8

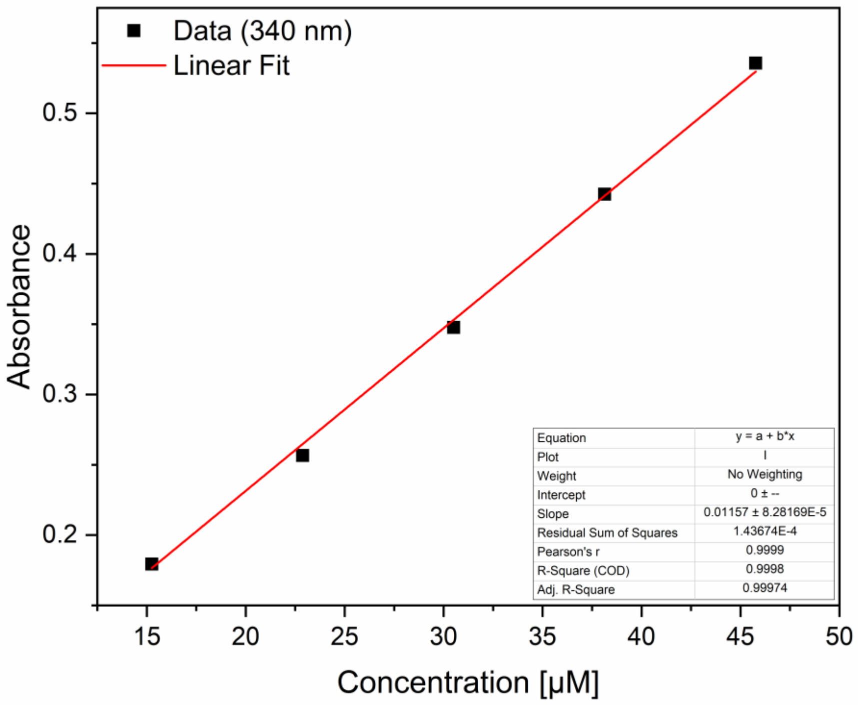

Figure S65: Determination of the molar absorptivity in acetonitrile at 20 ºC.

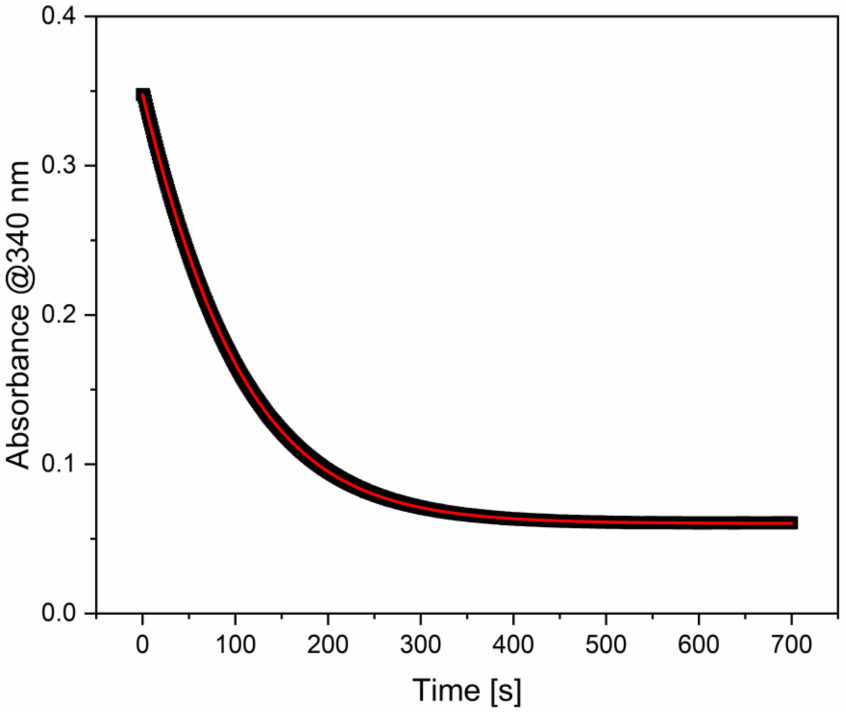

Figure S66: Change in absorbance at 340 nm during the irradiation of **AB** with a 340 nm LED at 20 ºC in CH_3CN. The red line represents the fit as exported from COPASI.

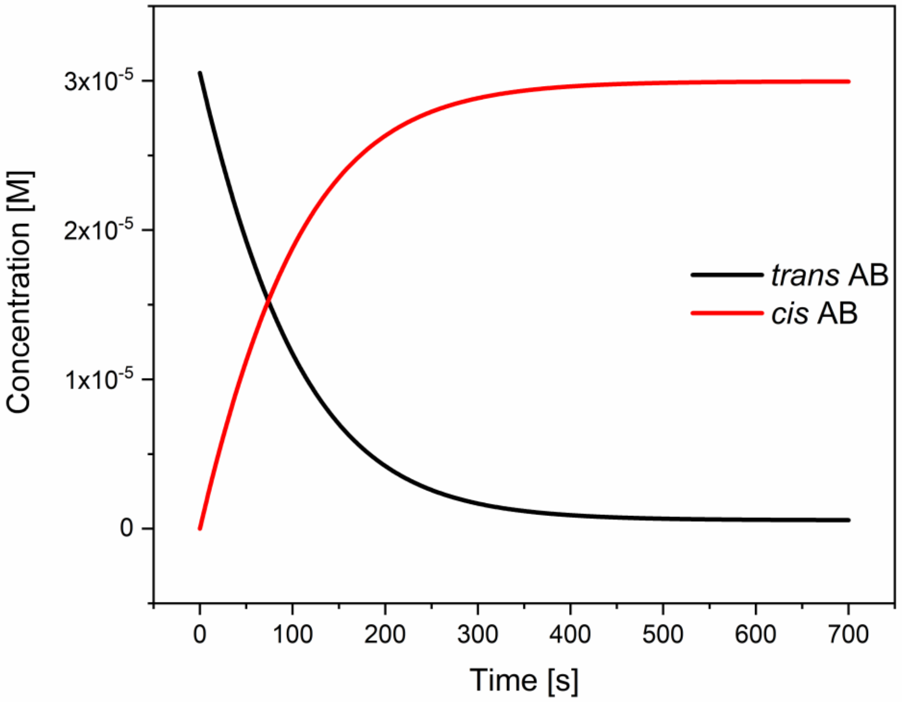

Figure S67: Evolution of the concentration of *trans* **AB** and *cis* **AB** during the irradiation of *trans* **AB** with a 340 nm LED as fitted by **Equation S1** in COPASI.

Computational Analysis and Simulated UV-Vis Spectra

AODs **1-7** were optimized considering the four possible stable states the respective dyad can occupy (S1 with *EE* configuration, N=N double bond is *E*, C=C double bond is *E*; S2 with *ZE* configuration, N=N double bond is *Z*, C=C double bond is *E*; S3 with *ZZ* configuration, N=N double bond is *Z*, C=C double bond is *Z*; S4 with *EZ* configuration, N=N double bond is *E*, C=C double bond is *Z*). Frequency calculations were performed and no imaginary frequencies observed, confirming that the geometries correspond to a local minimum.

The structures were optimized with the r2scan-3c method (verytightopt; defgrid3 for gas phase), with the Orca 5.0.4 software package,[9] first in the gas phase and afterwards in DMSO using the SMD solvation model.[10]

The UV-Vis spectra of the four different *E* and *Z* configurations were calculated at the TD-PBE0/def2-TZVP level over the first 25 singlet transitions on the geometries calculated in DMSO. The SMD implicit solvent method (for DMSO) was applied.[10]

All xyz coordinates are reported as separate files, while the simulated UV-Vis of the four different configurations are reported below (top left S1, top right S2, bottom left S3, bottom right S4).

Table S2: Calculated energy differences between stable states S2/S3/S4 and S1.

Entry	Dyad	DMSO		
		ΔG_{calc} S2-S1 [kcal mol-1]	ΔG_{calc} S3-S1 [kcal mol^{-1}]	ΔG_{calc} S4-S1 [kcal mol^{-1}]
1	**1**	9.4	10.4	1.2
2	**2**	9.2	9.3	1.9
3	**3**	9.2	9.7	1.9
4	**4**	9.8	11.4	1.9
5	**5**	8.6	9.2	1.1
6	**6**	8.8	9.9	2.0
7	**7**	9.5	11.6	1.0

Simulated UV-Vis Spectra

Azobenzene-Oxindole Dyad 1

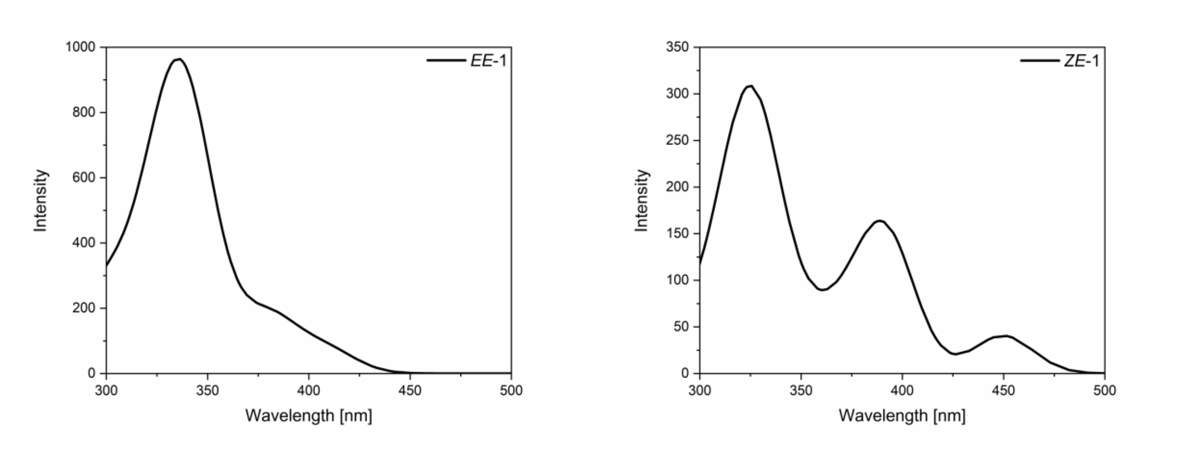

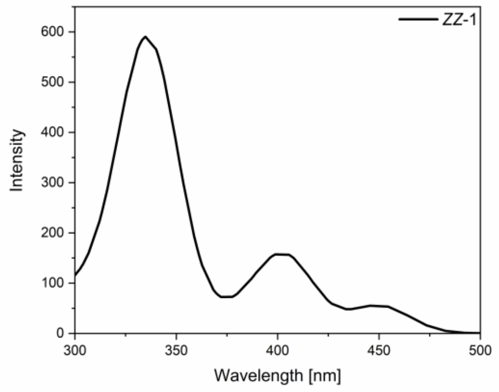

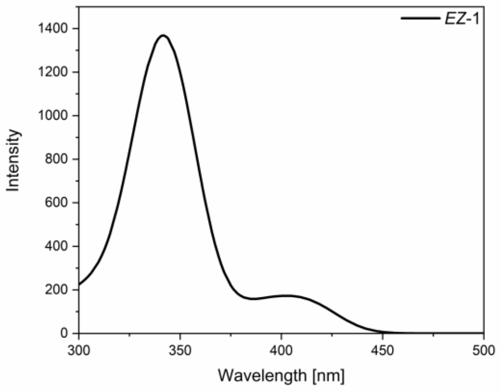

Azobenzene-Oxindole Dyad 2

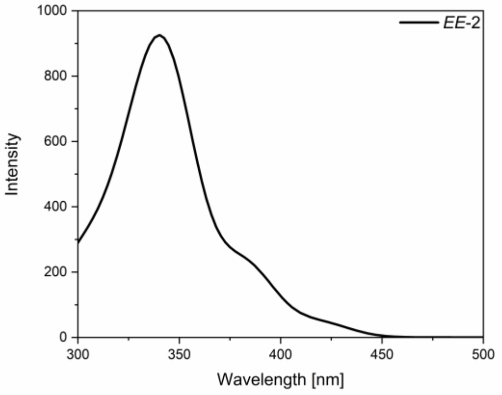

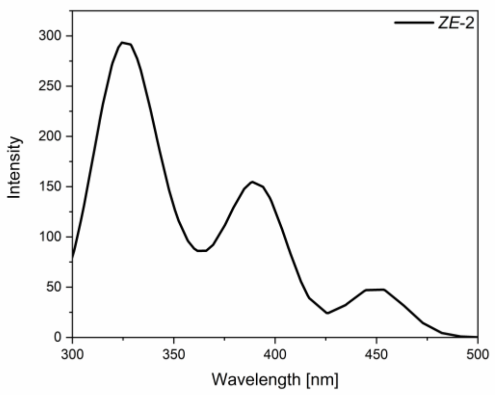

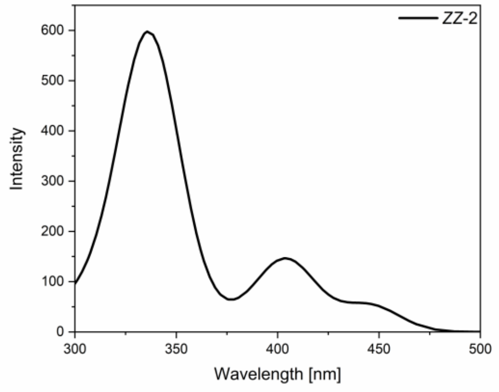

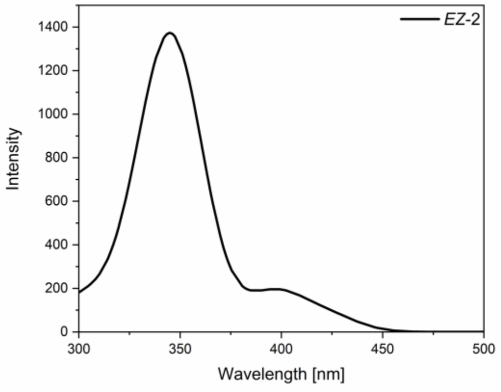

Azobenzene-Oxindole Dyad 3

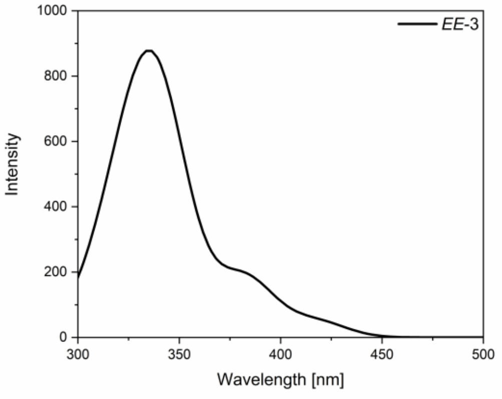

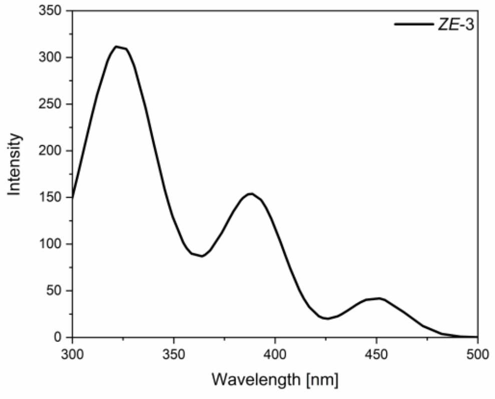

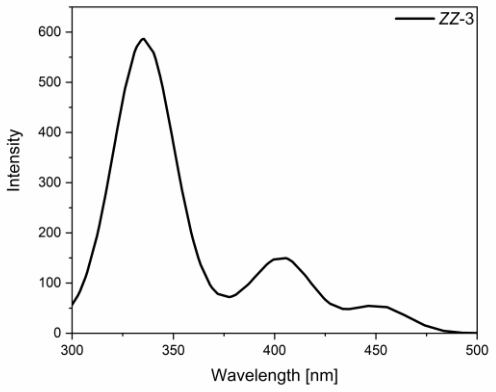

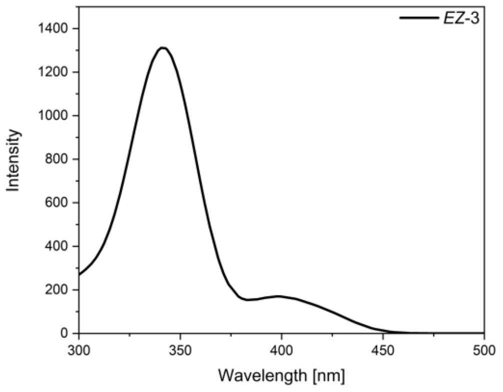

Azobenzene-Oxindole Dyad 4

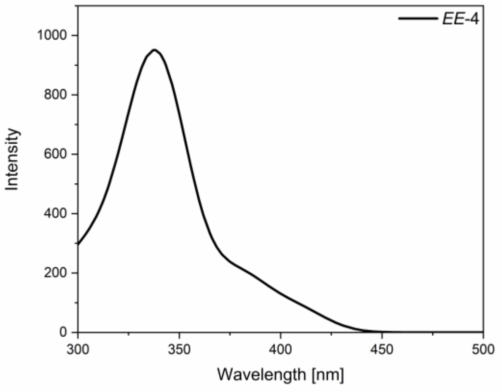

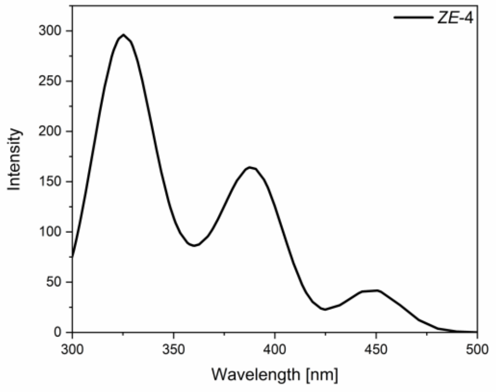

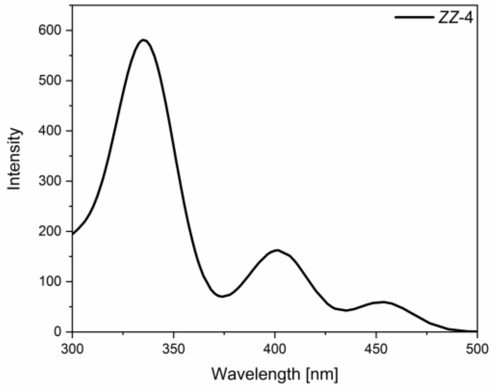

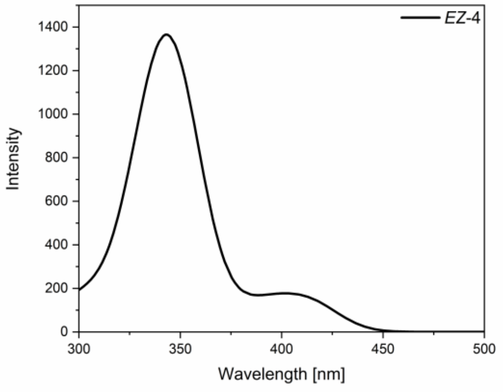

Azobenzene-Oxindole Dyad 5

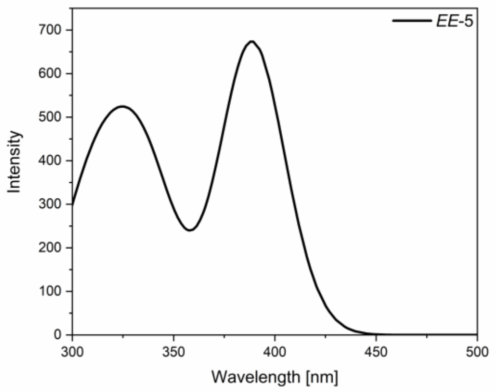

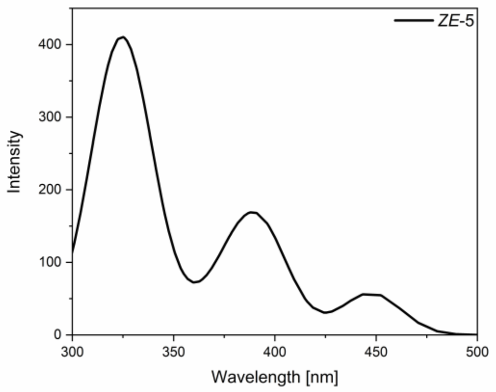

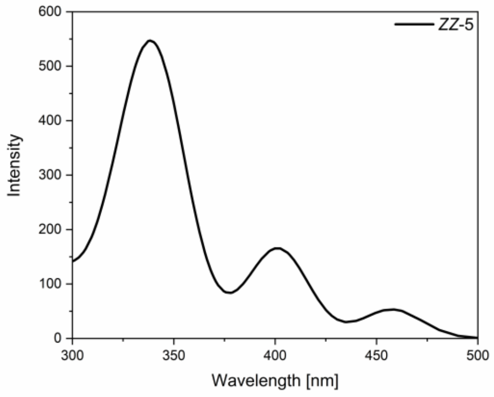

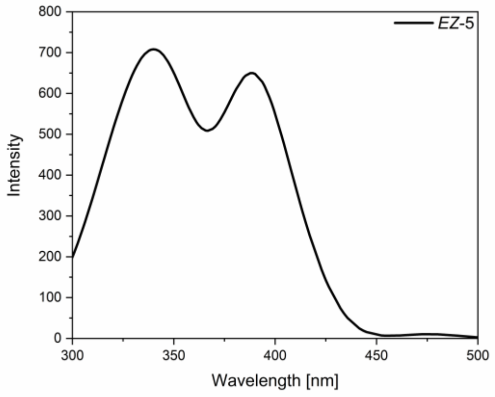

Azobenzene-Oxindole Dyad 6

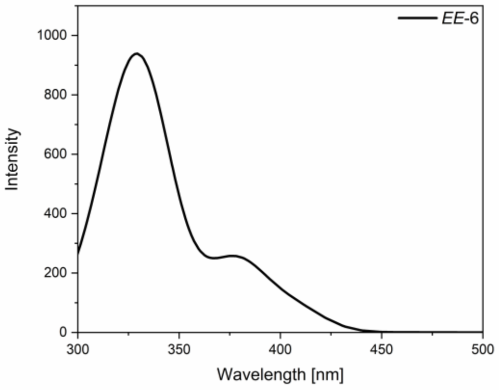

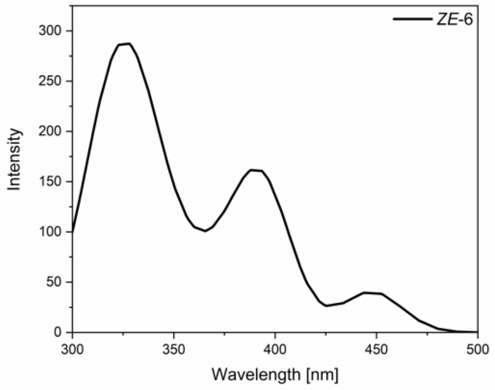

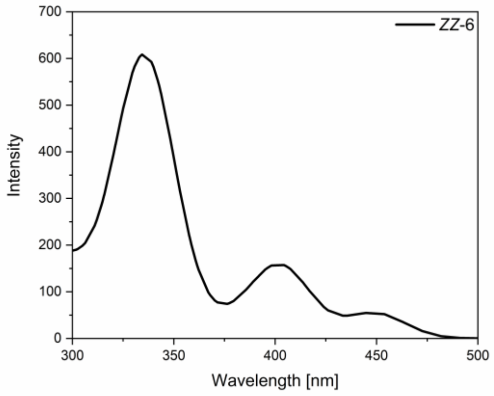

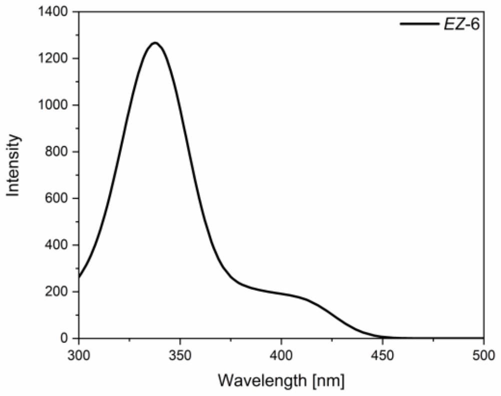

Azobenzene-Oxindole Dyad 7

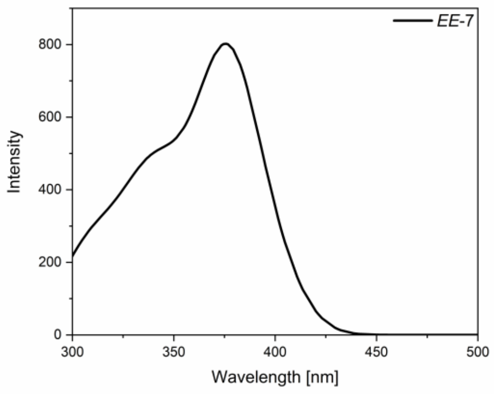

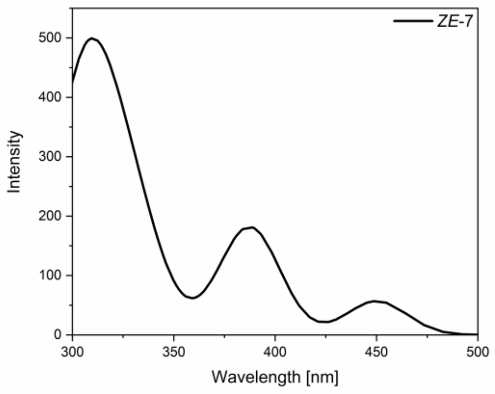

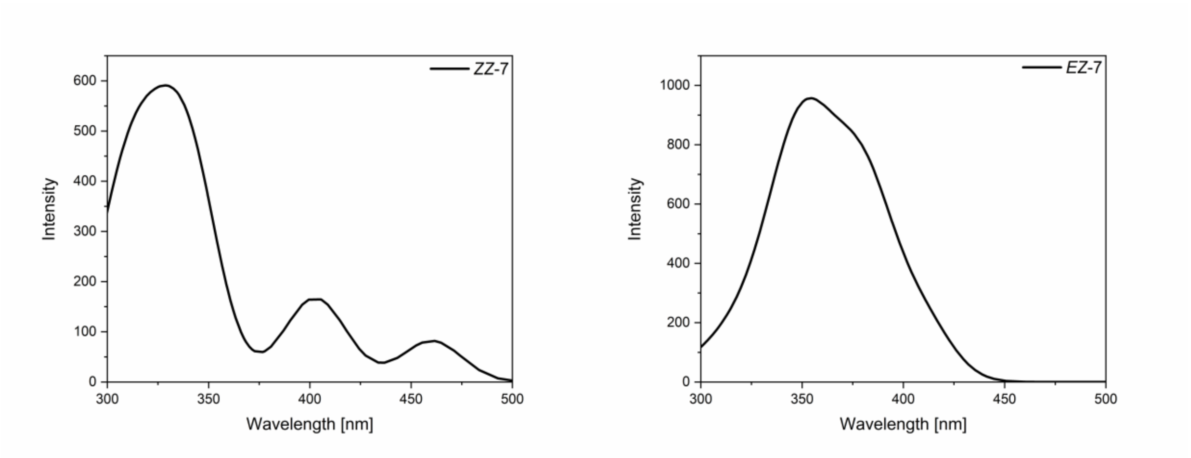
ZZ-7
Intensity
Wavelength [nm]
0
100
200
300
400
500
600
300
350
400
450
500
EZ-7
Intensity
Wavelength [nm]
0
200
400
600
800
1000
300
350
400
450
500

References

[1] C. Feldmeier, H. Bartling, E. Riedle, R. M. Gschwind, *J. Magn. Reson.* **2013**, *232*, 39–44.
[2] F. T. Bergmann, S. Hoops, B. Klahn, U. Kummer, P. Mendes, J. Pahle, S. Sahle, *J. Biotech.* **2017**, *261*, 215–220.
[3] K. Rustler, P. Nitschke, S. Zahnbrecher, J. Zach, S. Crespi, B. König, *J. Org. Chem.* **2020**, *85*, 4079–4088.
[4] P. Gerstel, S. Klumpp, F. Hennrich, A. Poschlad, V. Meded, E. Blasco, W. Wenzel, M. M. Kappes, C. Barner-Kowollik, *ACS Macro Letters* **2014**, *3*, 10–15.
[5] D. Doellerer, D. R. S. Pooler, A. Guinart, S. Crespi, B. L. Feringa, *Chem. Eur. J.* **2023**, *29*, e202301634.
[6] S. Hoops, S. Sahle, R. Gauges, C. Lee, J. Pahle, N. Simus, M. Singhal, L. Xu, P. Mendes, U. Kummer, *Bioinformatics* **2006**, *22*, 3067–3074.
[7] K. Stranius, K. Börjesson, *Sci. Rep.* **2017**, *7*, 41145.
[8] H. J. Kuhn, S. E. Braslavsky, R. Schmidt, *Pure Appl. Chem.* **2004**, *76*, 2105–2146.
[9] F. Neese, F. Wennmohs, U. Becker, C. Riplinger, *J. Chem. Phys.* **2020**, *152*, 224108.
[10] A. V. Marenich, C. J. Cramer, D. G. Truhlar, *J. Phys. Chem. B* **2009**, *113*, 6378–6396.

Appendix

NMR Spectra

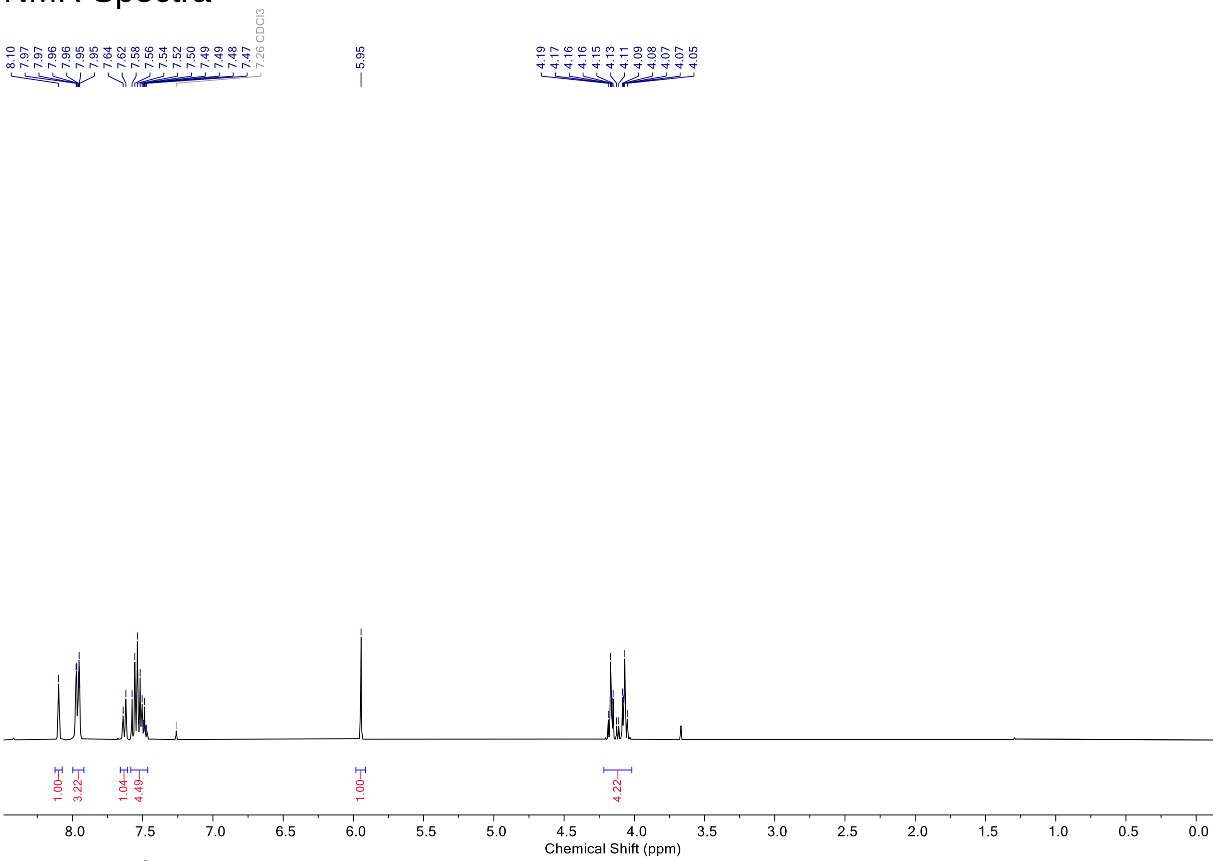

Figure S68: ¹H NMR spectrum of **8** ($CDCl_3$).

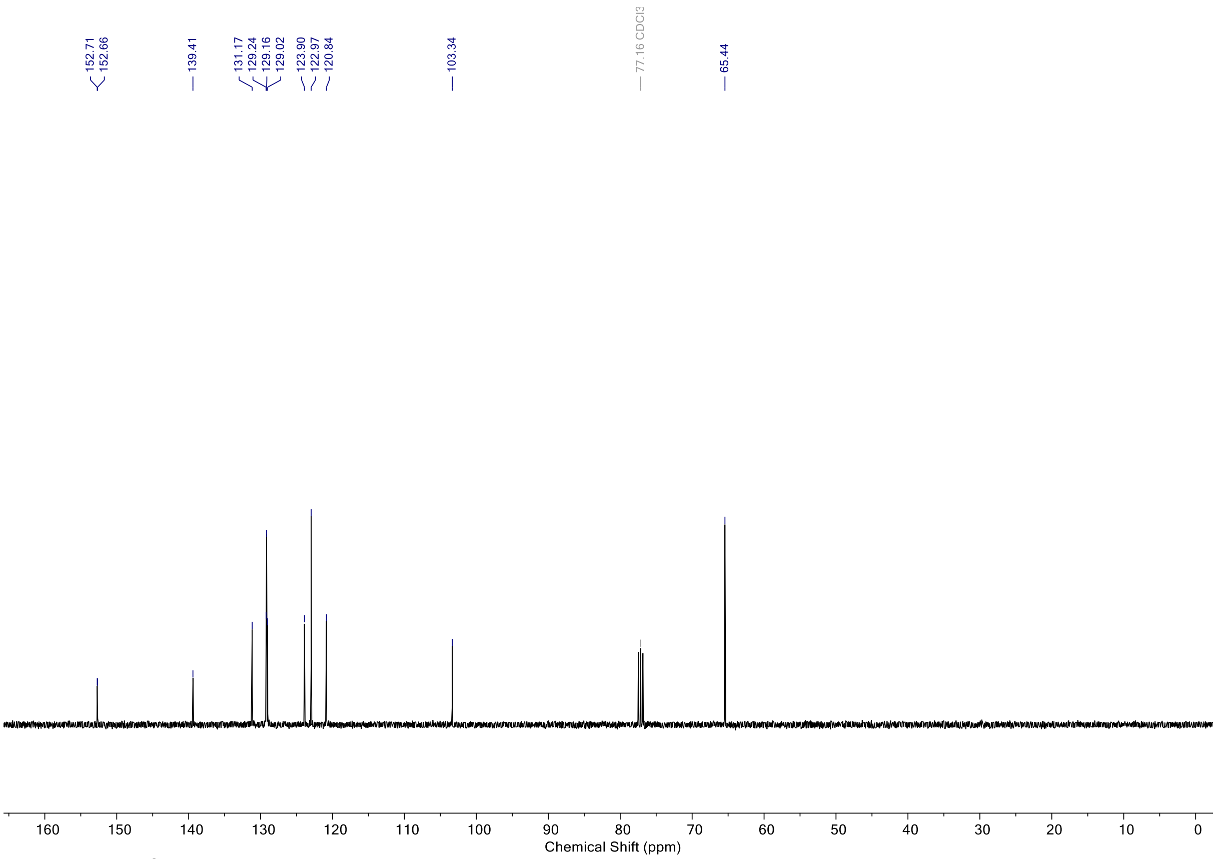

Figure S69: ^{13}C{^{1}H} NMR spectrum of **8** ($CDCl_3$).

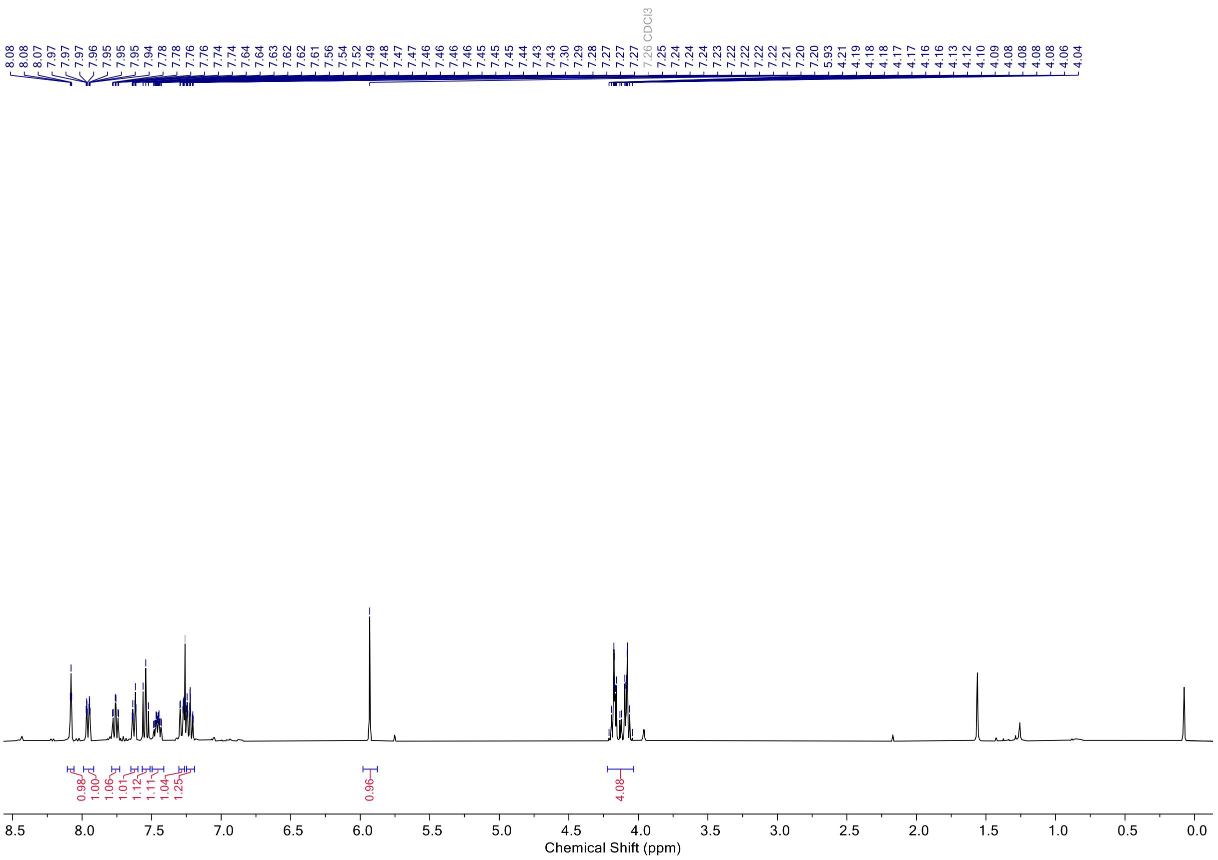

Figure S70: **¹H NMR** spectrum of **9** ($CDCl_3$).

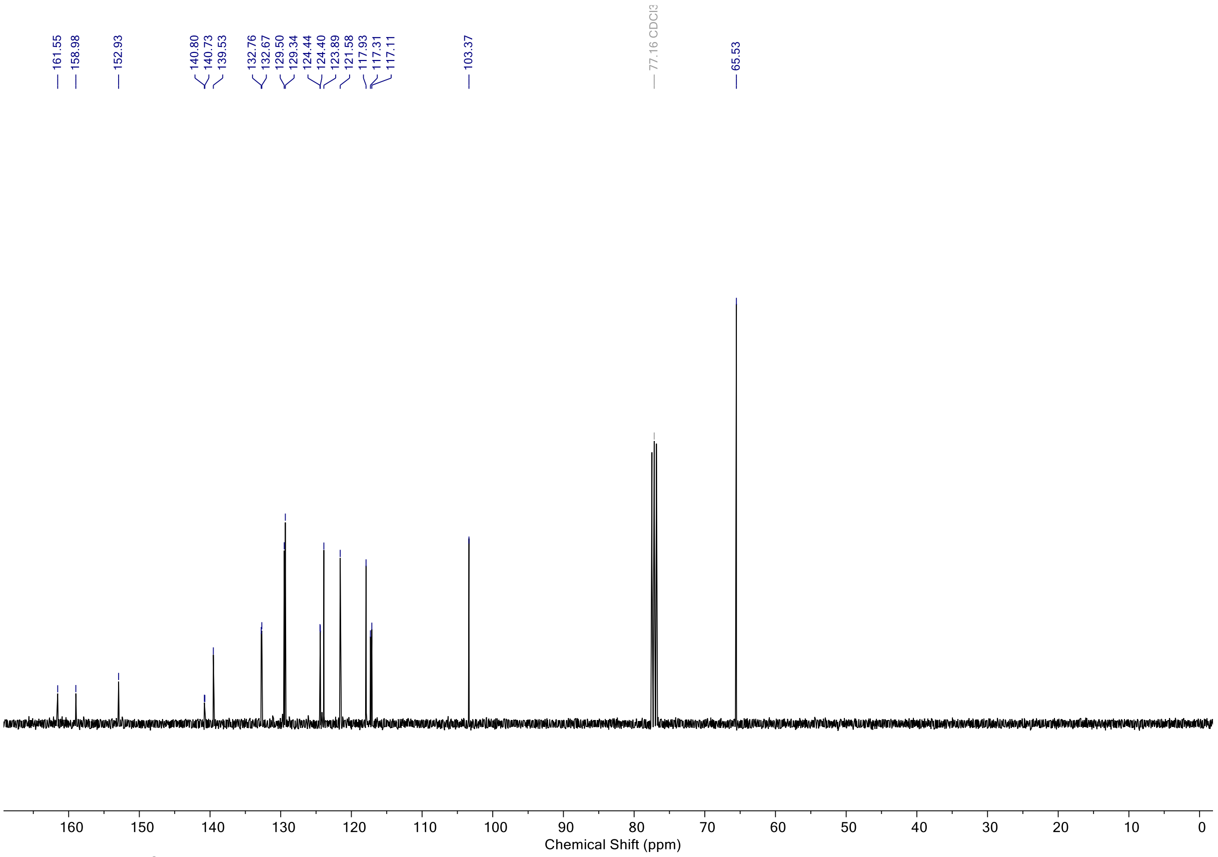

Figure S71: **$^{13}C\{^{1}H\}$ NMR** spectrum of **9** ($CDCl_3$).

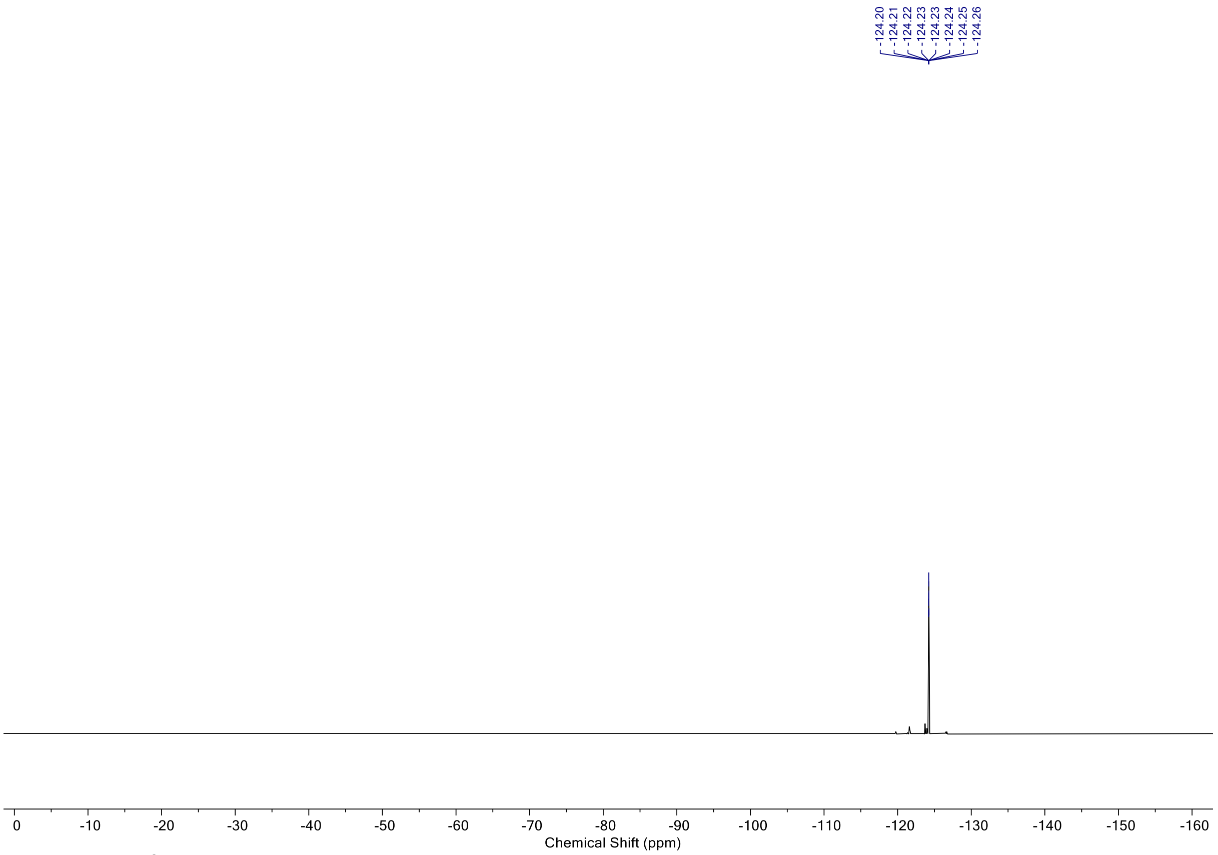

Figure S72: ^{19}F NMR spectrum of **9** ($CDCl_3$).

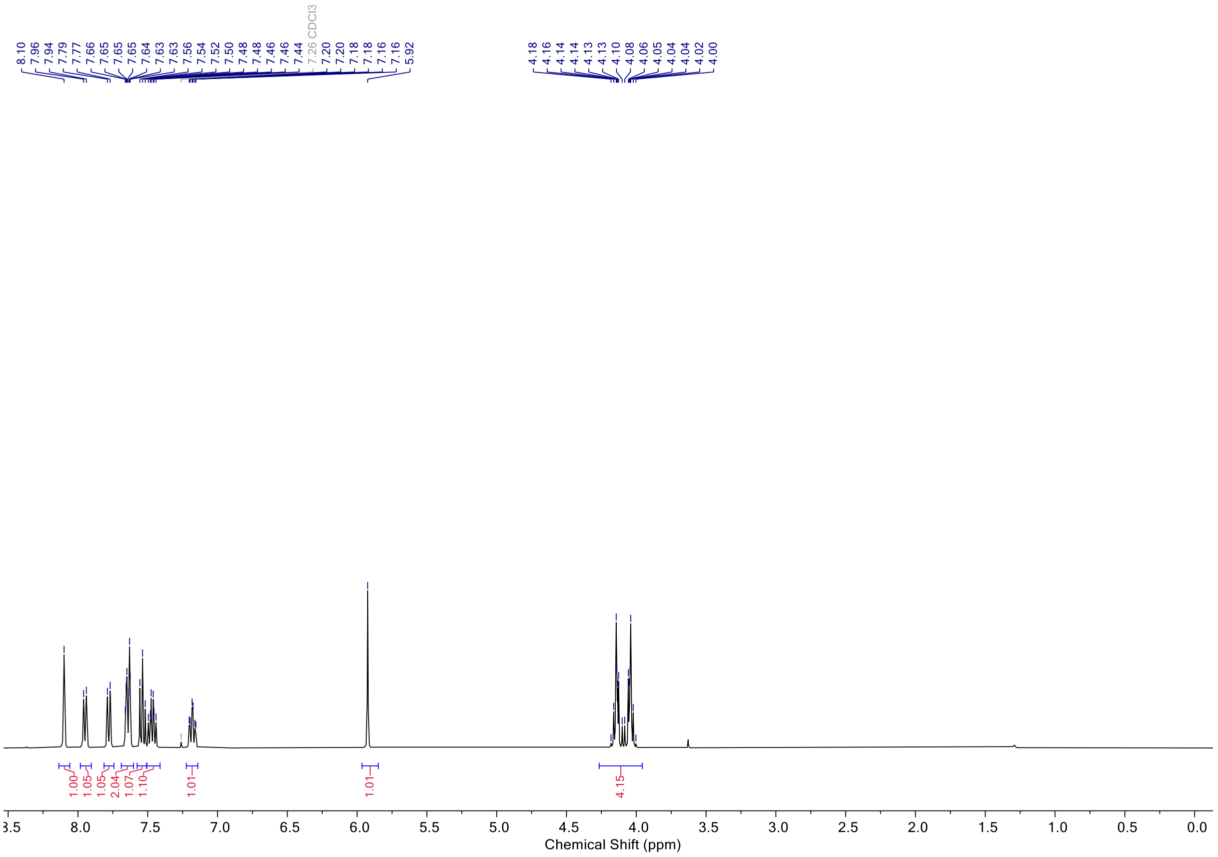

Figure S73: ^{1}H NMR spectrum of **10** ($CDCl_3$).

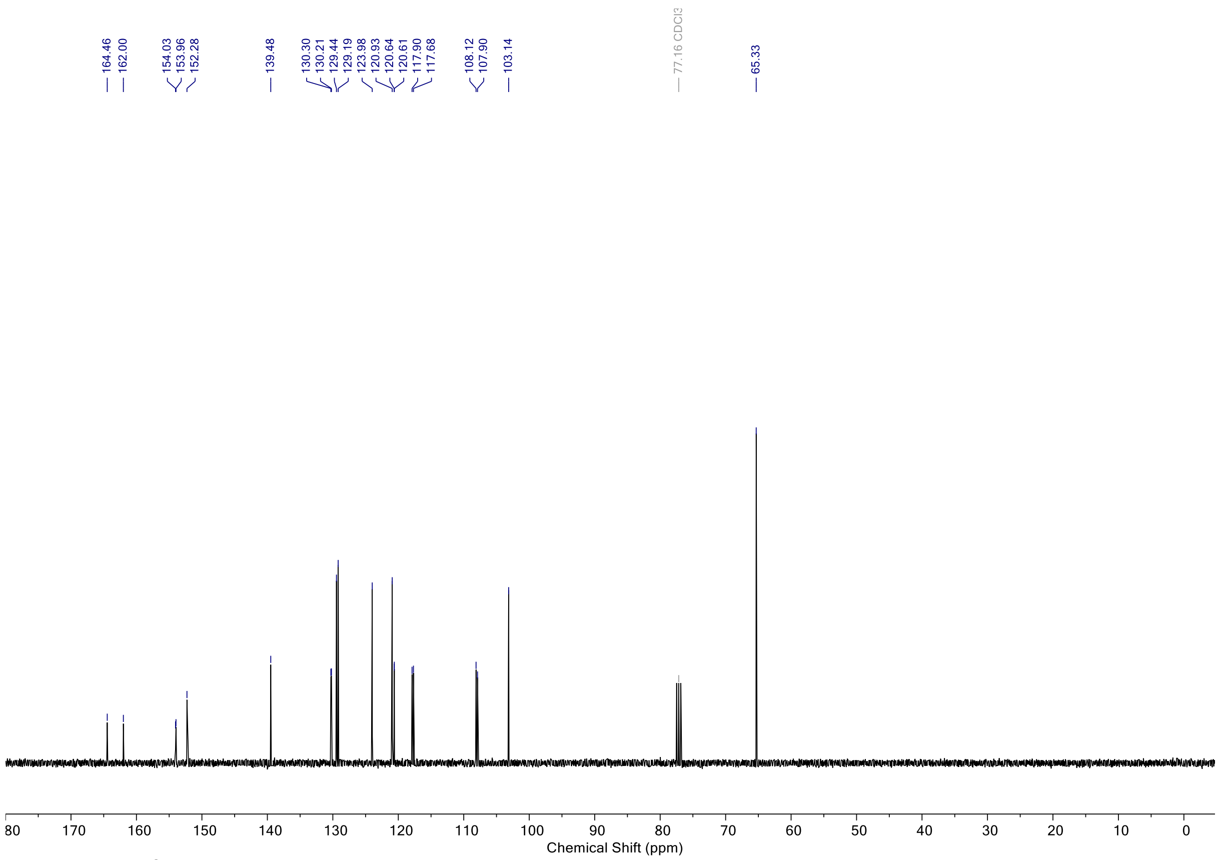

Figure S74: ^{13}C{^{1}H} NMR spectrum of **10** ($CDCl_3$).

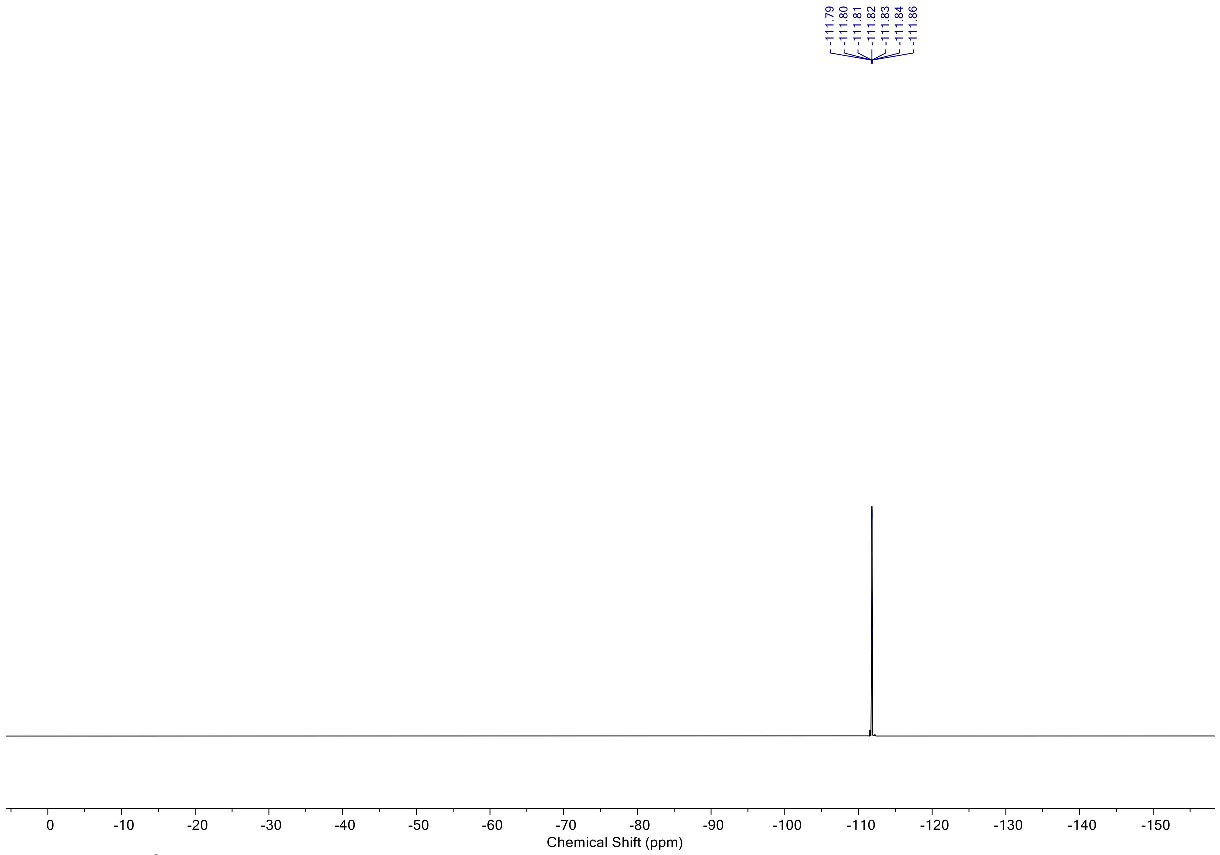

Figure S75: ^{19}F NMR spectrum of **10** ($CDCl_3$).

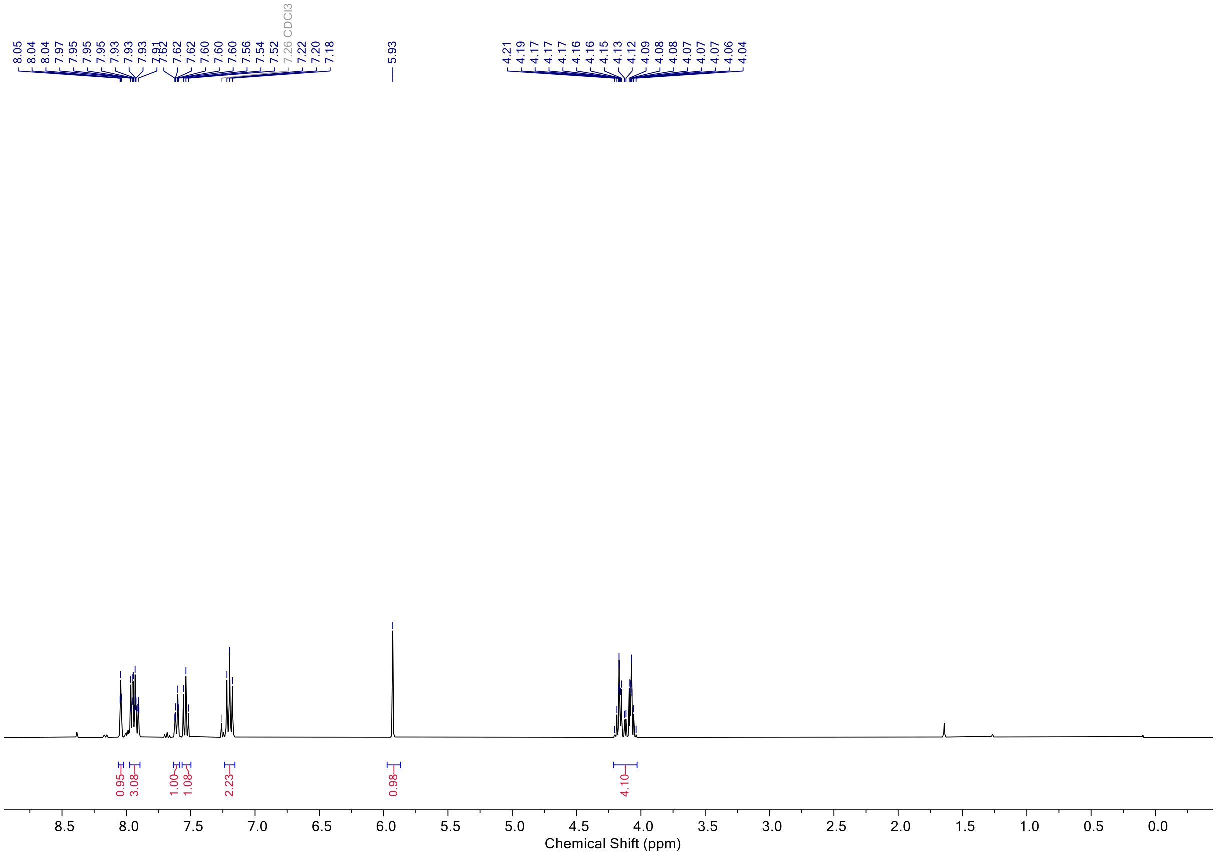

Figure S76: **^{1}H NMR** spectrum of **11** ($CDCl_3$).

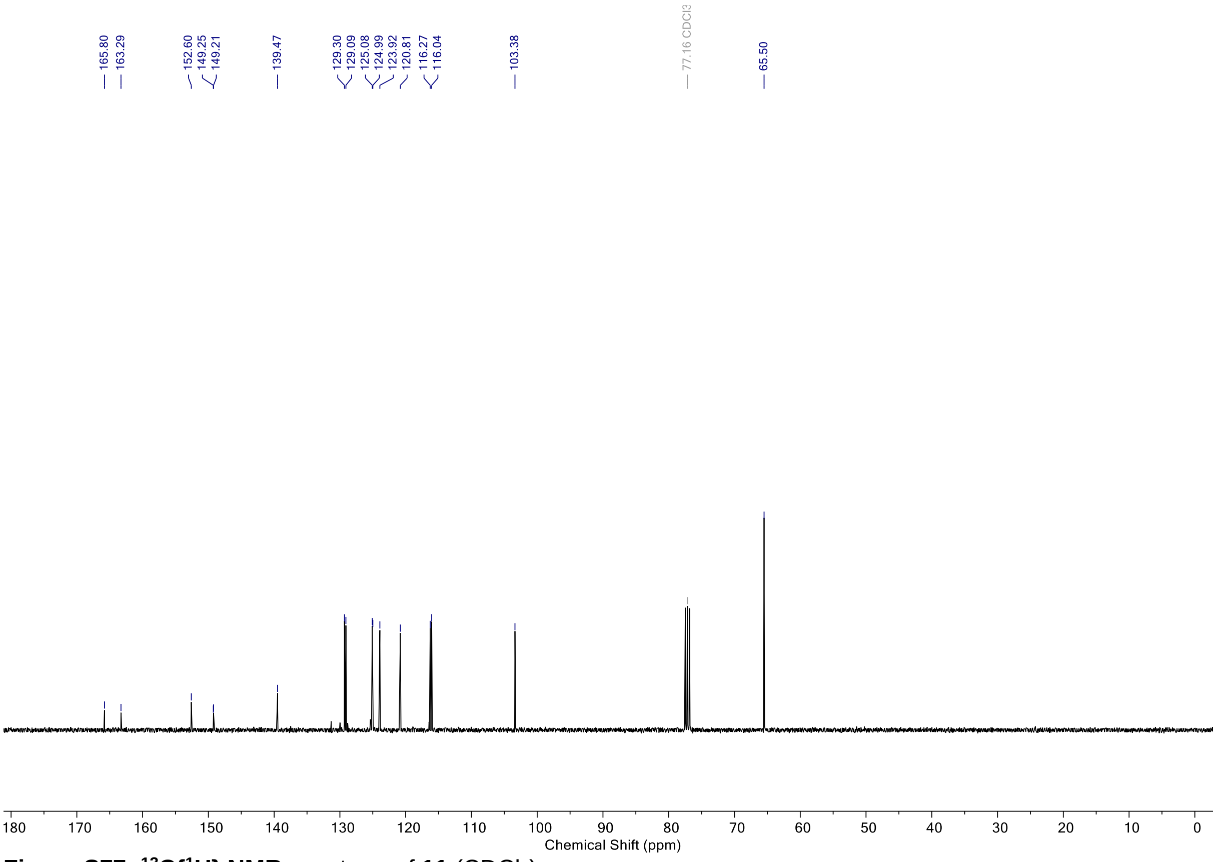

Figure S77: **^{13}C{^{1}H} NMR** spectrum of **11** ($CDCl_3$).

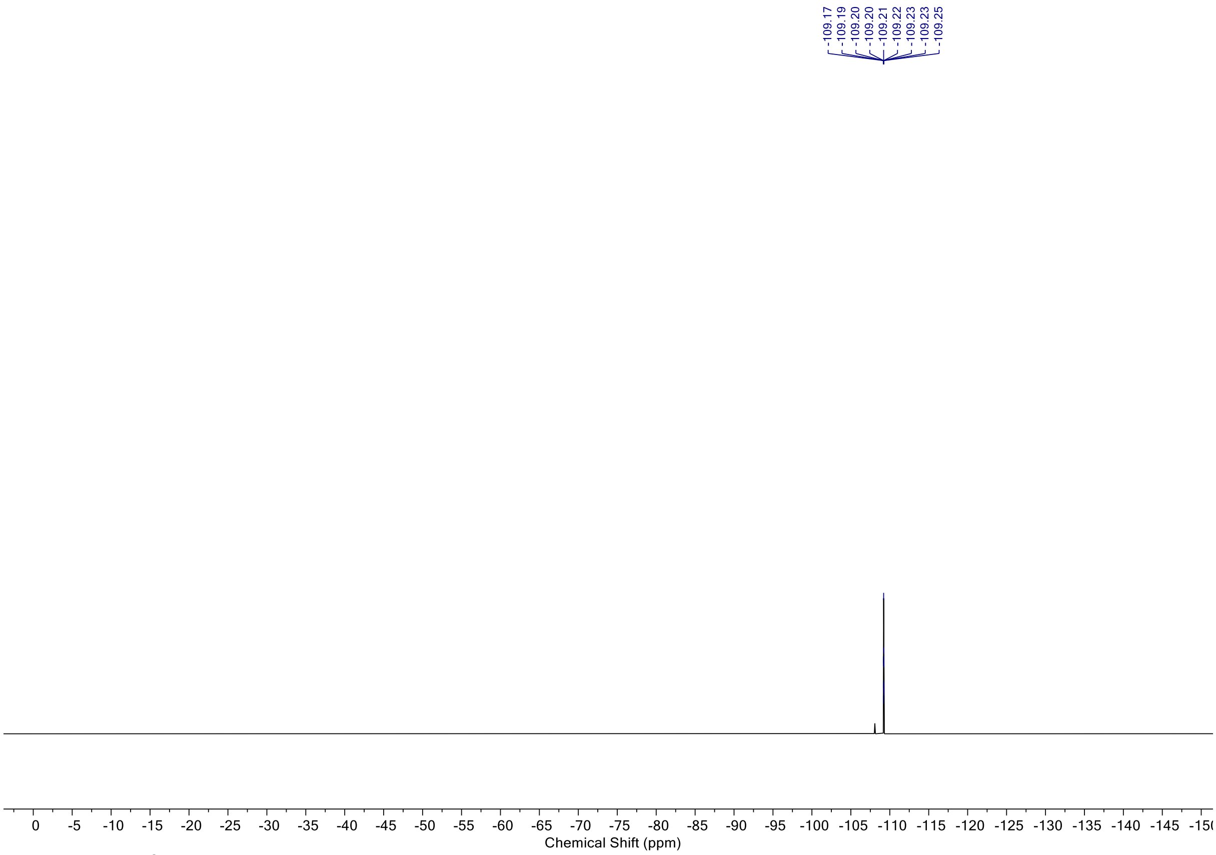

Figure S78: ^{19}F NMR spectrum of **11** ($CDCl_3$).

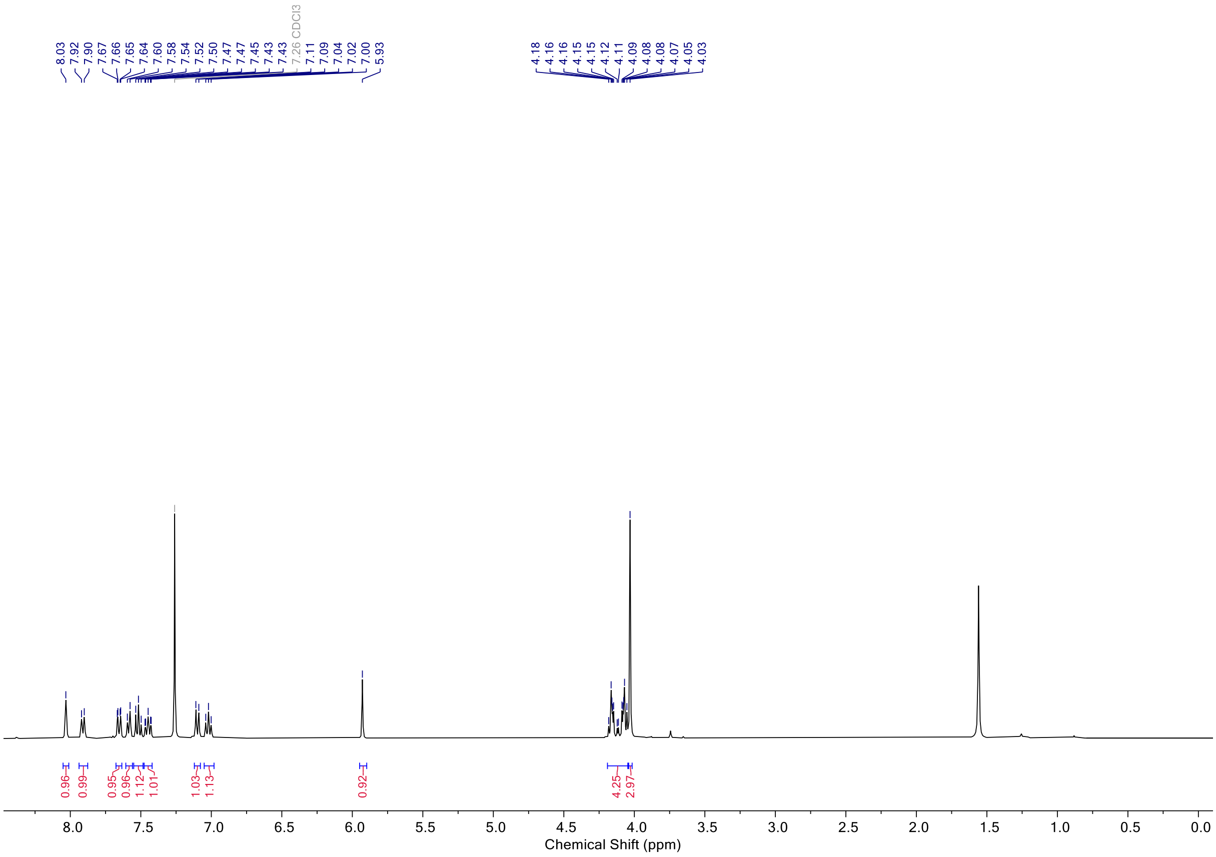

Figure S79: ^{1}H NMR spectrum of **12** ($CDCl_3$).

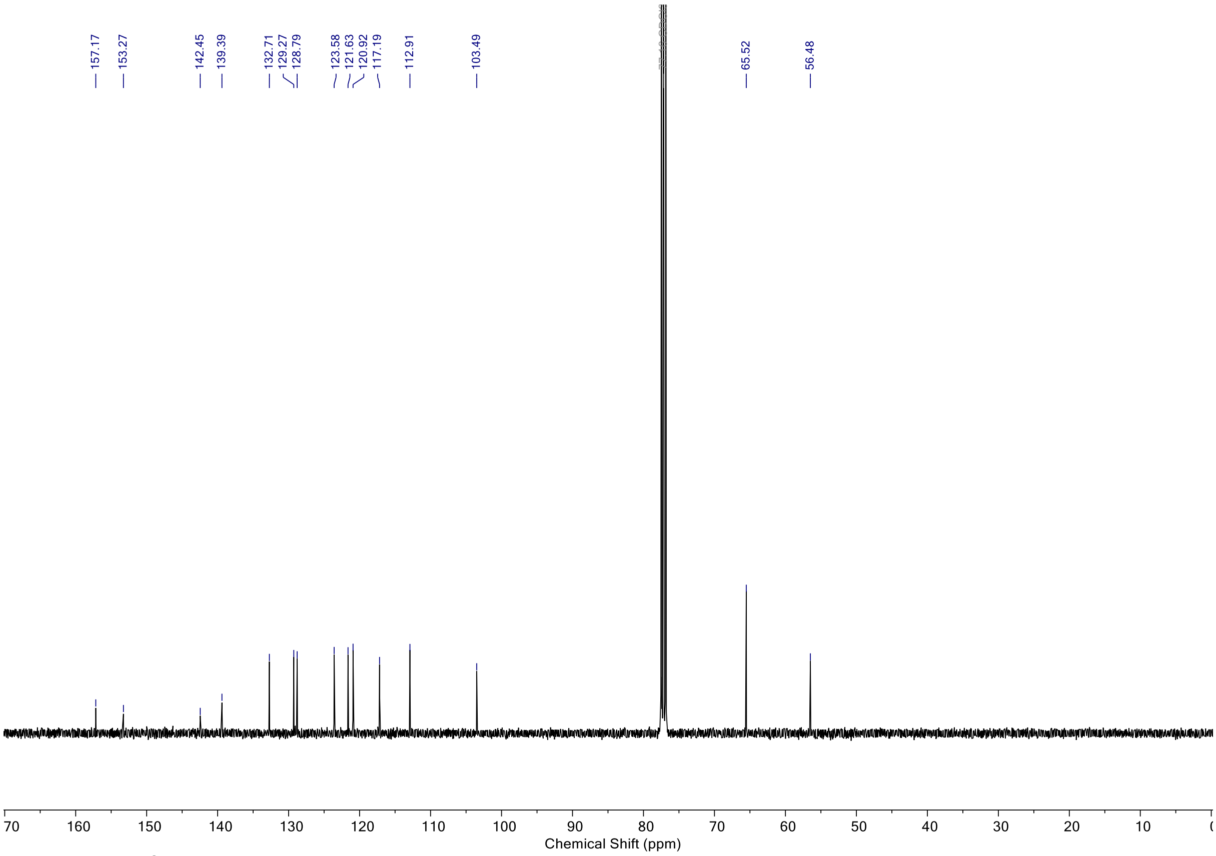

Figure S80: ^{13}**C{**^{1}**H} NMR** spectrum of **12** (CDCl₃).

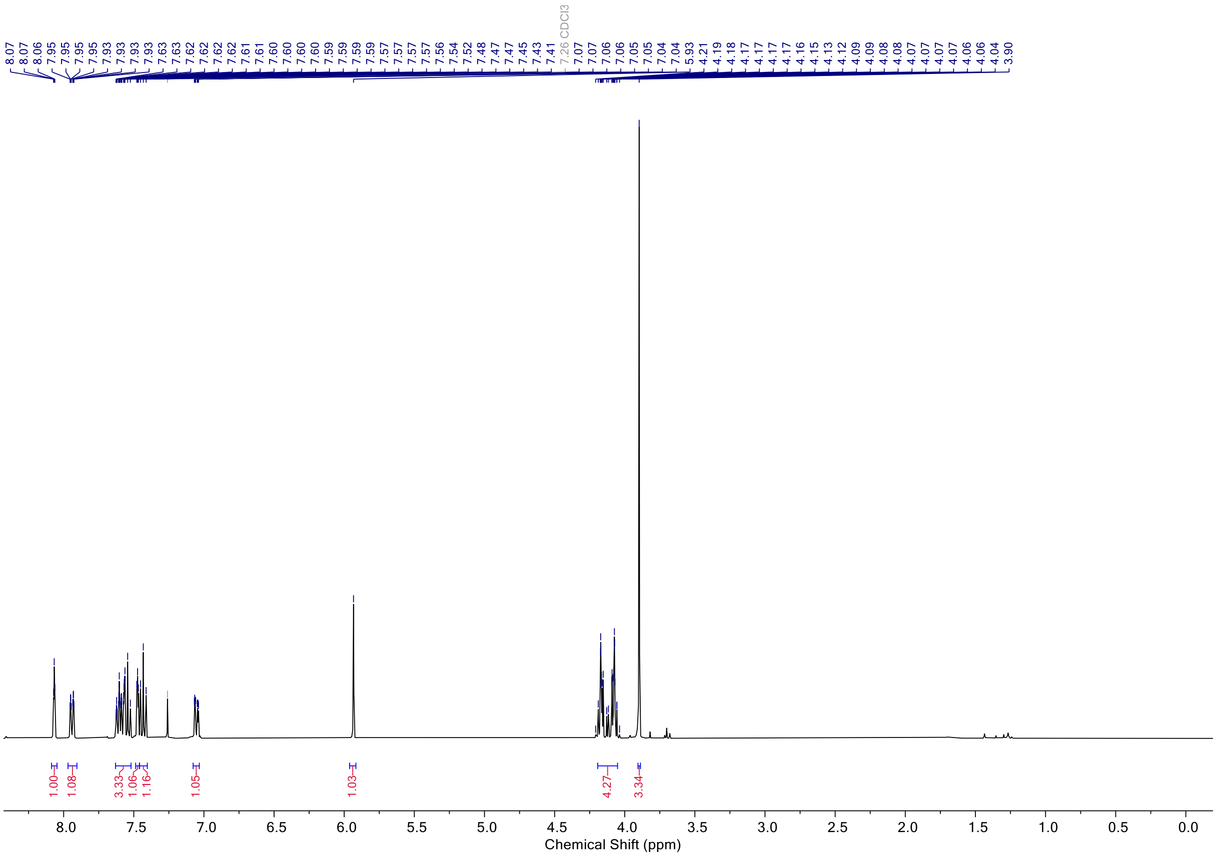

Figure S81: ^{1}**H NMR** spectrum of **13** (CDCl₃).

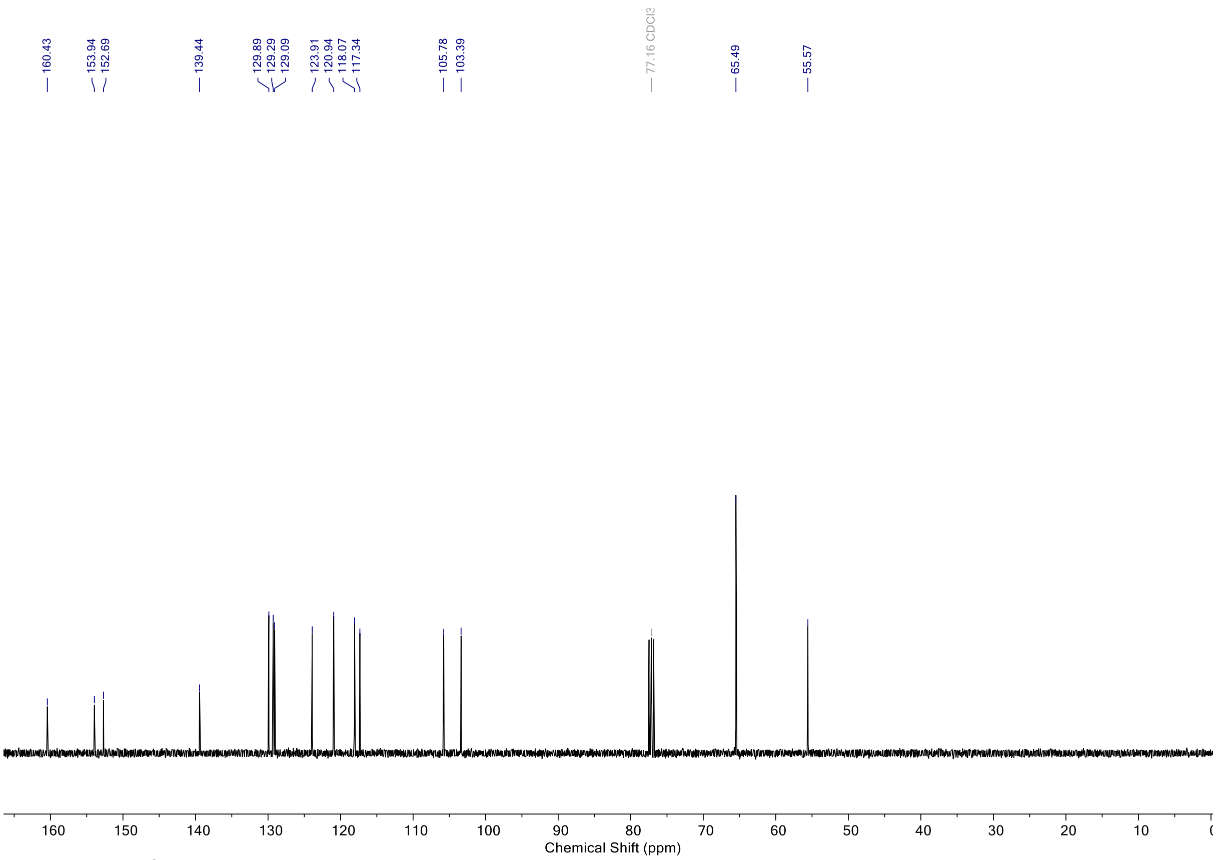

Figure S82: $^{13}C\{^{1}H\}$ **NMR** spectrum of **13** ($CDCl_3$).

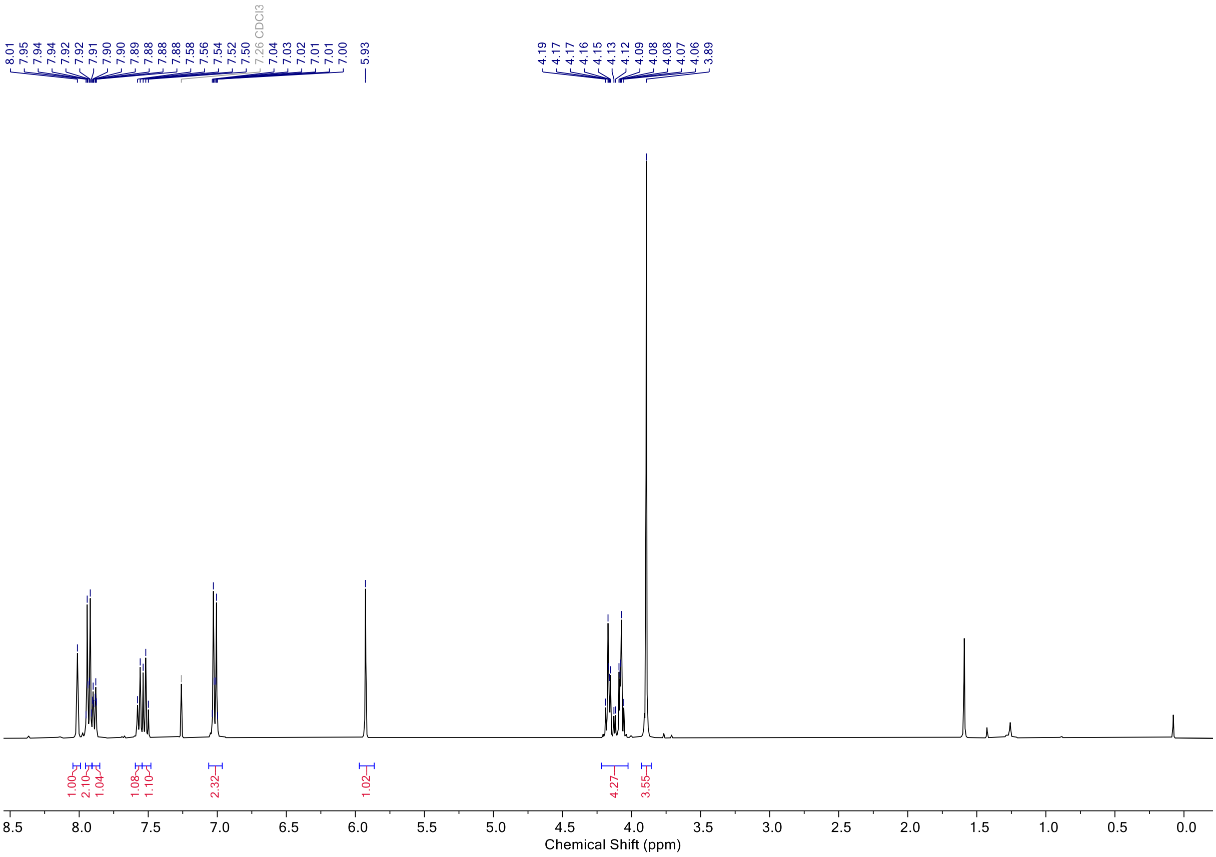

Figure S83: ^{1}**H NMR** spectrum of **14** ($CDCl_3$).

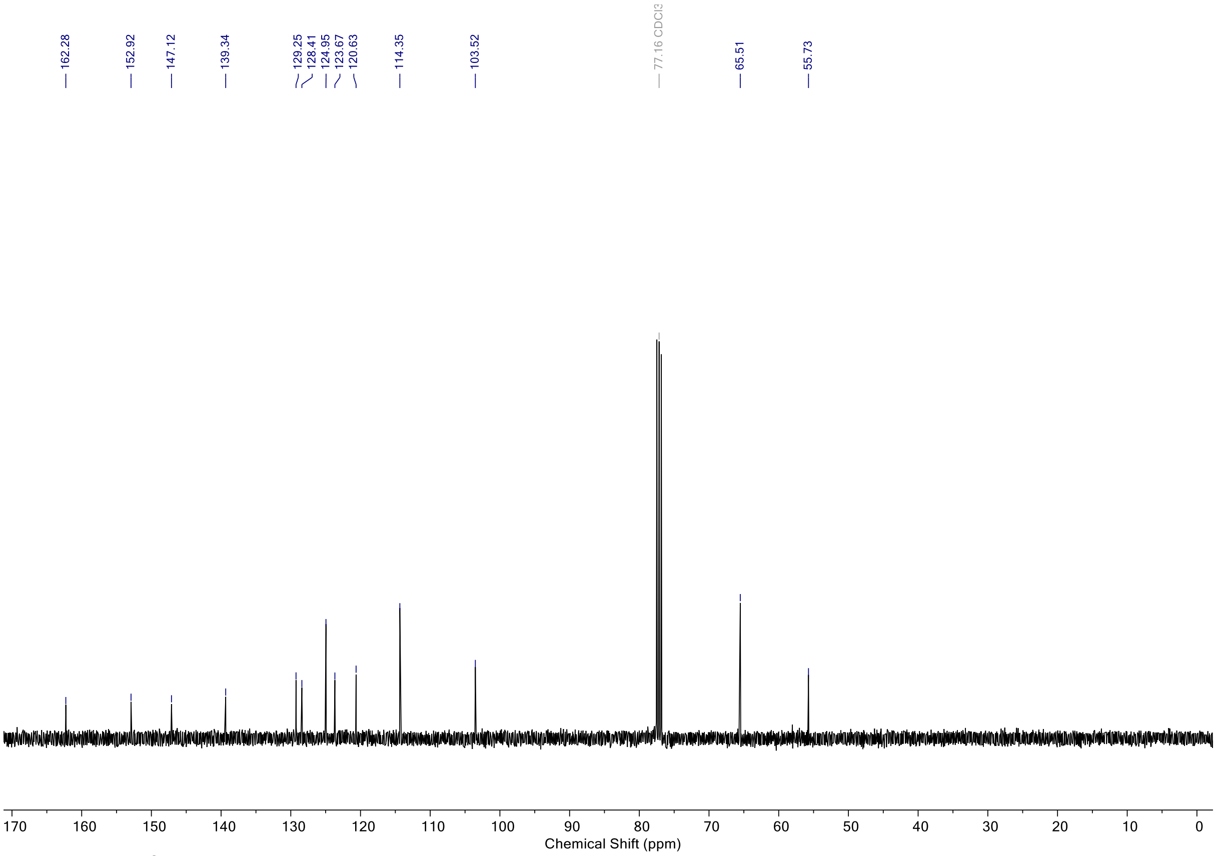

Figure S84: ^{13}**C{**^{1}**H} NMR** spectrum of **14** ($CDCl_3$).

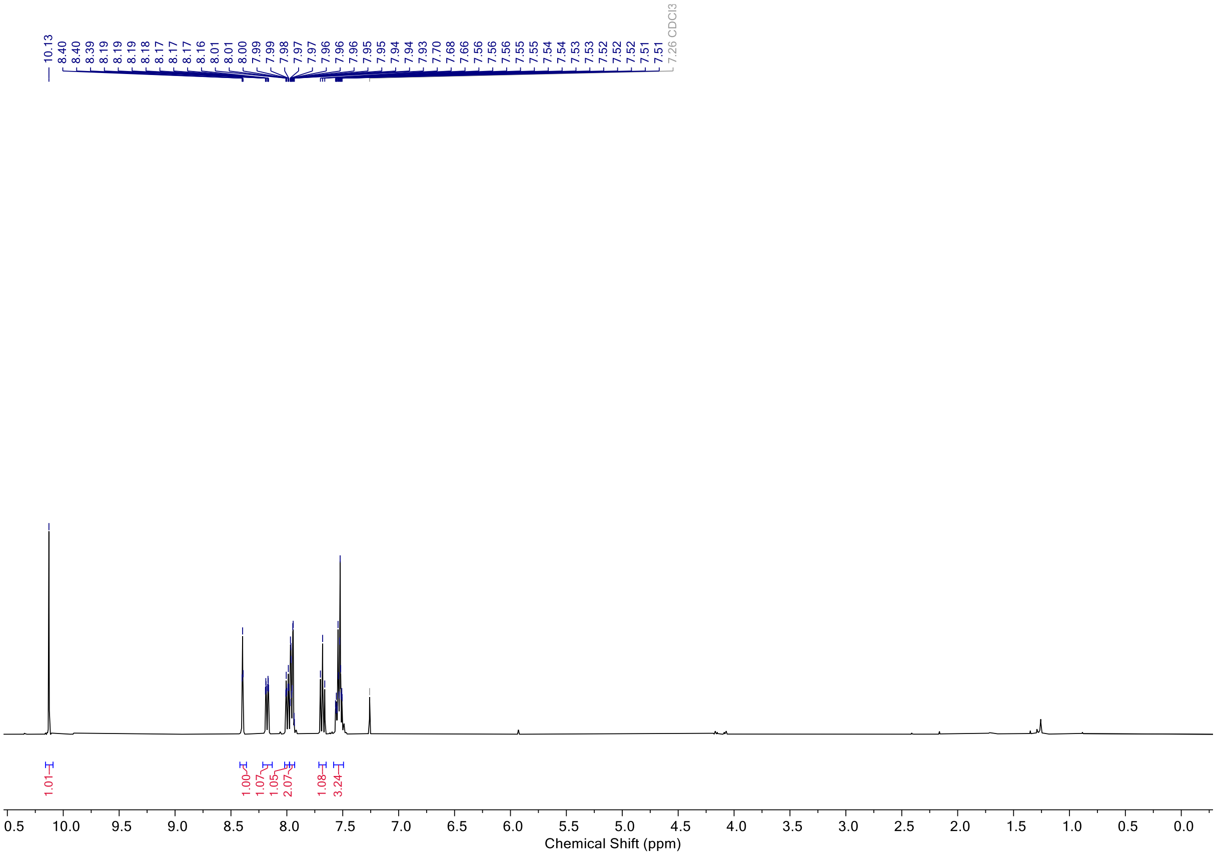

Figure S85: ^{1}**H NMR** spectrum of **15** ($CDCl_3$).

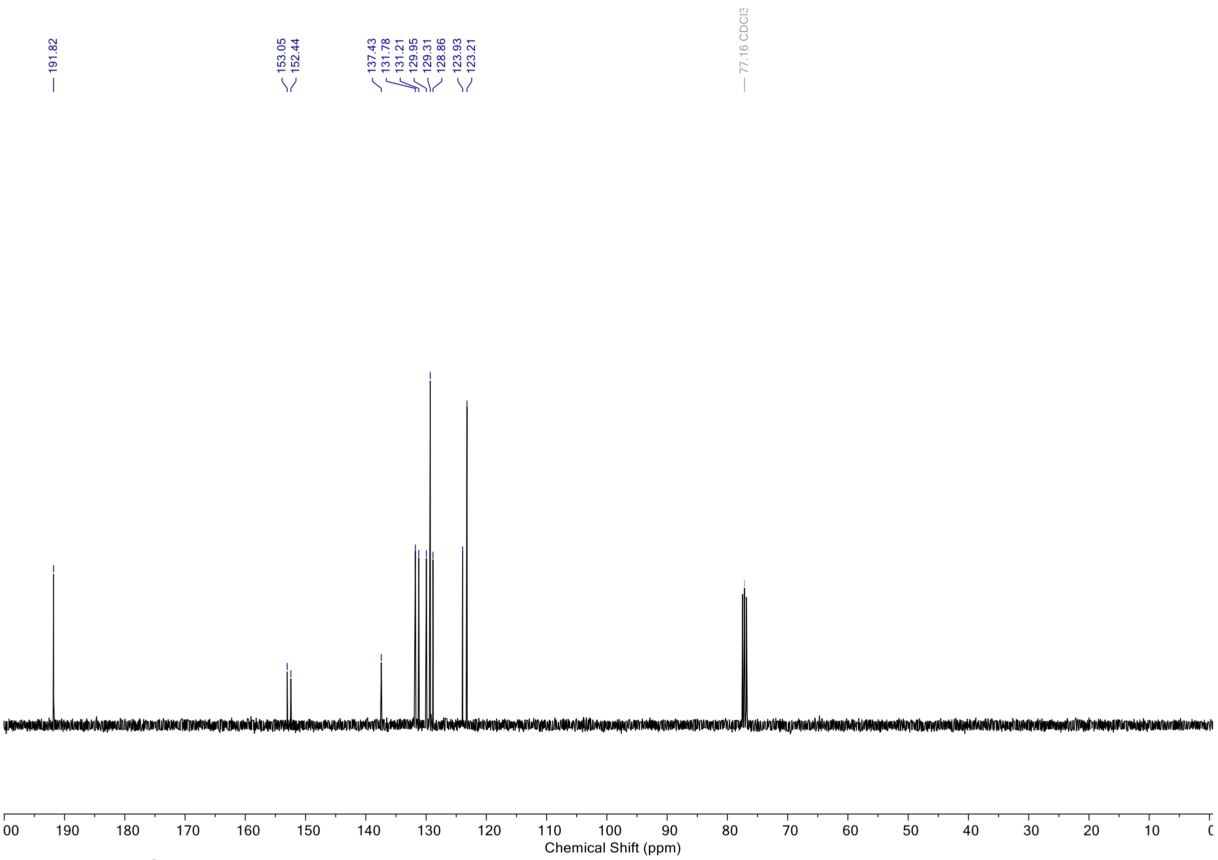

Figure S86: $^{13}C\{^{1}H\}$ **NMR** spectrum of **15** ($CDCl_3$).

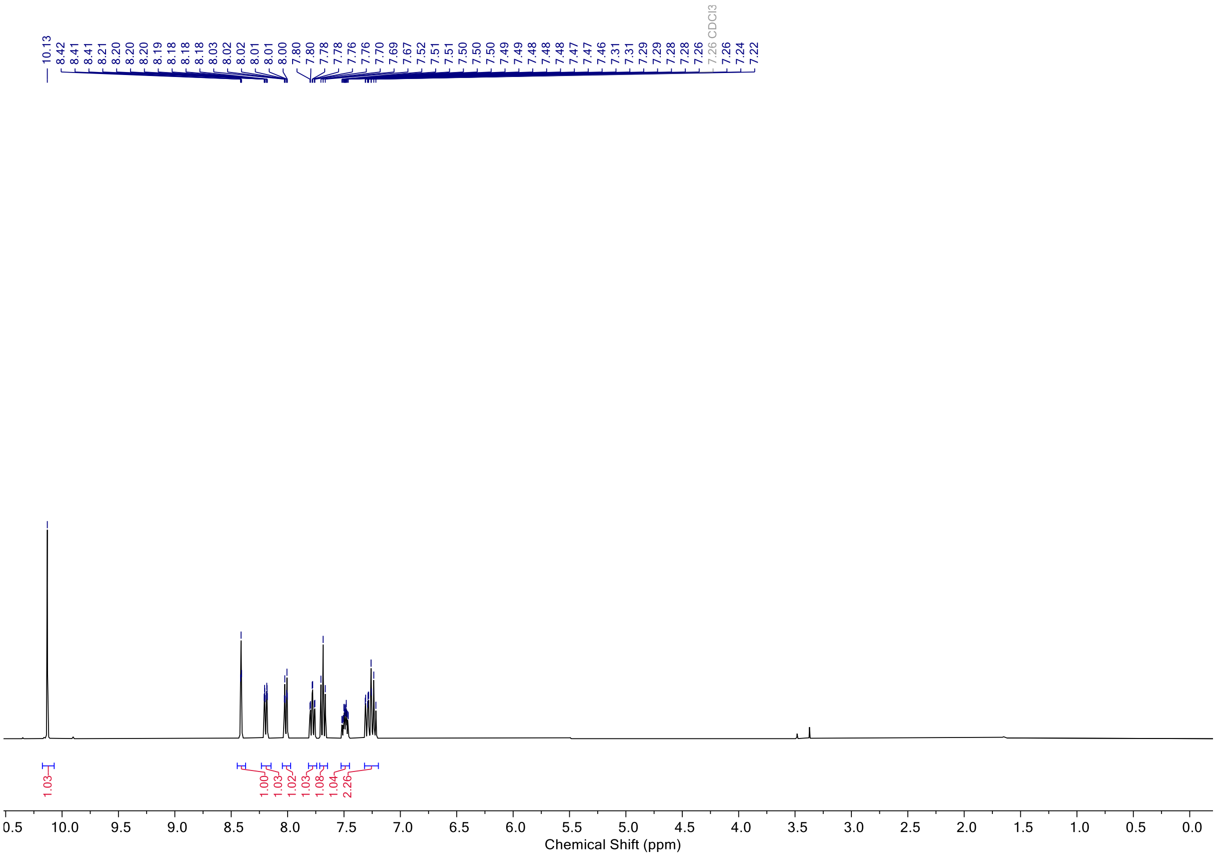

Figure S87: ^{1}H **NMR** spectrum of **16** ($CDCl_3$).

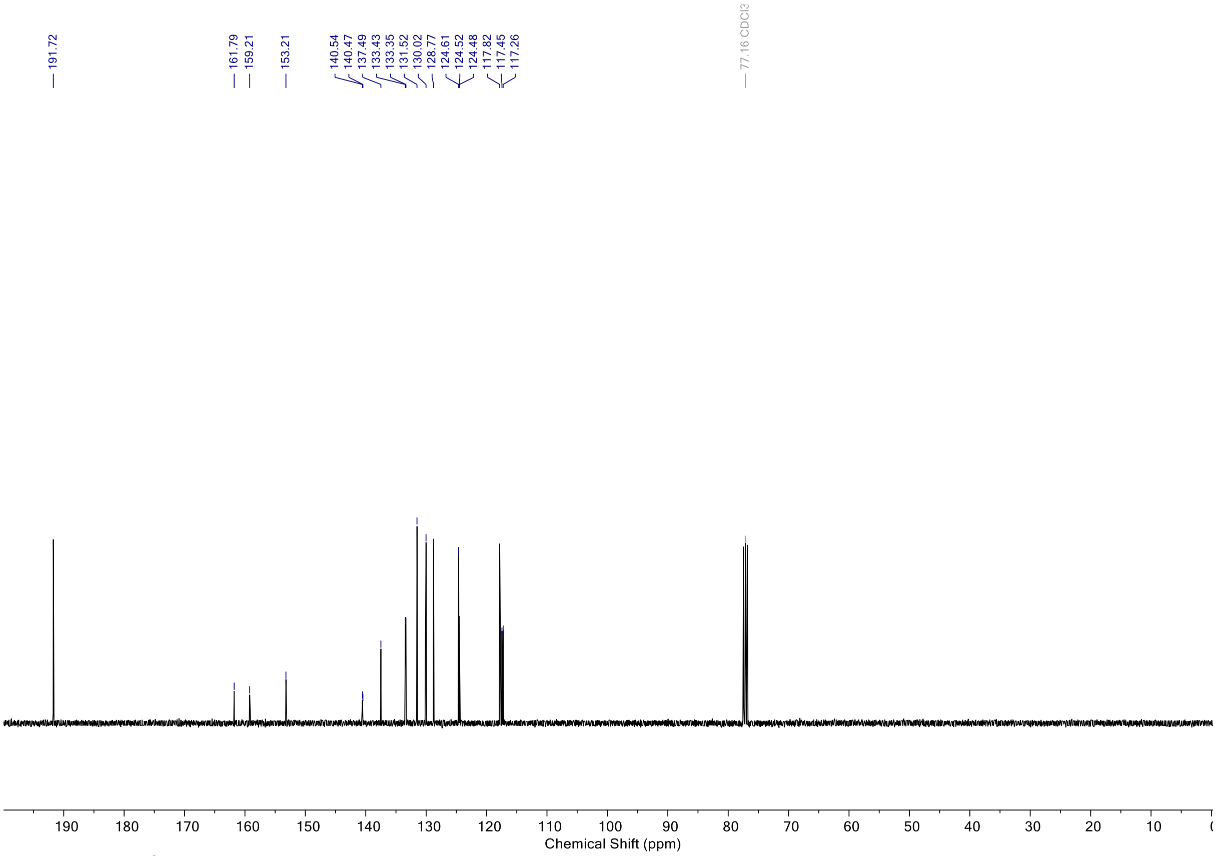

Figure S88: $^{13}C\{^{1}H\}$ NMR spectrum of **16** ($CDCl_3$).

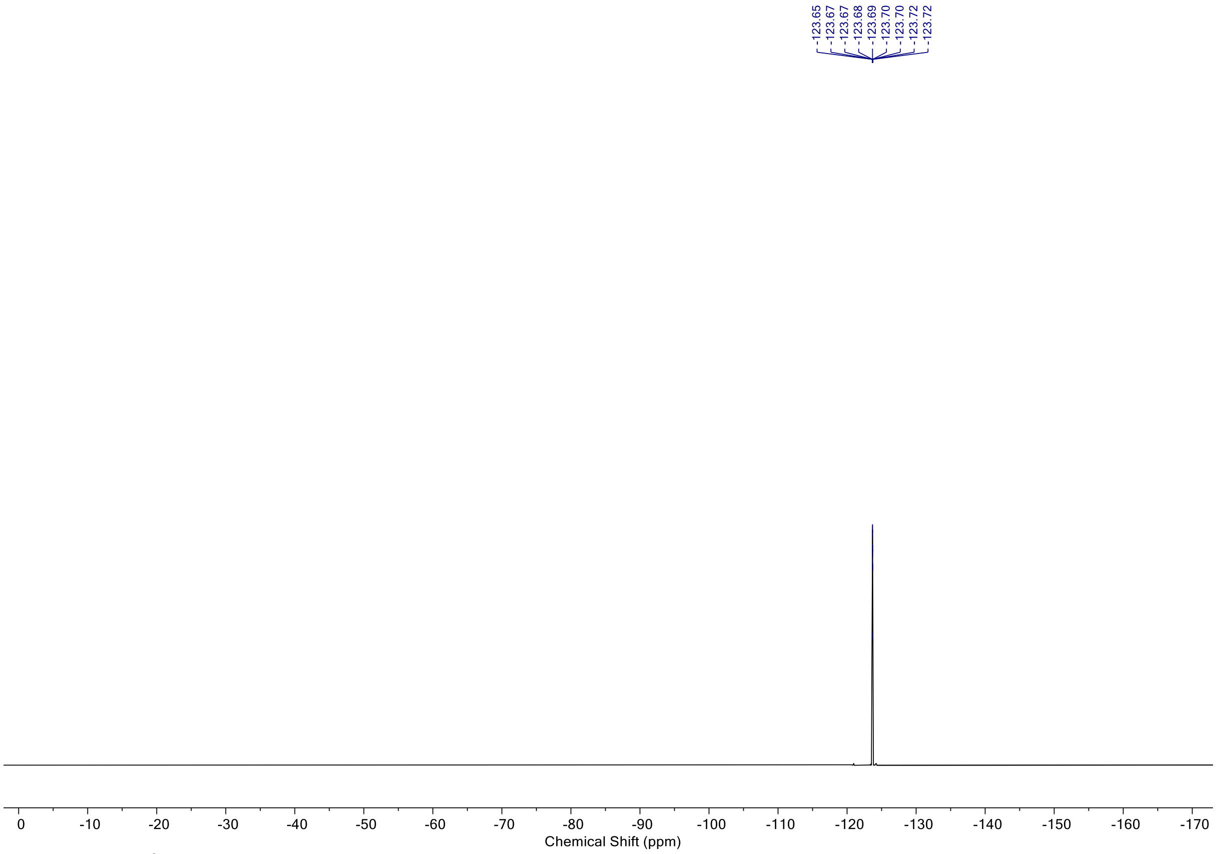

Figure S89: ^{19}F NMR spectrum of **16** ($CDCl_3$).

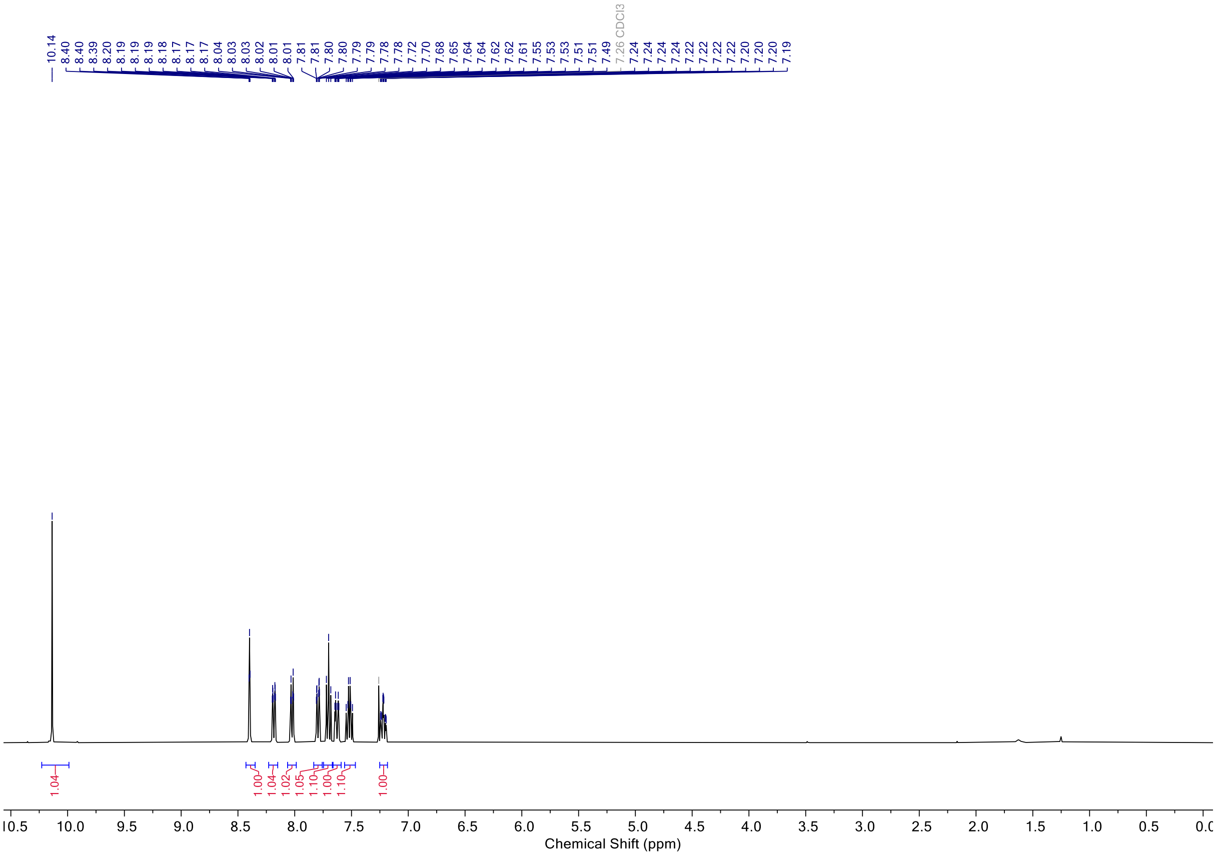

Figure S90: **¹H NMR** spectrum of **17** (CDCl₃).

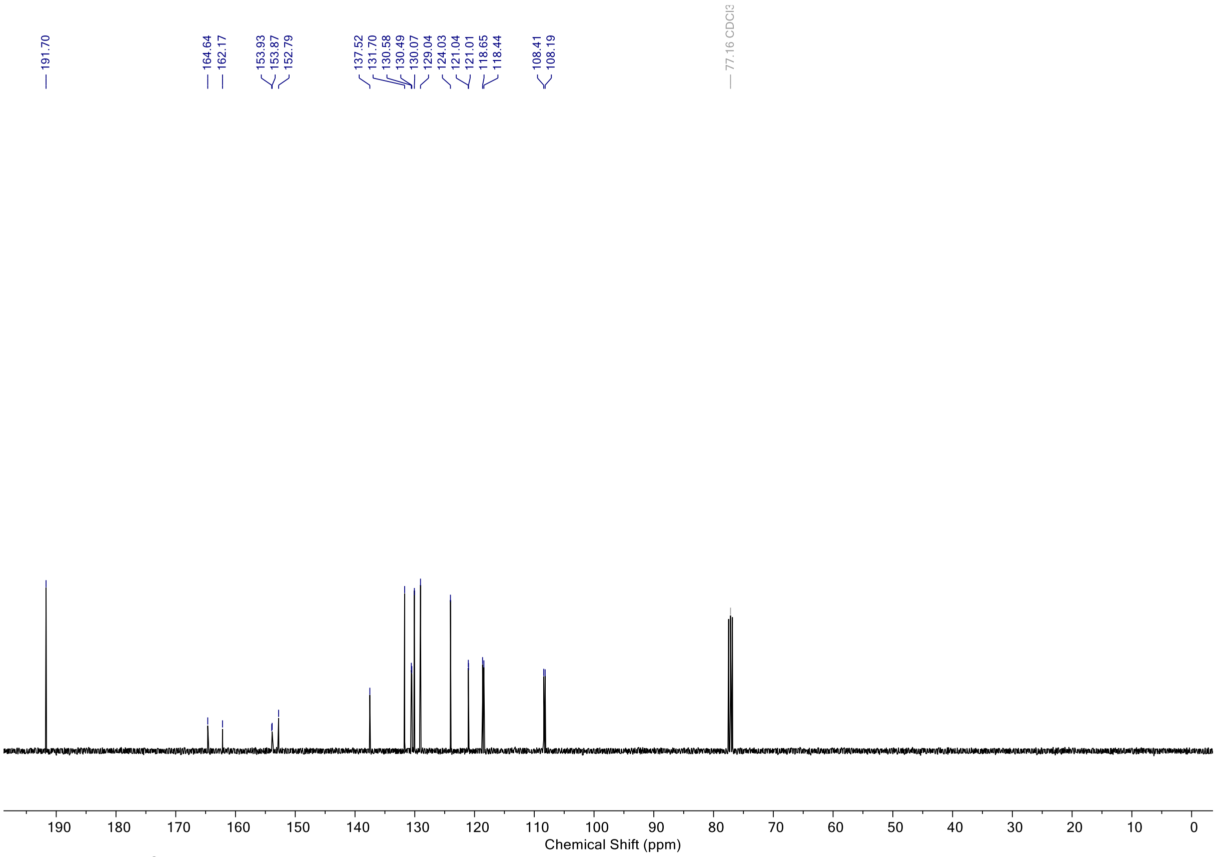

Figure S91: **¹³C{¹H} NMR** spectrum of **17** (CDCl₃).

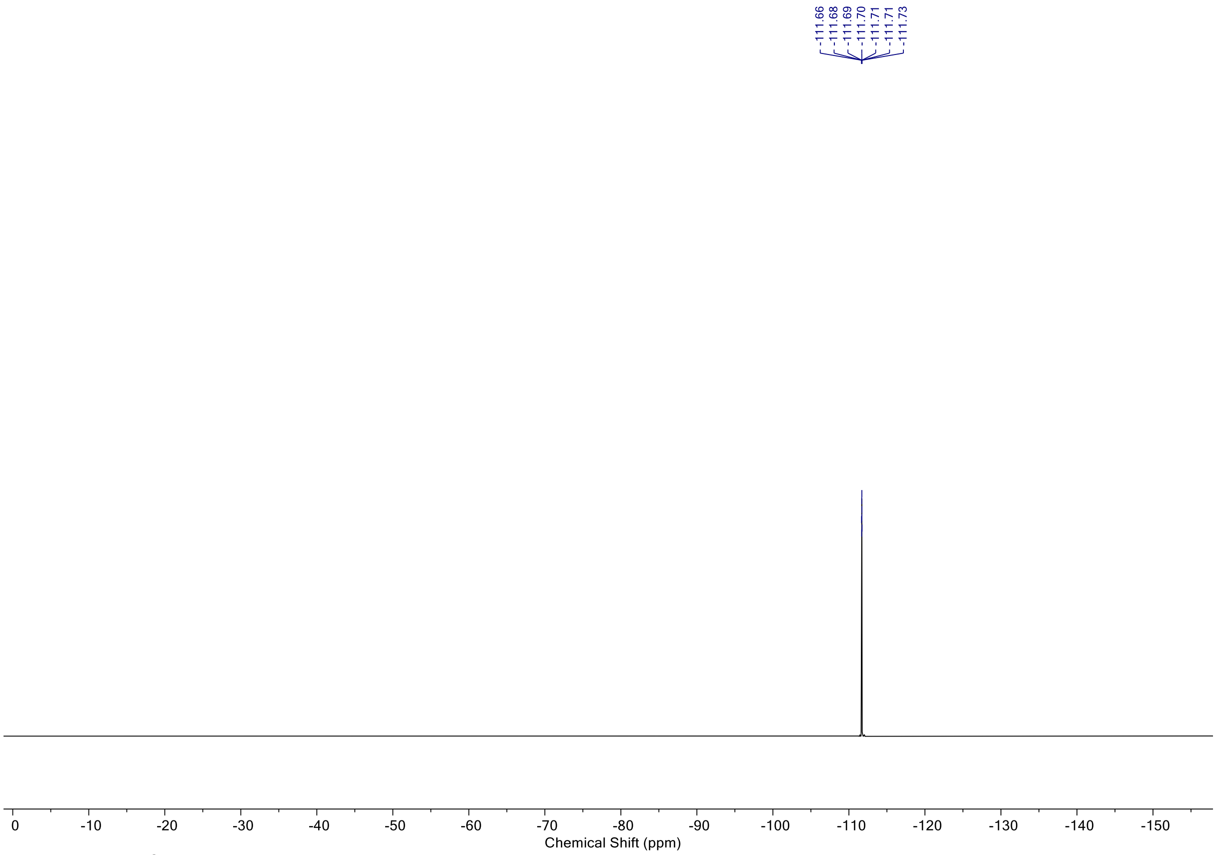

Figure S92: ^{19}F NMR spectrum of **17** ($CDCl_3$).

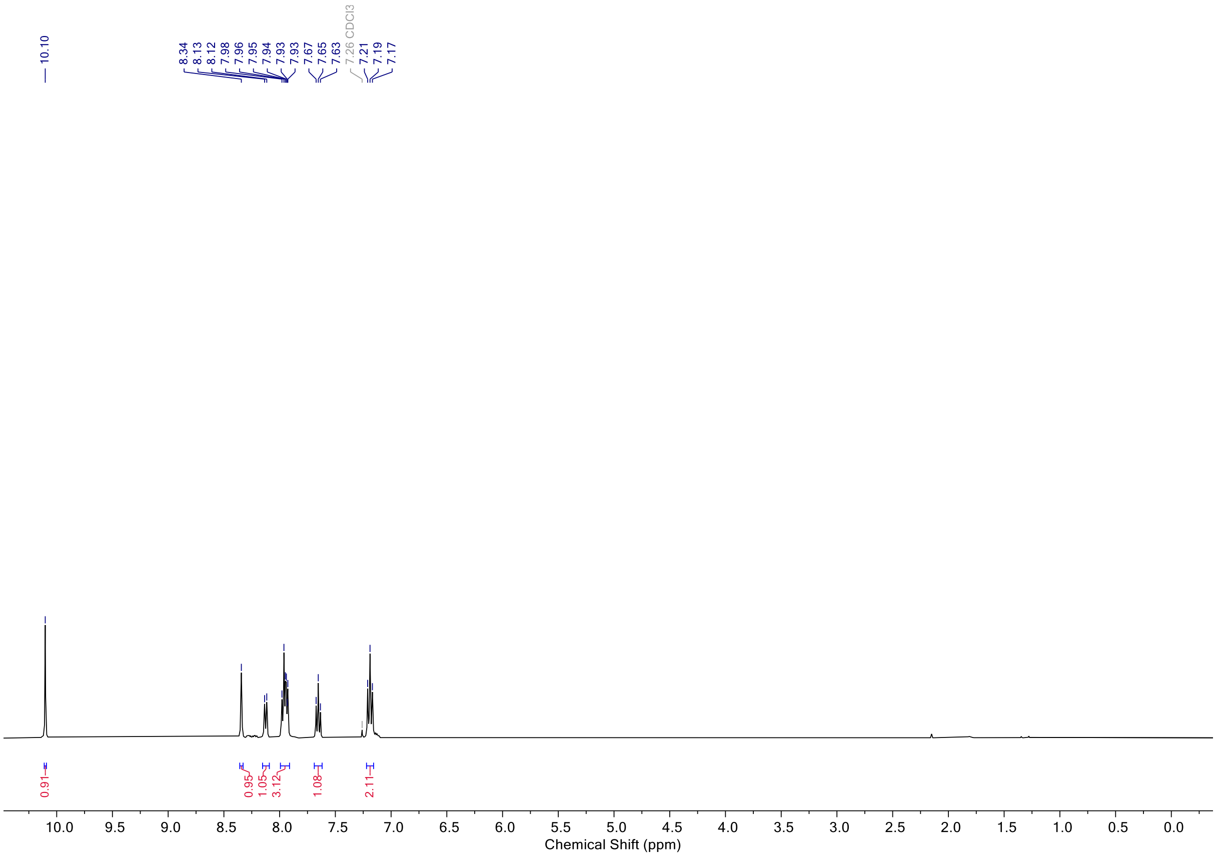

Figure S93: ^{1}H NMR spectrum of **18** ($CDCl_3$).

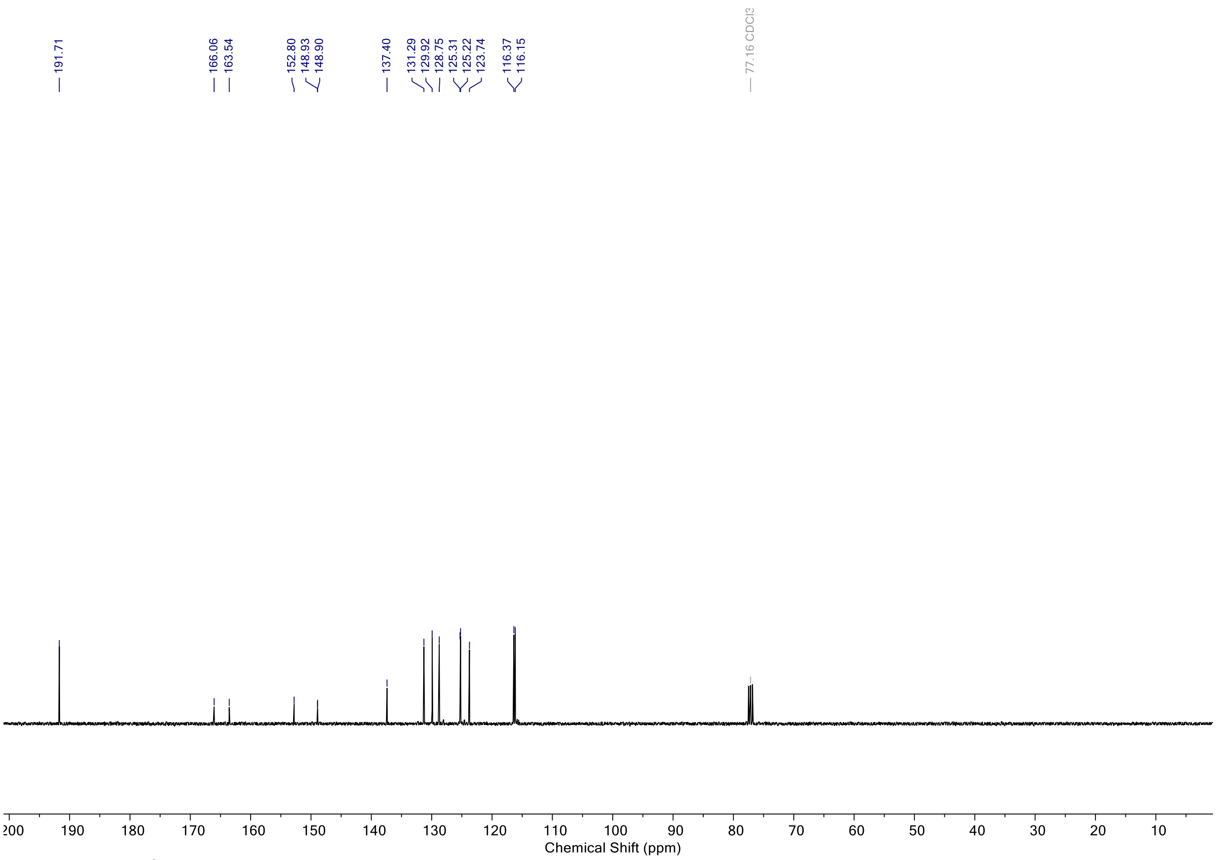

Figure S94: $^{13}C\{^{1}H\}$ NMR spectrum of **18** ($CDCl_3$).

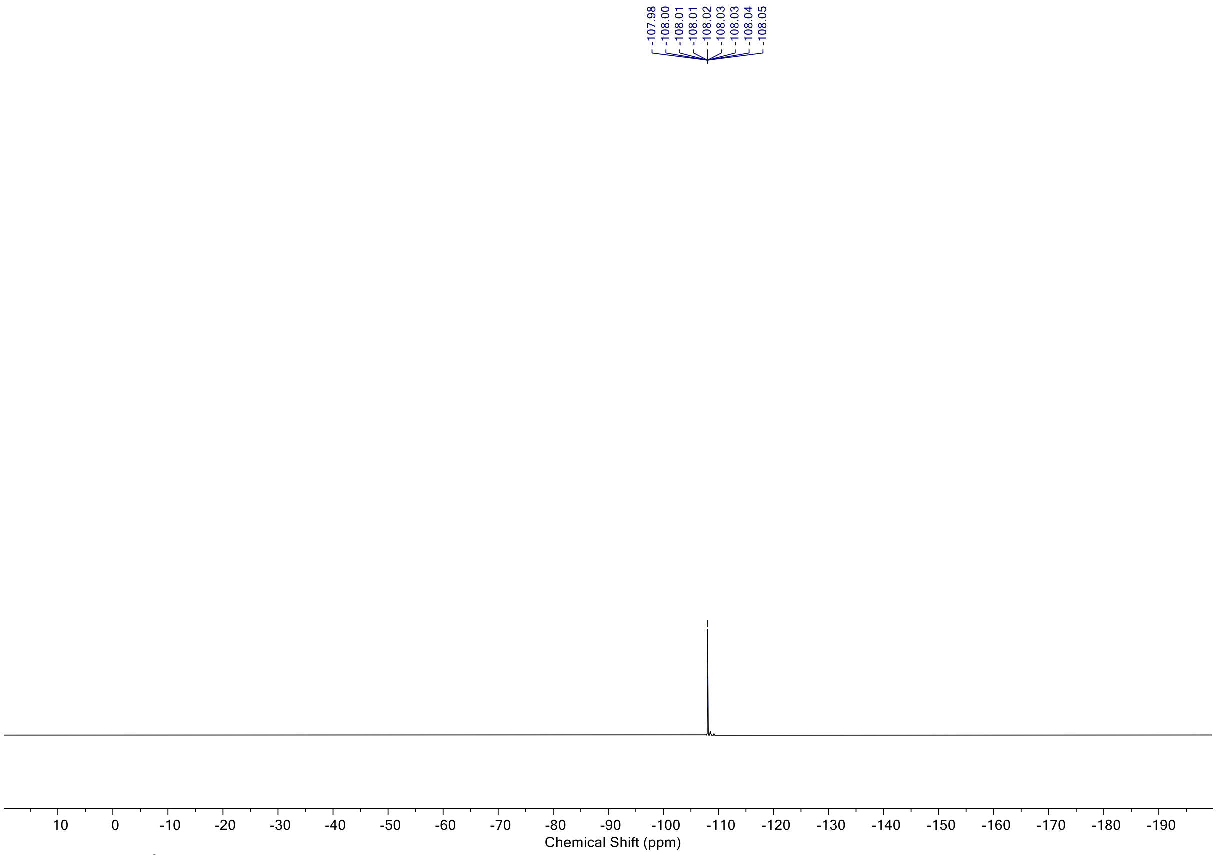

Figure S95: ^{19}F NMR spectrum of **18** ($CDCl_3$).

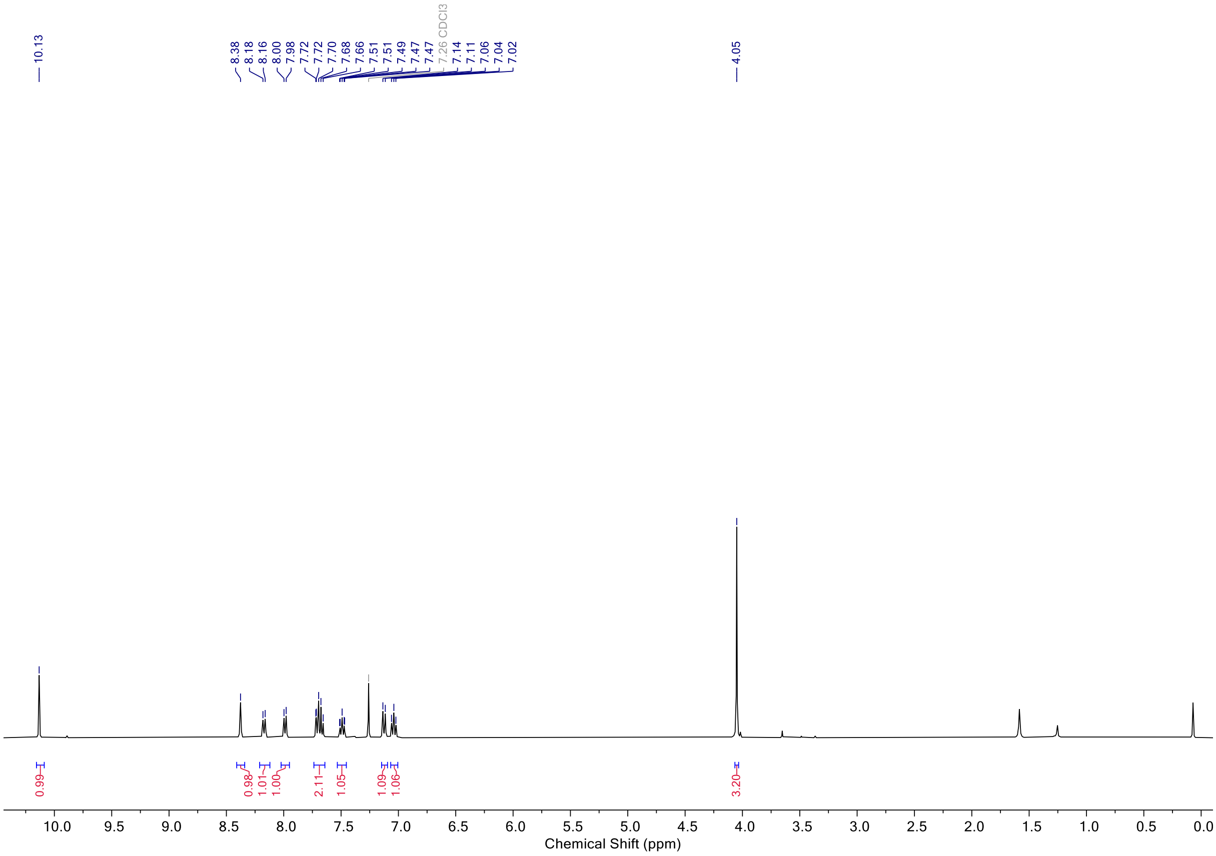

Figure S96: ^{1}**H NMR** spectrum of **19** ($CDCl_3$).

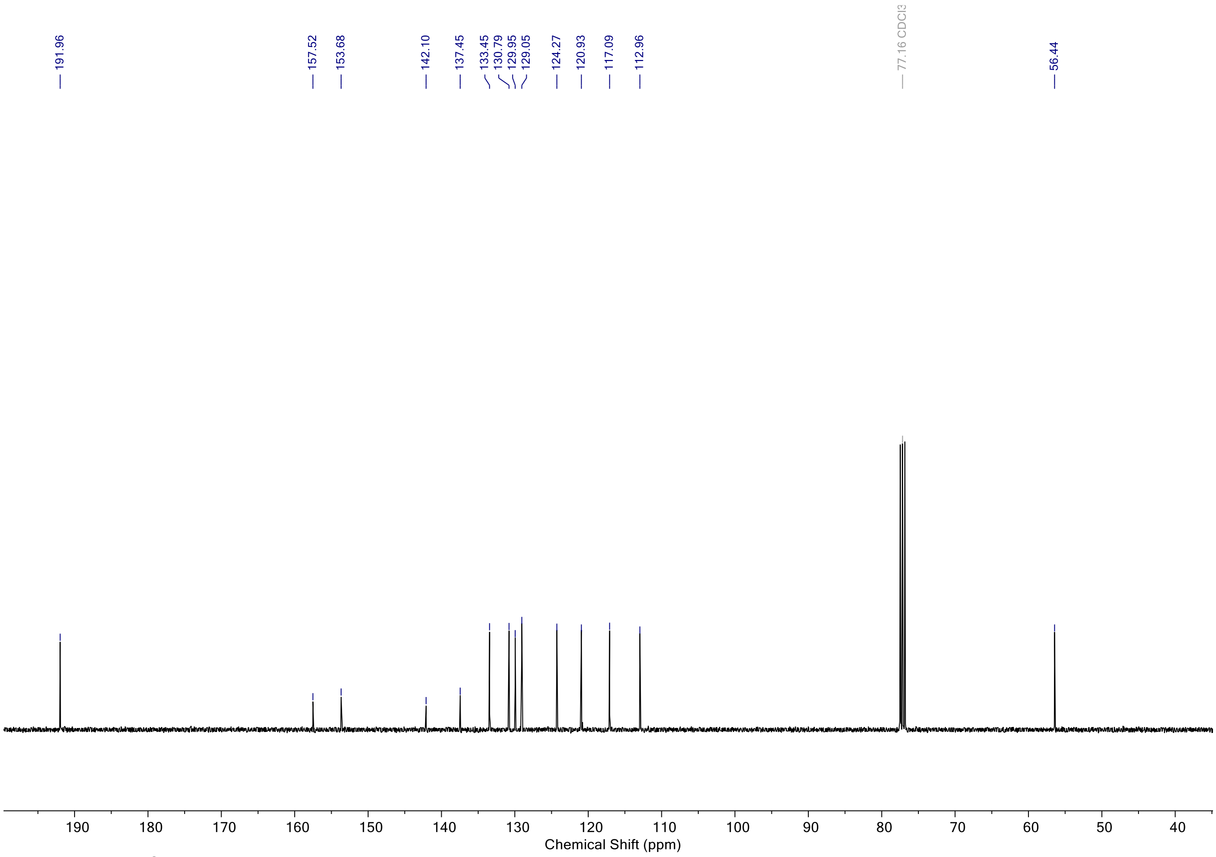

Figure S97: ^{13}**C{**^{1}**H} NMR** spectrum of **19** ($CDCl_3$).

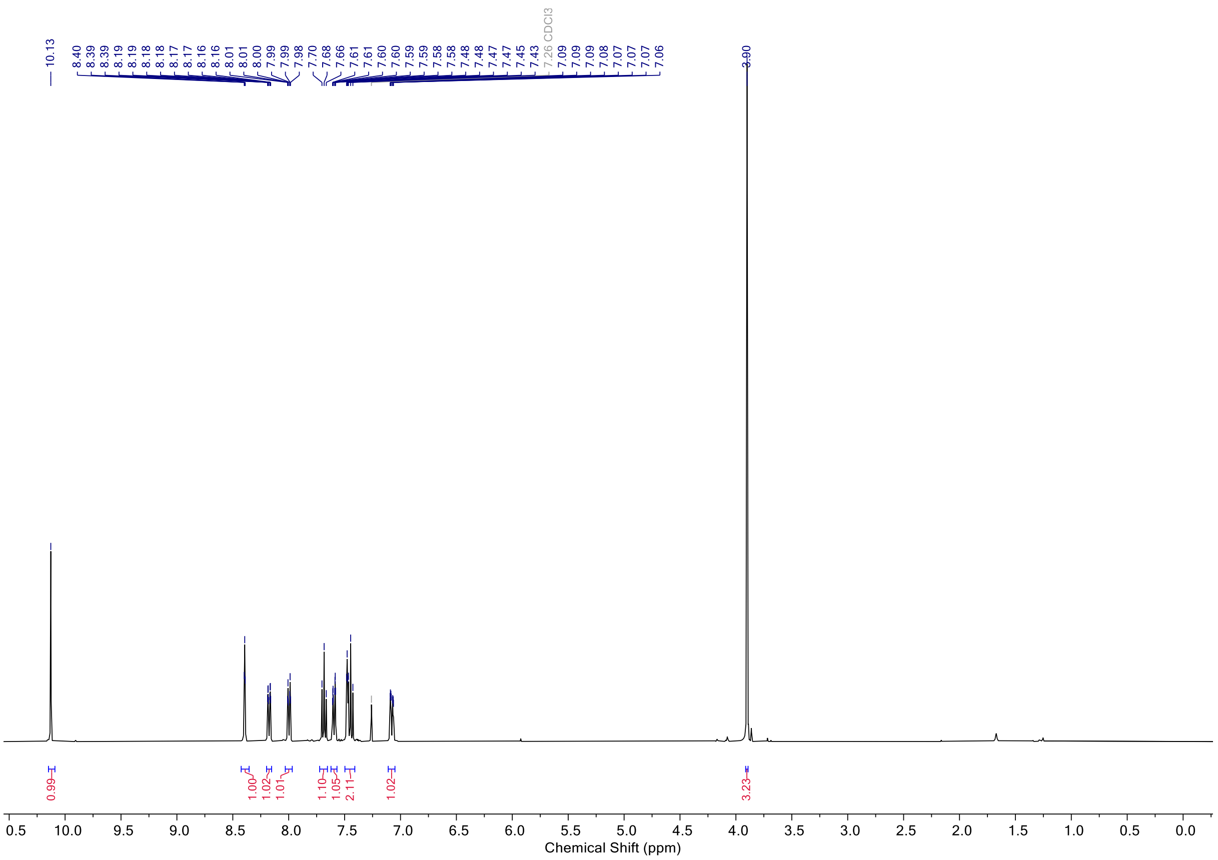

Figure S98: **¹H NMR** spectrum of **20** ($CDCl_3$).

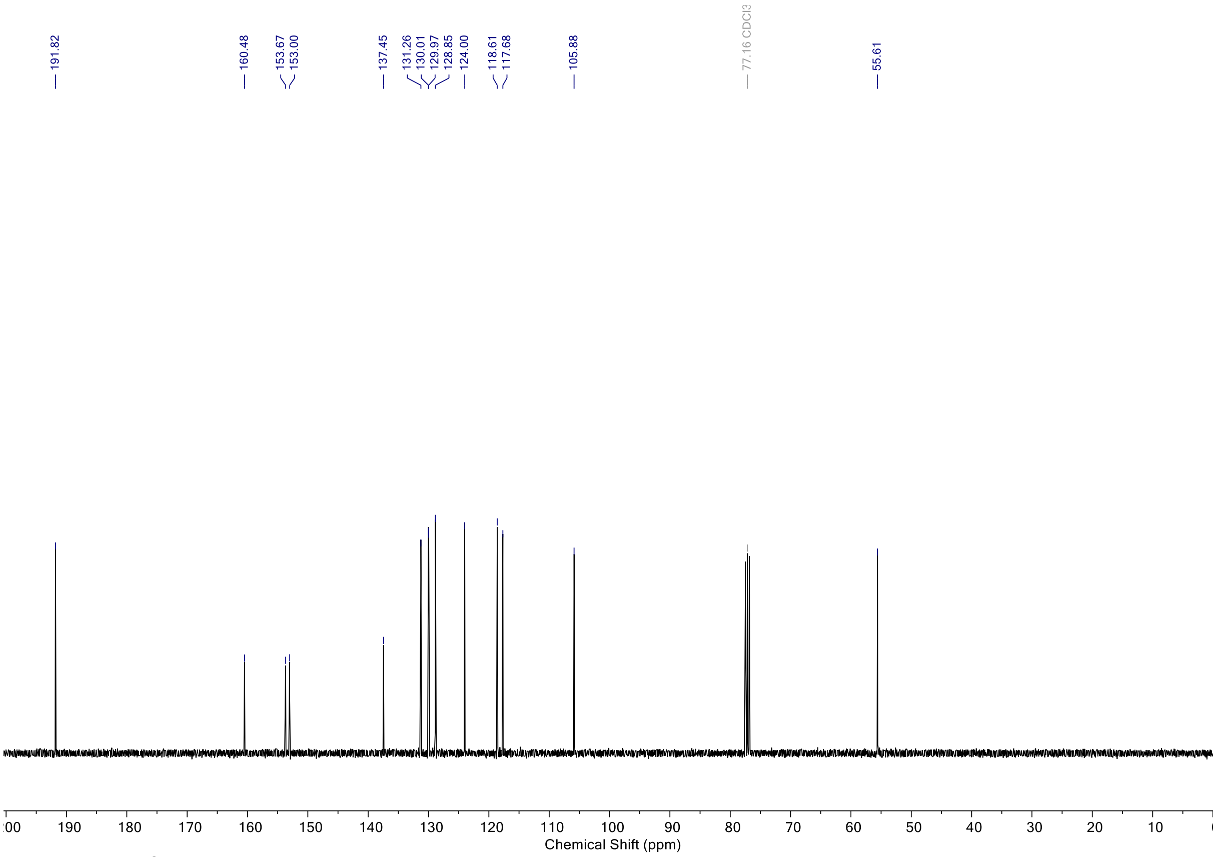

Figure S99: **^{13}C{^{1}H} NMR** spectrum of **20** ($CDCl_3$).

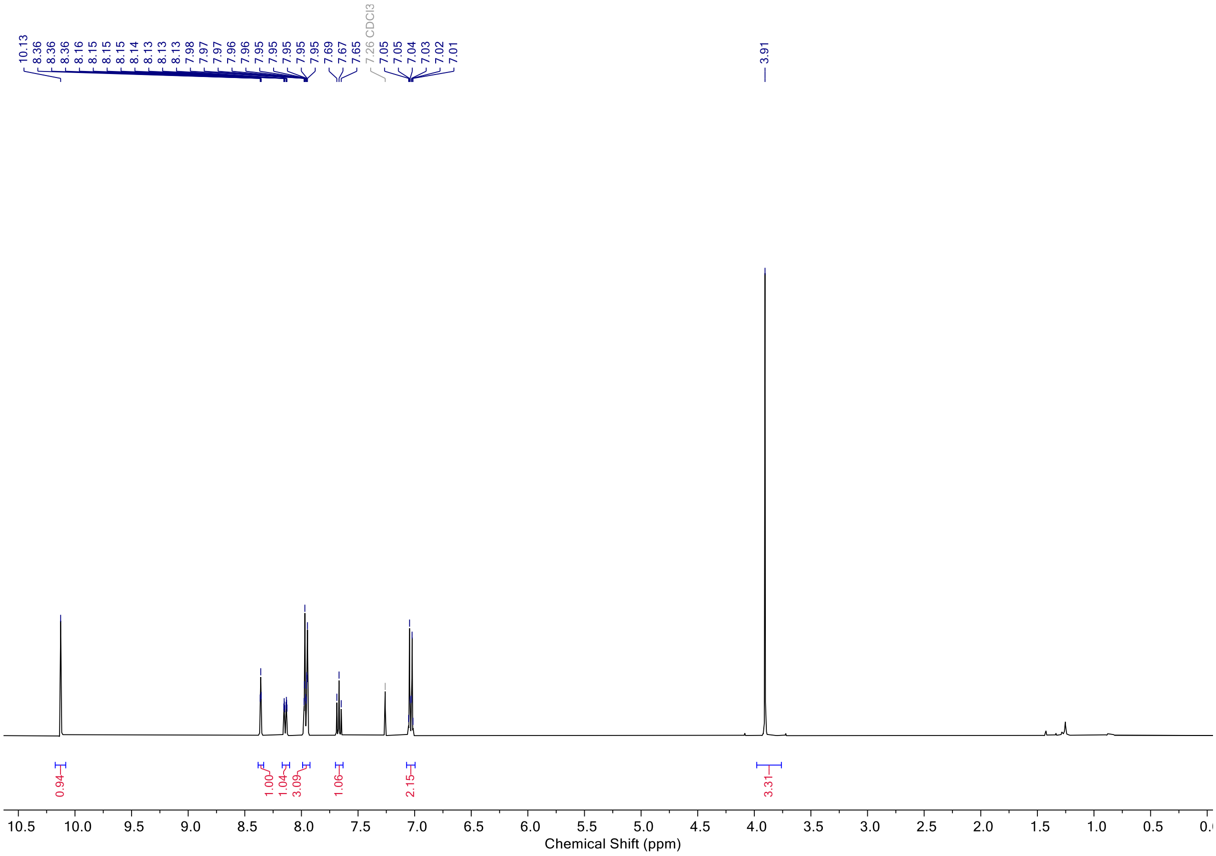

Figure S100: ^{1}H NMR spectrum of **21** ($CDCl_3$).

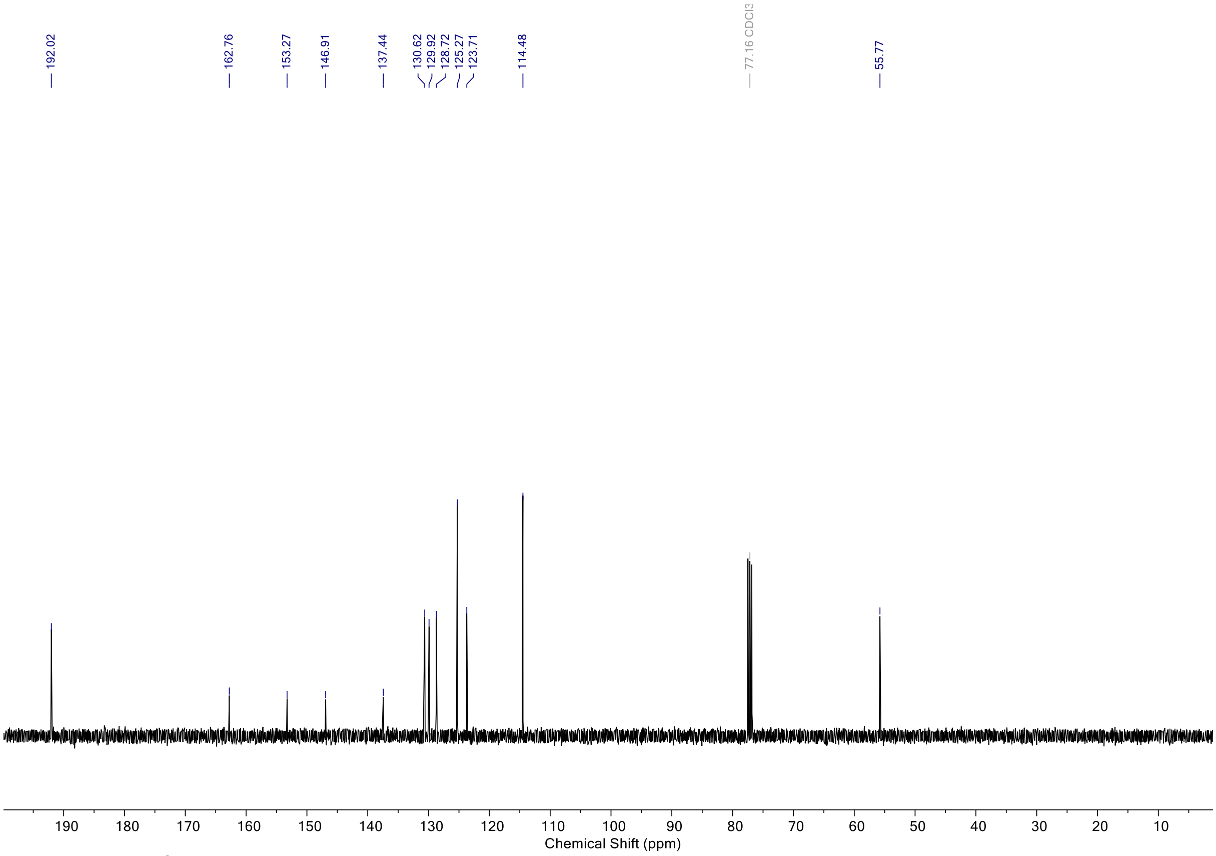

Figure S101: ^{13}C{^{1}H} NMR spectrum of **21** ($CDCl_3$).

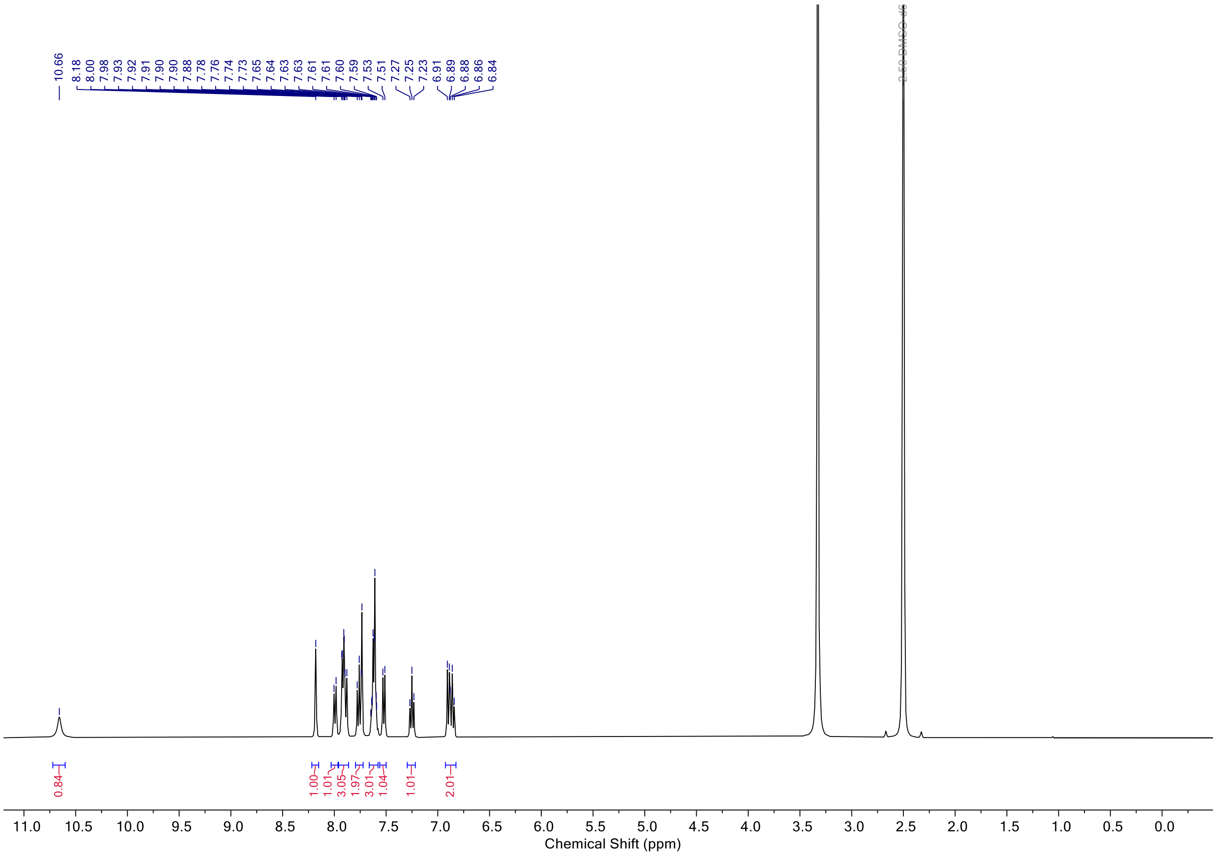

Figure S102: ^{1}H NMR spectrum of ***EE*-1** ($(CD_3)_2SO$).

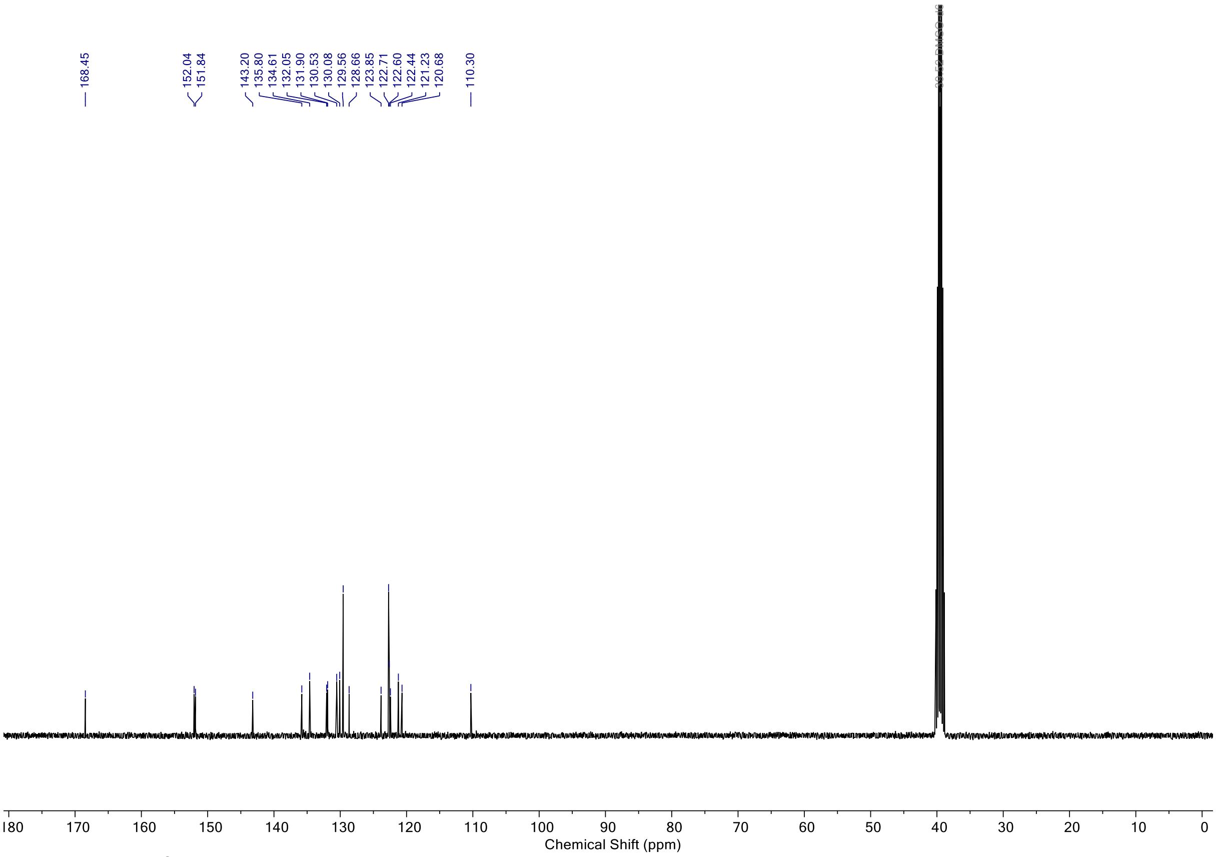

Figure S103: ^{13}C{^{1}H} NMR spectrum of ***EE*-1** ($(CD_3)_2SO$).

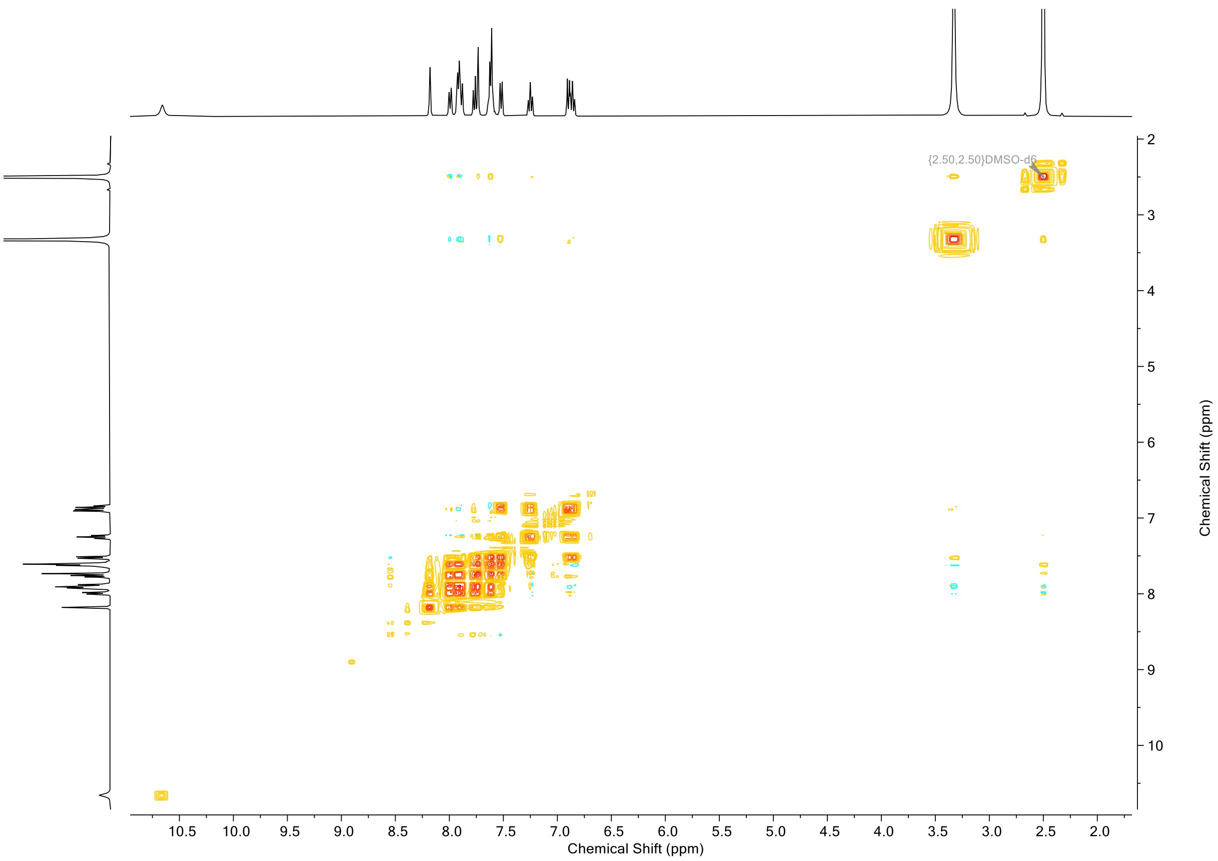

Figure S104: COSY spectrum of ***EE*-1** (($(CD_3)_2SO$).

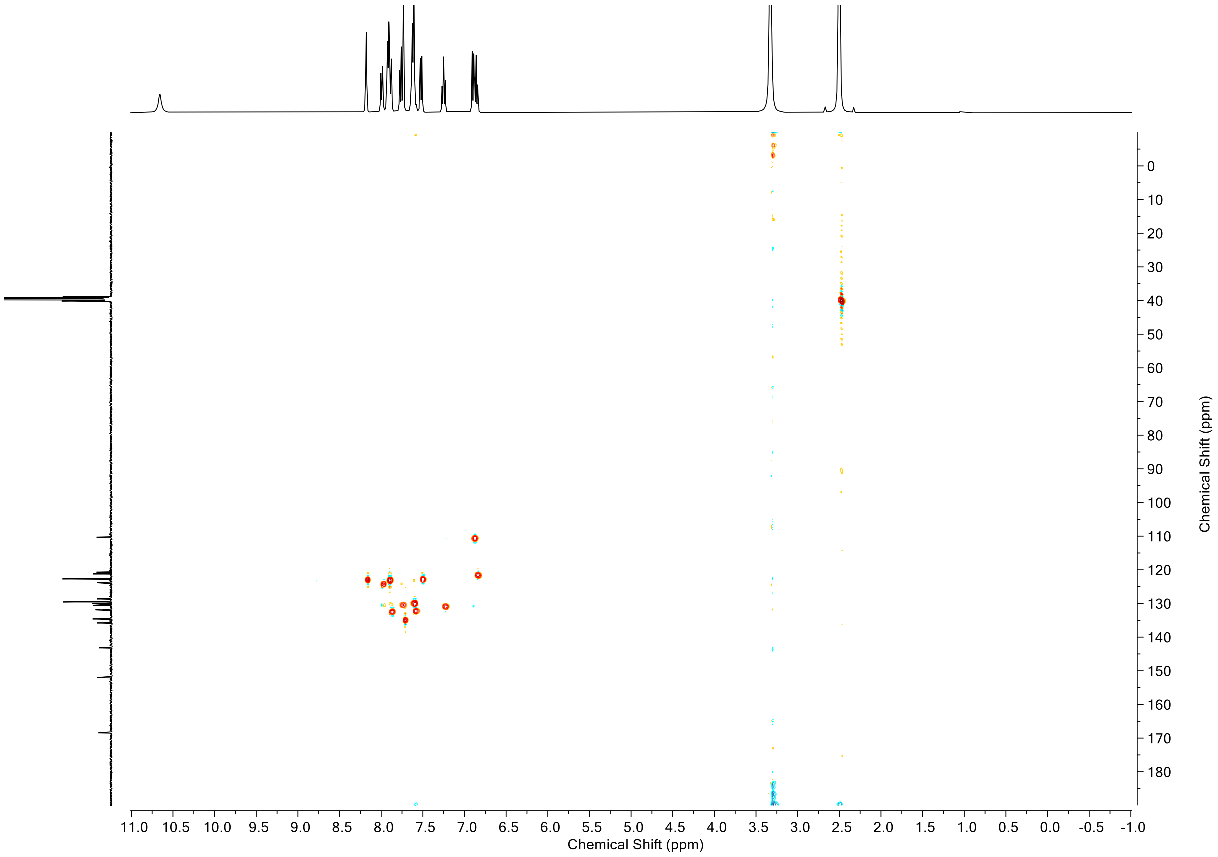

Figure S105: HSQC spectrum of ***EE*-1** (($(CD_3)_2SO$).

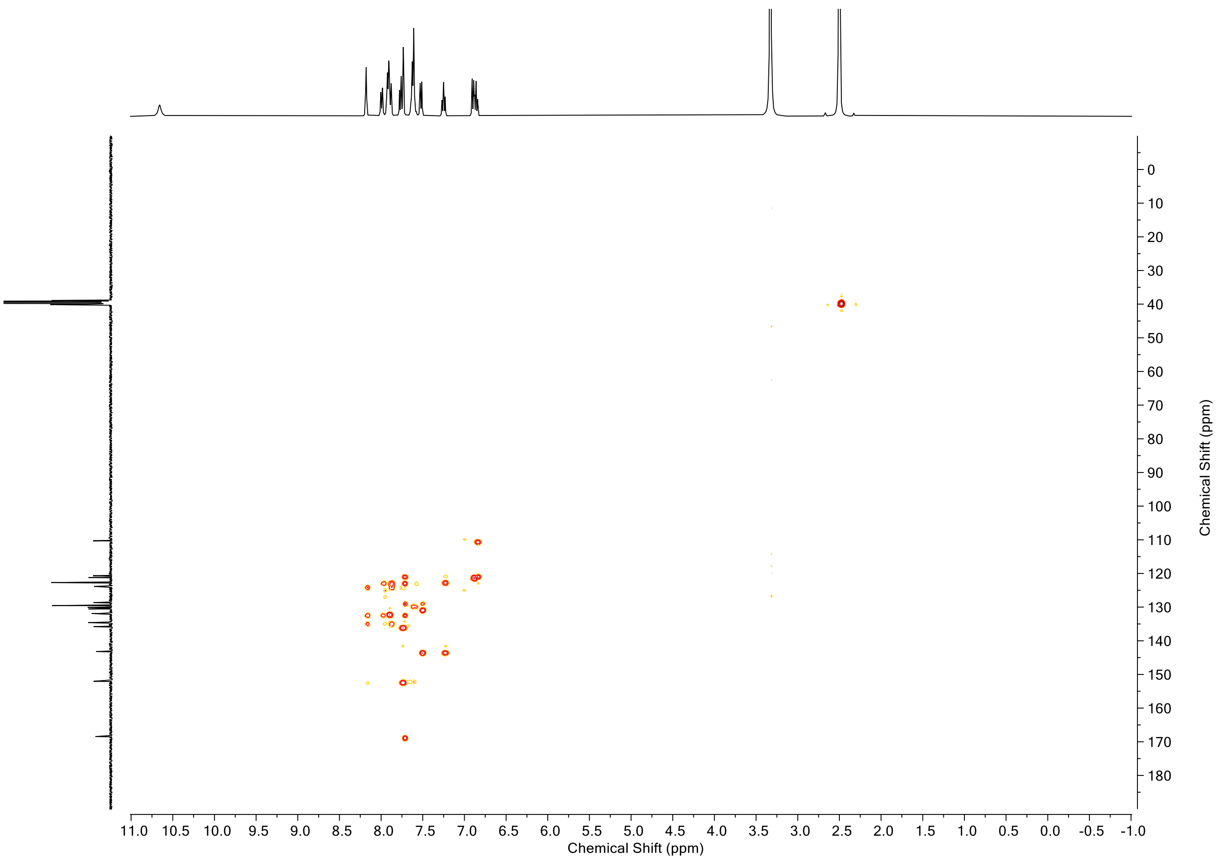

Figure S106: HMBC spectrum of ***EE*-1** ($(CD_3)_2SO$).

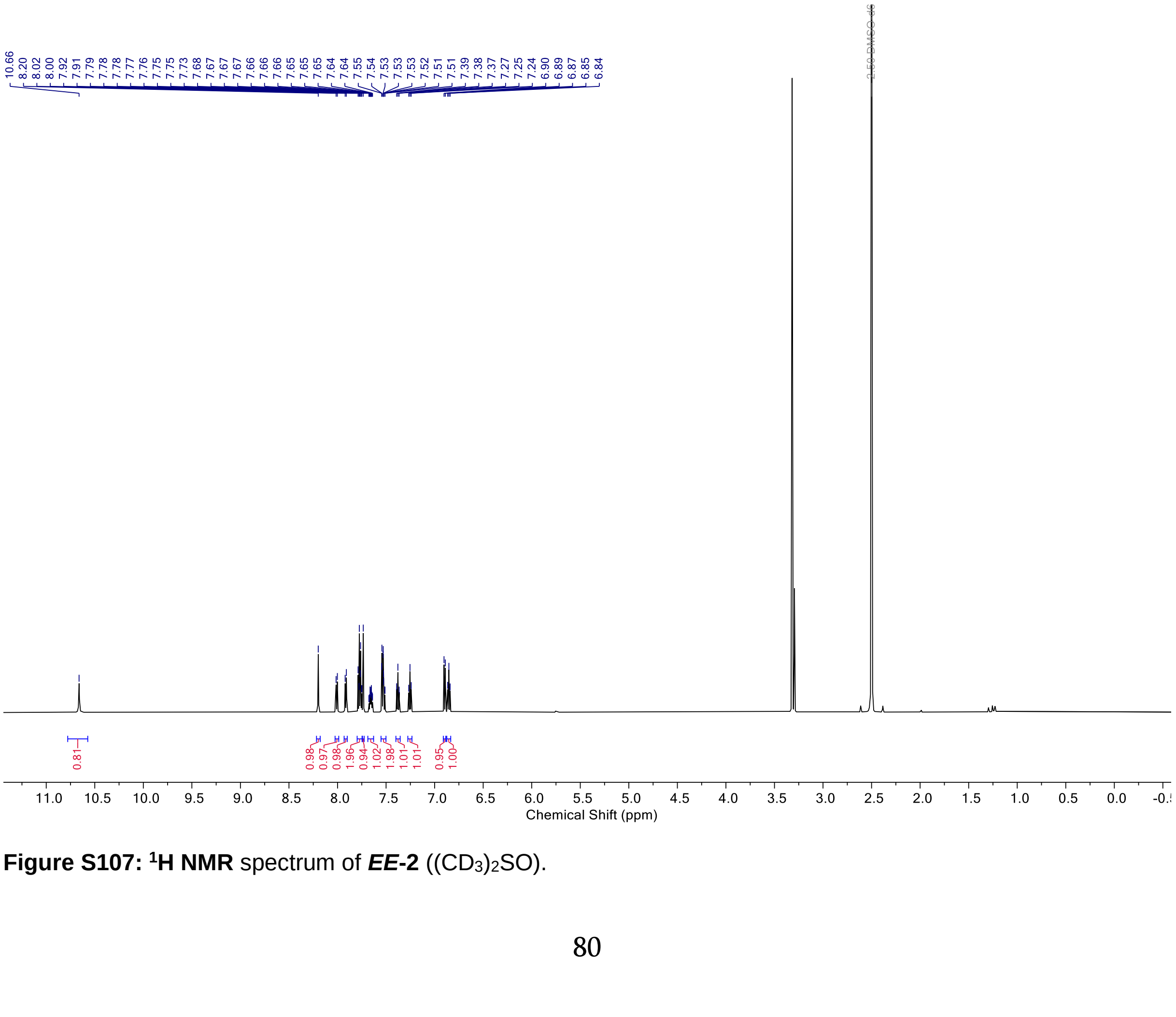

Figure S107: ^{1}H NMR spectrum of ***EE*-2** ($(CD_3)_2SO$).

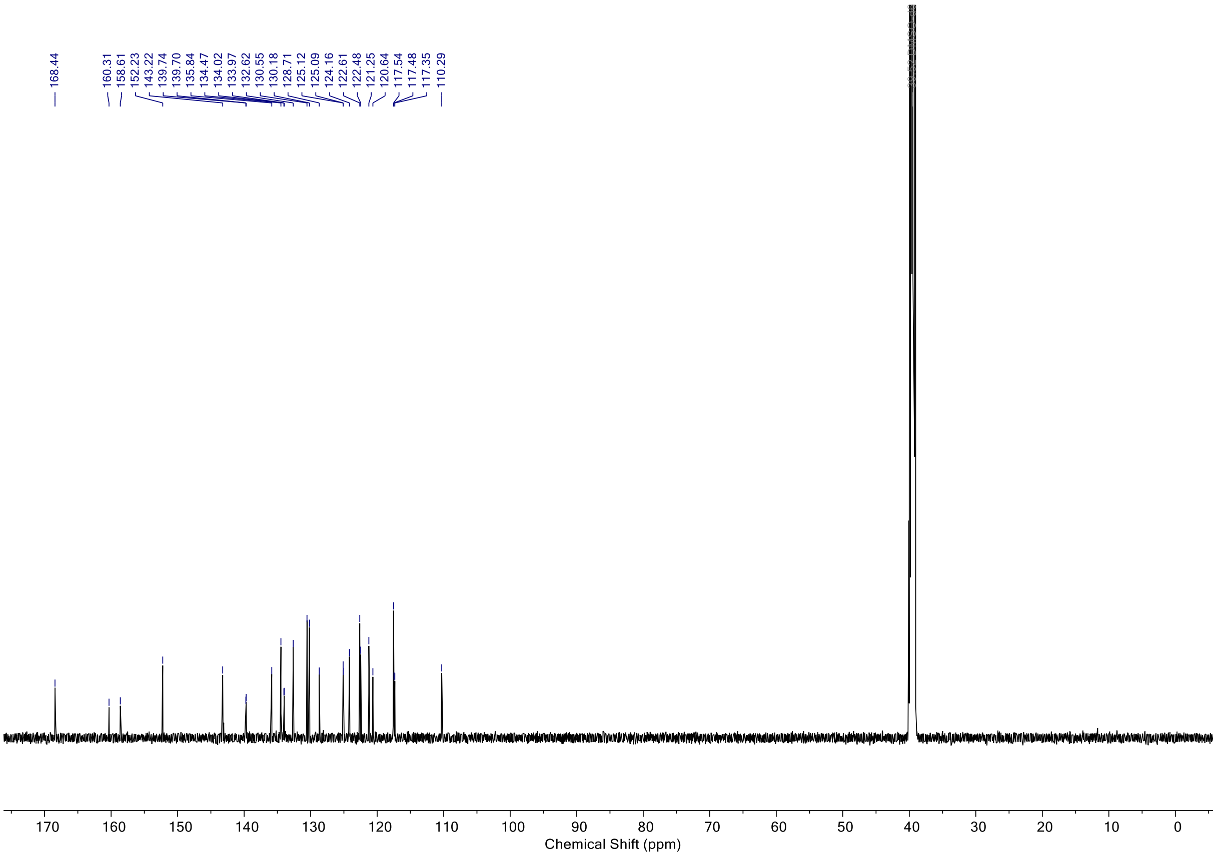

Figure S108: $^{13}C\{^1H\}$ NMR spectrum of ***EE*-2** ($(CD_3)_2SO$).

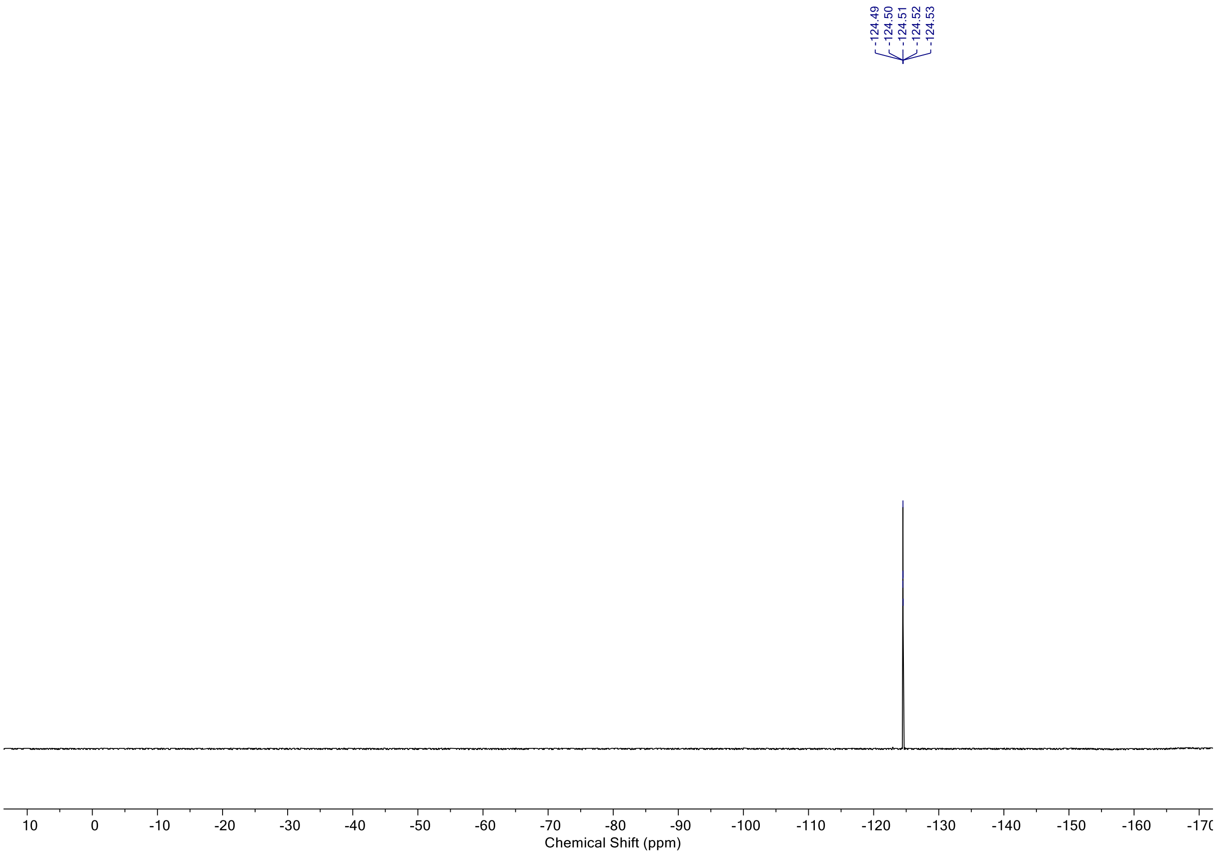

Figure S109: ^{19}F NMR spectrum of ***EE*-2** ($(CD_3)_2SO$).

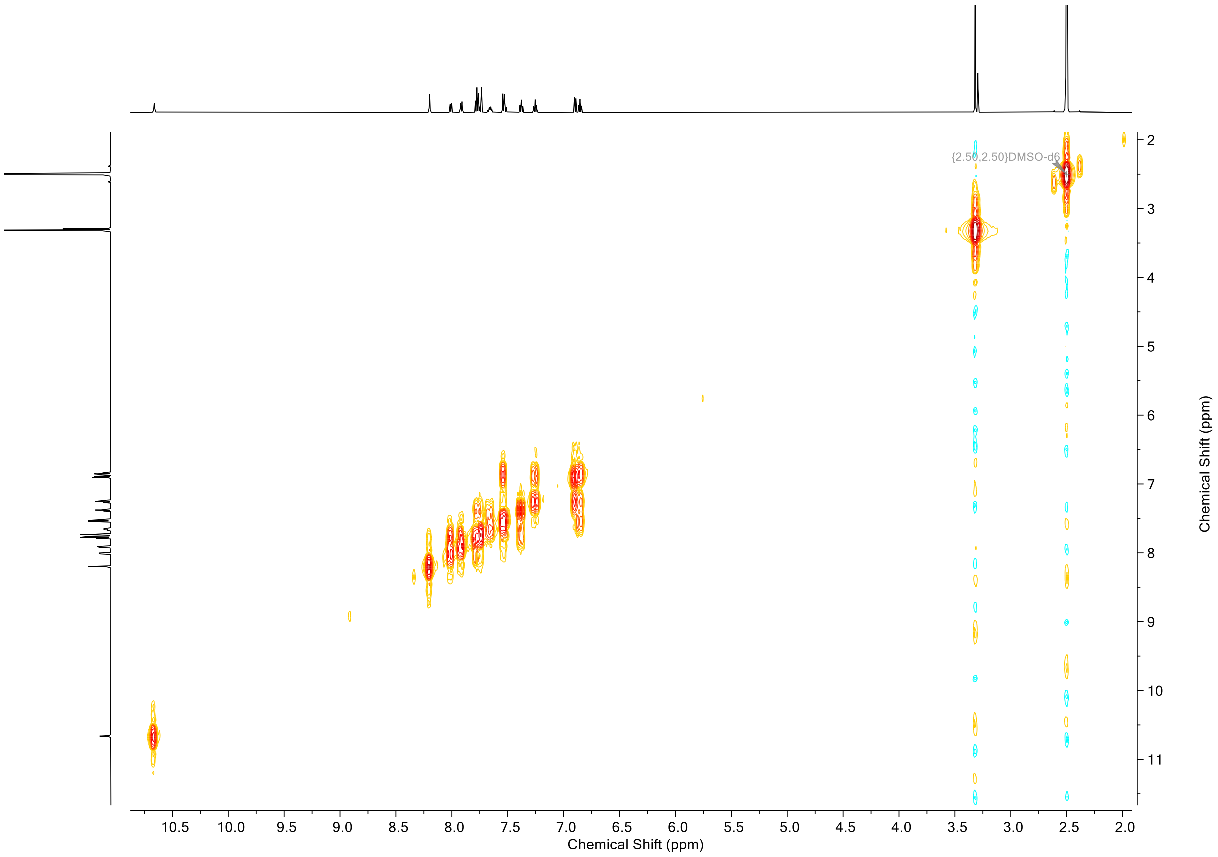

Figure S110: COSY spectrum of ***EE*-2** ($(CD_3)_2SO$).

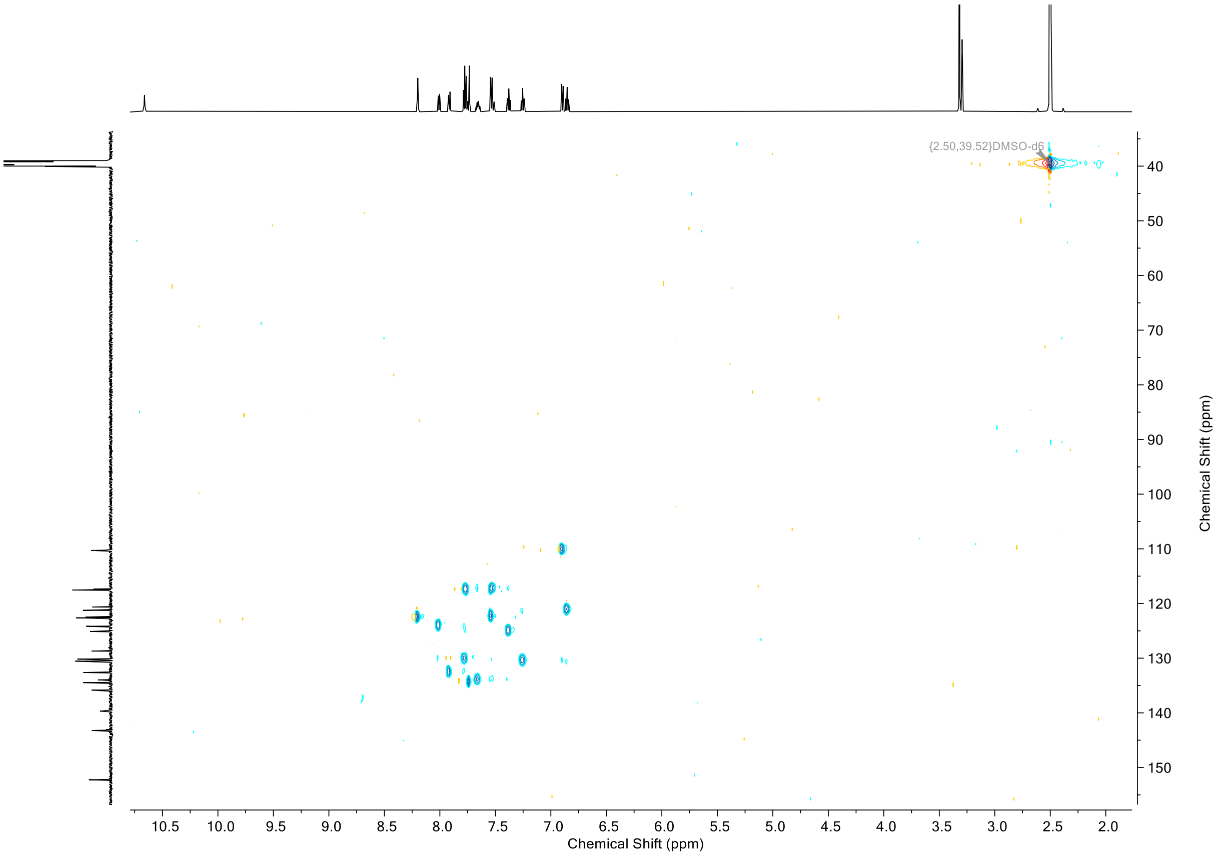

Figure S111: HSQC spectrum of ***EE*-2** ($(CD_3)_2SO$).

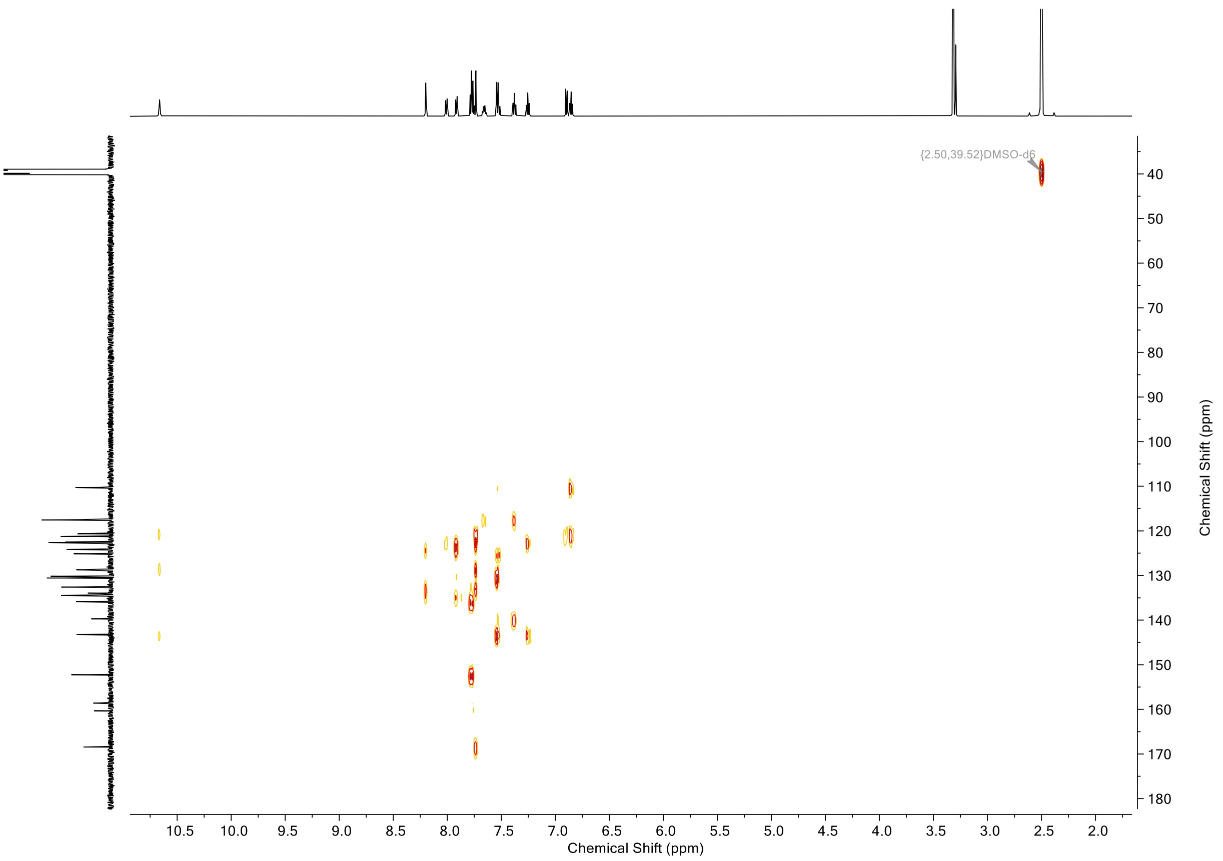

Figure S112: HMBC spectrum of ***EE*-2** ($(CD_3)_2SO$).

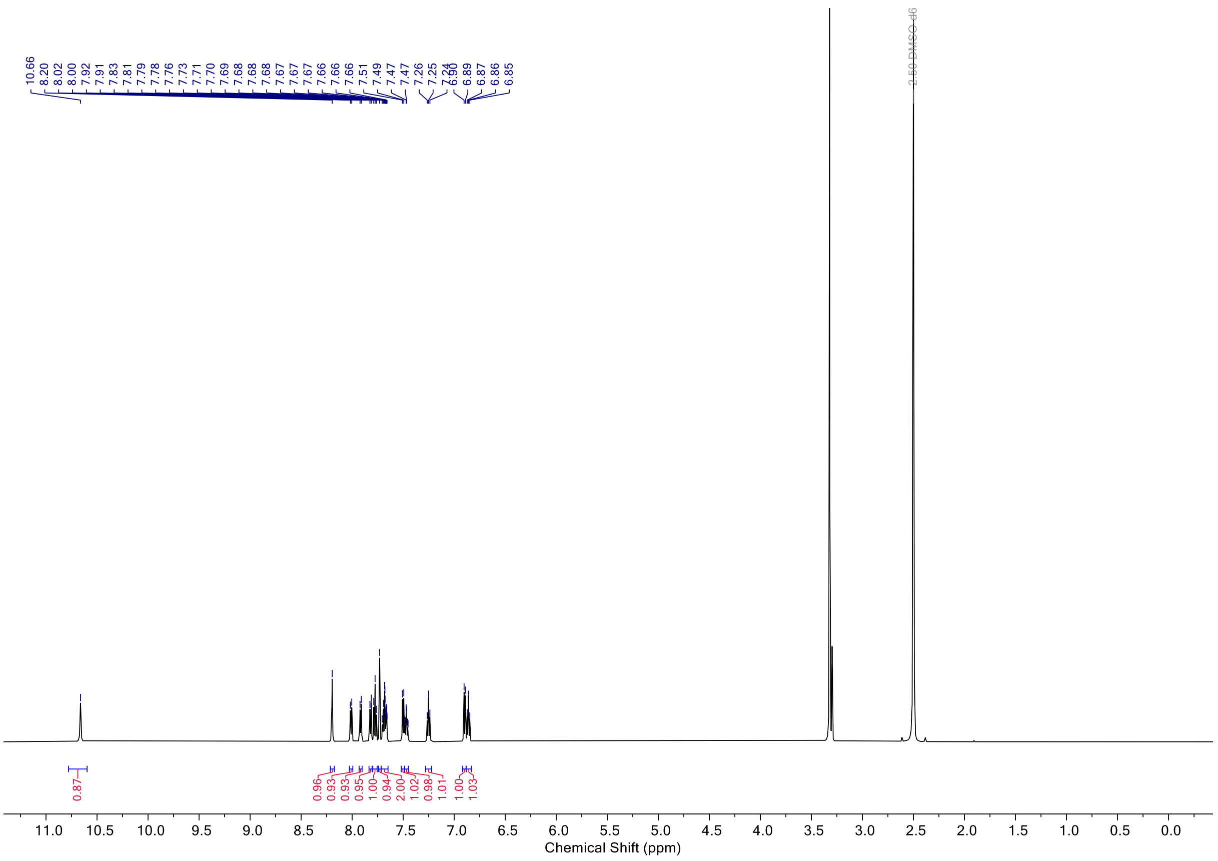

Figure S113: ^{1}H NMR spectrum of ***EE*-3** ($(CD_3)_2SO$).

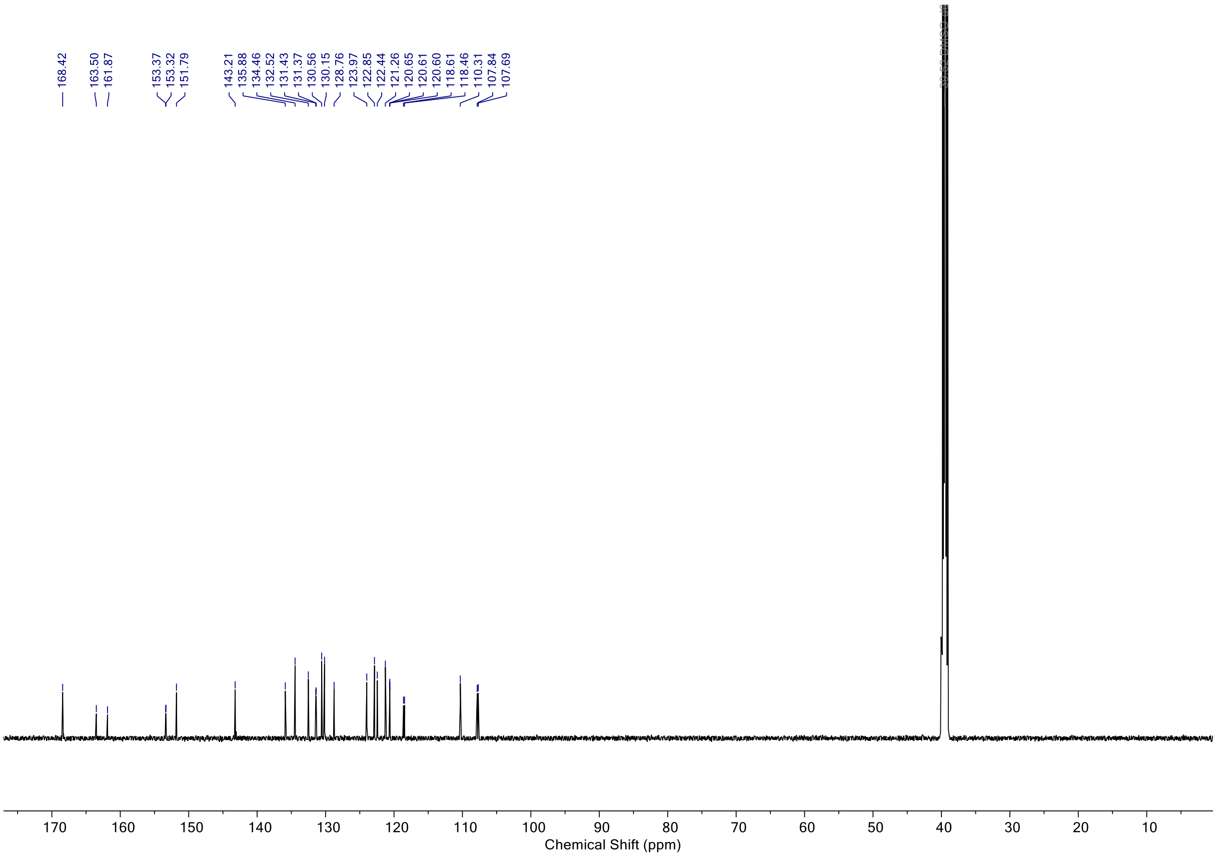

Figure S114: ^{13}C{^{1}H} NMR spectrum of ***EE*-3** ($(CD_3)_2SO$).

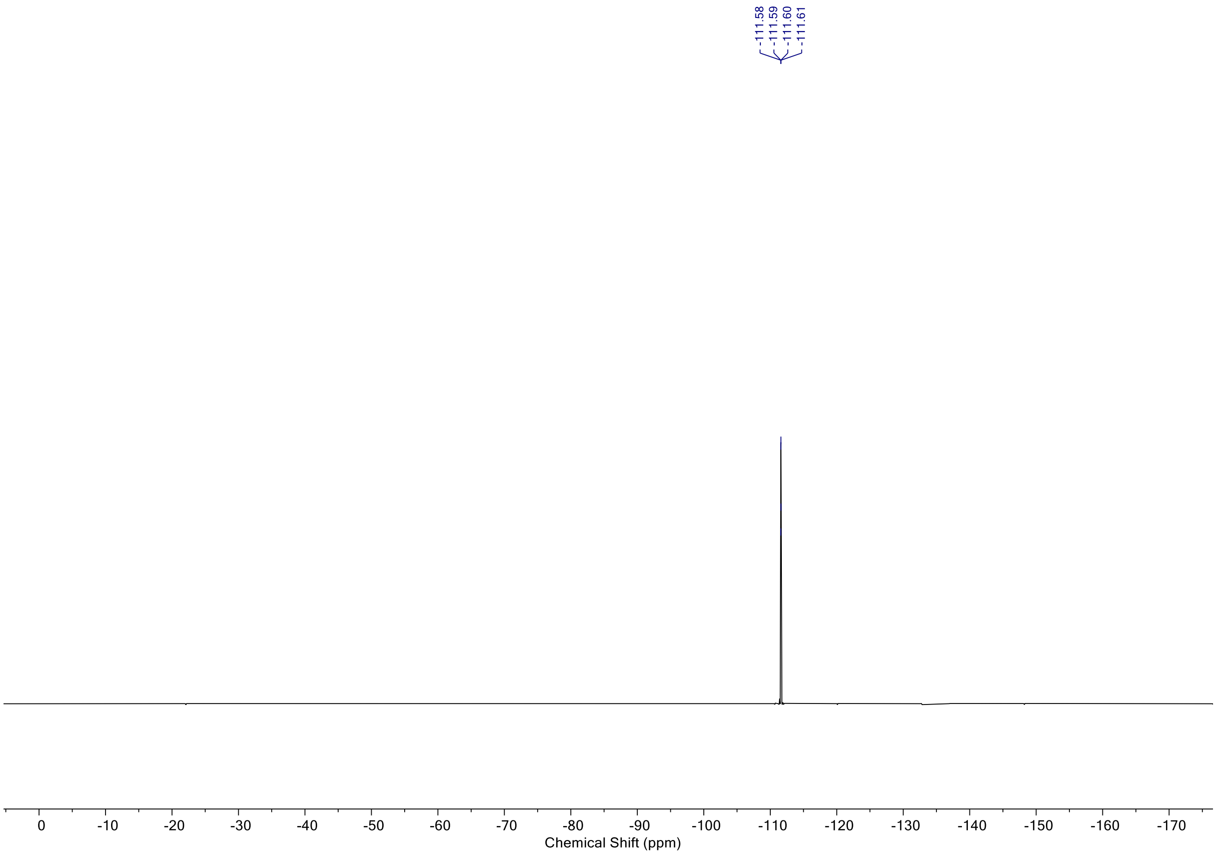

Figure S115: ^{19}F NMR spectrum of ***EE*-3** ($(CD_3)_2SO$).

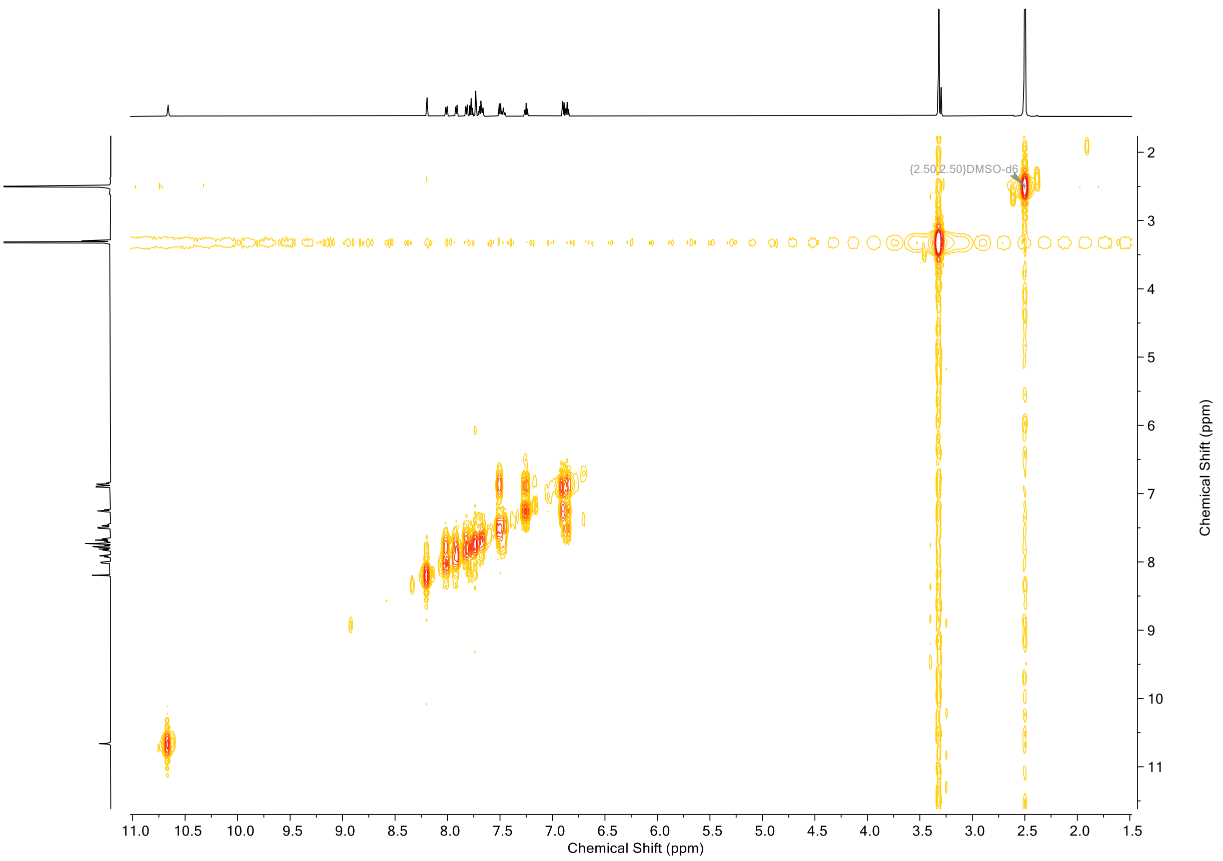

Figure S116: COSY spectrum of ***EE*-3** ($(CD_3)_2SO$).

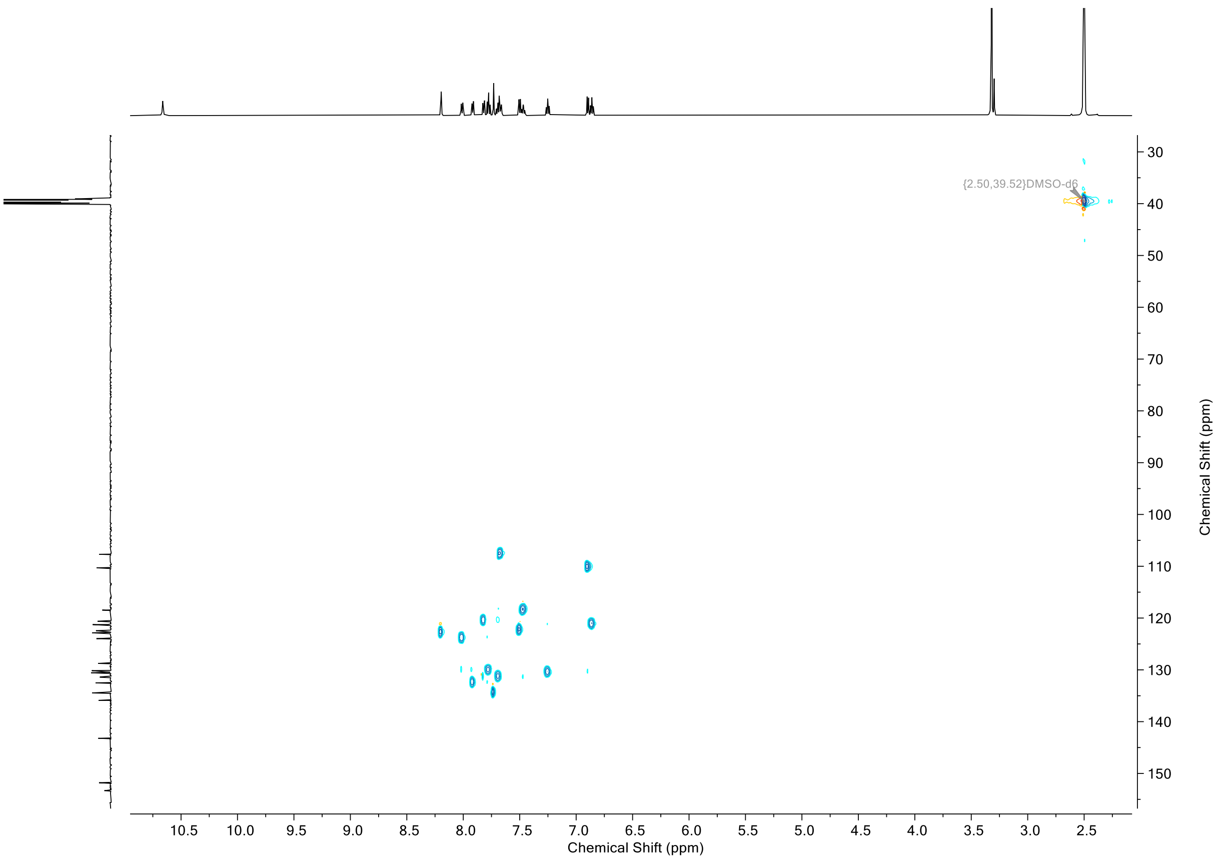

Figure S117: HSQC spectrum of ***EE*-3** ($(CD_3)_2SO$).

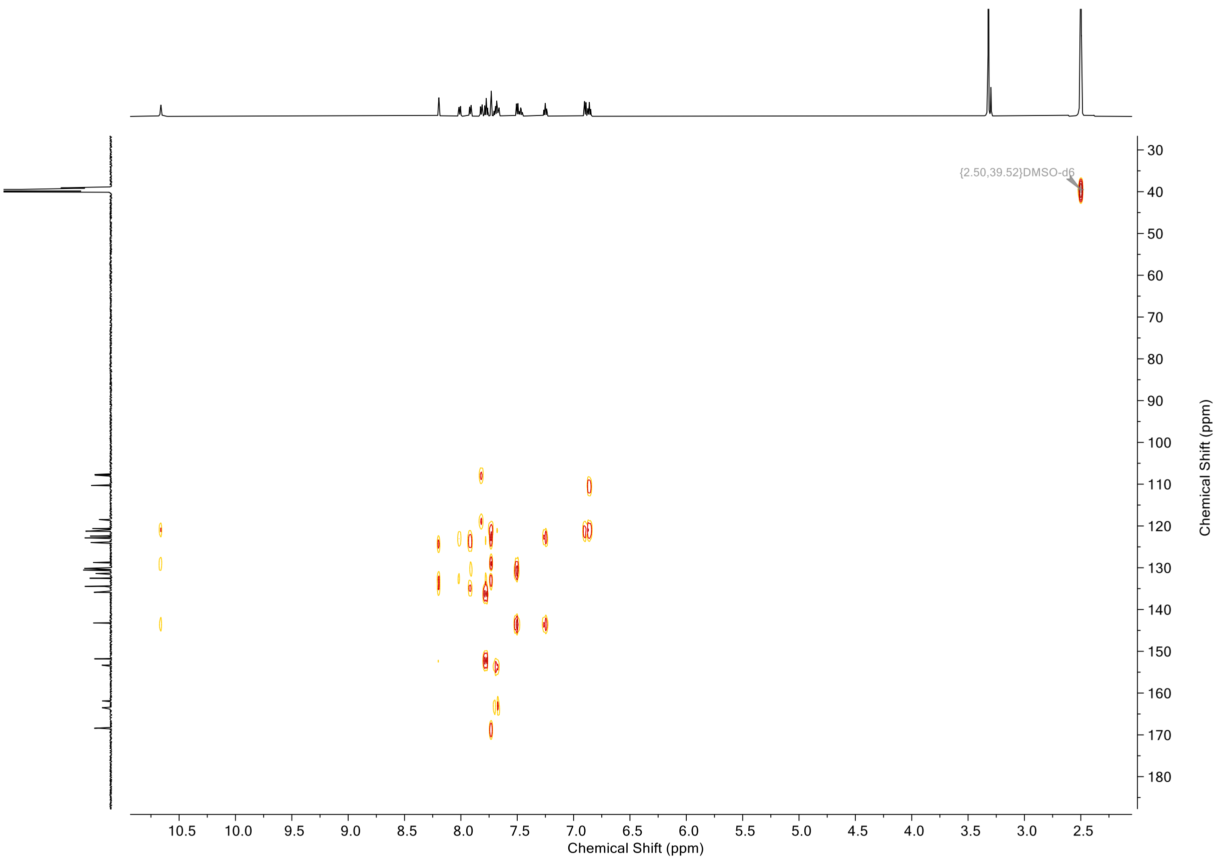

Figure S118: HMBC spectrum of ***EE*-3** ($(CD_3)_2SO$).

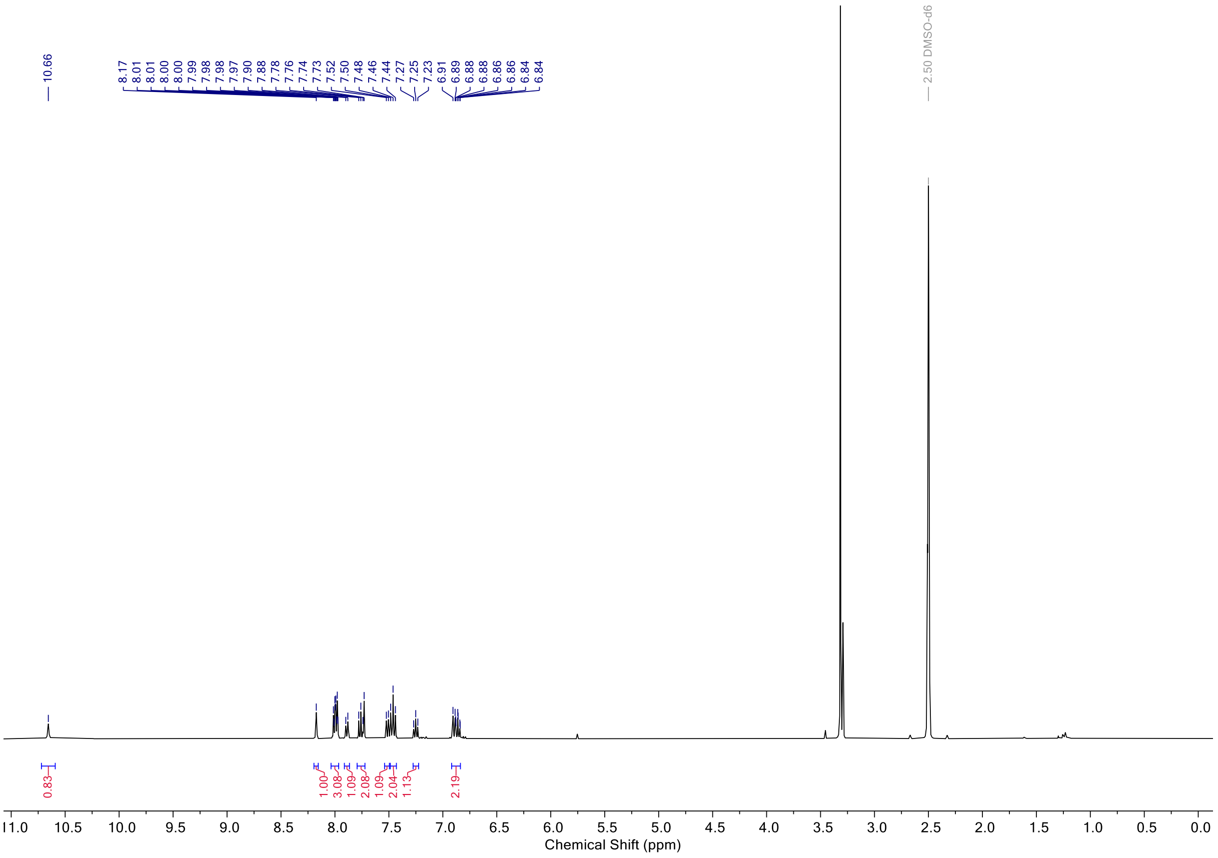

Figure S119: ^{1}H NMR spectrum of ***EE*-4** ($(CD_3)_2SO$).

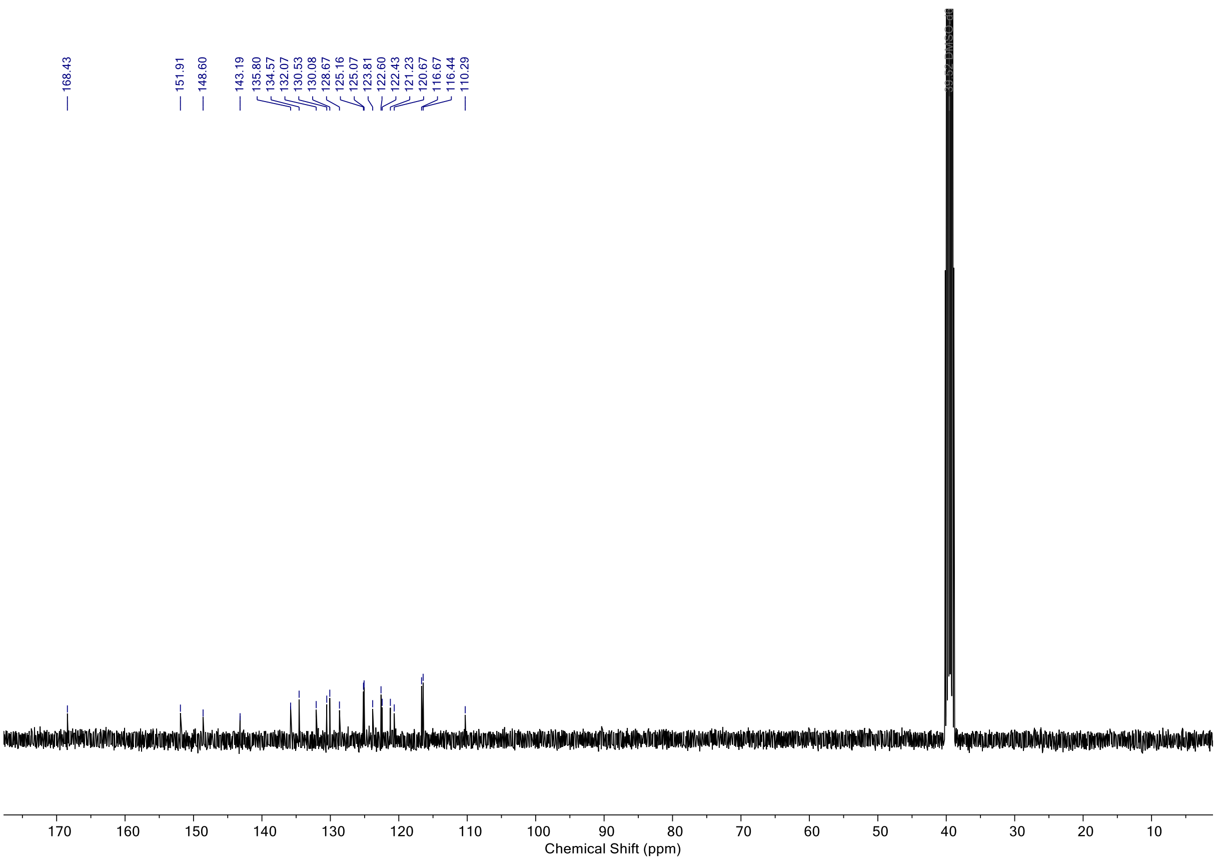

Figure S120: ^{13}C{^{1}H} NMR spectrum of ***EE*-4** ($(CD_3)_2SO$).

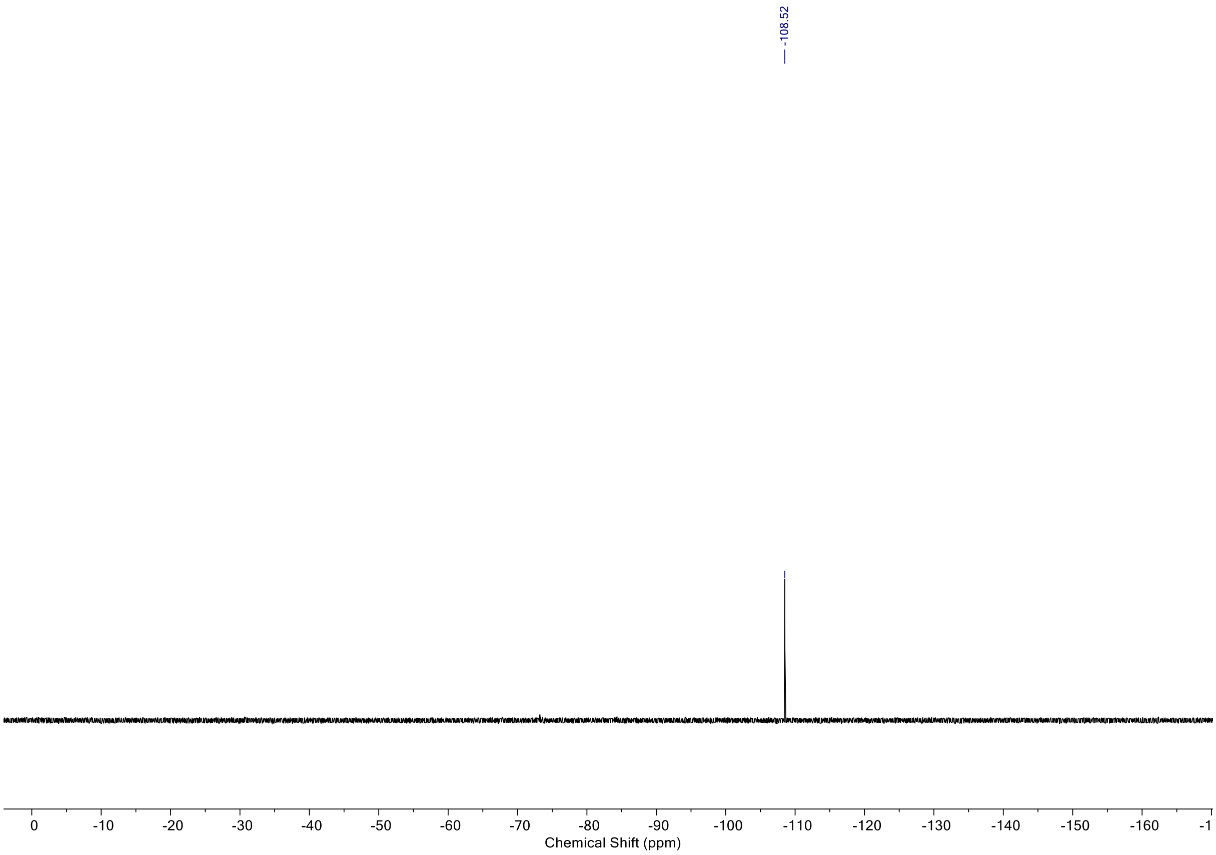

Figure S121: ^{19}F NMR spectrum of ***EE*-4** ($(CD_3)_2SO$).

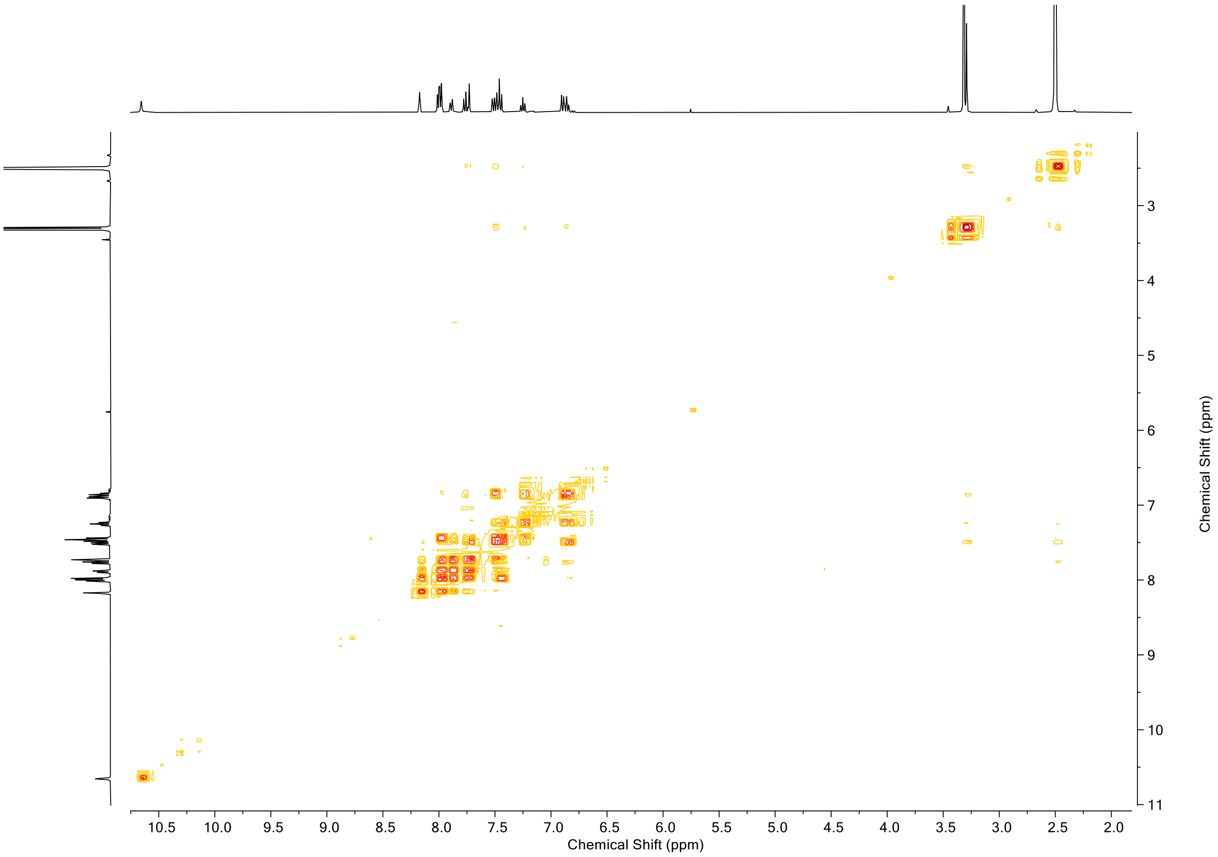

Figure S122: COSY spectrum of ***EE*-4** (($(CD_3)_2SO$).

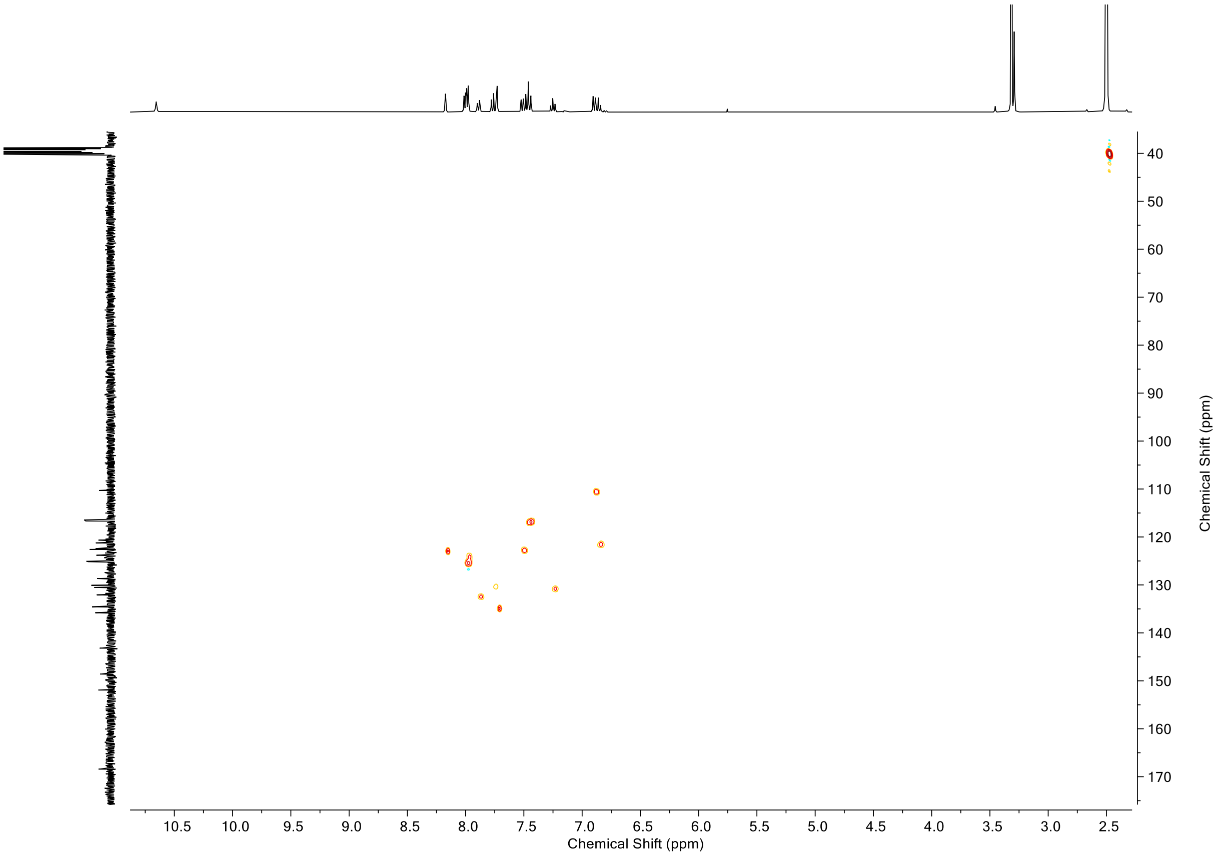

Figure S 123: HSQC spectrum of ***EE*-4** (($(CD_3)_2SO$).

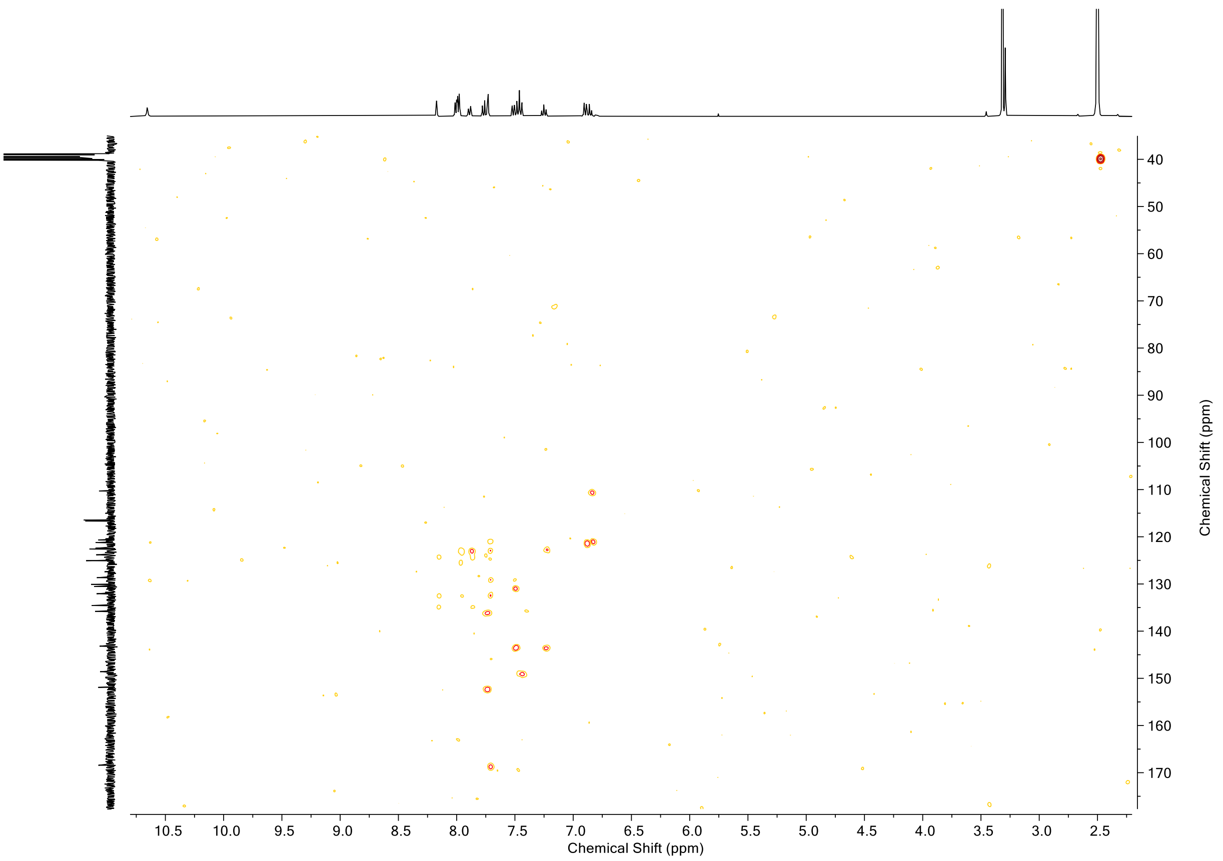

Figure S124: HMBC spectrum of ***EE*-4** ($(CD_3)_2SO$).

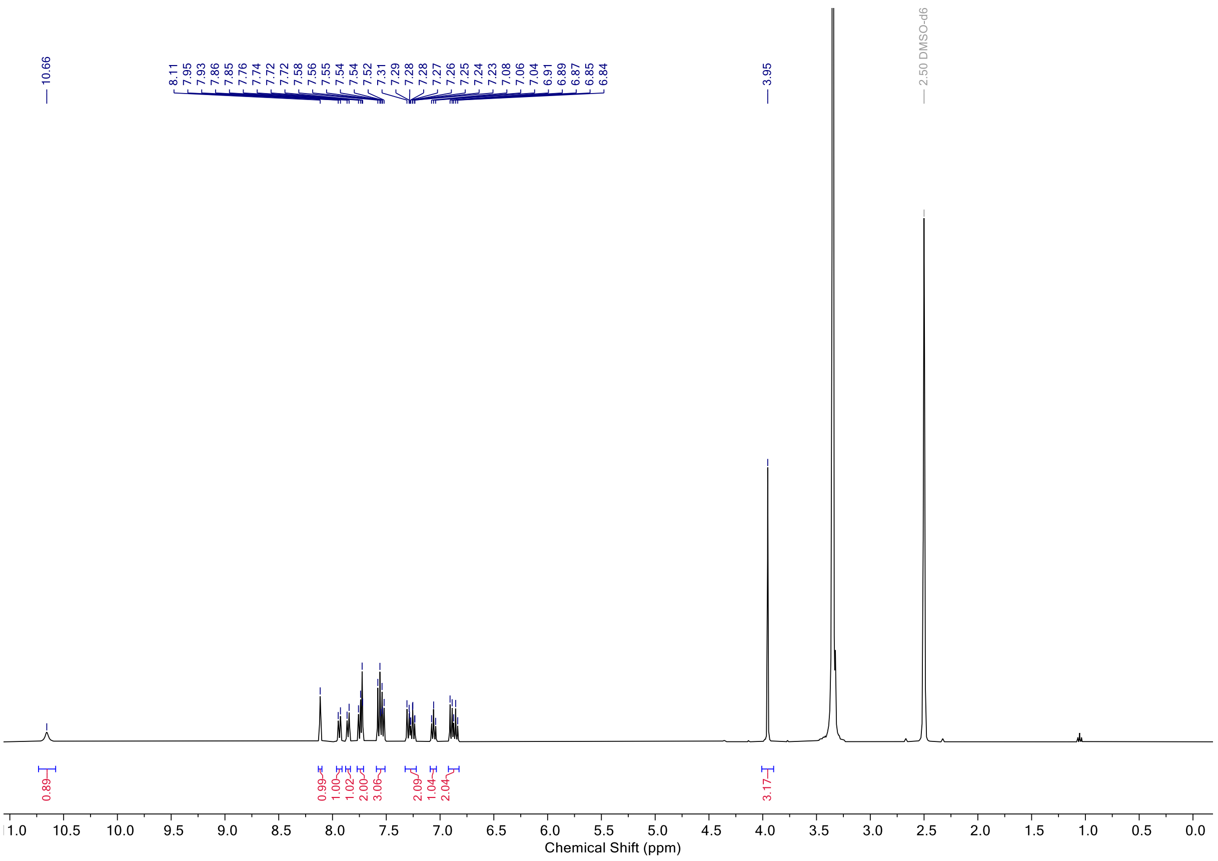

Figure S125: ^{1}H NMR spectrum of ***EE*-5** ($(CD_3)_2SO$).

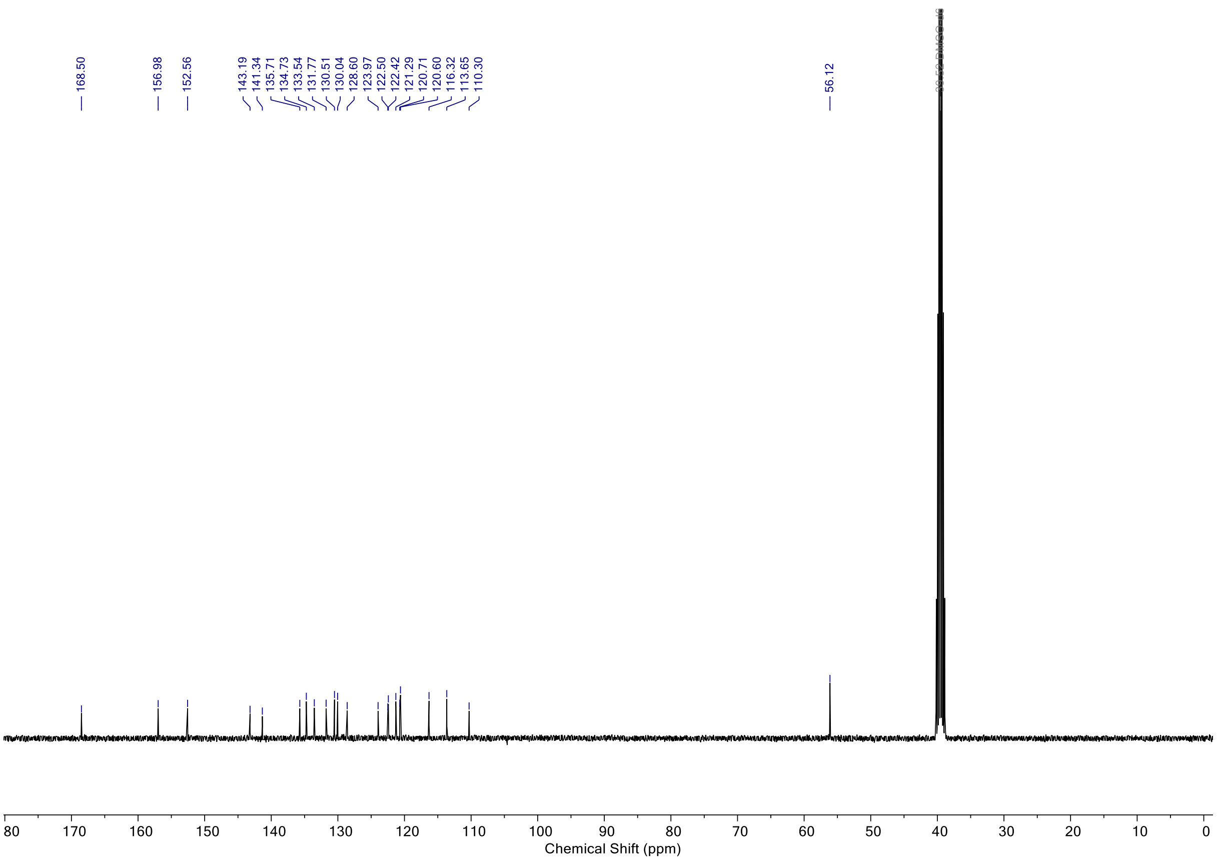

Figure S126: $^{13}C\{^{1}H\}$ NMR spectrum of ***EE*-5** ($(CD_3)_2SO$).

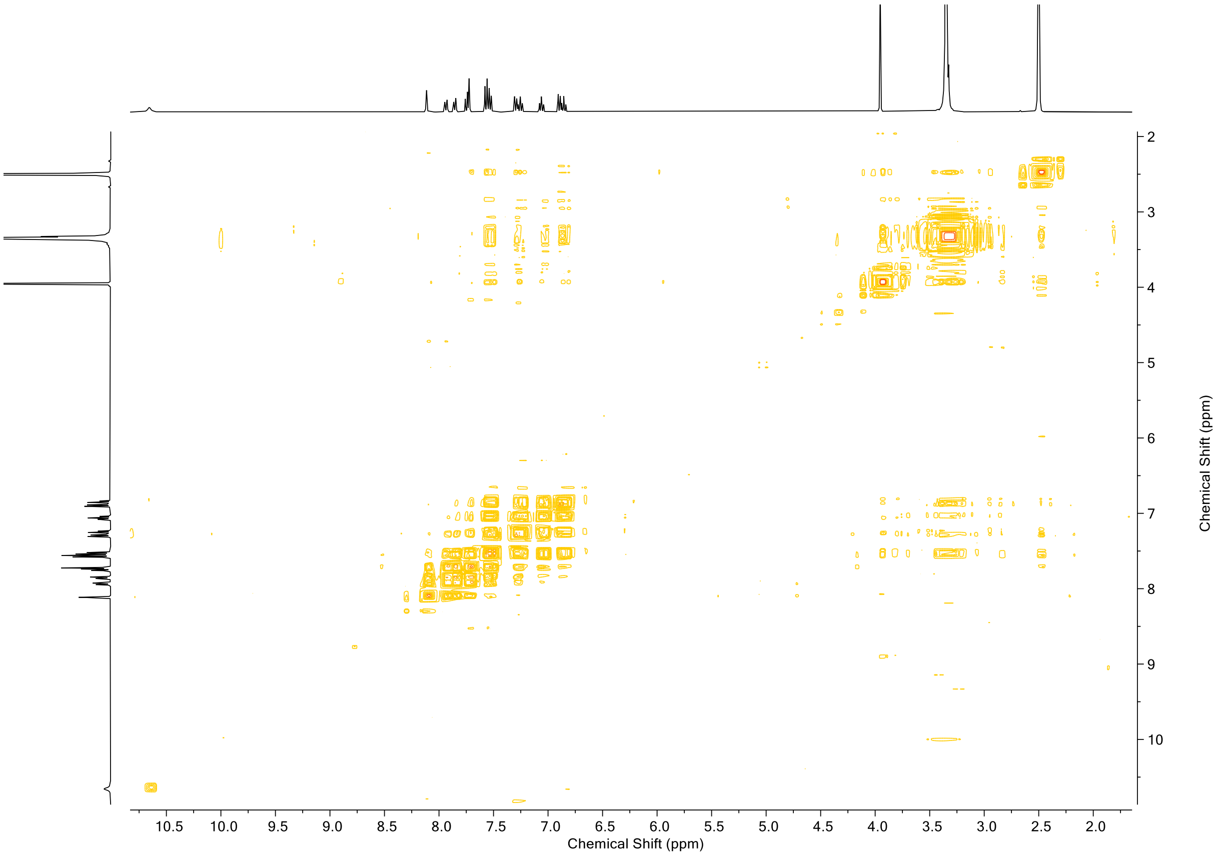

Figure S127: COSY spectrum of ***EE*-5** ($(CD_3)_2SO$).

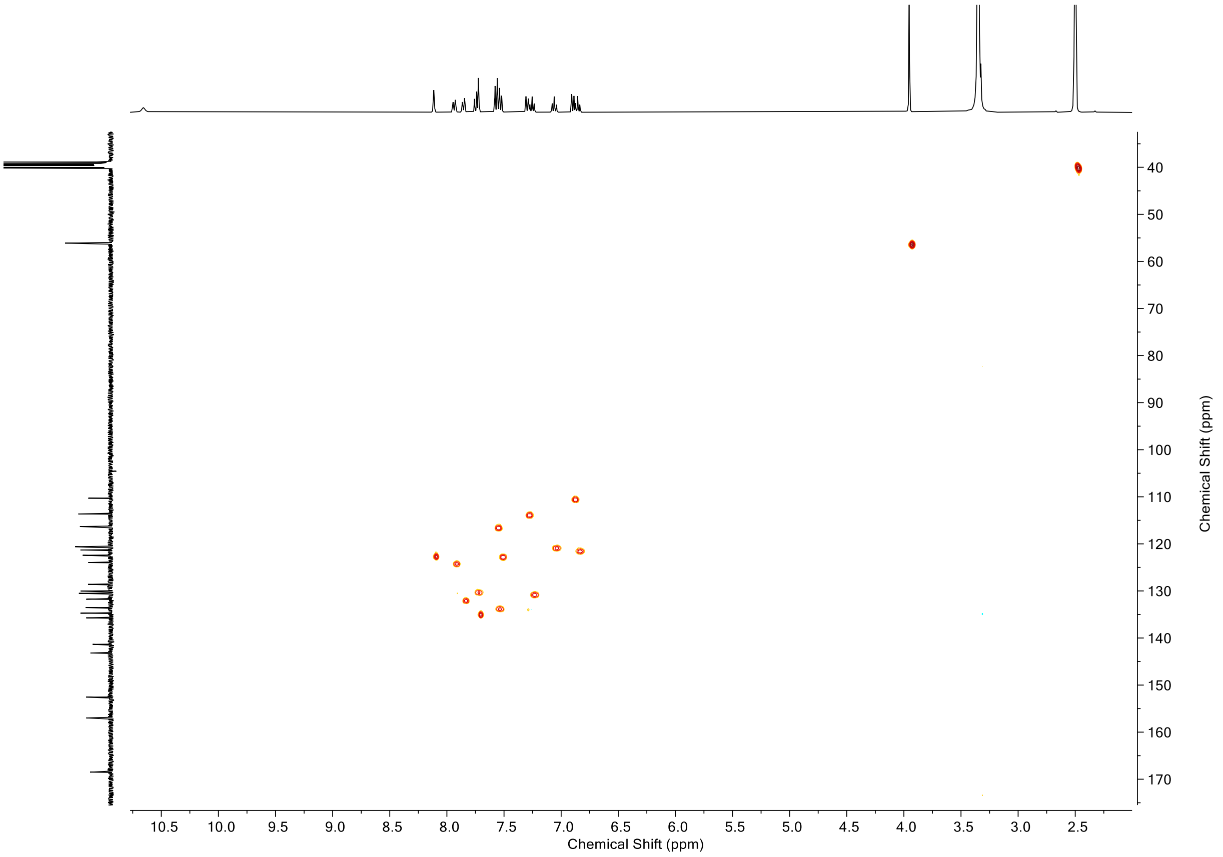

Figure S128: HSQC spectrum of ***EE*-5** ($(CD_3)_2SO$).

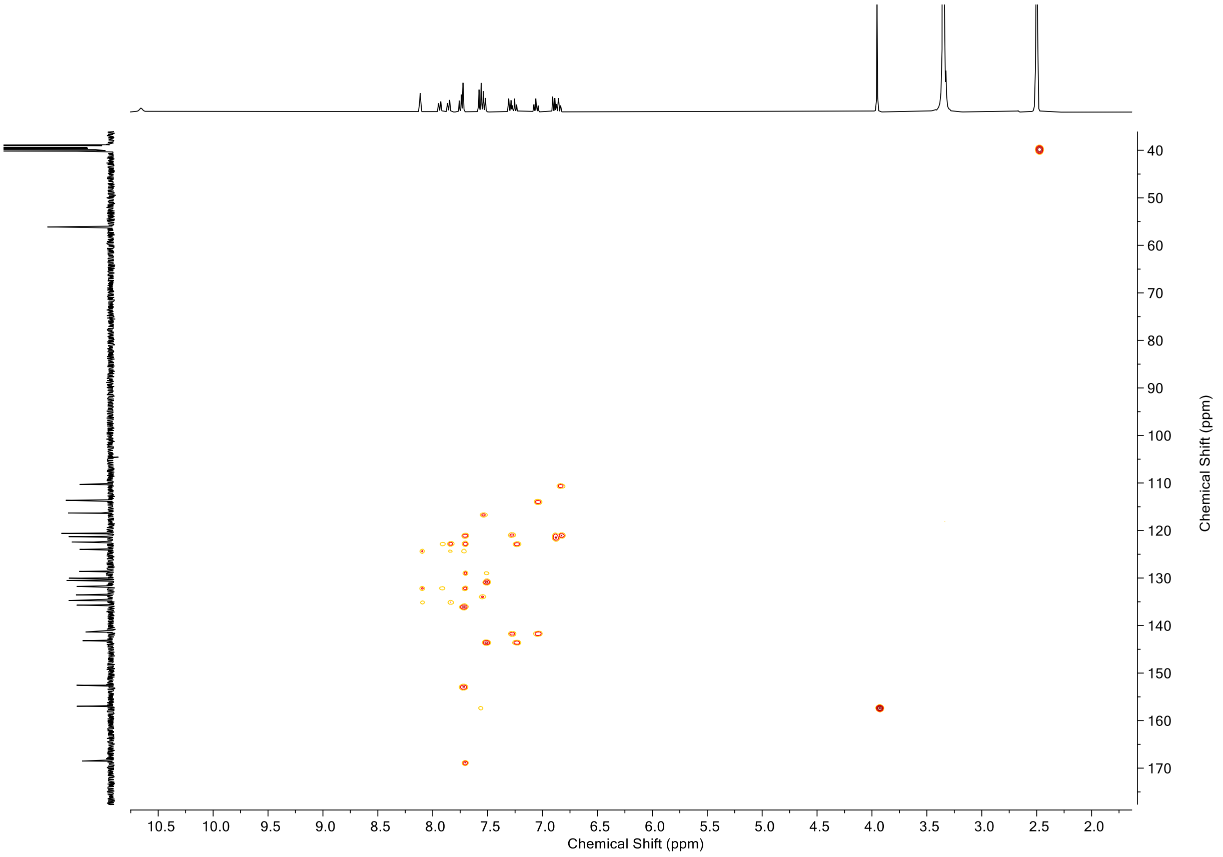

Figure S129: HMBC spectrum of ***EE*-5** ($(CD_3)_2SO$).

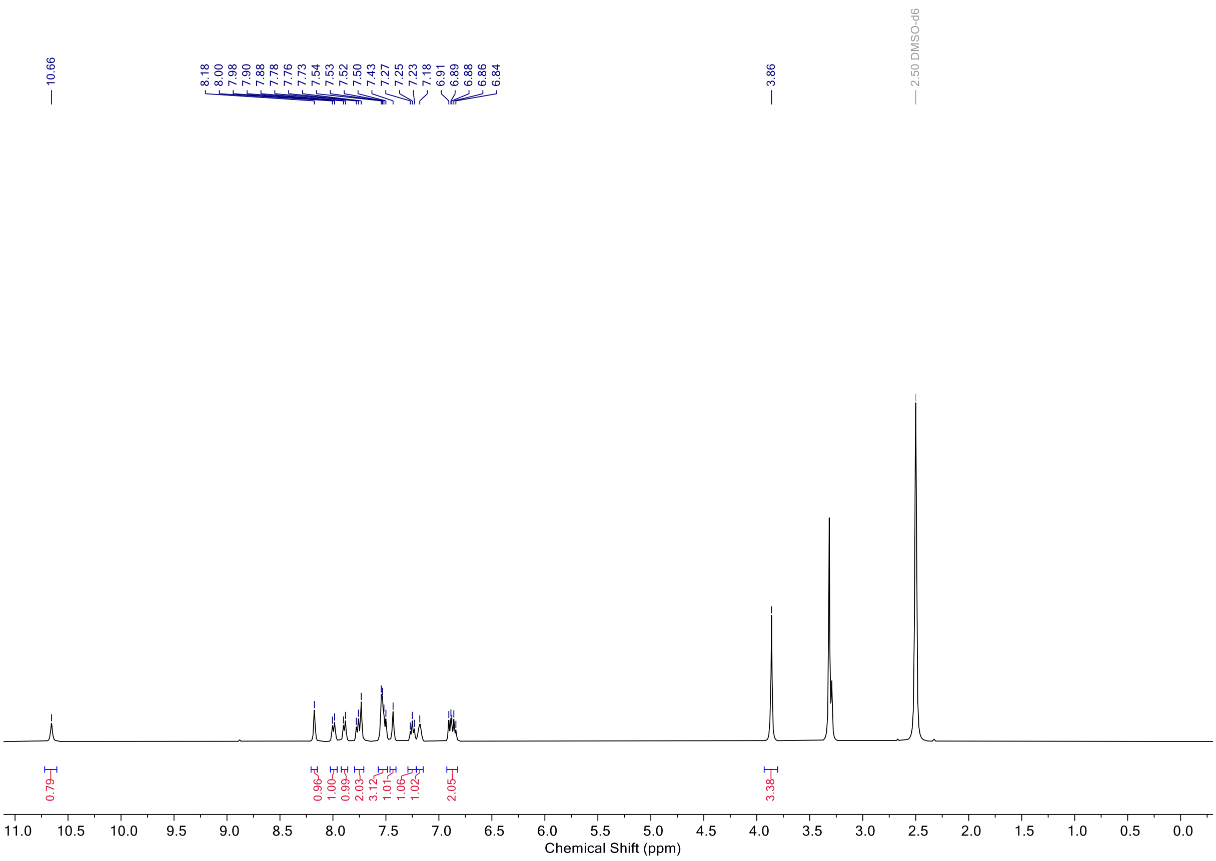

Figure S130: ^{1}H NMR spectrum of ***EE*-6** ($(CD_3)_2SO$).

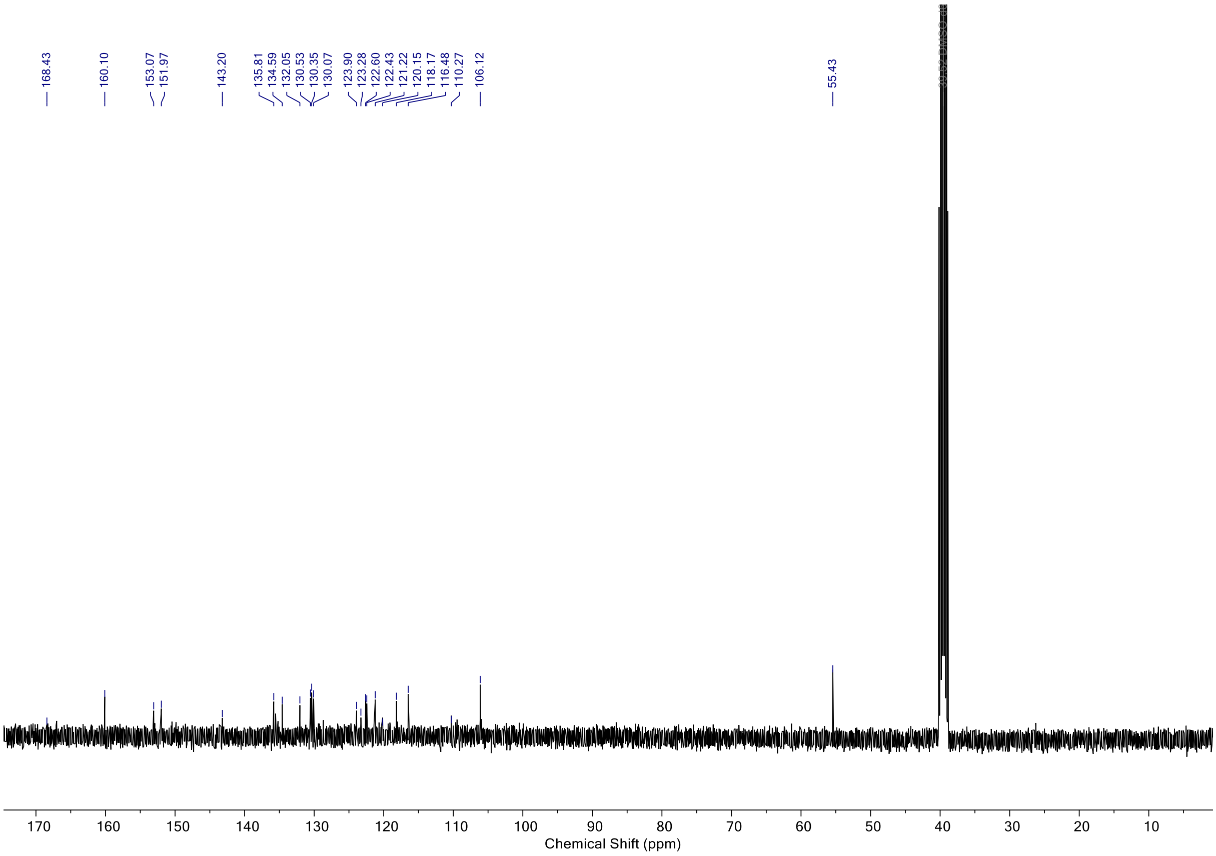

Figure S131: ^{13}C{^{1}H} NMR spectrum of ***EE*-6** ($(CD_3)_2SO$).

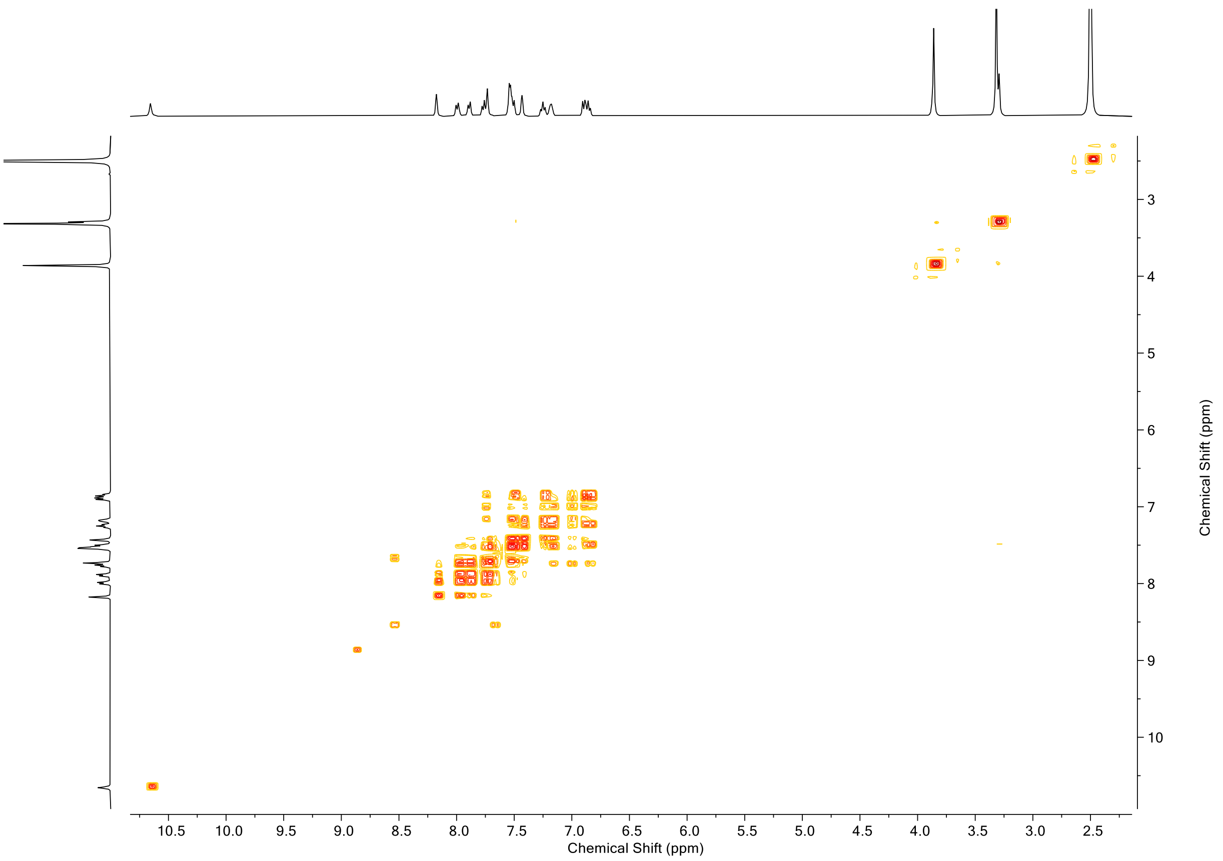

Figure S132: COSY spectrum of ***EE*-6** ($(CD_3)_2SO$).

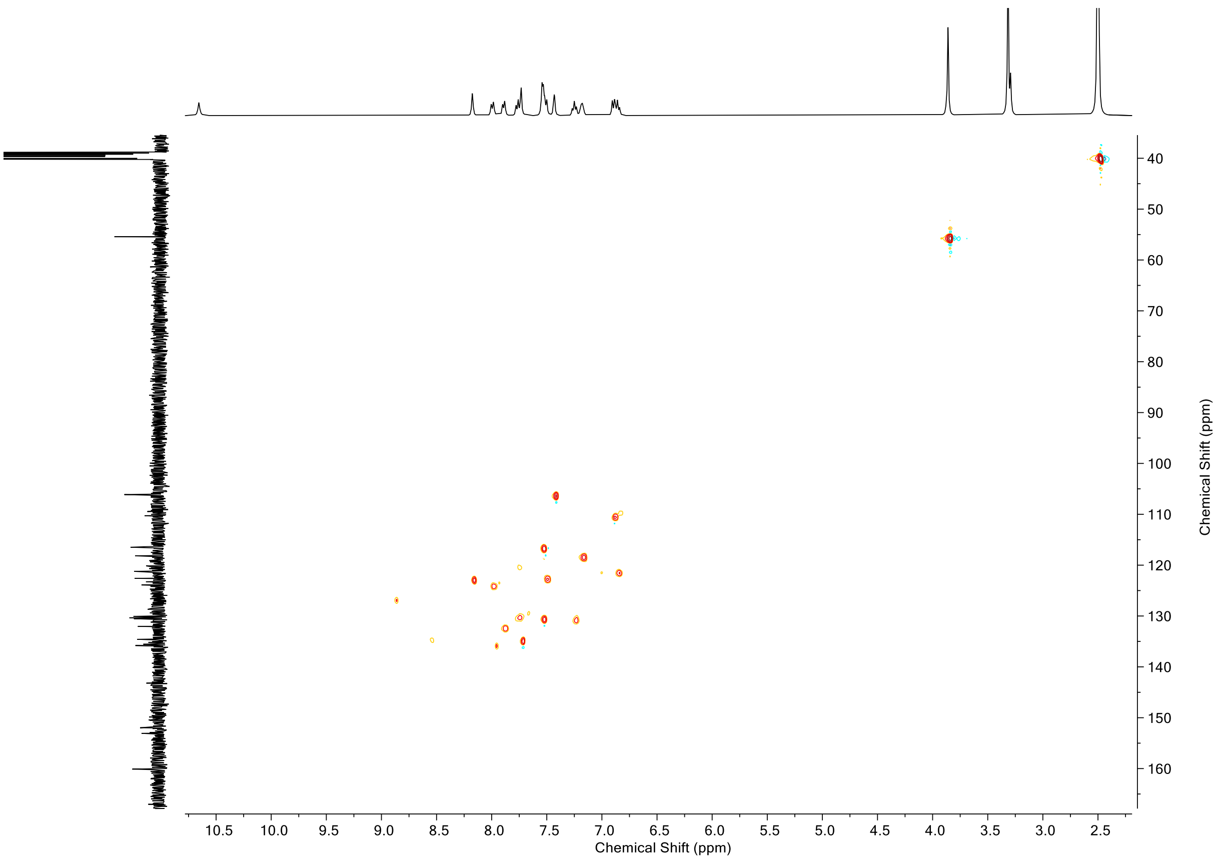

Figure S133: HSQC spectrum of ***EE*-6** ($(CD_3)_2SO$).

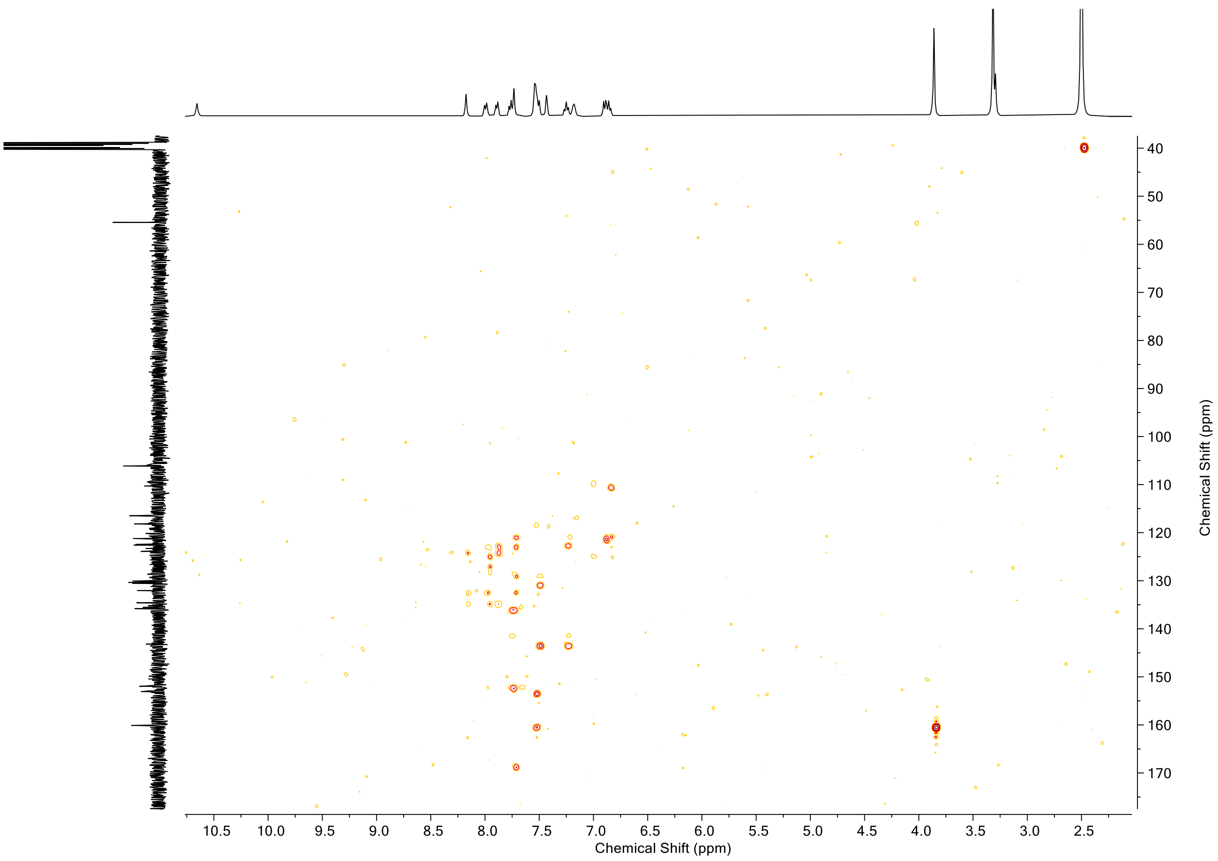

Figure S134: HMBC spectrum of ***EE*-6** (($(CD_3)_2SO$).

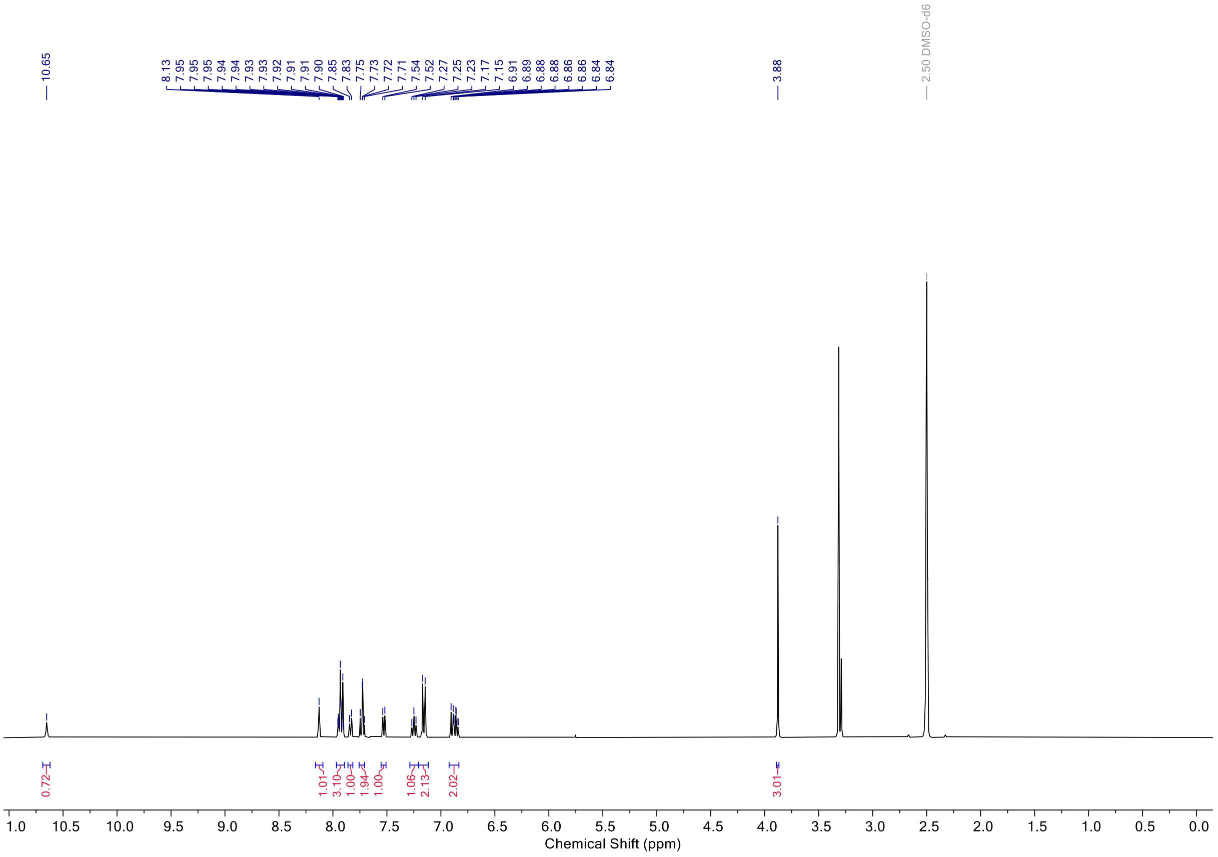

Figure S135: ^{1}H NMR spectrum of ***EE*-7** (($(CD_3)_2SO$).

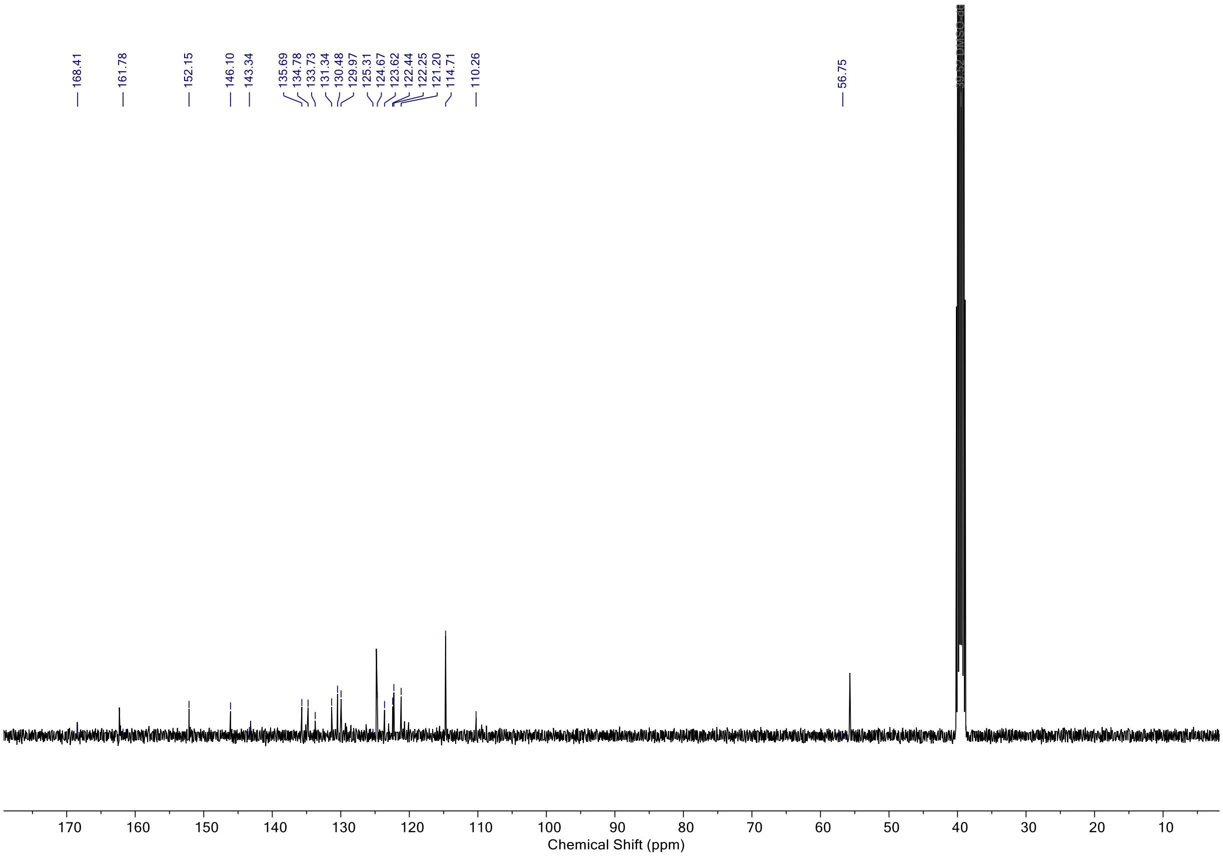

Figure S136: $^{13}C\{^{1}H\}$ NMR spectrum of ***EE*-7** ($(CD_3)_2SO$).

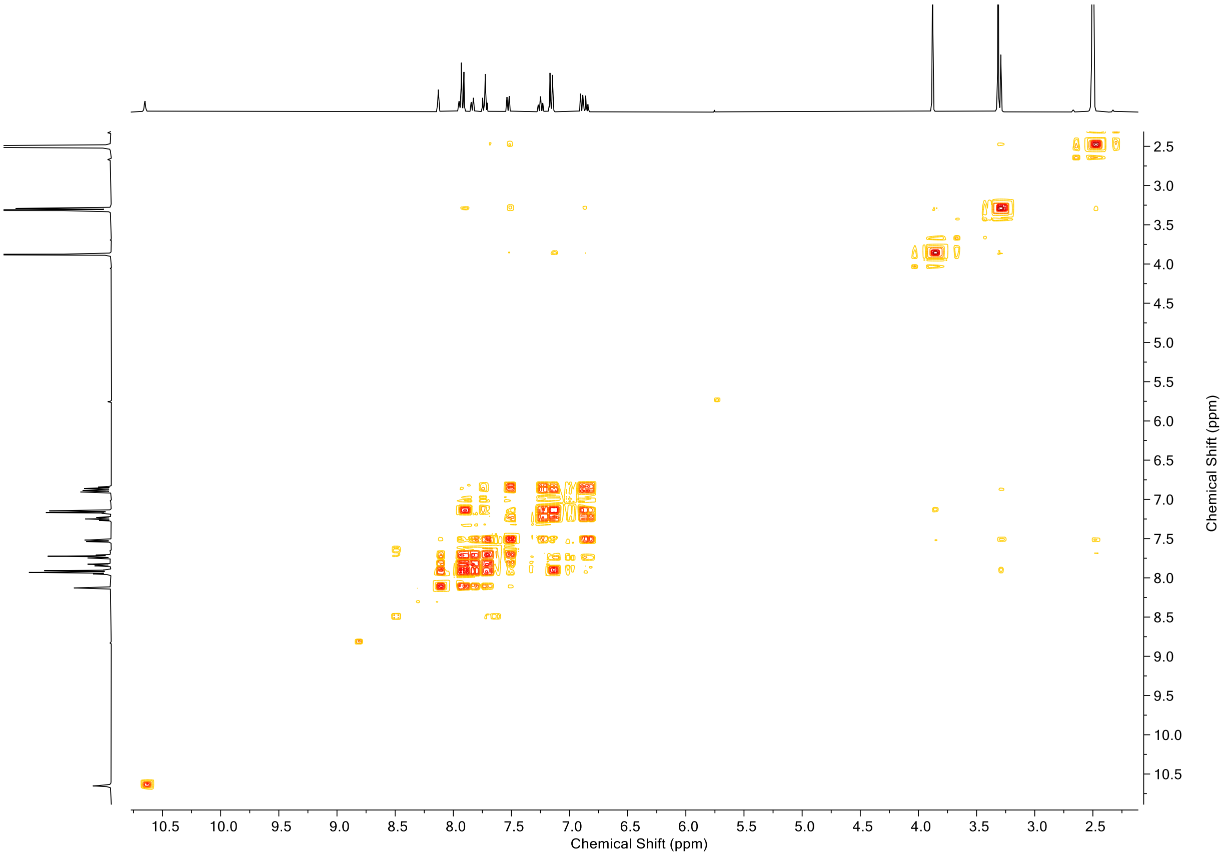

Figure S137: COSY spectrum of ***EE*-7** ($(CD_3)_2SO$).

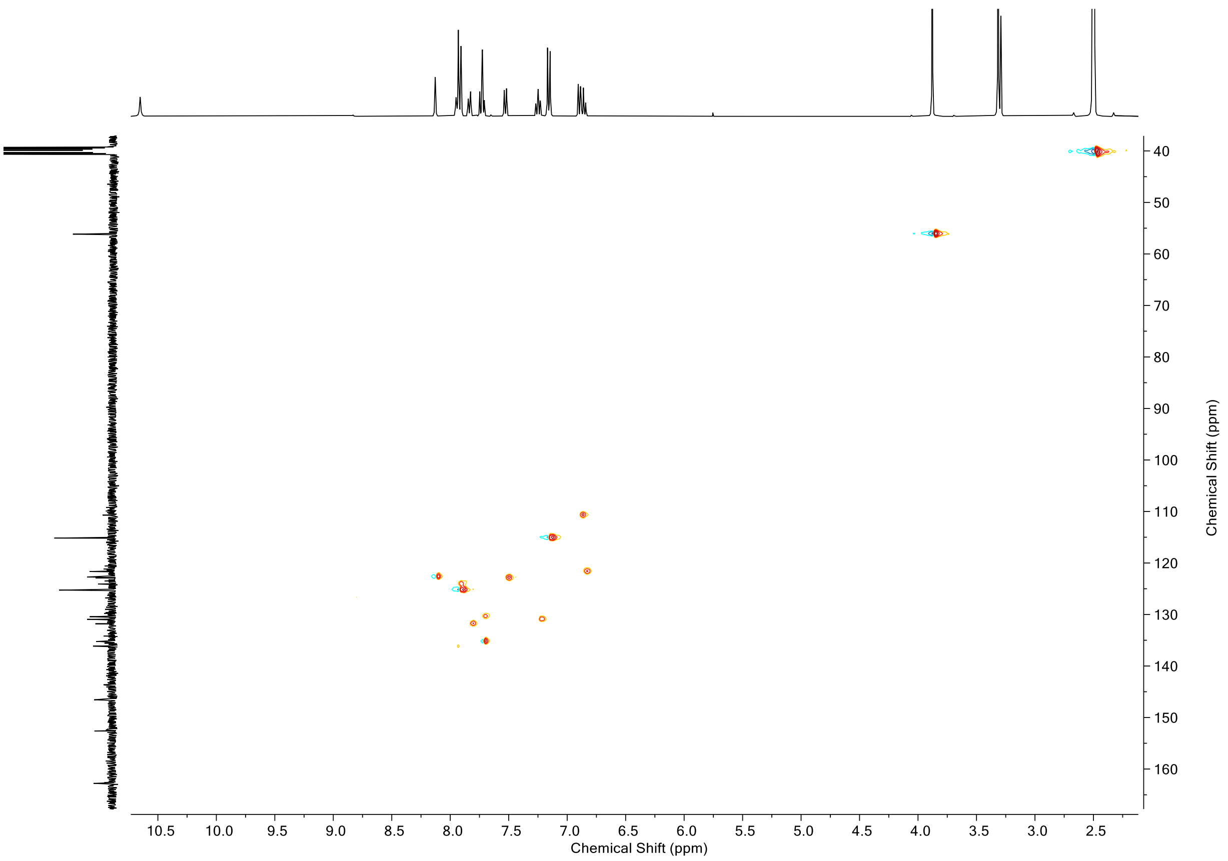

Figure S138: HSQC spectrum of ***EE*-7** ($(CD_3)_2SO$).

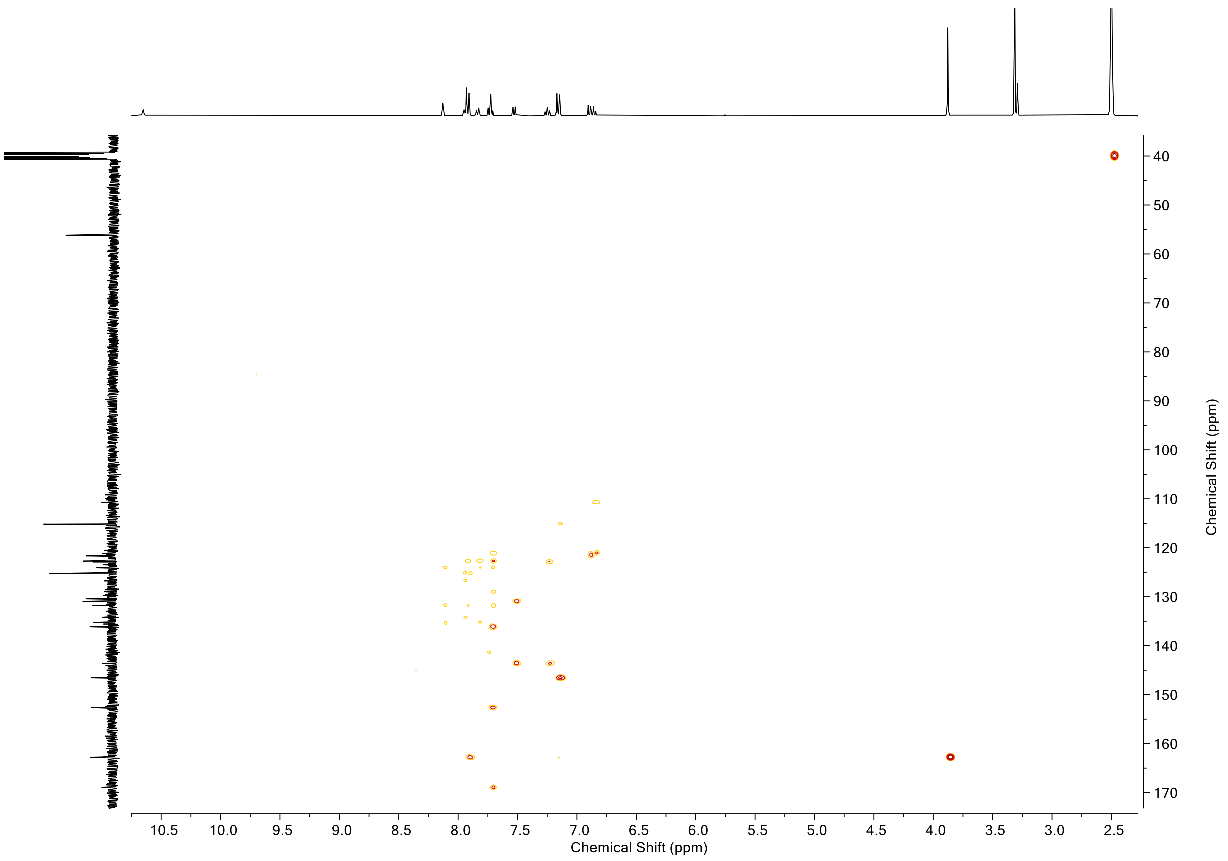

Figure S139: HMBC spectrum of ***EE*-7** ($(CD_3)_2SO$).

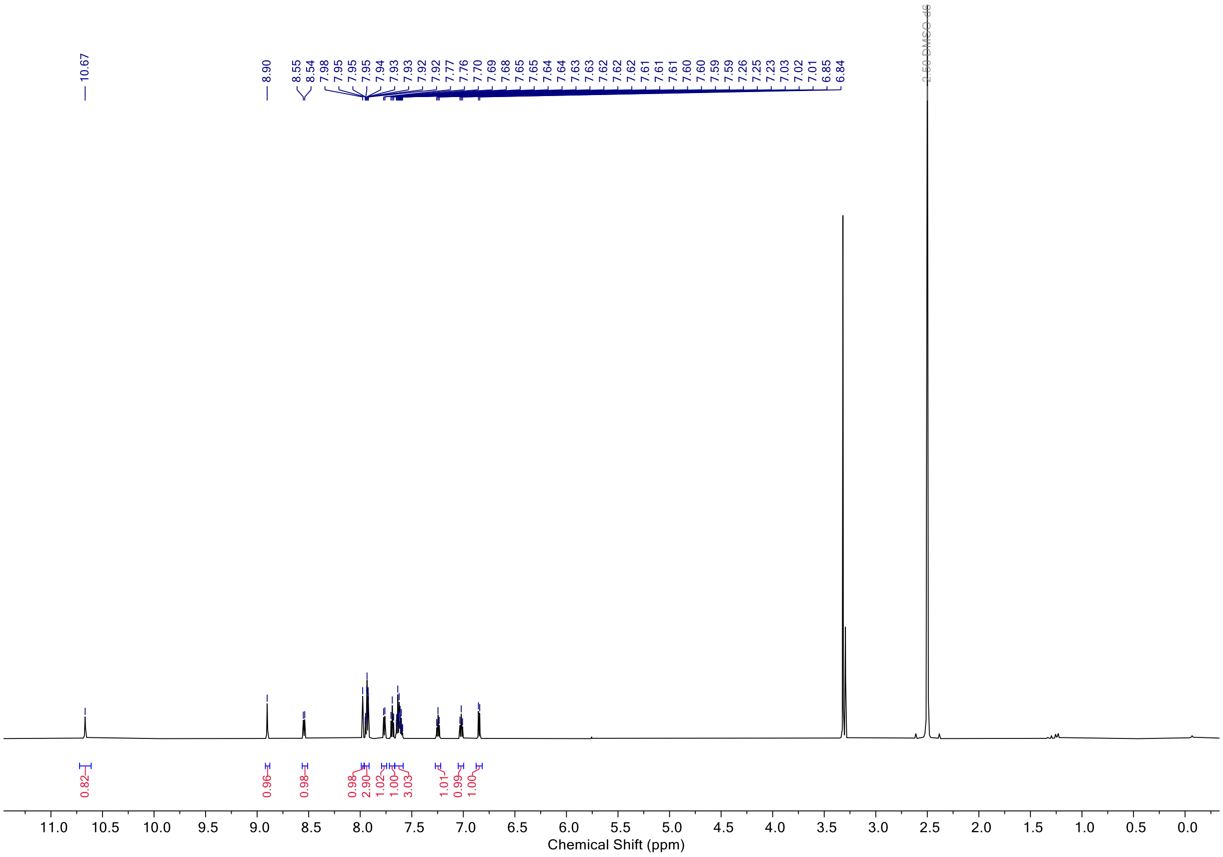

Figure S140: ^{1}H NMR spectrum of ***EZ*-1** ($(CD_3)_2SO$).

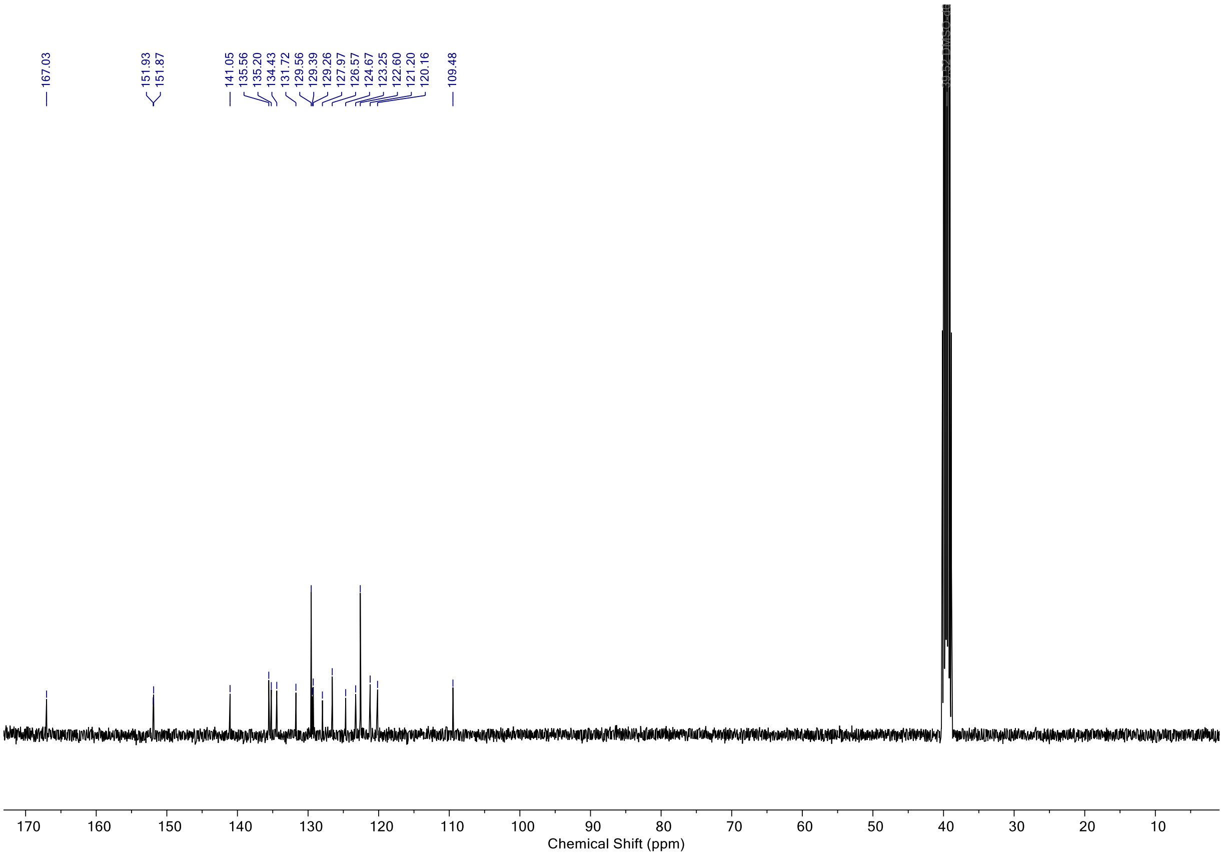

Figure S141: ^{13}C{^{1}H} NMR spectrum of ***EZ*-1** ($(CD_3)_2SO$).

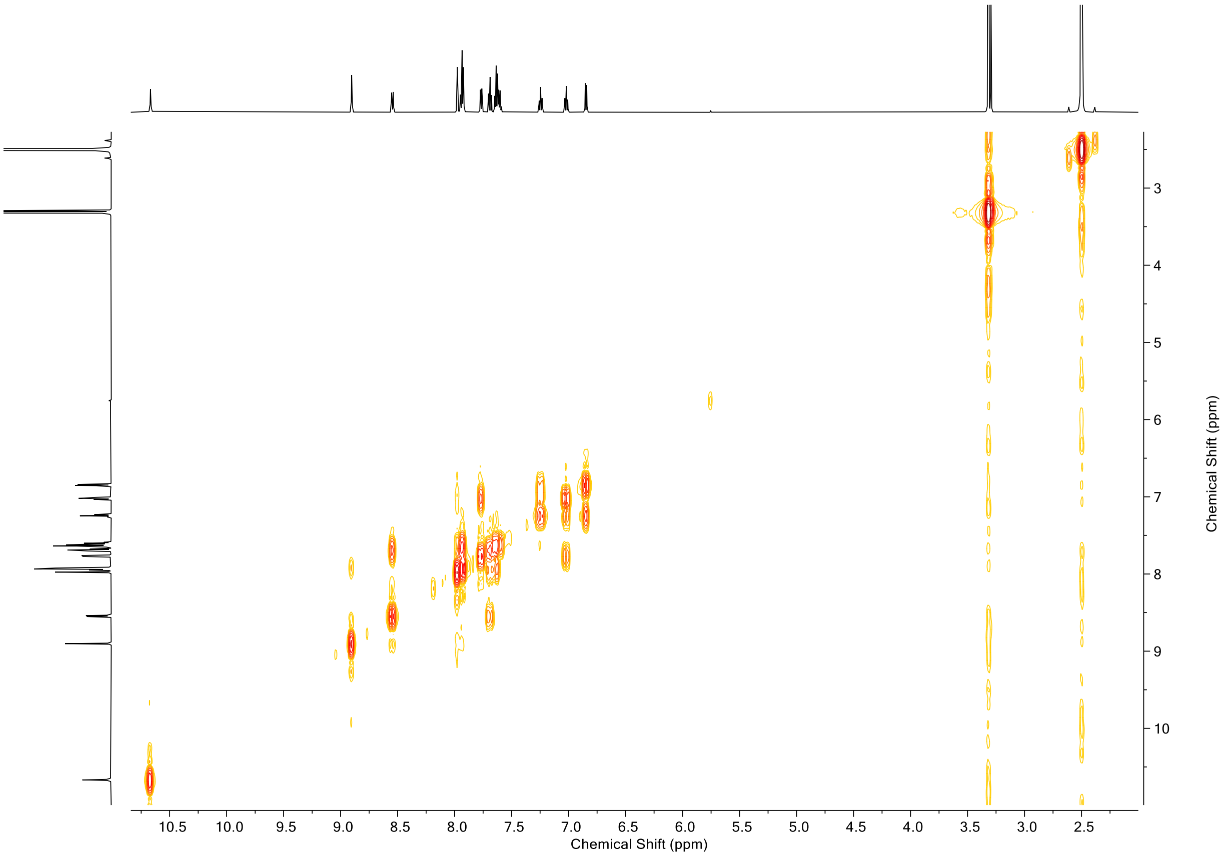

Figure S142: COSY spectrum of ***EZ*-1** (($(CD_3)_2SO$).

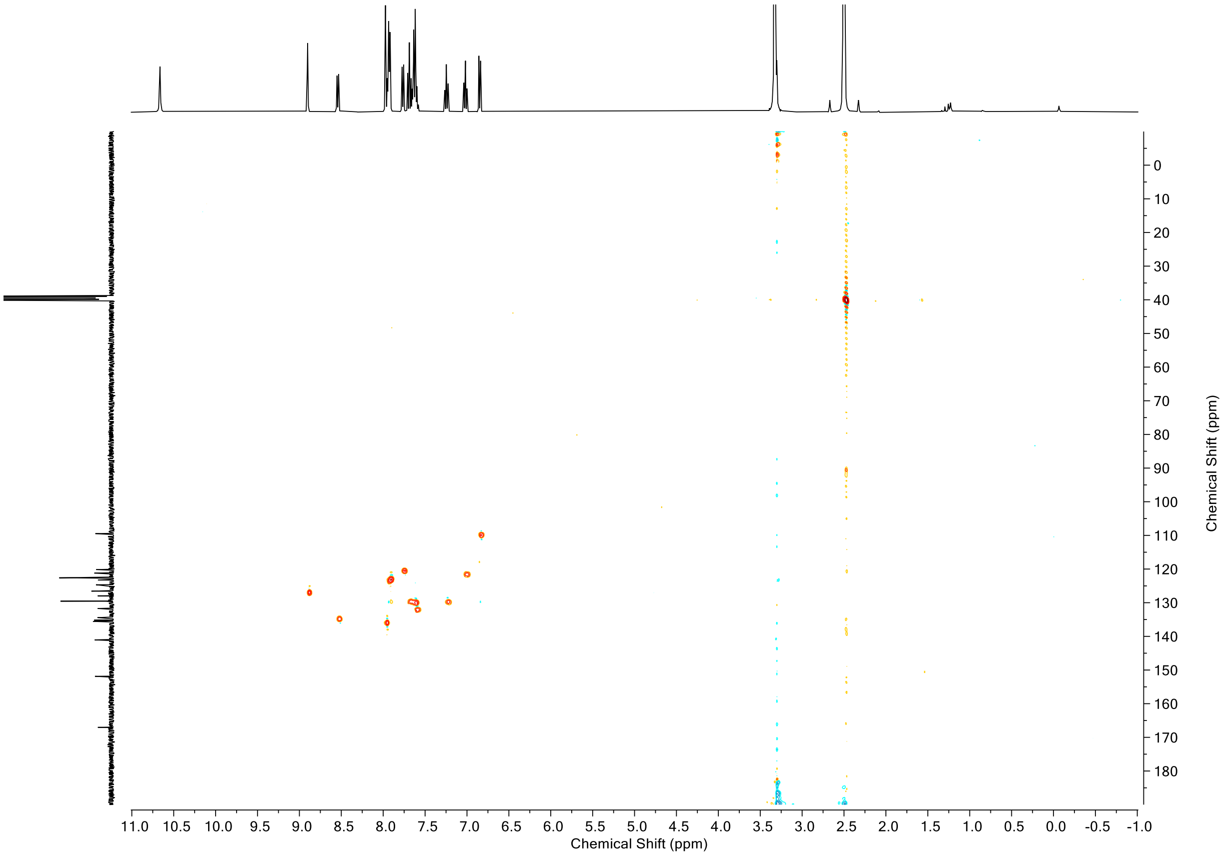

Figure S143: HSQC spectrum of ***EZ*-1** ($(CD_3)_2SO$).

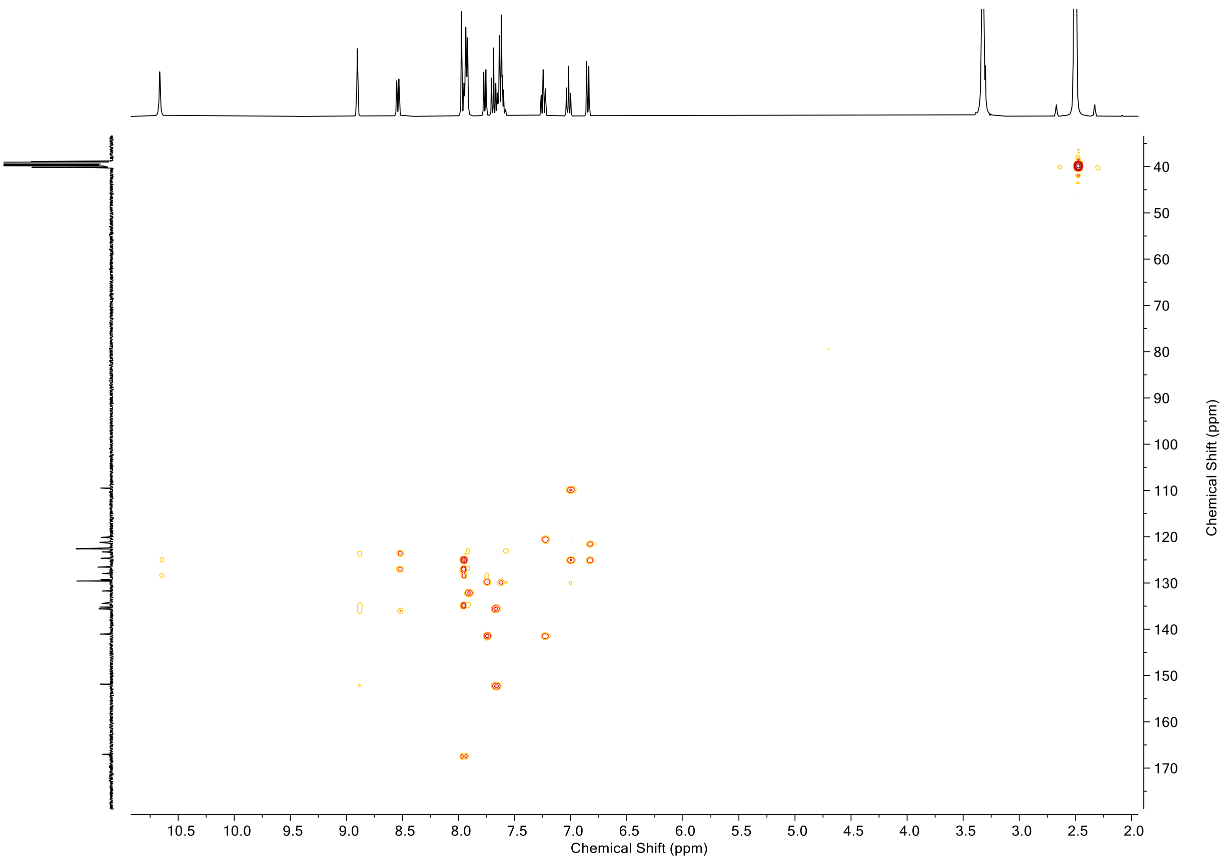

Figure S144: HMBC spectrum of ***EZ*-1** (($(CD_3)_2SO$).

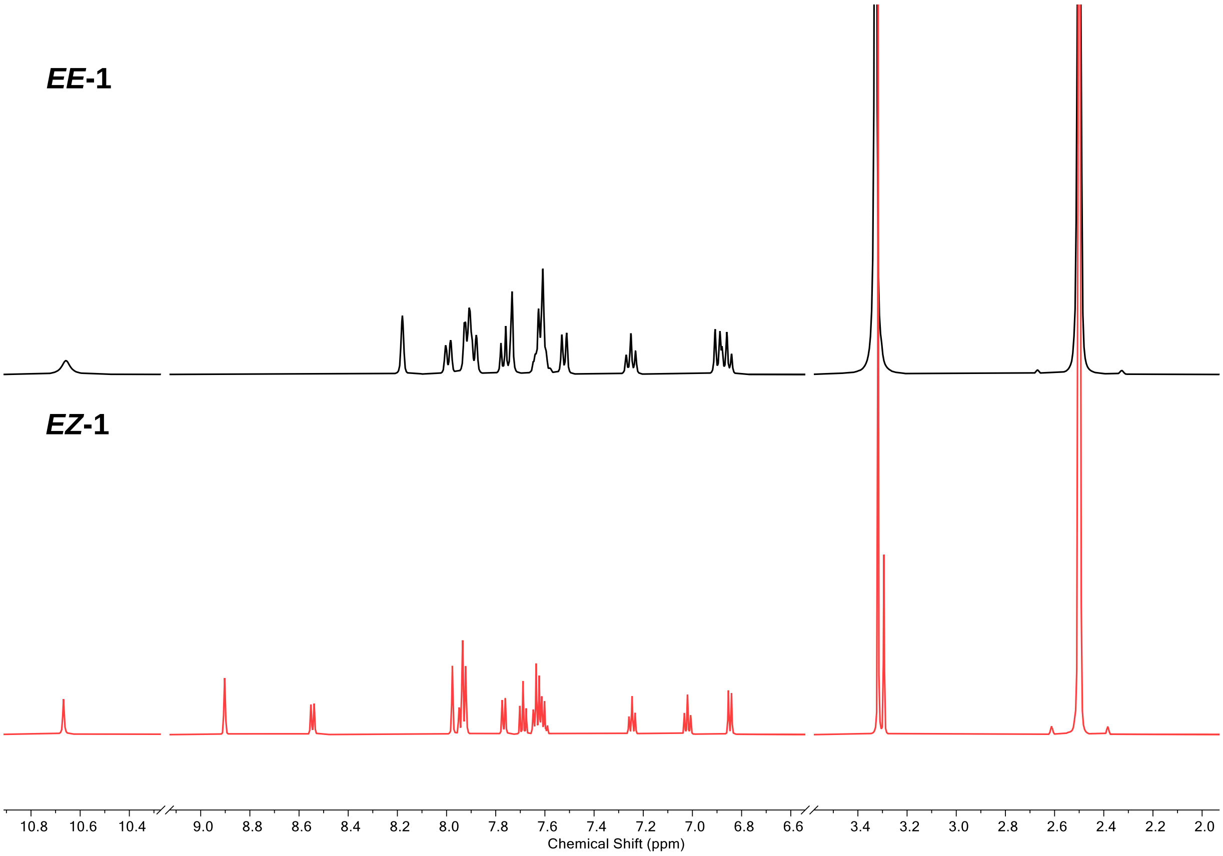

Figure S145: Comparison of **^{1}H NMR** spectra of ***EE*-1** (black) and ***EZ*-1** (red) (($(CD_3)_2SO$).

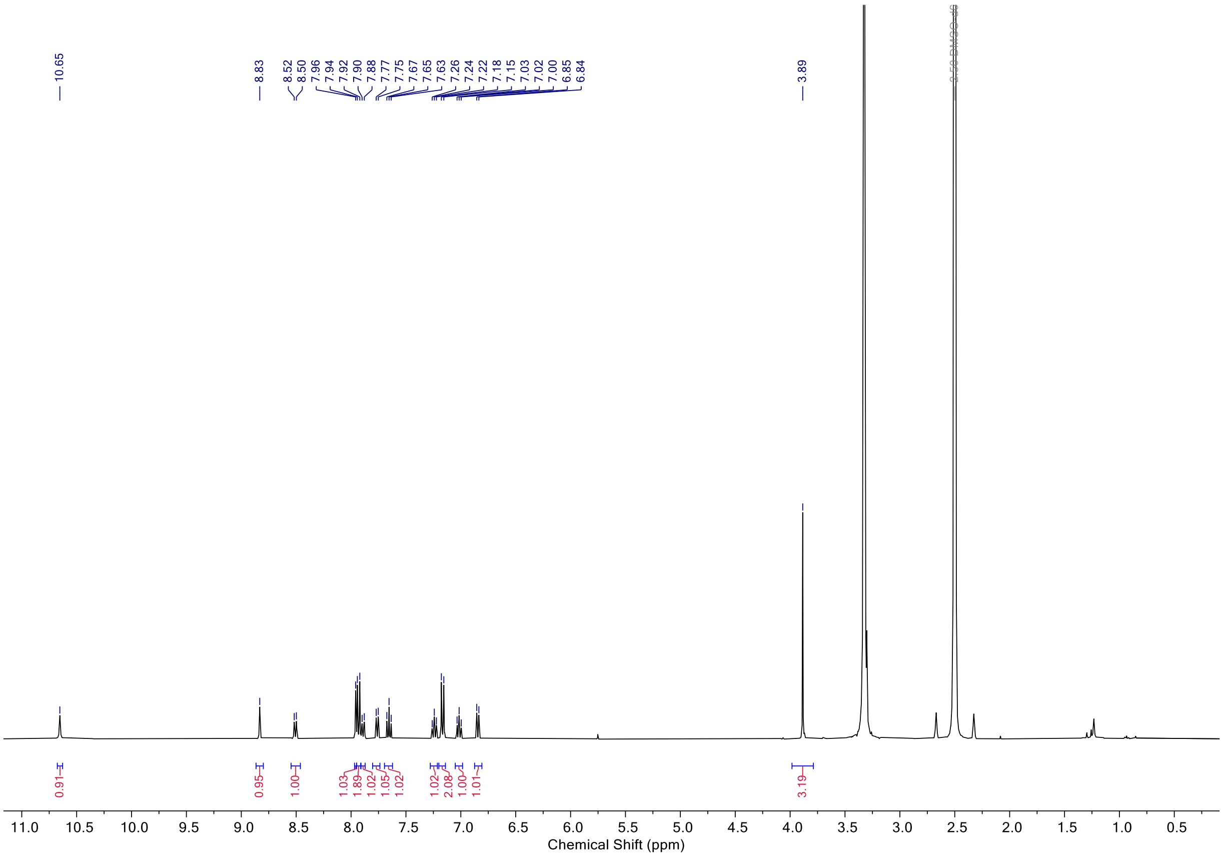

Figure S146: ^{1}H NMR spectrum of ***EZ*-7** ($(CD_3)_2SO$).

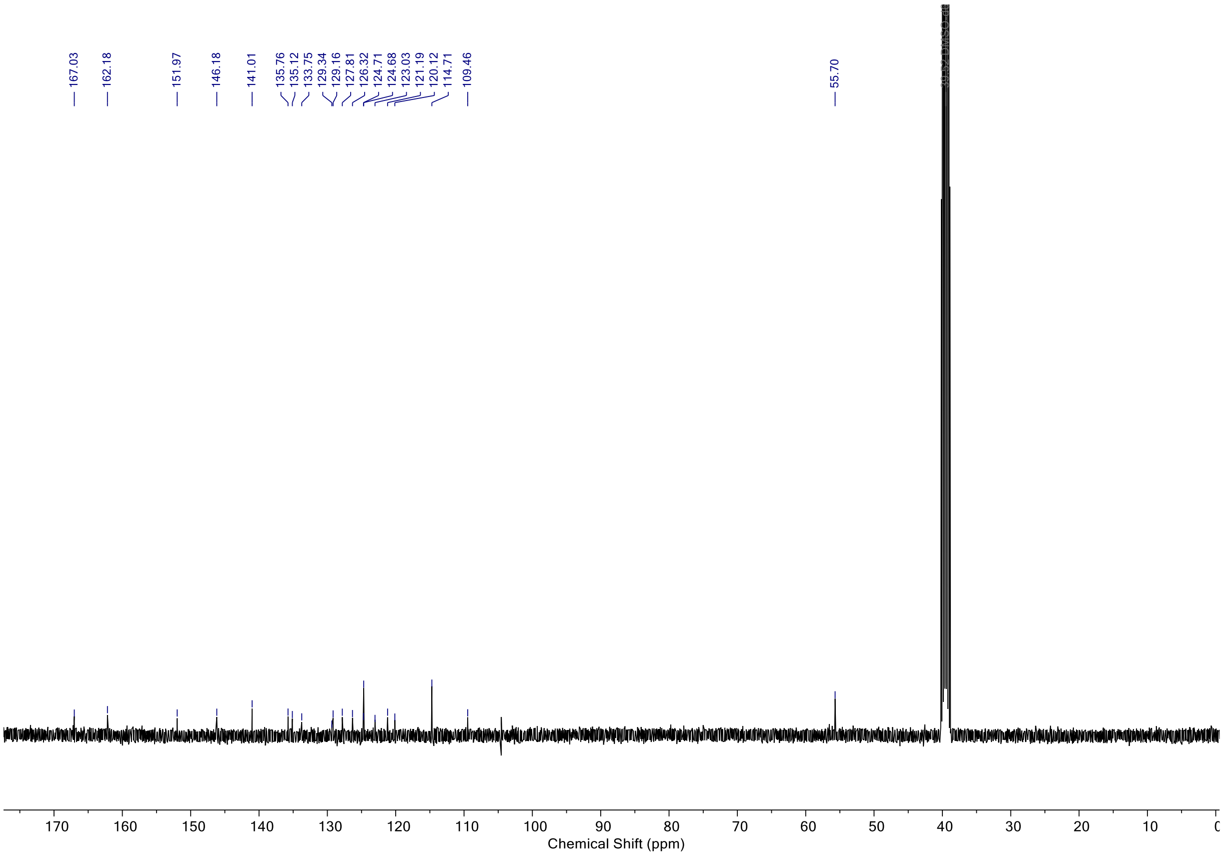

Figure S147: $^{13}C\{^1H\}$ NMR spectrum of ***EZ*-7** ($(CD_3)_2SO$).

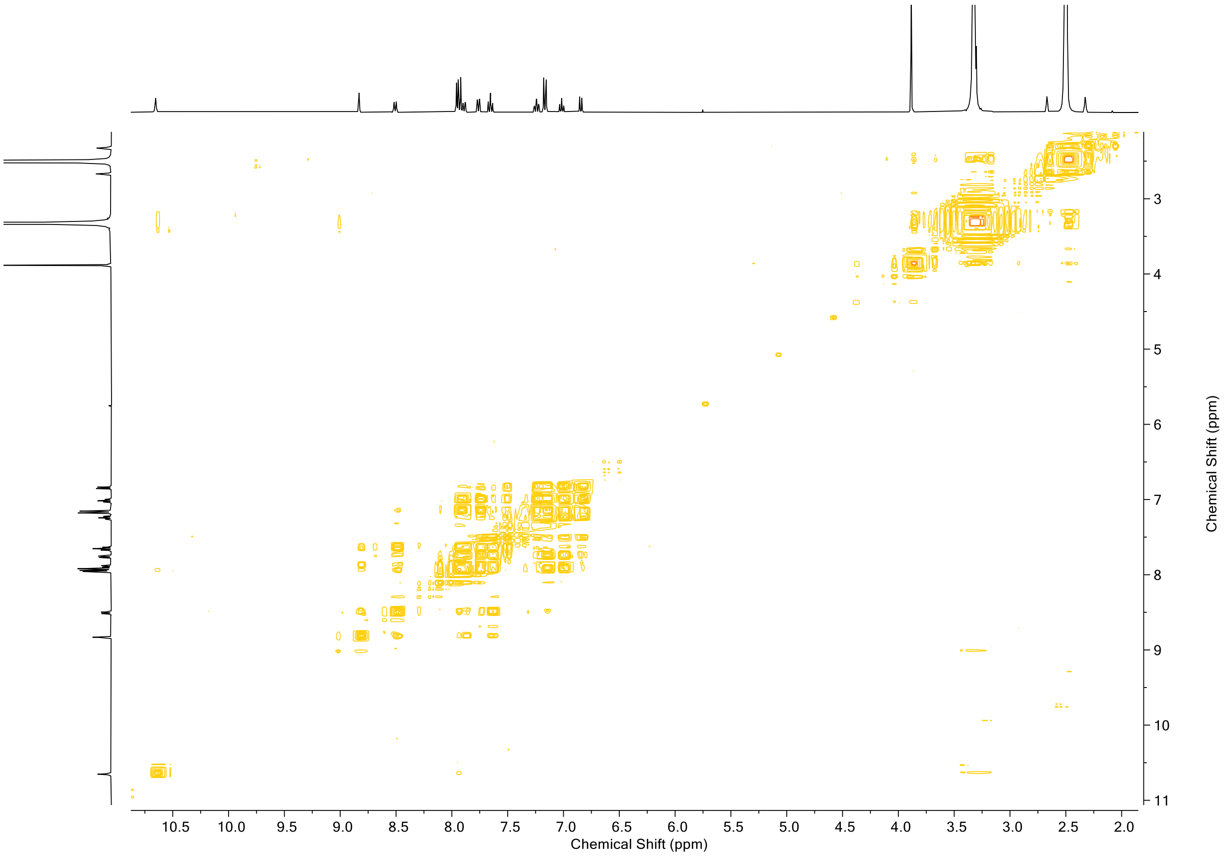

Figure S148: COSY spectrum of ***EZ*-7** ((CD$_3$)$_2$SO).

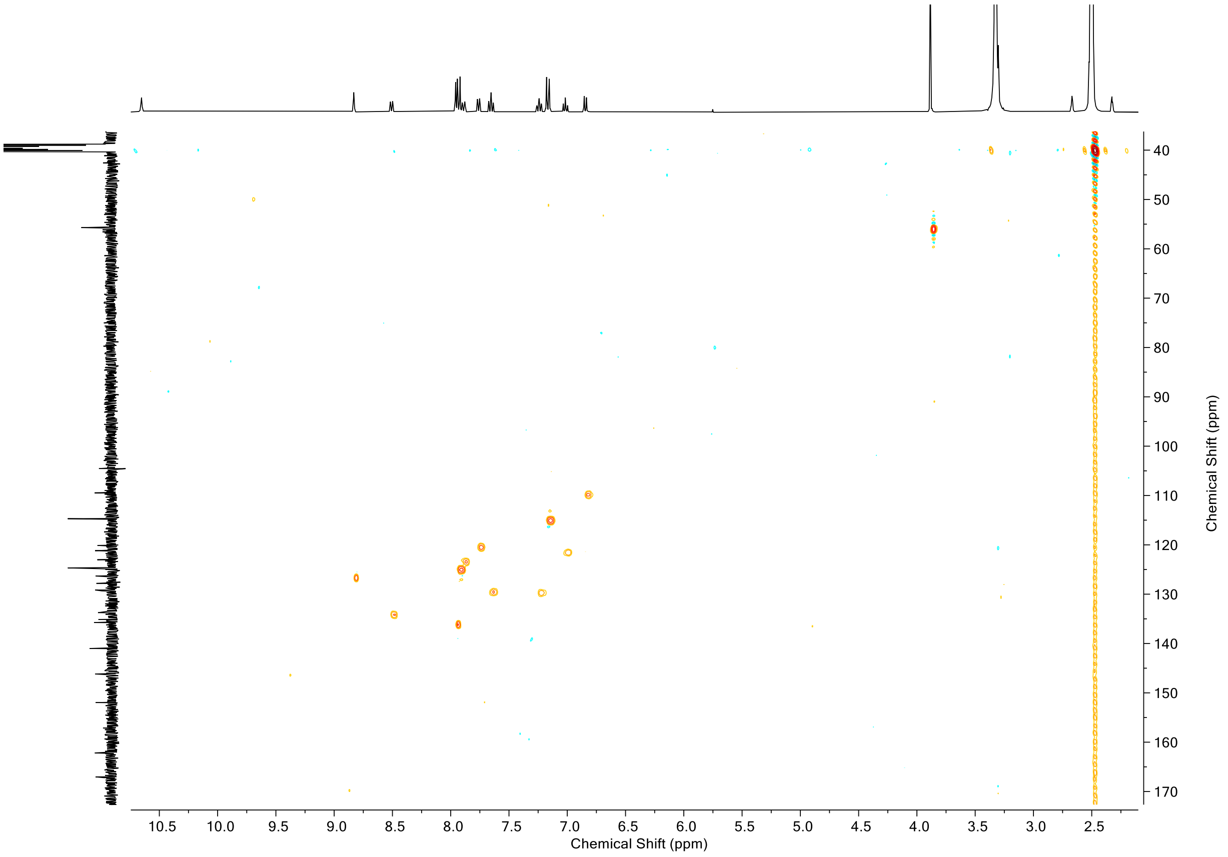

Figure S149: HSQC spectrum of ***EZ*-7** ((CD$_3$)$_2$SO).

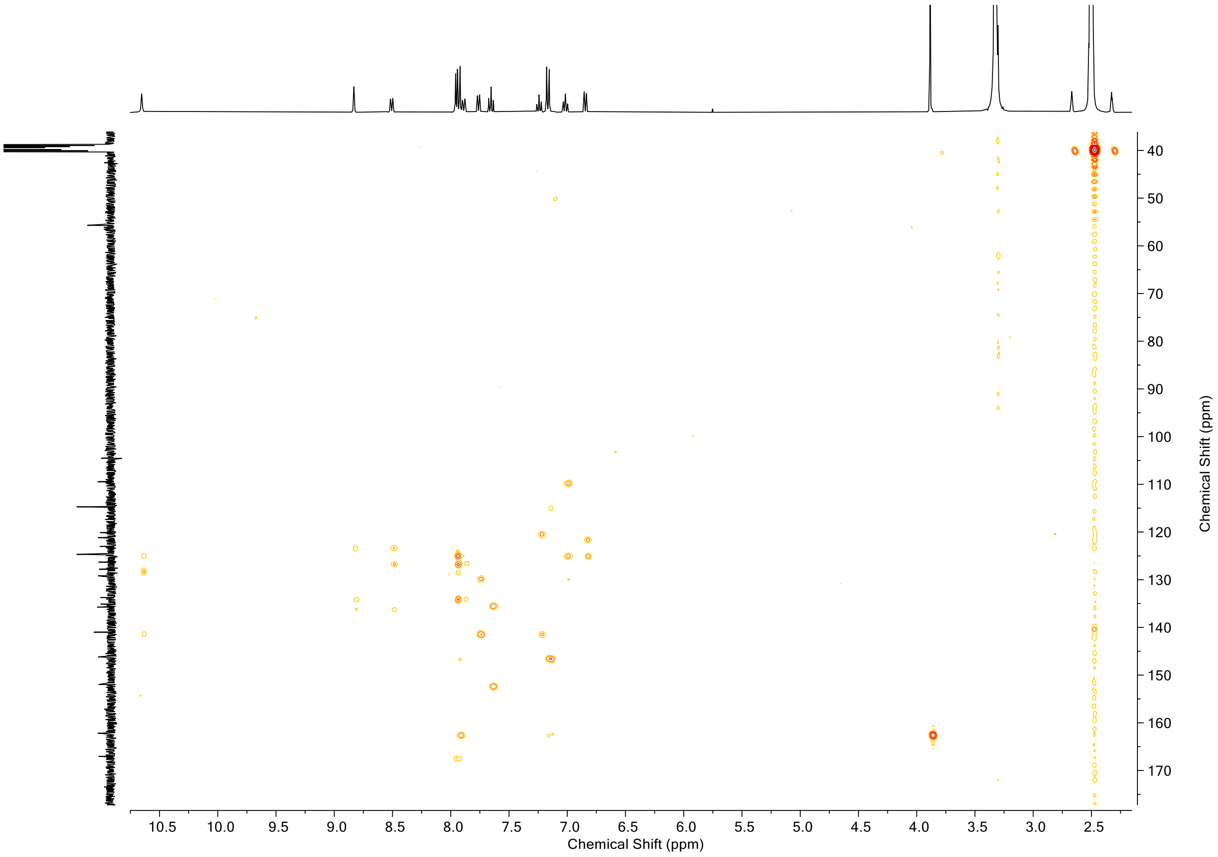

Figure S150: HMBC spectrum of ***EZ*-7** (($(CD_3)_2SO$).

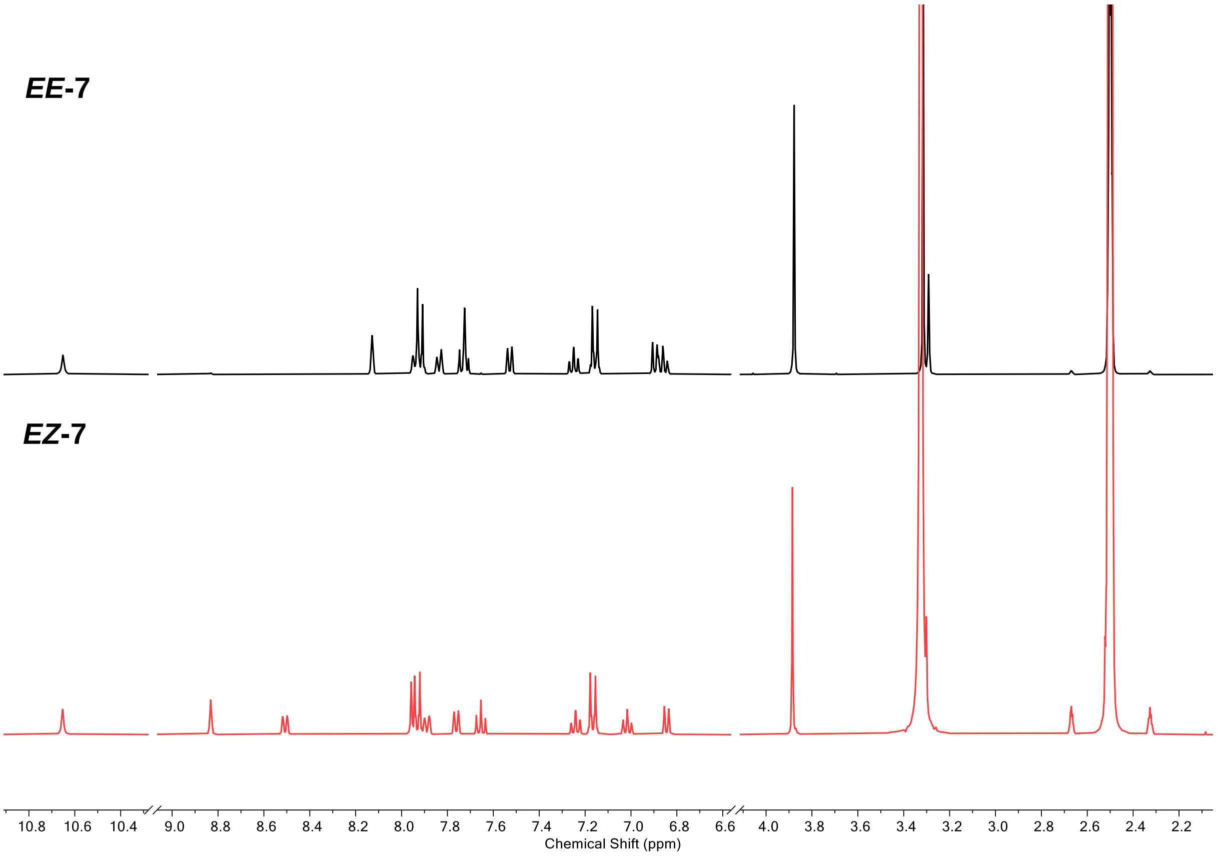

Figure S151: Comparison of **^{1}H NMR** spectra of ***EE*-7** (black) and ***EZ*-7** (red) (($(CD_3)_2SO$).
